# A Facilitated Peer Mentoring Program With a Dedicated Curriculum to Foster Career Advancement of Academic Hospitalists

**DOI:** 10.15766/mep_2374-8265.11366

**Published:** 2023-12-08

**Authors:** Doris Lin, R. Michelle Schmidt, Chirayu Shah, Andrew Caruso, Xiaofan Huang, Kristen A. Staggers, Joslyn Fisher

**Affiliations:** 1 Associate Professor, Department of Medicine, Baylor College of Medicine; 2 Assistant Professor, Department of Medicine, Michael E. DeBakey Veterans Affairs Medical Center and Baylor College of Medicine; 3 Statistician, Institute for Clinical and Translational Research, Baylor College of Medicine

**Keywords:** Academic Hospitalists, Career Advancement, Facilitated Peer Mentoring, Faculty Development, Hospital Medicine, Internal Medicine, Mentoring/Coaching, Quantitative Research, Self-Assessment

## Abstract

**Introduction:**

In the field of hospital medicine, there is both a limited pool of senior faculty to mentor the rapidly growing number of junior faculty and a lack of career development curricula focused on scholarly activities specific to the needs of the hospitalist. These deficits have resulted in a disproportionately low number of academic hospitalists being promoted to associate and full professor. We implemented a facilitated peer mentoring program with a dedicated curriculum to foster career advancement of academic hospitalists.

**Methods:**

We recruited 29 academic hospitalists and divided them into five small groups, each guided by one senior faculty. Peer members participated in a 9-month curriculum consisting of alternating large- and small-group sessions that reviewed topics important for academic advancement. Quantitative analysis assessed feasibility of the program, as measured by participation and knowledge improvement on curriculum topics, with pre- and postprogram surveys.

**Results:**

Results demonstrated feasibility of the large-group sessions as measured through participation. Small-group participation was more variable. Pre- and postsurvey results showed significant knowledge improvement (*p* < .05) in nearly all of the curriculum topics.

**Discussion:**

Currently, there is a gap in both mentorship and scholarly skills of academic hospitalists. Our facilitated peer mentoring program with a dedicated curriculum can be used as a framework for other hospitalist programs to support career development.

## Educational Objectives

By the end of this activity, learners will be able to:

1. Explain the purpose of peer mentoring for academic hospitalists.
2. Identify their individual career goals.
3. Describe where to obtain the institutional resources on curriculum vitae (CV) development, promotion pathways, and educational and clinical portfolios.
4. Describe the institutional CV structure, promotion pathways, and hospitalist activities to include in an academic CV and portfolio.
5. Select at least two article types they have the skills to write and conferences that accept abstract submissions.

## Introduction

Historically, mentorship has been a cornerstone in career advancement of academic faculty. Successful mentorship has been linked to improved faculty retention,^1,2^ scholarly productivity,^3,4^ reduction in time to promotion,^5^ and increased career satisfaction.^6^ One of the common mentoring models described in the literature is traditional dyadic mentoring, where a senior faculty member guides a less experienced junior mentee. For academic hospitalists, however, this model poses a significant challenge as there are more early-career clinicians than available senior mentors, so one-on-one mentoring is often not achievable. In order to overcome this barrier, peer mentoring has been recognized as an alternative to the dyadic model. Peer mentoring engages those who have similar rank and interest to collaborate and work together toward career advancement. Several peer models have demonstrated positive results, including improvement in personal and professional growth, social networks, and academic productivity.^7–9^ Furthermore, peer mentoring can be advantageous to clinician educators, a career in which most academic hospitalists will find themselves.

In addition to lack of mentorship, many academic hospitalists begin their careers with few scholarly skills as they start working immediately after residency without receiving training to develop and write original research-type articles. Academic hospitalists often work as clinician educators, a role in which most funded effort typically focuses on patient care and clinical productivity despite increasing demands for participation in less supported activities such as learner education and scholarly effort.^10,11^ Since scholarship remains a key to career success in academic institutions, lack of time and experience to produce publications is a detriment to promotion and career advancement. As a result, there is currently a critical gap in promotion, as demonstrated by a recent study that evaluated 1,500 academic hospitalists at the top 25 US internal medicine residencies and found that only 9% achieved associate professor and 3% advanced to full professor rank.^12^

Our hospitalist program consists of over 100 hospitalists and mirrors other programs across the country with a high number of junior faculty and relatively few senior faculty. To overcome both barriers—lack of mentorship and scholarly skills—we developed a facilitated peer mentoring program incorporating a dedicated curriculum educating members on the purpose of peer mentoring, resources for career advancement, and types of scholarly activities that might be more readily achievable for busy hospitalists. Currently, scant literature exists that focuses specifically on peer mentoring of academic hospitalists. Several mentoring publications have been published in *MedEdPORTAL*; however, these concentrate on improving mentoring relationships^13,14^ and peer mentorship of academic faculty in general.^15,16^ Additionally, we found only one article in PubMed describing the implementation of a facilitated peer mentoring model specific to hospitalists.^17^ That program was a small, single-center, qualitative study focusing on career goals and opportunities within hospital medicine. We expanded upon this work to include exploration of other domains. Our aims were to (1) determine feasibility of the program as measured through participation, (2) enhance knowledge of curriculum-specific topics, and (3) evaluate participants’ perception regarding efficacy for their personal career advancement.

## Methods

Academic hospitalists practicing at three affiliate hospitals (safety net, Veterans Affairs, private university) received an email invitation to participate. The email described the program aims, curriculum, time commitment, target audience (junior faculty at the level of instructor or assistant professor and preferably in their position for fewer than 6 years), and voluntary nature of participation. The primary author and the section chief met prior to the launch of the program to identify faculty who possessed the skills and experience necessary to serve as senior mentor-facilitators. Five agreed to lead their own small group and received a brief overview of the aims, structure, and curriculum. From the email solicitation, we recruited 29 faculty and subsequently divided them into five small groups, each consisting of four to seven peer members and one senior facilitator. We grouped mentees to optimally match clinical and scholarly interests. Each peer participant completed electronic pre- and postprogram surveys (Appendices A and B, respectively), which were sent to their email prior to the start and upon completion of the program. The Baylor College of Medicine Institutional Review Board approved the project.

Power and sample size were determined prior to the start of the program. To consider this program feasible, we aimed to achieve an average participation rate of at least 50% in the large- and small-group sessions, assuming that the actual participation rate would be closer to 70%. Although we would have liked 100% attendance at all sessions, we decided a more realistic participation rate of at least 50% would signify that the program was feasible because we wanted the peer members to attend at least two of the four large-group sessions and two of the three small-group sessions. A one-sided noninferiority *z* test for a binomial proportion would have 87% power to reject the null hypothesis that the participation rate is inferior to 50% (margin of −10%) with 20 participants at a 5% significance level. Our feasibility analysis was based on 27 participants (two mentees officially withdrew), so our project was sufficiently powered to test the primary aim. The second aim assessed knowledge improvement on curriculum topics using a 5-point Likert scale, and we compared pre- and postprogram survey scores using the Wilcoxon signed rank test. We summarized survey responses by median with 25th and 75th percentiles, or frequency with percentage, to look at the entire distribution of the Likert scale.

Two of the authors developed a dedicated curriculum consisting of four PowerPoint presentations: (1) Introduction to Facilitated Peer Mentorship and Identifying Career Goals, (2) Building a Curriculum Vitae (CV), (3) Pathways to Promotion and Introduction to Educational and Patient Care/Clinical Portfolios, and (4) Strategies for Hospitalist Educators to Achieve Scholarly Success (Appendices C–F, respectively). We presented each session to all members in a large-group setting. Each large-group presentation was 50 minutes, with an additional 10 minutes at the end for questions. Session 1 introduced the concept of facilitated peer mentoring, reviewed the program objectives and timeline, and ended with identification of individual career goals (Appendix C). Session 2 defined the CV and provided tips on building one (Appendix D). Session 3 reviewed institutional promotion pathways, the structure of academic and clinical portfolios, and activities to include in each type (Appendix E). Prior to sessions 2 and 3, we obtained CVs and portfolios from colleagues within the hospitalist group in order to share examples of how to incorporate hospitalist-based activities into a CV and portfolio. Showing examples was an important aspect of the large-group sessions, so future presenters should obtain CVs and portfolios to demonstrate examples of hospitalist-specific activities tailored to their institution. Senior facilitators and presenters will need to familiarize themselves with their own institutional promotion pathways and portfolios as sessions 2 and 3 should be tailored to institutional criteria and templates. Session 4 reviewed clinician educator prototypes, considered types of articles that might be more feasible for hospitalist educators, and offered high-yield tips on manuscript submissions and generating scholarship (Appendix F). We have provided talking points for presenters in the notes section of the slides.

After each large-group session, the presenter instructed peer members to begin work on what had been discussed in the prior large group. The presenter then instructed the small groups to meet approximately 4–6 weeks after each large-group session in order to review, continue work on previously presented topics, and have the senior facilitator available to answer questions. Senior mentors utilized facilitator guides (Appendices G–I) that provided activities for each of the corresponding small-group sessions. The overall program structure is shown in the Figure. All sessions occurred over a 9-month period from August 2021 to April 2022 with alternating large- and small-group sessions. Program duration can be shortened or lengthened at the discretion of program organizers/senior facilitators.

**Figure.**
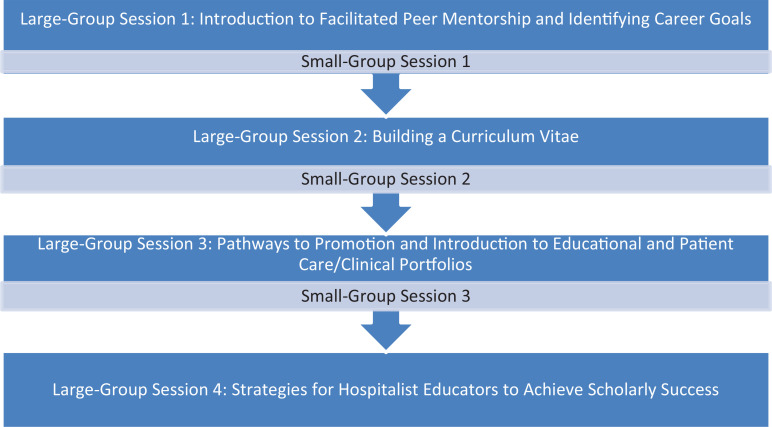
Facilitated peer mentoring program structure.

## Results

Although 29 faculty initially joined the program as peer mentees, two members withdrew shortly after the program began, one due to lack of time and another for personal leave. Another four joined but ultimately did not participate in any of the large- or small-group sessions. These four were included in the analysis for feasibility since they did not officially withdraw. The average participation rate for the large-group sessions was 57%, which was noninferior to 50% with a margin of −10% (*p* = .04), demonstrating feasibility, which was the initial aim of the project. Participation in the small groups was more variable. One small group met three times, with participation in each of the sessions >70%. Another group met once, with all members in attendance. The facilitator of this group then met individually with two of the more engaged members throughout the study period. The other three senior facilitators met individually with peer members. Senior facilitators and participants cited schedule conflicts and limited time as barriers to meeting with the full small group.

The second aim assessed knowledge improvement on curriculum topics utilizing pre- and postprogram surveys. A total of 19 participants completed both pre- and postprogram surveys. Overall, there was a statistically significant increase in participants’ self-rated knowledge of where to obtain resources on CV development (*p* = .02), promotion pathways (*p* = .004), and educator (*p* = .001) and patient care (*p* = .03) portfolios (Table 1). Important links for these resources, as well as where they could be located on the institutional website, were emphasized in the large-group sessions. Data from self-assessment surveys at baseline and after the program are shown in Table 2. Although satisfaction with academic rank and accomplishments did not change significantly, this could have been a reflection of the high number of participants who were still early in their career. Before the program began, most agreed or strongly agreed that participation in a facilitated peer mentoring program would assist in career advancement and interest in becoming an effective mentor, which is the reason there was no significant change to these questions postprogram. Upon completion of the program, participants demonstrated significant improvement in knowledge of the institutional CV structure (*p* = .009); activities to include in the CV (*p* = .02); promotion pathways (*p* = .008); criteria to apply for promotion (*p* = .01); portfolio categories (*p* = .01), specifically a patient care portfolio (*p* = .002); activities that would count towards each portfolio (*p* = .002); article types they had the skills to write (*p* = .02); how to submit a manuscript (*p* = .02); and conferences accepting abstract submissions (*p* = .02). Those who felt they had a career goal also improved (*p* = .02). Moreover, 84% of participants (*n* = 16) were satisfied with the program, 79% (*n* = 15) felt participation would help in achieving career goals, and 95% (*n* = 18) would recommend the program to their colleagues.

**Table 1.**
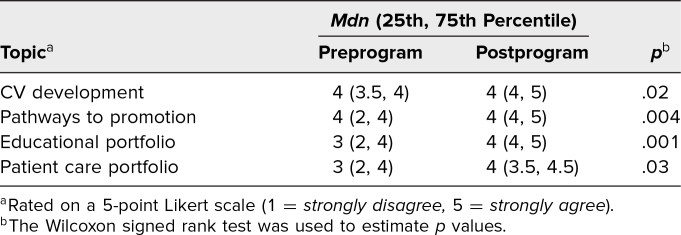
Responses to “I Know Where to Obtain the Resources Available on These Topics” (*N* = 19)

**Table 2.**
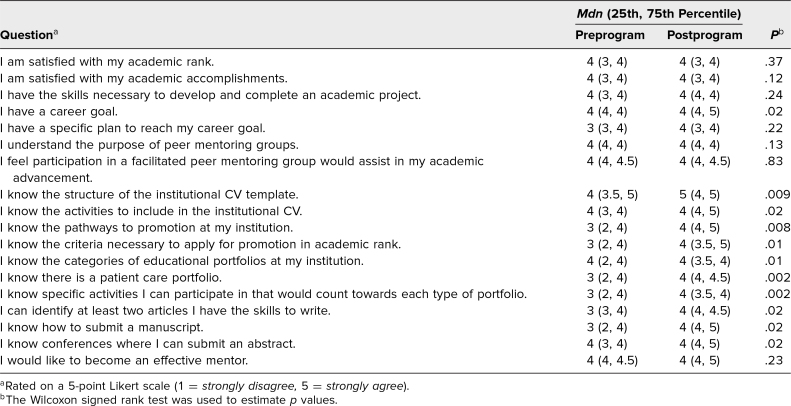
Responses to Self-Assessment Survey (*N* = 19)

## Discussion

We developed and implemented an innovative facilitated peer mentoring program with its own dedicated curriculum to overcome current gaps including a dearth of senior mentors, lack of skills to produce scholarly work, and the need to better prepare faculty for academic promotion. Overcoming these challenges is necessary to cultivate successful academic hospitalist programs.^18–20^ Our program provided early-career faculty with much-needed education on CV development, pathways to promotion, and scholarly work that might be more feasible for busy clinicians. Although our institution offers workshops on some of these topics for all faculty, we sought to make our large- and small-group sessions more personalized to hospitalists, coupling them with support and guidance from peers and senior faculty. Compared to other peer programs described in the literature, our program was distinct as we also incorporated a hospitalist-based curriculum detailing opportunities for career development through peer mentorship as well as promotion of scholarship. Results demonstrated feasibility of the large-group sessions as measured through participation. Additionally, the surveys showed significant knowledge improvement (*p* < .05) in nearly all of the curriculum topics addressed in the sessions. Small-group participation, however, was not as robust.

We recognize a few limitations to the project. One limitation was the use of a Likert scale, which, although commonly used in educational research, can be influenced by acquiescence bias where participants are likely to agree regardless of how they feel. A follow-up evaluation to determine if participants used the information for their own career advancement and long-term assessment of scholarly productivity and success in academic promotion among peer members would give a more realistic evaluation of the program. Second, our results demonstrated self-reported knowledge improvement instead of objective assessment. Adding a pre- and postprogram summative assessment would provide a better reflection of actual knowledge improvement. Lastly, we experienced significant variability in small-group participation, which we acknowledge as a negative impact to a peer mentoring program. Both senior facilitators and peer mentees cited lack of time due to administrative and clinical duties as a major barrier to meeting together in their small groups. With no dedicated funding or time to support participation, a well-documented barrier to mentoring programs,^3,19,21^ both junior and senior faculty participated voluntarily.

We plan to use the lessons learned from implementation of the program for future iterations. First, we plan to develop more structured guidelines outlining the time commitment required for both senior facilitators and junior peer members. Since institutional and/or departmental funding for this type of activity is typically minimal to nonexistent, it would be worthwhile to acknowledge these voluntary efforts in other ways, such as awarding plaques of appreciation to senior facilitators and providing mentees with certificates of completion that can be listed in their CVs. Second, scheduling small groups well in advance with all members agreeing to preset dates and times may improve attendance and participation. Third, early concurrence of each small group's objective(s) could facilitate more effective use of small-group efforts. For example, one peer group might focus on CV preparation while another collaborates on a publication. Emphasizing the importance of peer participation, information sharing, and prior preparation of questions and materials to be discussed in the small groups is imperative to developing a successful program. Lastly, intermittent assessment and reorganization of the groups may be helpful since individual mentee interests can change over time.

Facilitated peer group mentoring is a low-cost, replicable solution for academic mentoring when gaps exist in the availability of traditional mentor-mentee dyads. Our program was unique as we also incorporated a dedicated curriculum to meet the demands of the busy hospitalist. The large-group sessions provided essential information on topics necessary for career advancement in an academic setting. The small groups allowed for peer collaboration with senior guidance. The sessions can be easily incorporated into any hospitalist program in their entirety or as separate sessions for faculty development. They can also be easily tailored to other specialties where peer mentoring may be advantageous. We view this program as a starting point to narrowing the gap of academic rank among hospitalists.

## Appendices


Preprogram Survey.docxPostprogram Survey.docxLarge-Group Session 1.pptxLarge-Group Session 2.pptxLarge-Group Session 3.pptxLarge-Group Session 4.pptxSmall-Group Session 1 Facilitator Guide.docxSmall-Group Session 2 Facilitator Guide.docxSmall-Group Session 3 Facilitator Guide.docx
*All appendices are peer reviewed as integral parts of the Original Publication.*
