# Supplementary material for: A Facilitated Peer Mentoring Program With a Dedicated Curriculum to Foster Career Advancement of Academic Hospitalists
Source: MedEdPORTAL. 2023 Dec 8;19:11366. doi: 10.15766/mep_2374-8265.11366 (PMC10704005; doi:10.15766/mep_2374-8265.11366)
Supplement: Supplementary file 1 — Preprogram Survey.docxPostprogram Survey.docxLarge-Group Session 1.pptxLarge-Group Session 2.pptxLarge-Group Session 3.pptxLarge-Group Session 4.pptxSmall-Group Session 1 Facilitator Guide.docxSmall-Group Session 2 Facilitator Guide.docxSmall-Group Session 3 Facilitator Guide.docx [file mep_2374-8265.11366-s001.zip › A. Preprogram Survey.docx]

**Appendix A- Preprogram Survey**

1. What year did you start working in the Section of General Internal Medicine?
2. What is your current academic title? Instructor, Assistant Professor, Associate Professor
3. Do you currently have a professional mentor(s)? If so, please describe:
4. Please choose your level of agreement with the following statements:

|  | Strongly Disagree | Disagree | Neutral | Agree | Strongly Agree |
| --- | --- | --- | --- | --- | --- |
| I am satisfied with my academic rank | 1 | 2 | 3 | 4 | 5 |
| I am satisfied with my academic accomplishments | 1 | 2 | 3 | 4 | 5 |
| I have the skills necessary to develop and complete an academic project | 1 | 2 | 3 | 4 | 5 |
| I understand the purpose of peer mentoring groups | 1 | 2 | 3 | 4 | 5 |
| I feel participation in a facilitated peer mentoring group would assist in my academic advancement | 1 | 2 | 3 | 4 | 5 |
| I have a career goal | 1 | 2 | 3 | 4 | 5 |
| I have a specific plan to reach my career goal | 1 | 2 | 3 | 4 | 5 |
| I would like to become an effective mentor | 1 | 2 | 3 | 4 | 5 |

1. Please list any current barriers to accomplishing your career goals.
2. I know where to obtain the resources available on these topics:

|  | Strongly Disagree | Disagree | Neutral | Agree | Strongly Agree |
| --- | --- | --- | --- | --- | --- |
| Curriculum Vitae (CV) Development | 1 | 2 | 3 | 4 | 5 |
| Pathways to Promotion | 1 | 2 | 3 | 4 | 5 |
| Educational Portfolios | 1 | 2 | 3 | 4 | 5 |
| Patient Care Portfolio | 1 | 2 | 3 | 4 | 5 |

|  | Strongly Disagree | Disagree | Neutral | Agree | Strongly Agree |
| --- | --- | --- | --- | --- | --- |
| I know the structure of the institutional CV template | 1 | 2 | 3 | 4 | 5 |
| I know the activities to include in the institutional CV | 1 | 2 | 3 | 4 | 5 |
| I know the pathways to promotion at my institution | 1 | 2 | 3 | 4 | 5 |
| I know the criteria necessary to apply for promotion in academic rank | 1 | 2 | 3 | 4 | 5 |
| I know the categories of educational portfolios at my institution | 1 | 2 | 3 | 4 | 5 |
| I know there is a patient care portfolio | 1 | 2 | 3 | 4 | 5 |
| I know specific activities I can participate in that would count towards each type of portfolio | 1 | 2 | 3 | 4 | 5 |
| I can identify at least 2 article types I have the skills to write | 1 | 2 | 3 | 4 | 5 |
| I know how to submit a manuscript | 1 | 2 | 3 | 4 | 5 |
| I know conferences where I can submit an abstract | 1 | 2 | 3 | 4 | 5 |

1. Please choose your level of agreement with the following statements:
