# Supplementary material for: A Facilitated Peer Mentoring Program With a Dedicated Curriculum to Foster Career Advancement of Academic Hospitalists
Source: MedEdPORTAL. 2023 Dec 8;19:11366. doi: 10.15766/mep_2374-8265.11366 (PMC10704005; doi:10.15766/mep_2374-8265.11366)
Supplement: Supplementary file 1 — Preprogram Survey.docxPostprogram Survey.docxLarge-Group Session 1.pptxLarge-Group Session 2.pptxLarge-Group Session 3.pptxLarge-Group Session 4.pptxSmall-Group Session 1 Facilitator Guide.docxSmall-Group Session 2 Facilitator Guide.docxSmall-Group Session 3 Facilitator Guide.docx [file mep_2374-8265.11366-s001.zip › B. Postprogram Survey.docx]

**Appendix B- Post-Program Survey**

1. What is your current academic title? Instructor, Assistant Professor, Associate Professor
2. Do you currently have a professional mentor(s)? If so, please describe:
3. Please choose your level of agreement with the following statements:

|  | Strongly Disagree | Disagree | Neutral | Agree | Strongly Agree |
| --- | --- | --- | --- | --- | --- |
| I am satisfied with my academic rank | 1 | 2 | 3 | 4 | 5 |
| I am satisfied with my academic accomplishments | 1 | 2 | 3 | 4 | 5 |
| I have the skills necessary to develop and complete an academic project | 1 | 2 | 3 | 4 | 5 |
| I understand the purpose of peer mentoring groups | 1 | 2 | 3 | 4 | 5 |
| I feel participation in a facilitated peer mentoring group would assist in my academic advancement | 1 | 2 | 3 | 4 | 5 |
| I have a career goal | 1 | 2 | 3 | 4 | 5 |
| I have a specific plan to reach my career goal | 1 | 2 | 3 | 4 | 5 |
| I would like to become an effective mentor | 1 | 2 | 3 | 4 | 5 |

1. Please list any current barriers to accomplishing your career goals.
2. I know where to obtain the resources available on these topics:

|  | Strongly Disagree | Disagree | Neutral | Agree | Strongly Agree |
| --- | --- | --- | --- | --- | --- |
| Curriculum Vitae (CV) Development | 1 | 2 | 3 | 4 | 5 |
| Pathways to Promotion | 1 | 2 | 3 | 4 | 5 |
| Educational Portfolios | 1 | 2 | 3 | 4 | 5 |
| Patient Care Portfolio | 1 | 2 | 3 | 4 | 5 |

|  | Strongly Disagree | Disagree | Neutral | Agree | Strongly Agree |
| --- | --- | --- | --- | --- | --- |
| I know the structure of the institutional CV template | 1 | 2 | 3 | 4 | 5 |
| I know the activities to include in the institutional CV | 1 | 2 | 3 | 4 | 5 |
| I know the pathways to promotion at my institution | 1 | 2 | 3 | 4 | 5 |
| I know the criteria necessary to apply for promotion in academic rank | 1 | 2 | 3 | 4 | 5 |
| I know the categories of educational portfolios at my institution | 1 | 2 | 3 | 4 | 5 |
| I know there is a patient care portfolio | 1 | 2 | 3 | 4 | 5 |
| I know specific activities I can participate in that would count towards each type of portfolio | 1 | 2 | 3 | 4 | 5 |
| I can identify at least 2 article types I have the skills to write | 1 | 2 | 3 | 4 | 5 |
| I know how to submit a manuscript | 1 | 2 | 3 | 4 | 5 |
| I know conferences where I can submit an abstract | 1 | 2 | 3 | 4 | 5 |

1. Please choose your level of agreement with the following statements:
2. Please rate your level of satisfaction with the facilitated peer mentoring program
3. Very unsatisfied
4. Unsatisfied
5. Neutral
6. Satisfied
7. Very satisfied
8. Participation in the peer mentoring program has helped me in achieving my career goals
   1. Strongly disagree
   2. Disagree
   3. Neutral
   4. Agree
   5. Strongly agree
9. Would you recommend the program to your colleagues? Yes or No
10. Do you have any suggestions/comments that will help us make the program better?
