# Supplementary material for: A Facilitated Peer Mentoring Program With a Dedicated Curriculum to Foster Career Advancement of Academic Hospitalists
Source: MedEdPORTAL. 2023 Dec 8;19:11366. doi: 10.15766/mep_2374-8265.11366 (PMC10704005; doi:10.15766/mep_2374-8265.11366)
Supplement: Supplementary file 1 — Preprogram Survey.docxPostprogram Survey.docxLarge-Group Session 1.pptxLarge-Group Session 2.pptxLarge-Group Session 3.pptxLarge-Group Session 4.pptxSmall-Group Session 1 Facilitator Guide.docxSmall-Group Session 2 Facilitator Guide.docxSmall-Group Session 3 Facilitator Guide.docx [file mep_2374-8265.11366-s001.zip › F. Large-Group Session 4.pptx]

## Slide 1
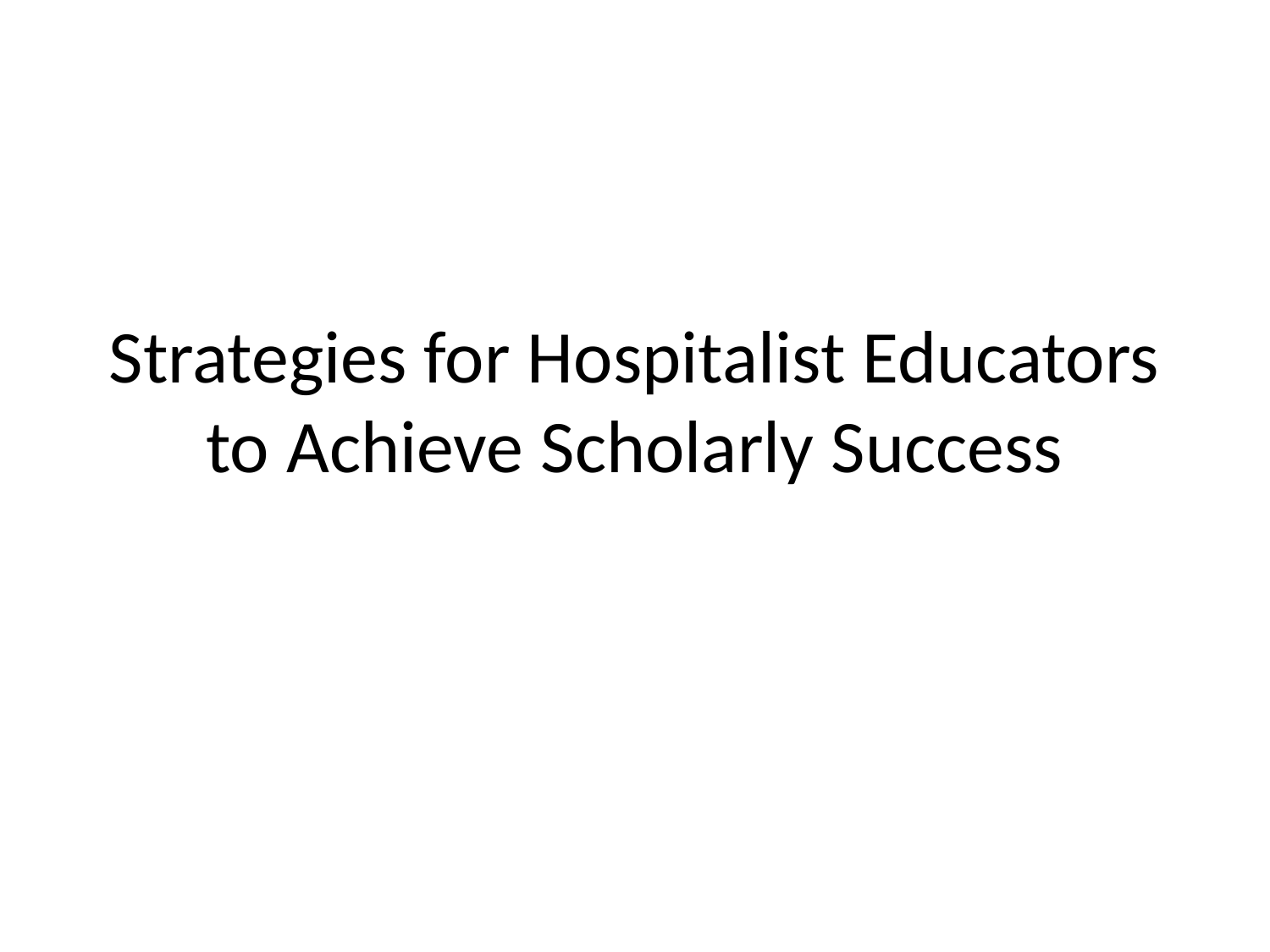

# Strategies for Hospitalist Educators to Achieve Scholarly Success

## Slide 2
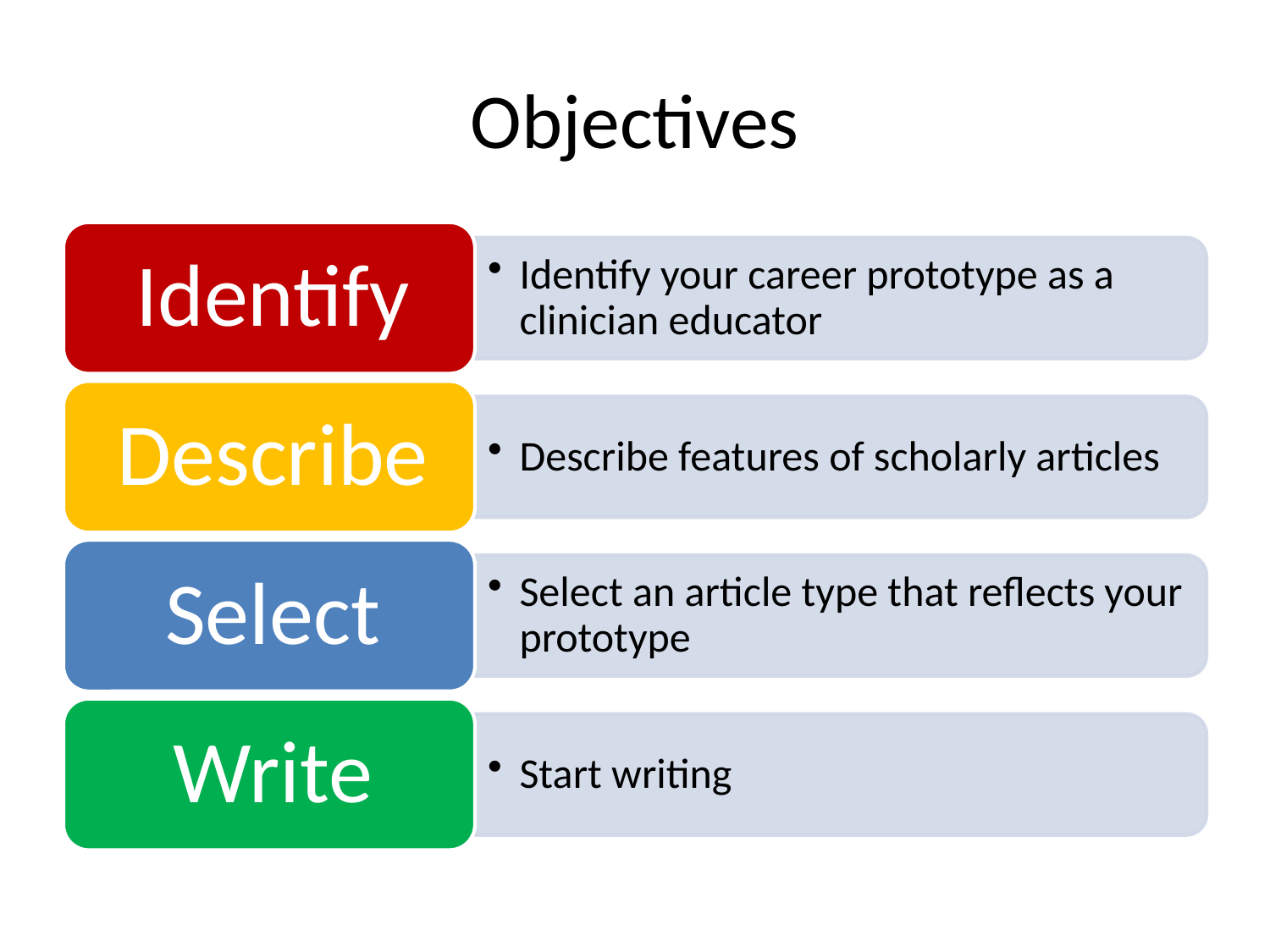

# Objectives

## Slide 3
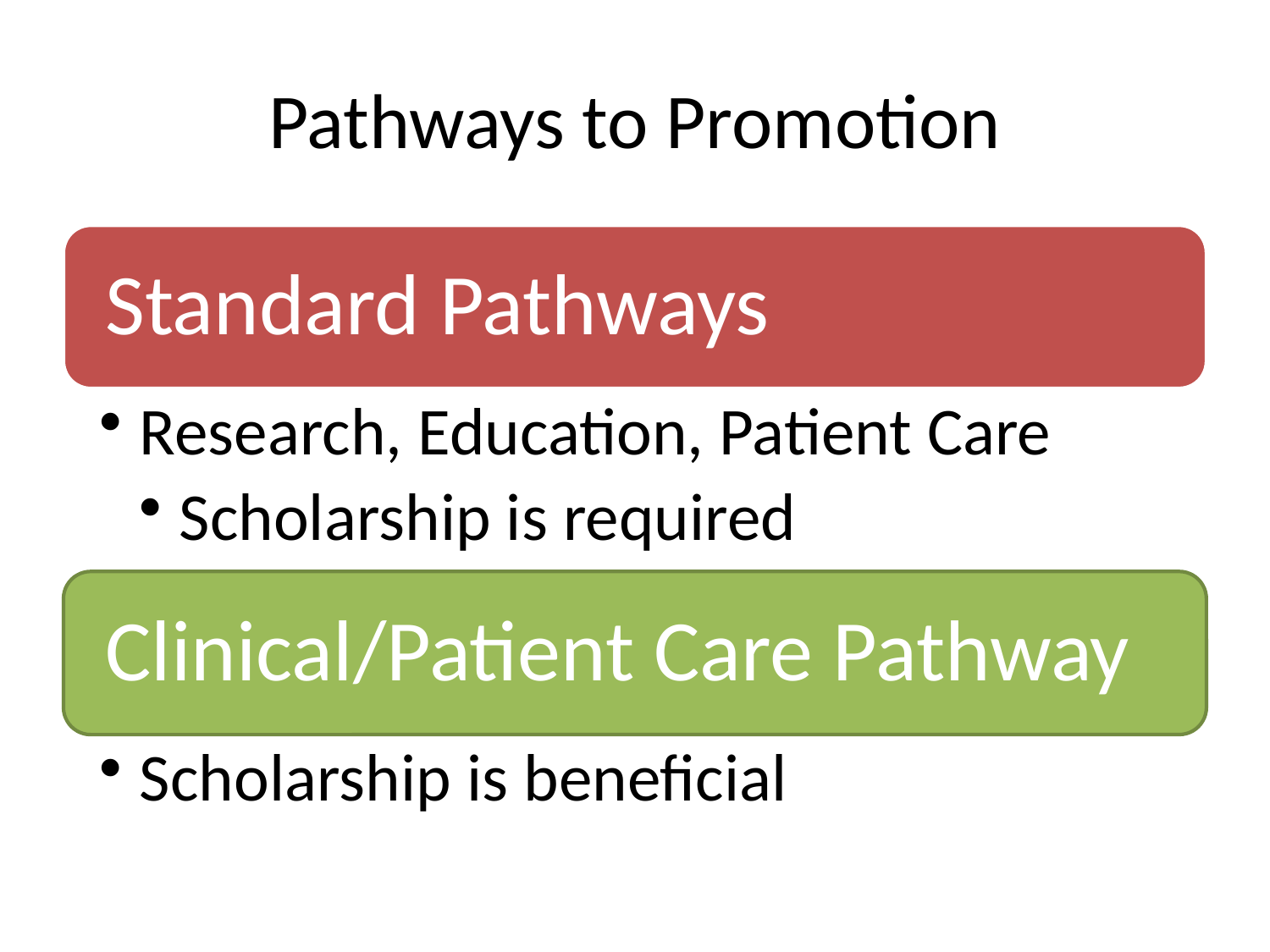

# Pathways to Promotion

## Slide 4
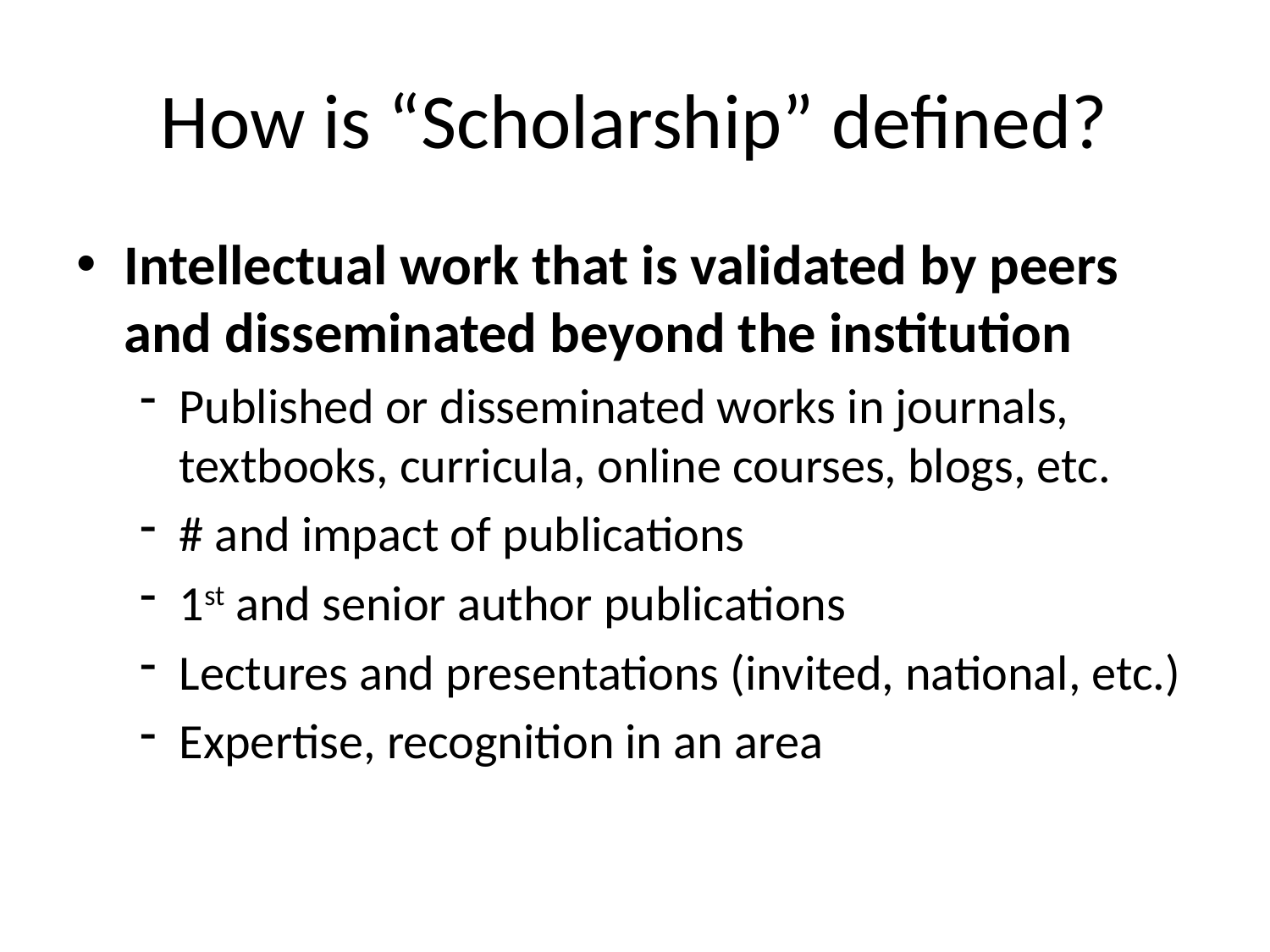

# How is “Scholarship” defined?
Intellectual work that is validated by peers and disseminated beyond the institution
Published or disseminated works in journals, textbooks, curricula, online courses, blogs, etc.
# and impact of publications
1st and senior author publications
Lectures and presentations (invited, national, etc.)
Expertise, recognition in an area

## Slide 5
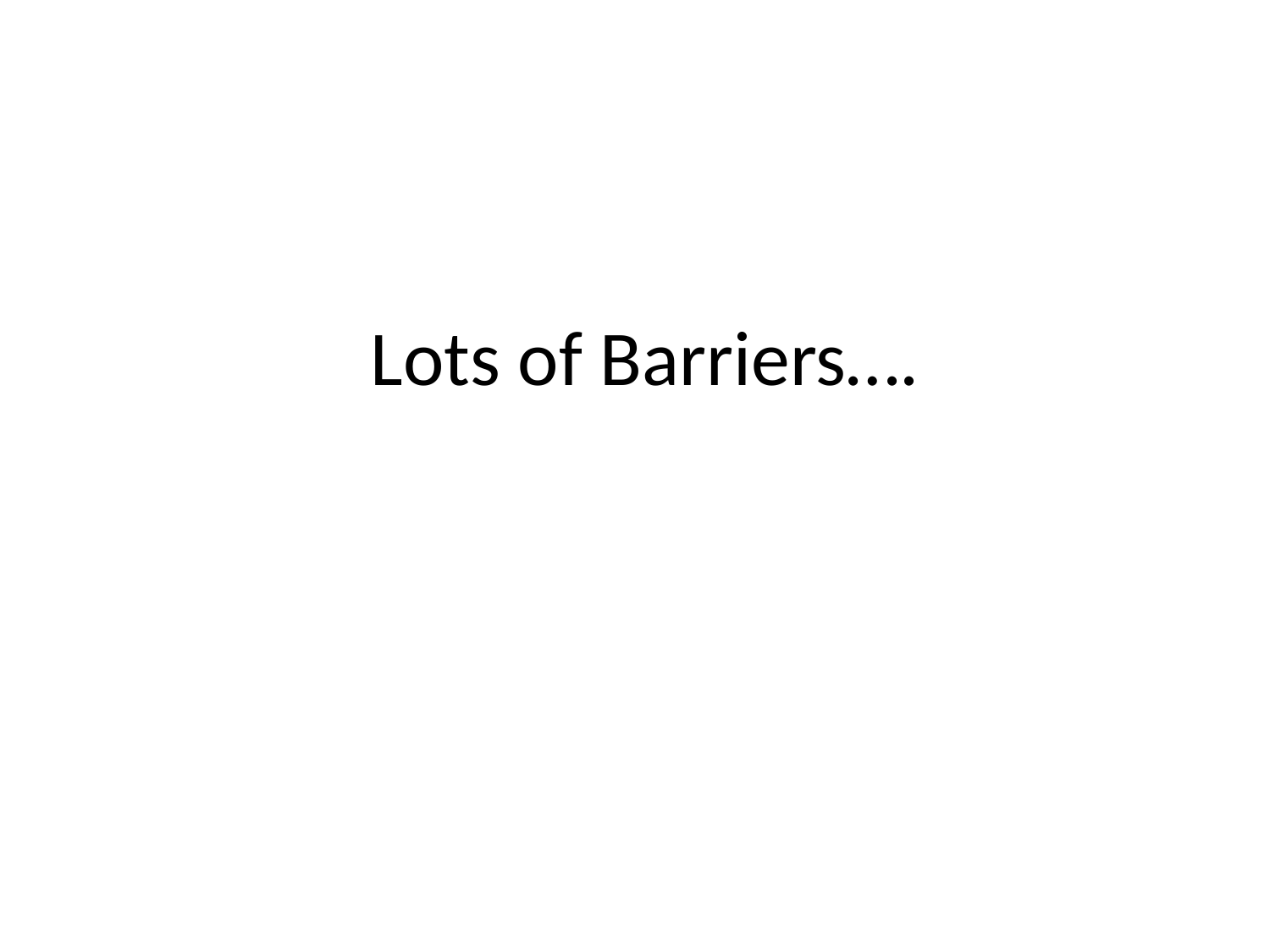

# Lots of Barriers….

## Slide 6
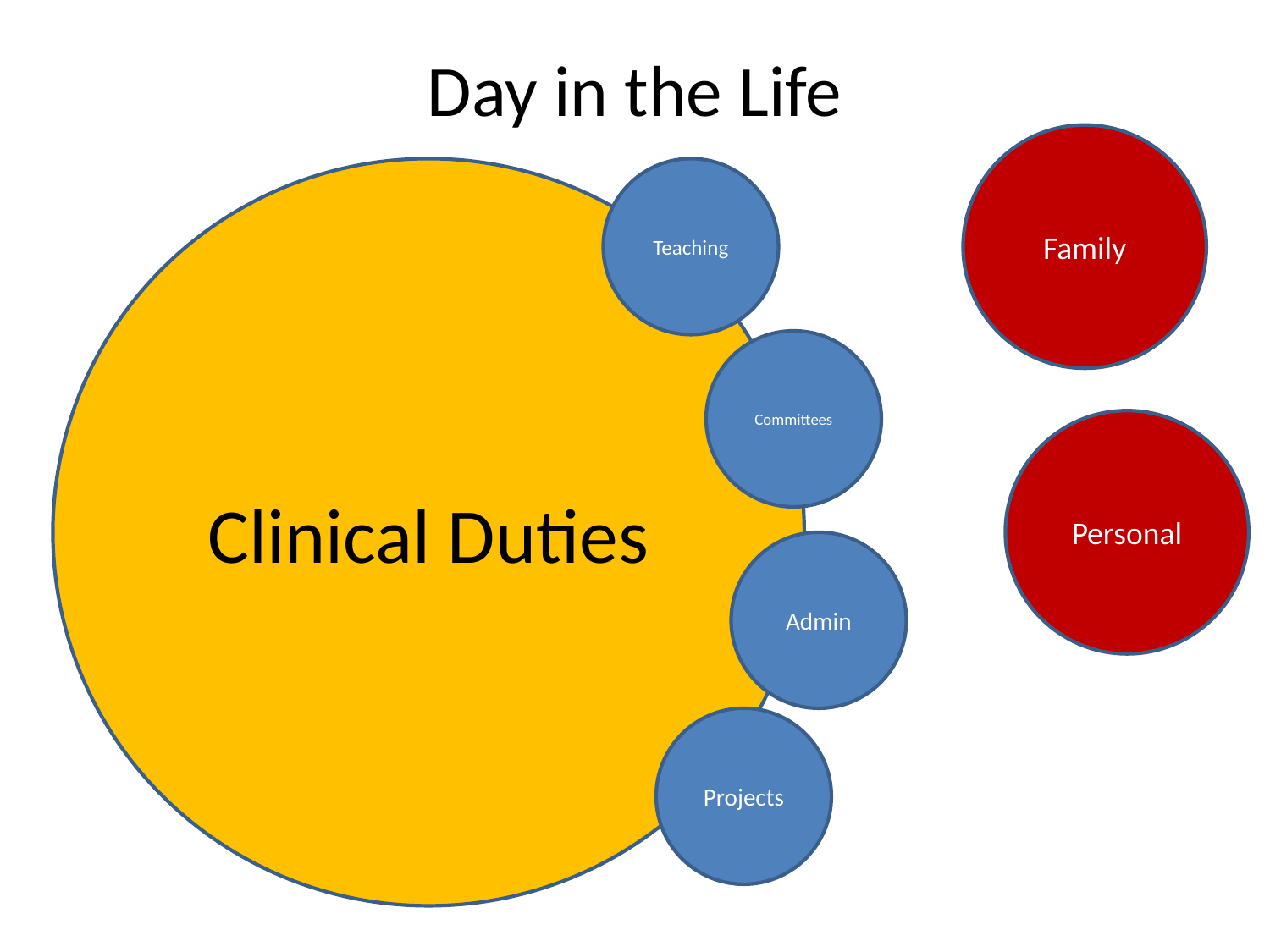

# Day in the Life
Family
Clinical Duties
Teaching
Committees
Personal
Admin
Projects

## Slide 7
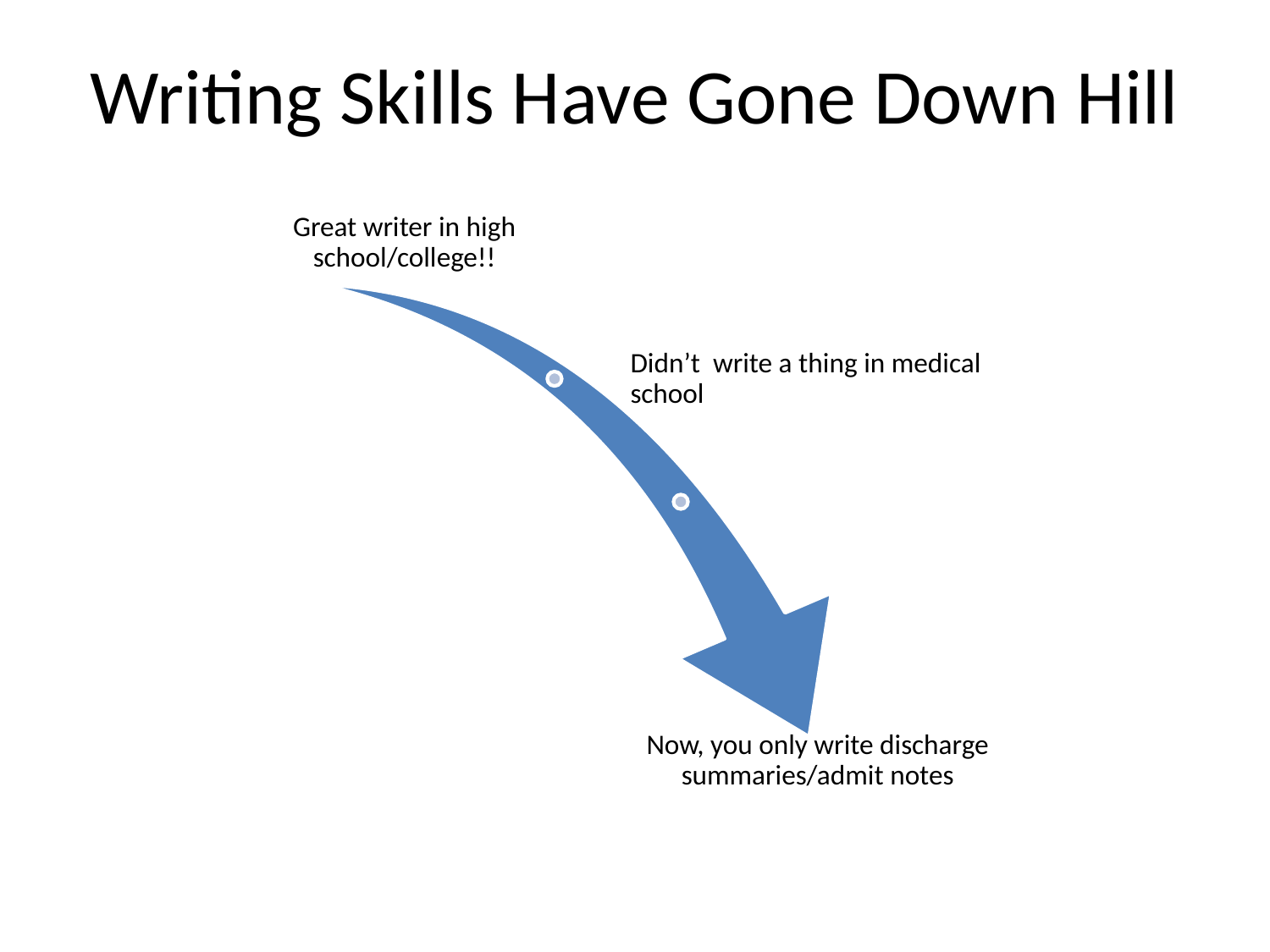

# Writing Skills Have Gone Down Hill

## Slide 8
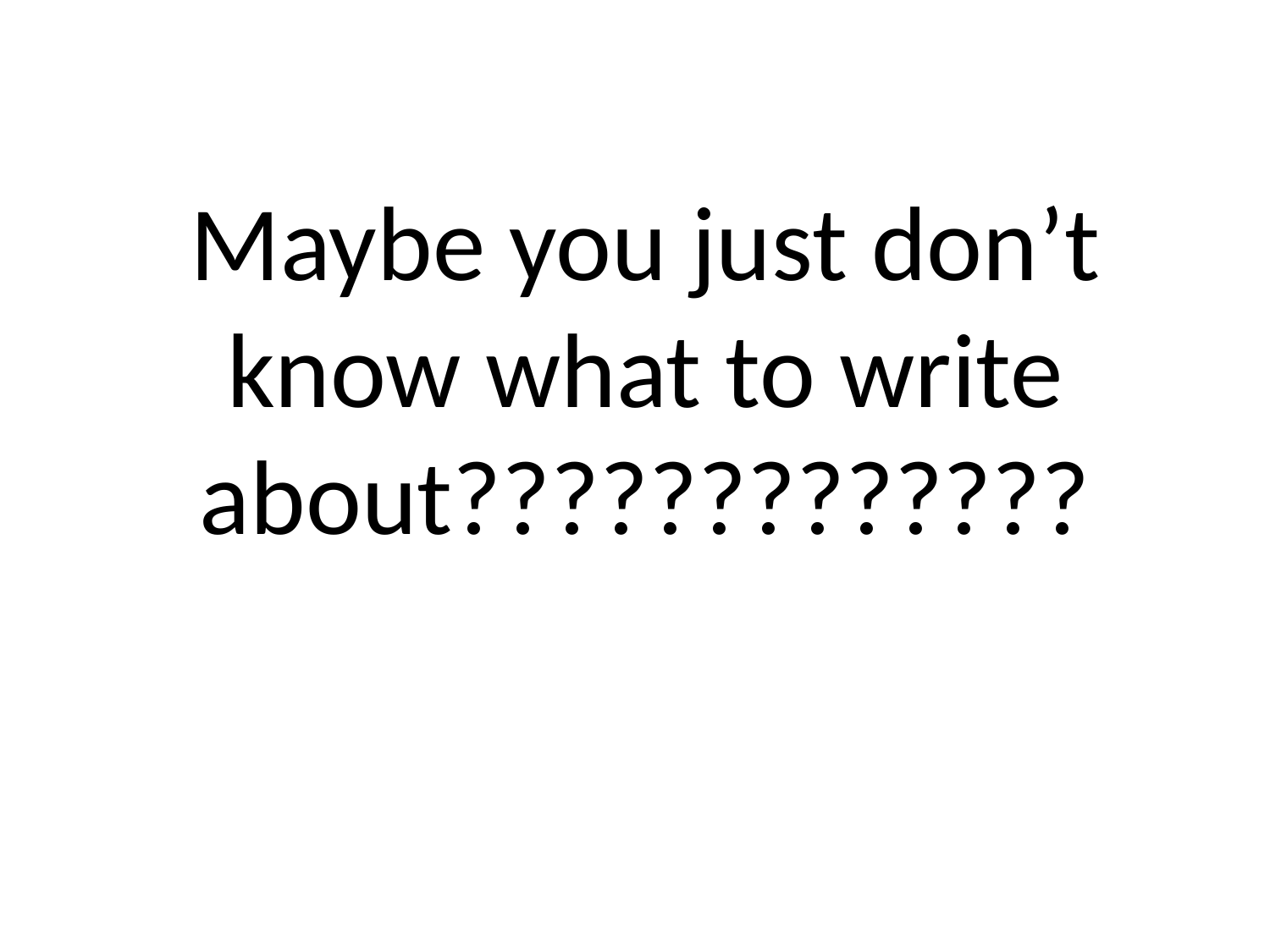

# Maybe you just don’t know what to write about?????????????

## Slide 9
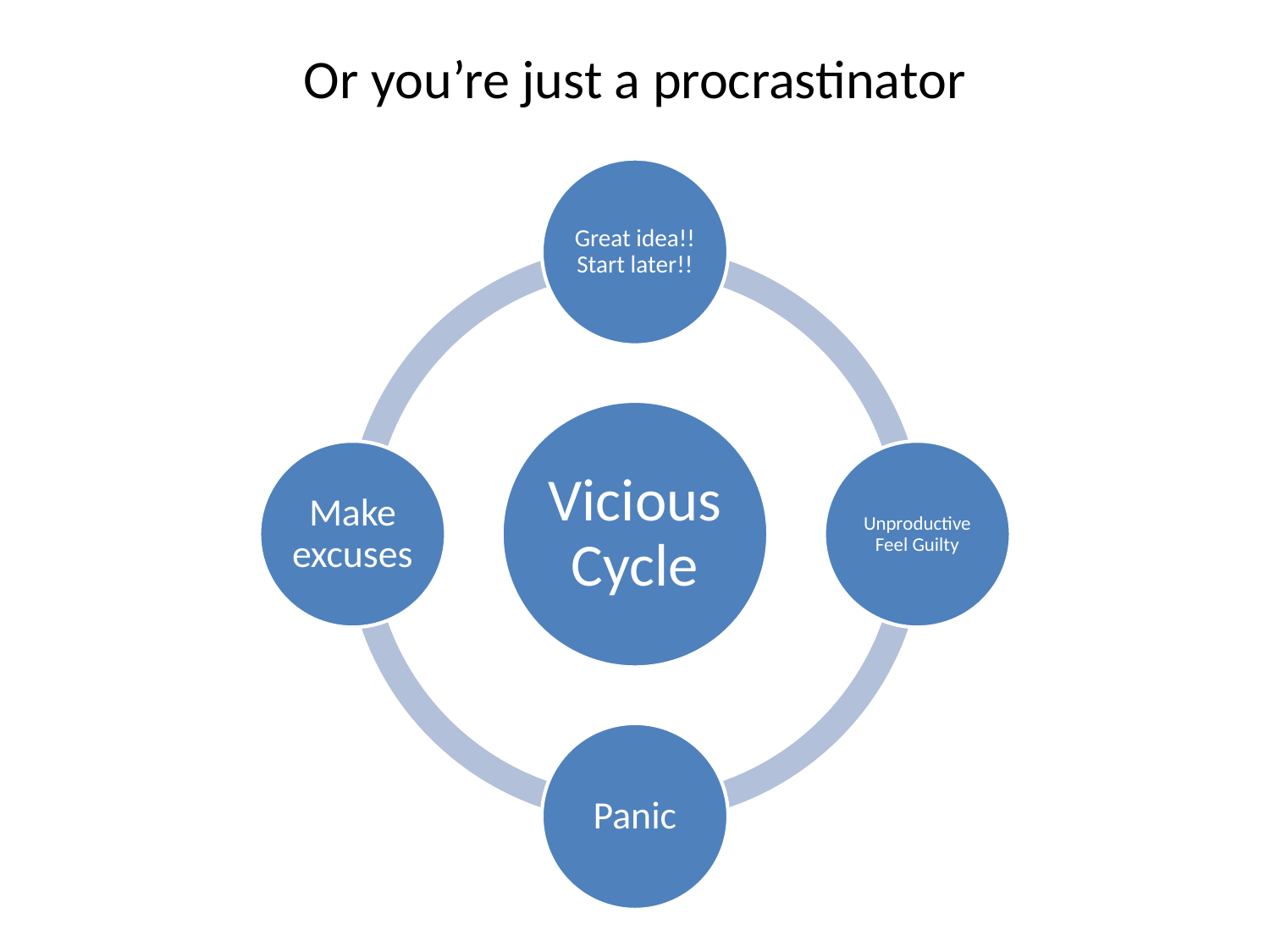

# Or you’re just a procrastinator

## Slide 10
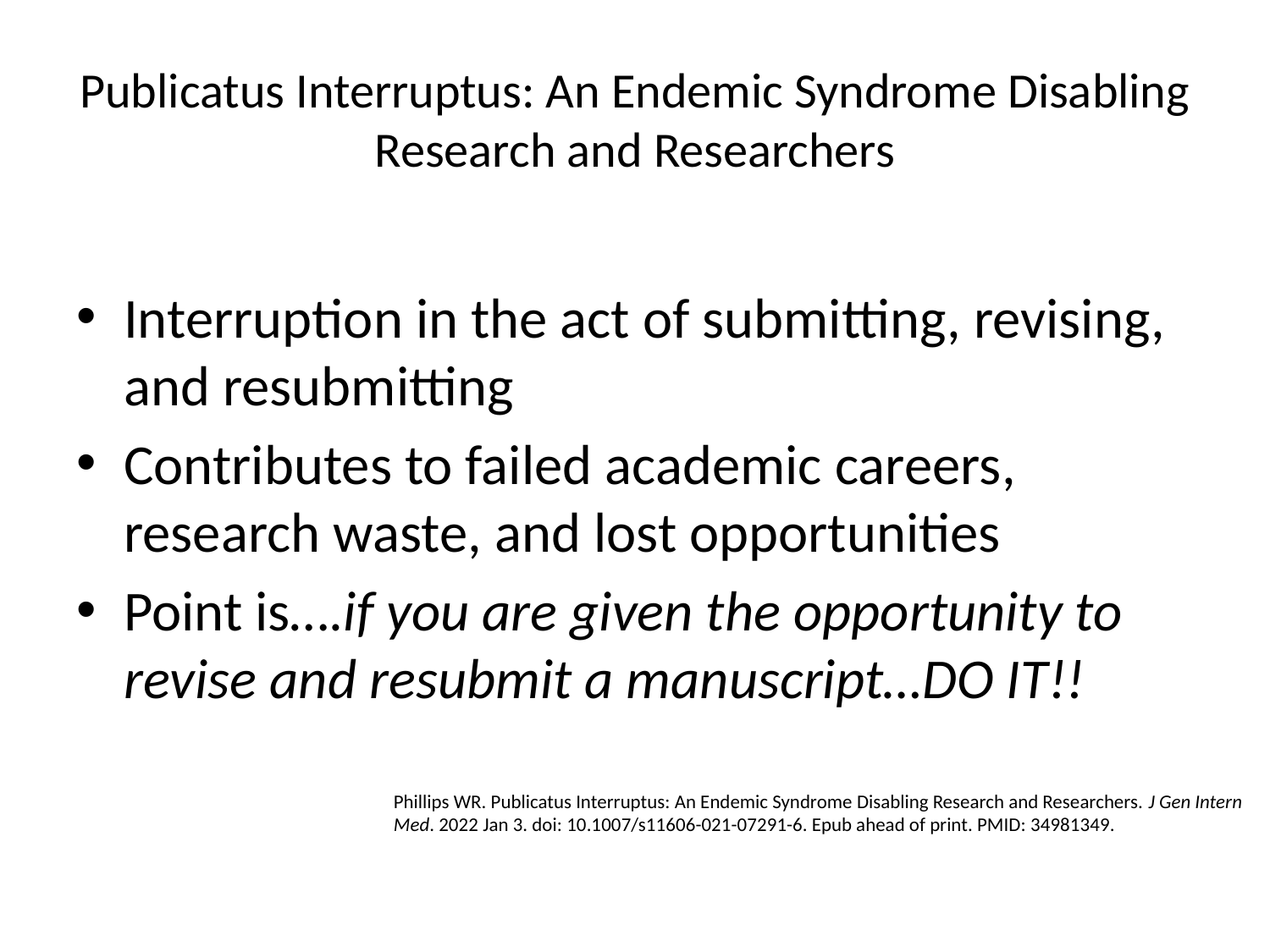

# Publicatus Interruptus: An Endemic Syndrome Disabling Research and Researchers
Interruption in the act of submitting, revising, and resubmitting
Contributes to failed academic careers, research waste, and lost opportunities
Point is….if you are given the opportunity to revise and resubmit a manuscript…DO IT!!
Phillips WR. Publicatus Interruptus: An Endemic Syndrome Disabling Research and Researchers. J Gen Intern Med. 2022 Jan 3. doi: 10.1007/s11606-021-07291-6. Epub ahead of print. PMID: 34981349.

## Slide 11
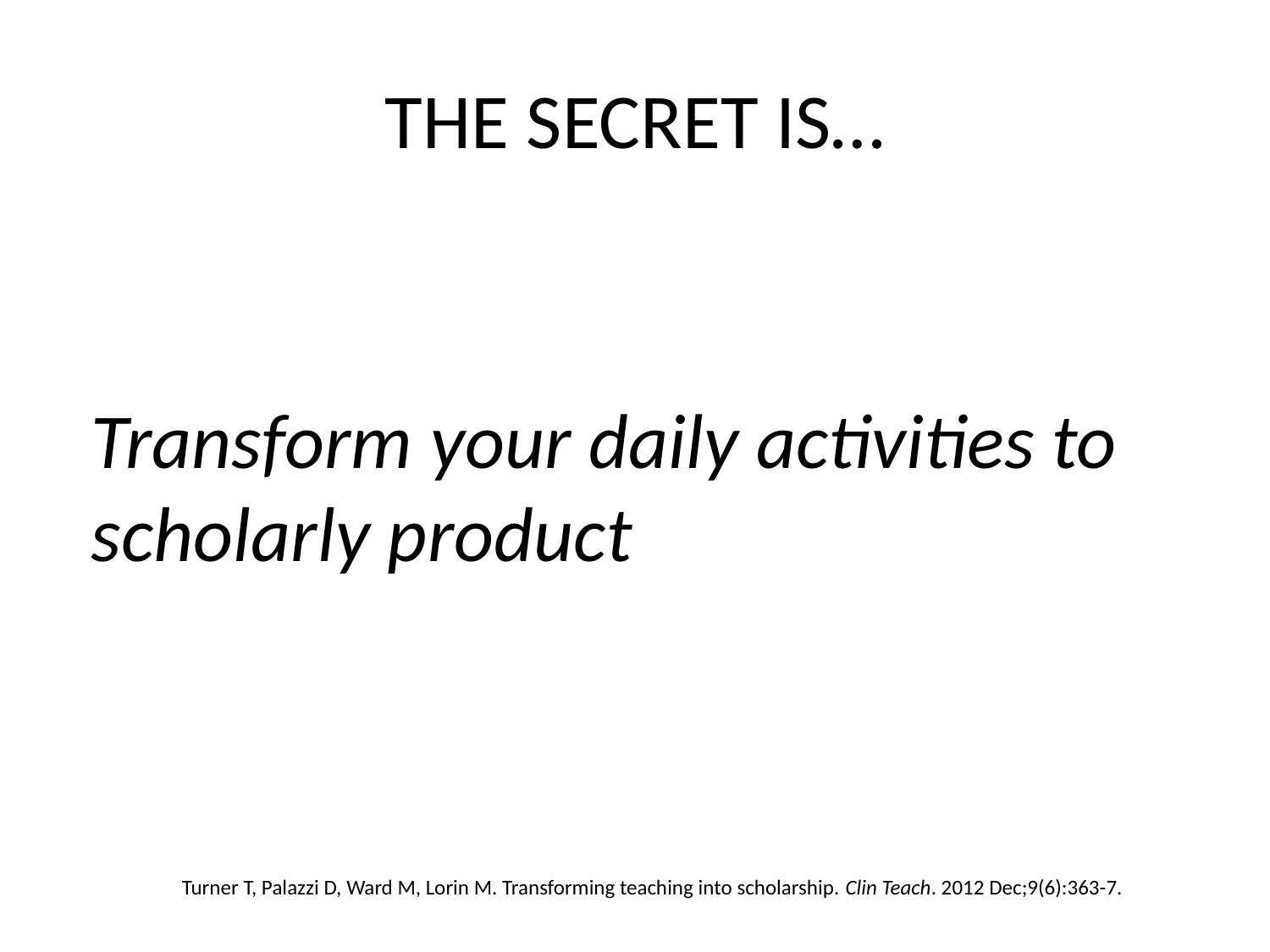

# THE SECRET IS…
Transform your daily activities to scholarly product
Turner T, Palazzi D, Ward M, Lorin M. Transforming teaching into scholarship. Clin Teach. 2012 Dec;9(6):363-7.

## Slide 12
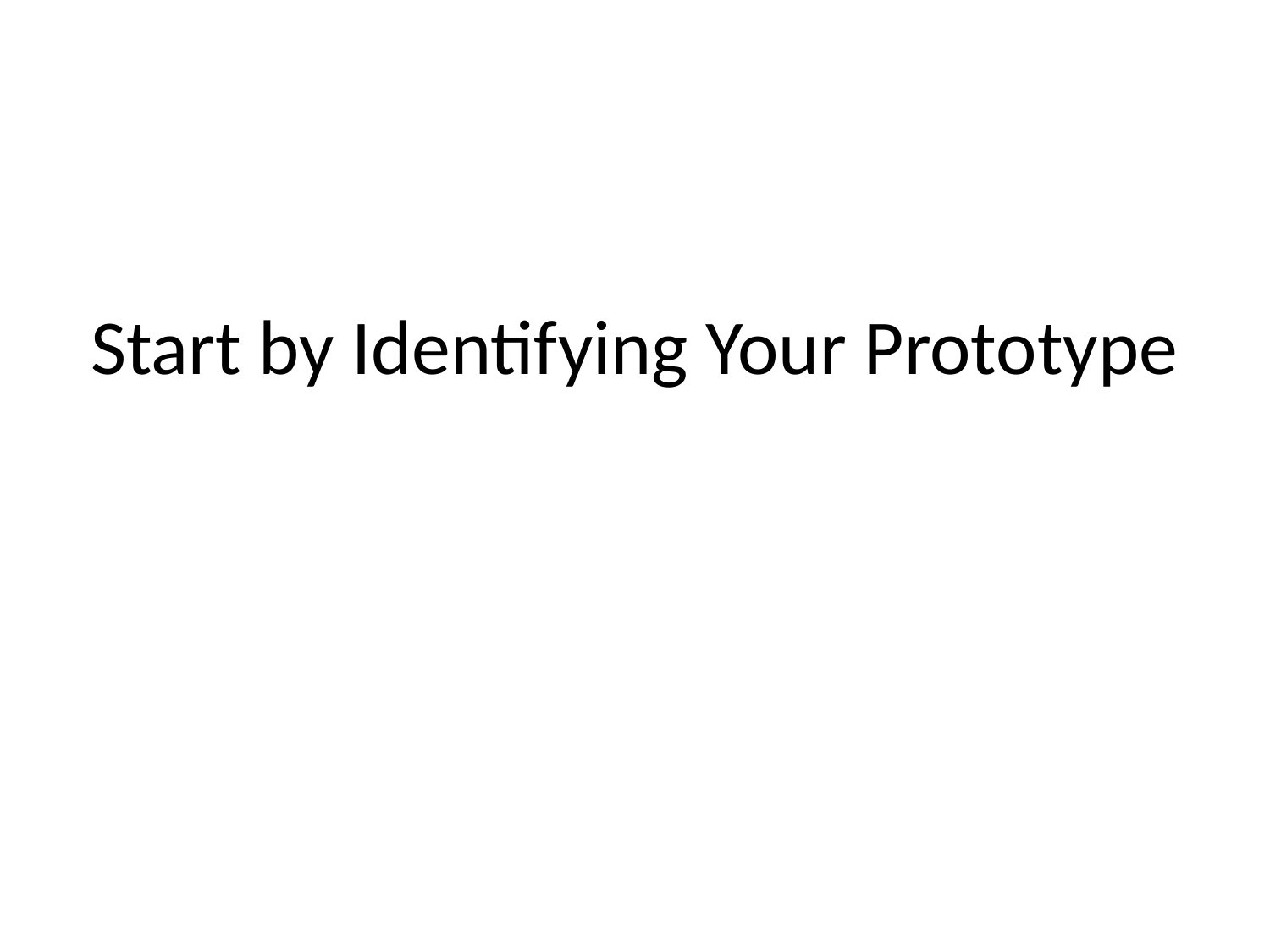

# Start by Identifying Your Prototype

## Slide 13
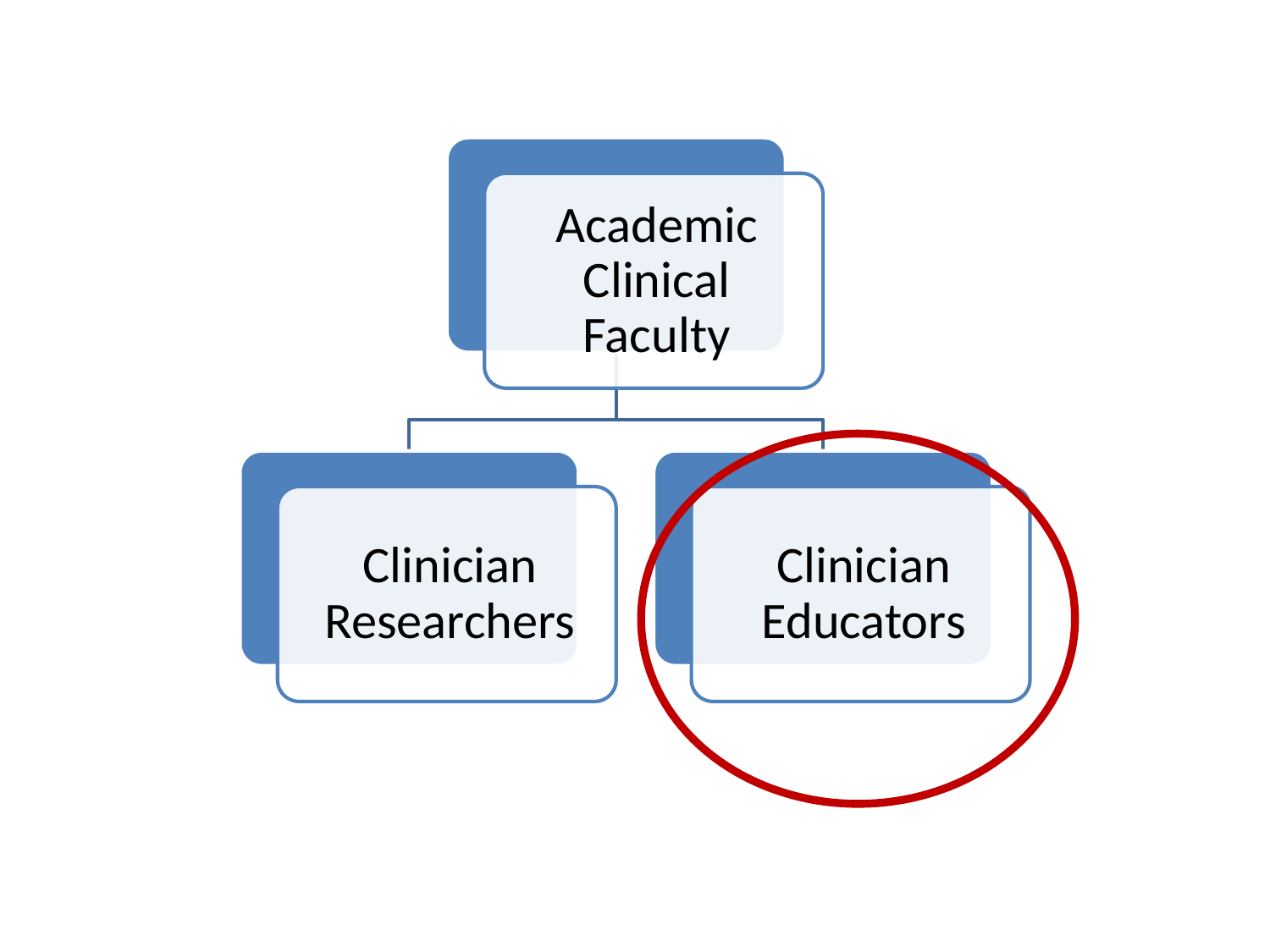

## Slide 14
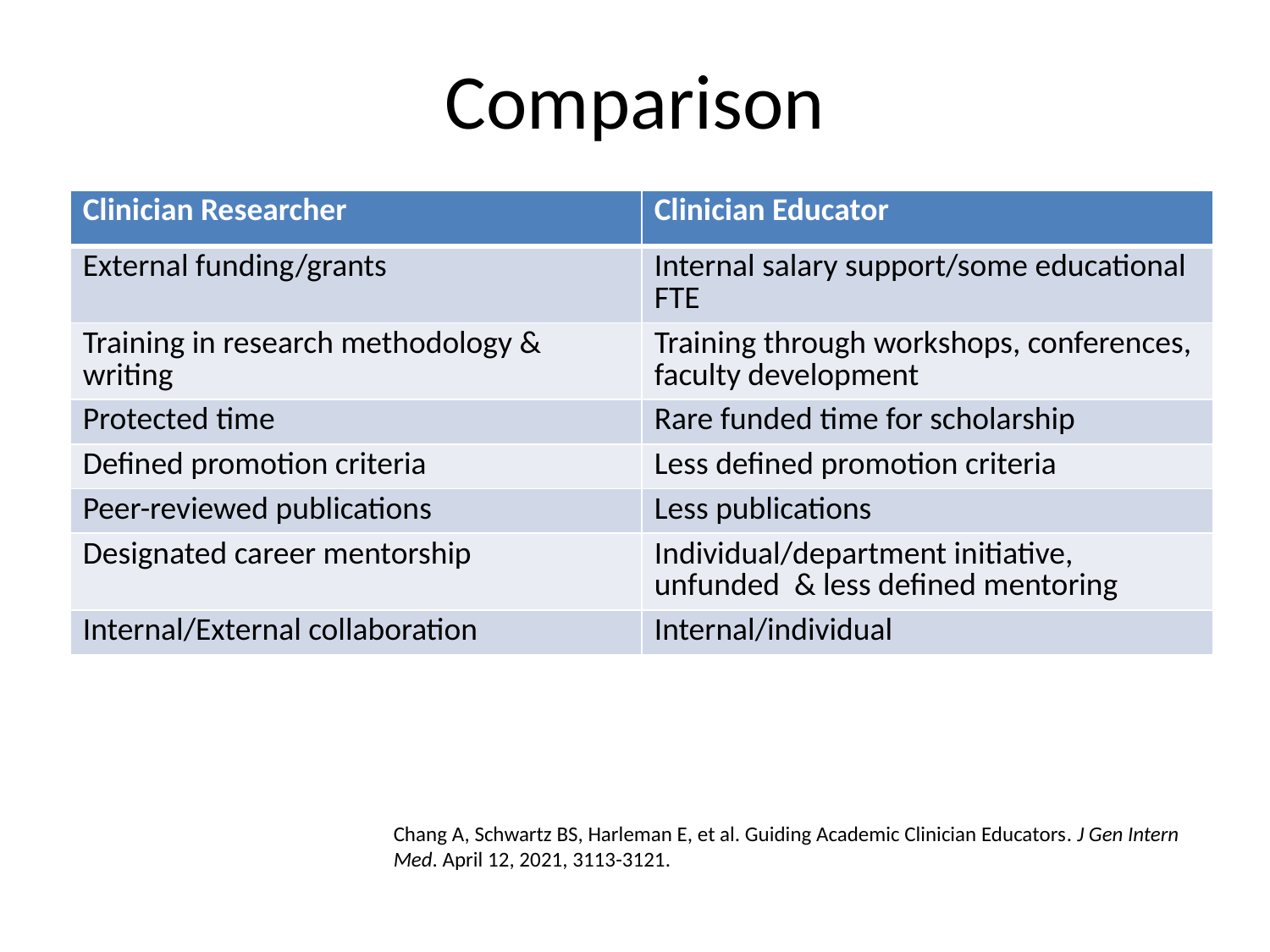

# Comparison
| Clinician Researcher | Clinician Educator |
| --- | --- |
| External funding/grants | Internal salary support/some educational FTE |
| Training in research methodology & writing | Training through workshops, conferences, faculty development |
| Protected time | Rare funded time for scholarship |
| Defined promotion criteria | Less defined promotion criteria |
| Peer-reviewed publications | Less publications |
| Designated career mentorship | Individual/department initiative, unfunded & less defined mentoring |
| Internal/External collaboration | Internal/individual |
Chang A, Schwartz BS, Harleman E, et al. Guiding Academic Clinician Educators. J Gen Intern Med. April 12, 2021, 3113-3121.

## Slide 15
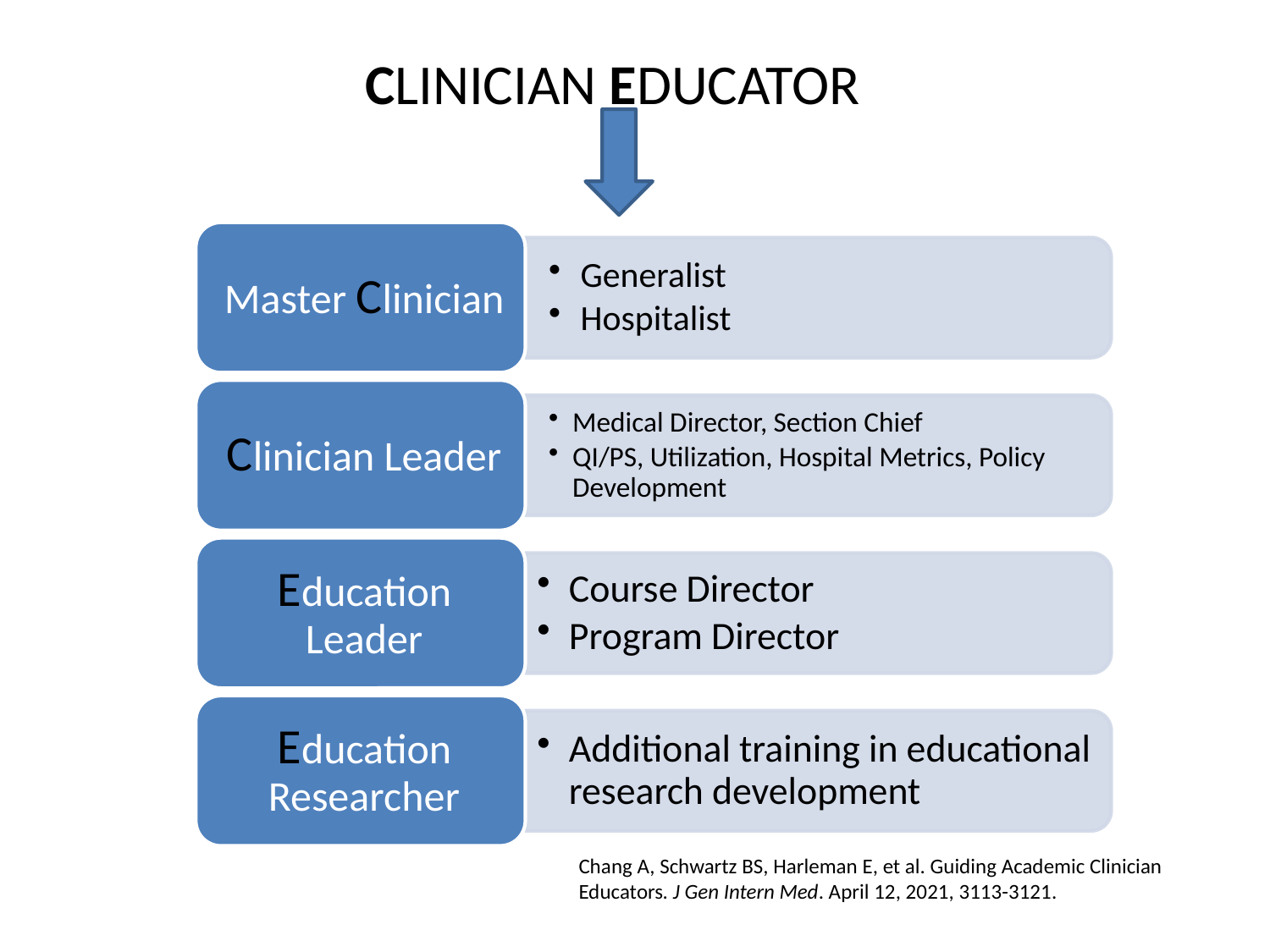

CLINICIAN EDUCATOR
Chang A, Schwartz BS, Harleman E, et al. Guiding Academic Clinician Educators. J Gen Intern Med. April 12, 2021, 3113-3121.

## Slide 16
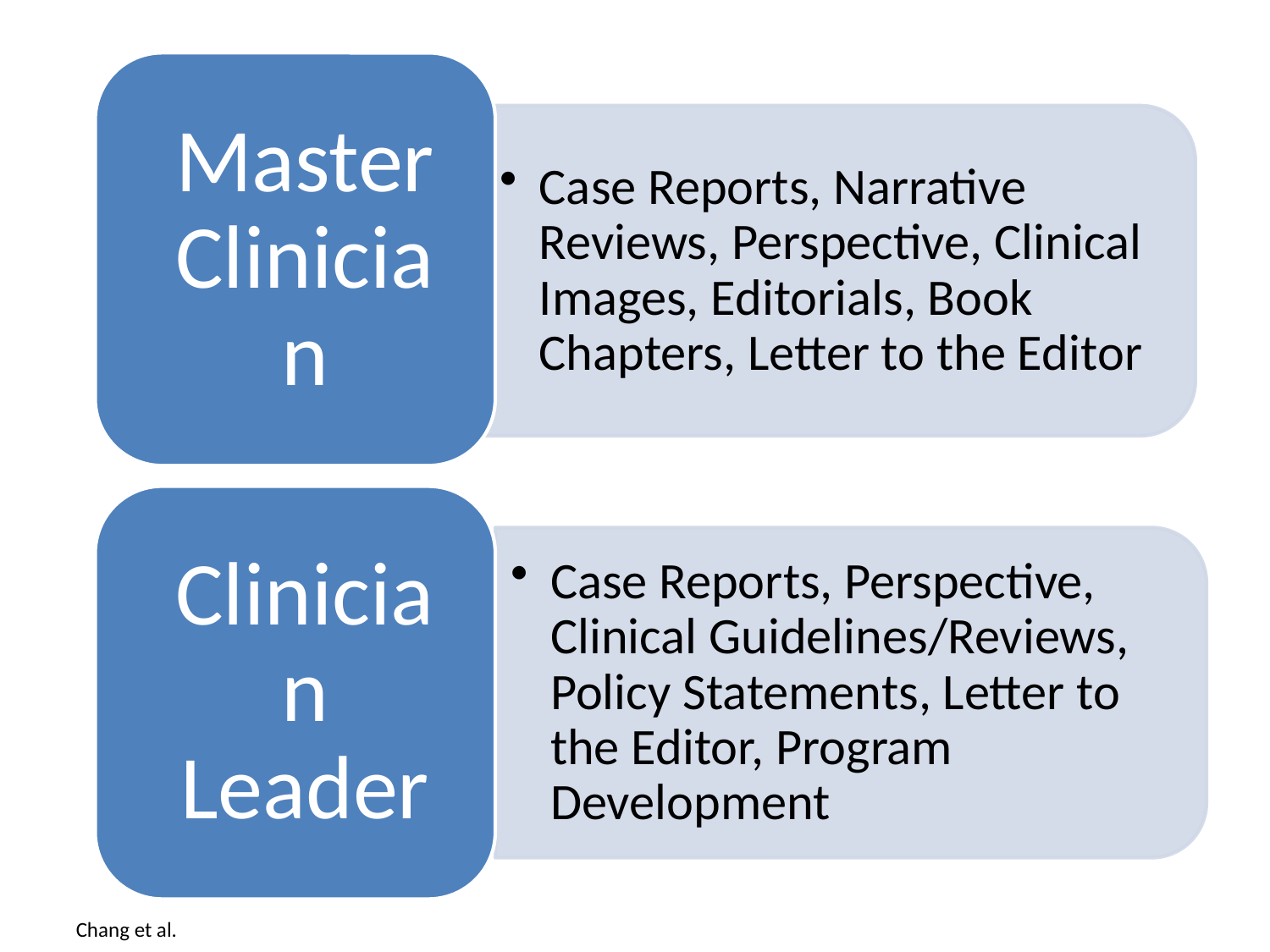

Chang et al.

## Slide 17
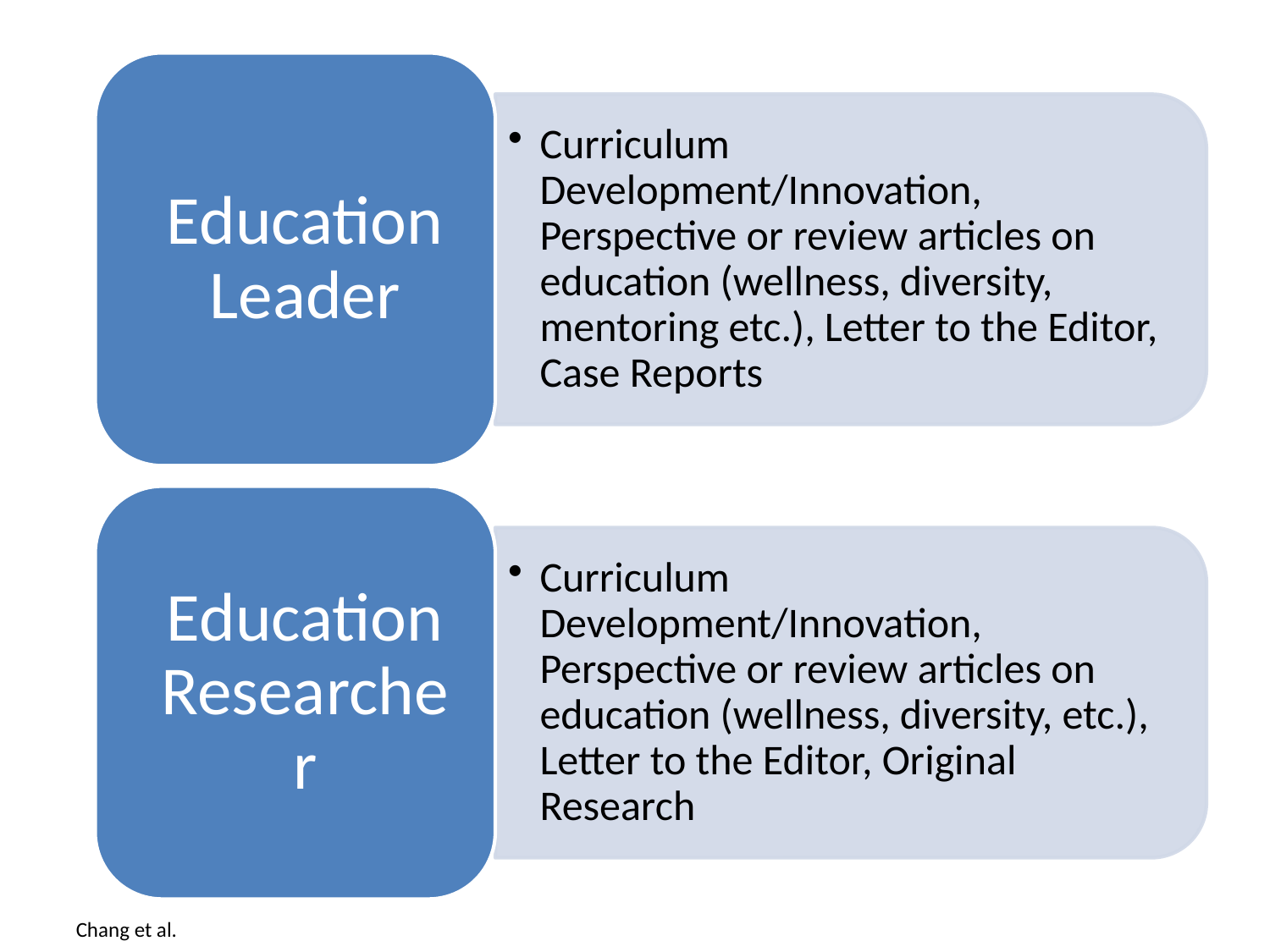

Chang et al.

## Slide 18
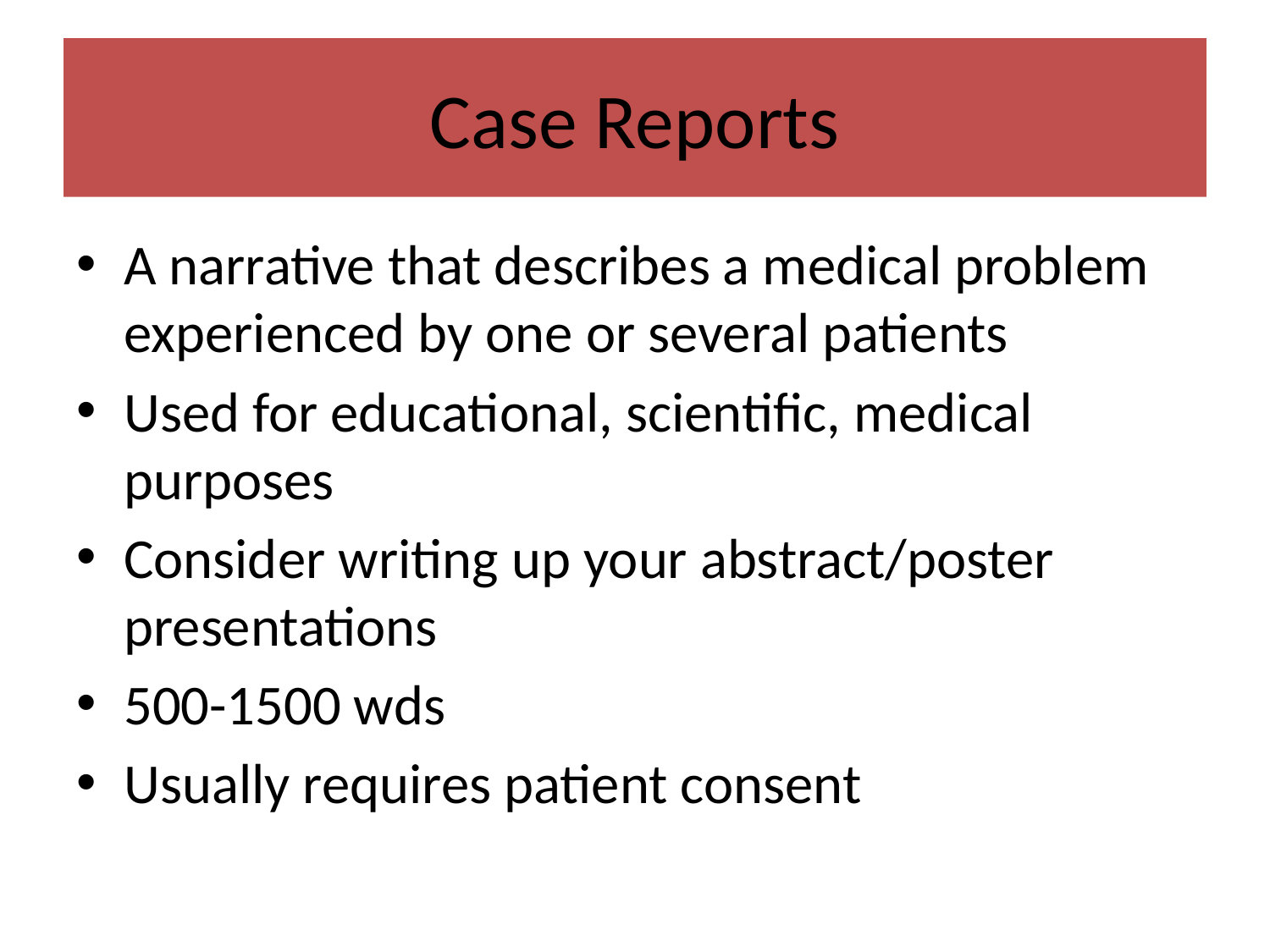

# Case Reports
A narrative that describes a medical problem experienced by one or several patients
Used for educational, scientific, medical purposes
Consider writing up your abstract/poster presentations
500-1500 wds
Usually requires patient consent

## Slide 19
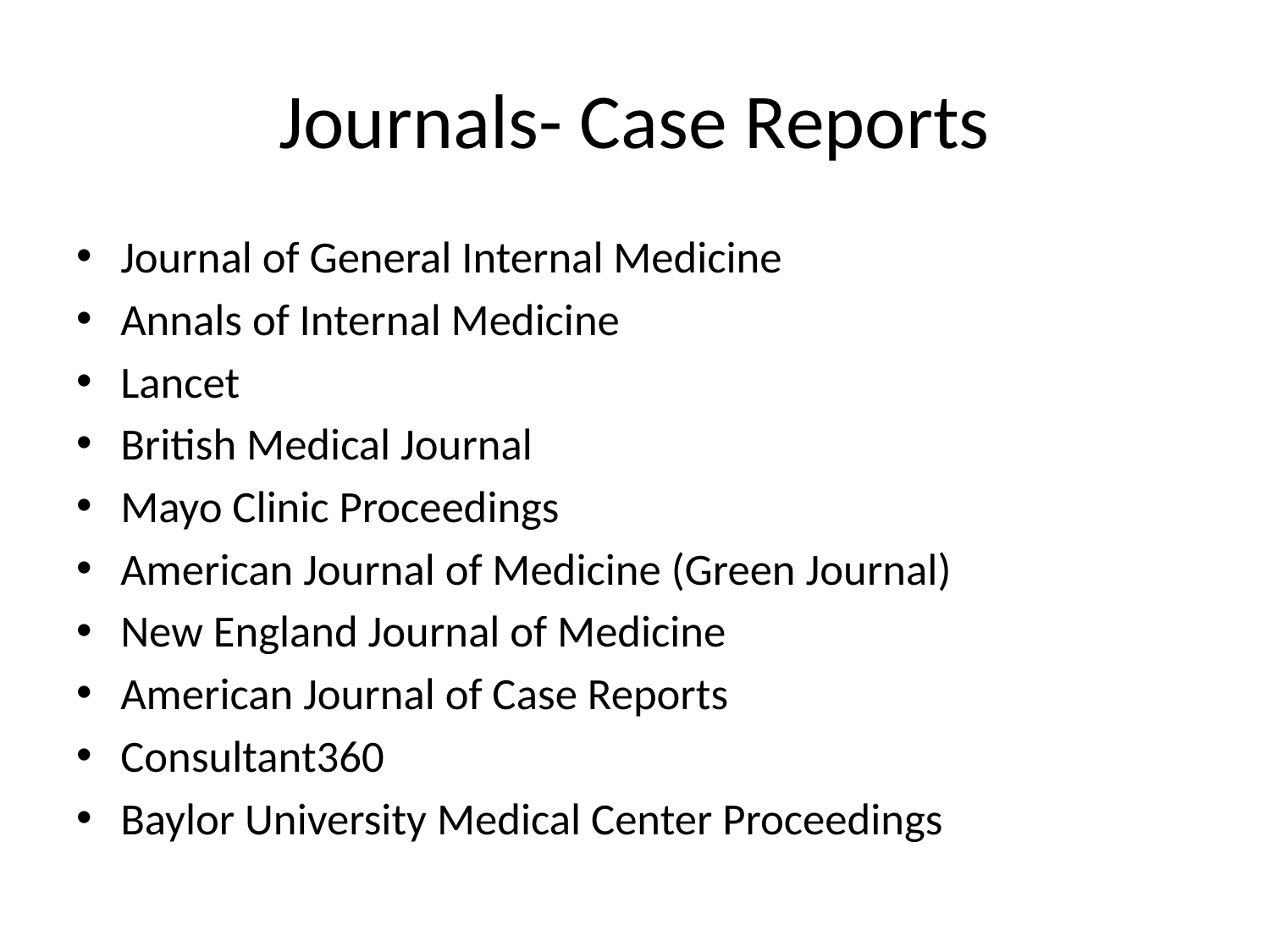

# Journals- Case Reports
Journal of General Internal Medicine
Annals of Internal Medicine
Lancet
British Medical Journal
Mayo Clinic Proceedings
American Journal of Medicine (Green Journal)
New England Journal of Medicine
American Journal of Case Reports
Consultant360
Baylor University Medical Center Proceedings

## Slide 20
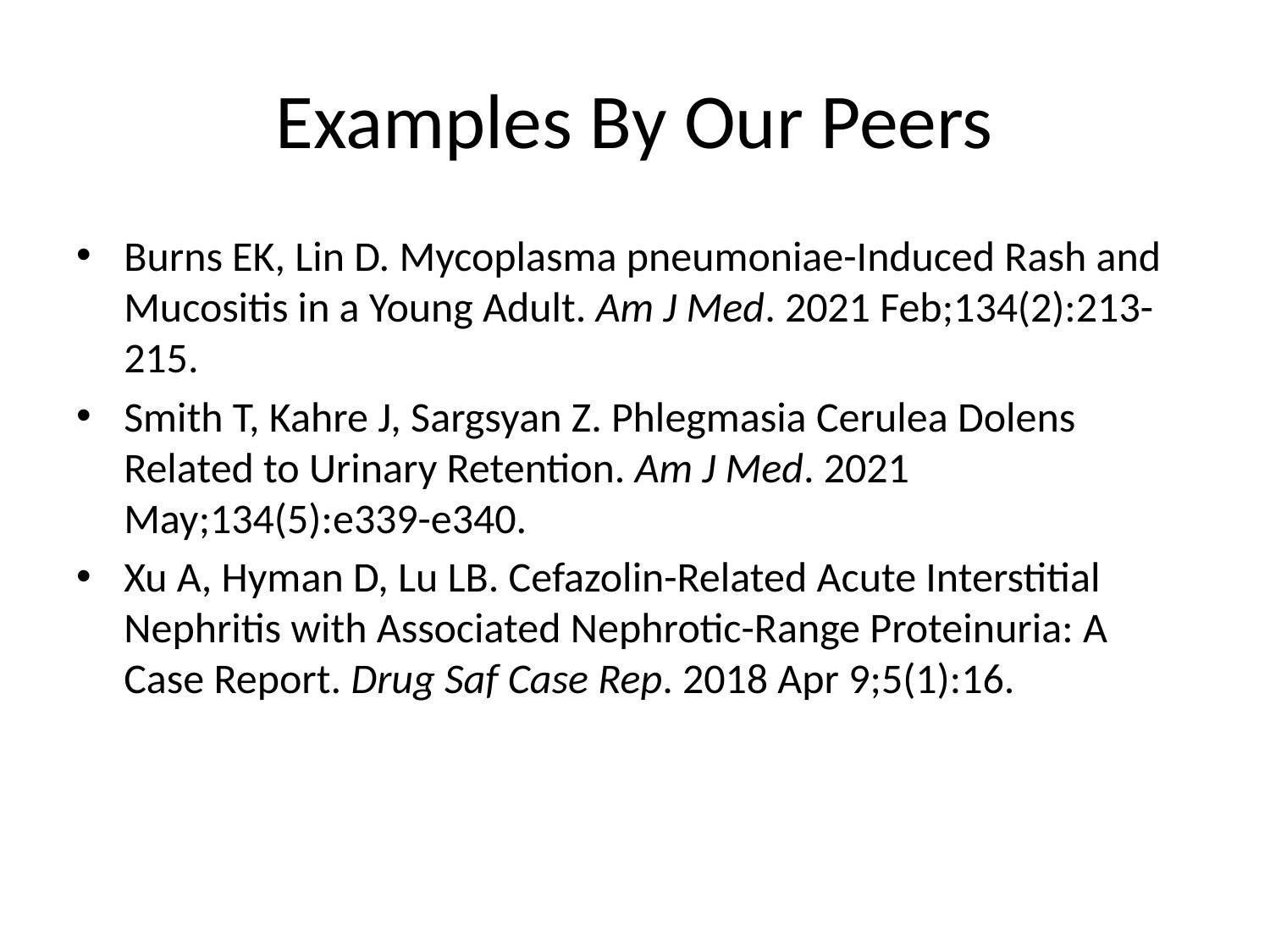

# Examples By Our Peers
Burns EK, Lin D. Mycoplasma pneumoniae-Induced Rash and Mucositis in a Young Adult. Am J Med. 2021 Feb;134(2):213-215.
Smith T, Kahre J, Sargsyan Z. Phlegmasia Cerulea Dolens Related to Urinary Retention. Am J Med. 2021 May;134(5):e339-e340.
Xu A, Hyman D, Lu LB. Cefazolin-Related Acute Interstitial Nephritis with Associated Nephrotic-Range Proteinuria: A Case Report. Drug Saf Case Rep. 2018 Apr 9;5(1):16.

## Slide 21
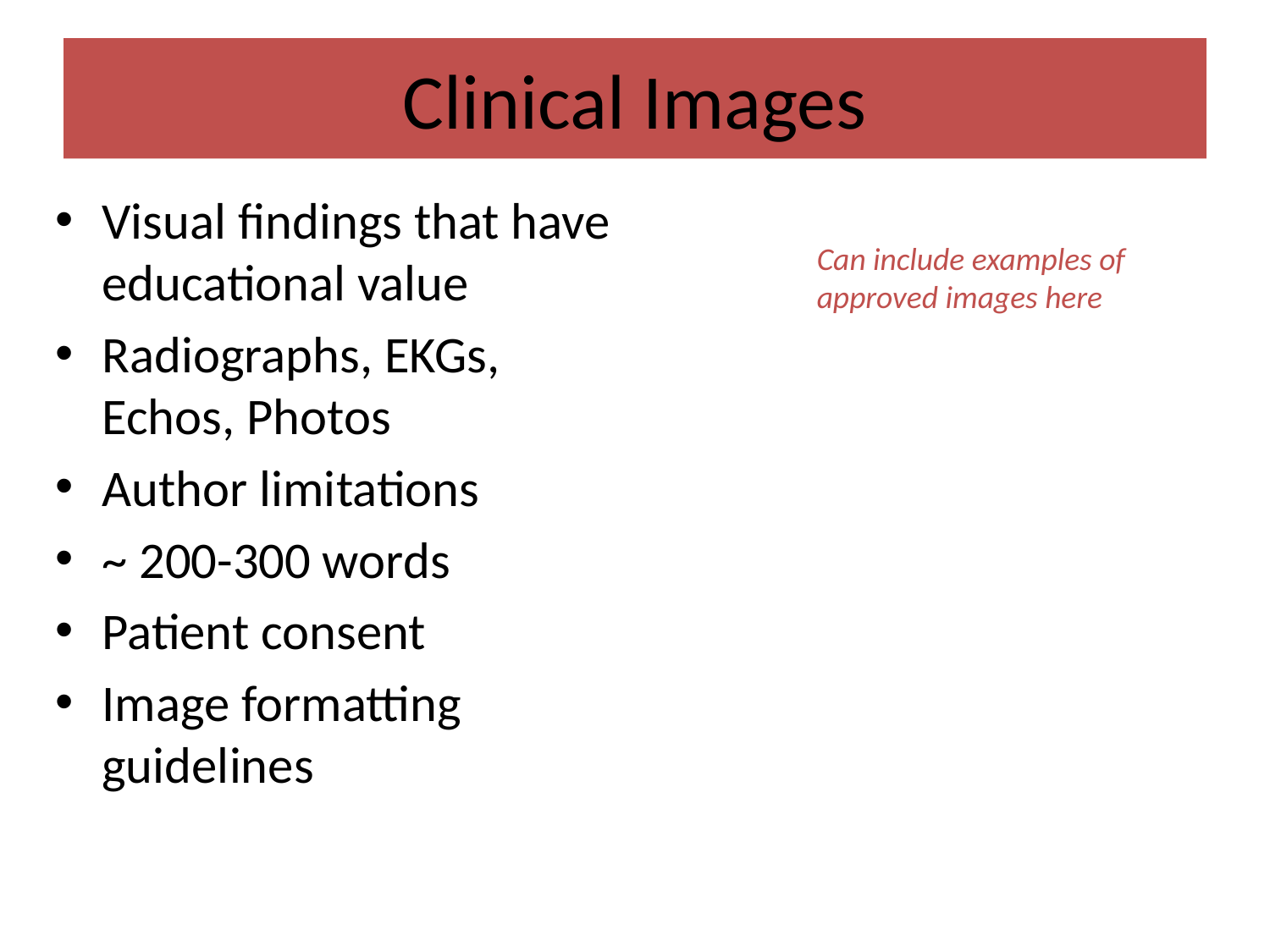

# Clinical Images
Visual findings that have educational value
Radiographs, EKGs, Echos, Photos
Author limitations
~ 200-300 words
Patient consent
Image formatting guidelines
Can include examples of approved images here

## Slide 22
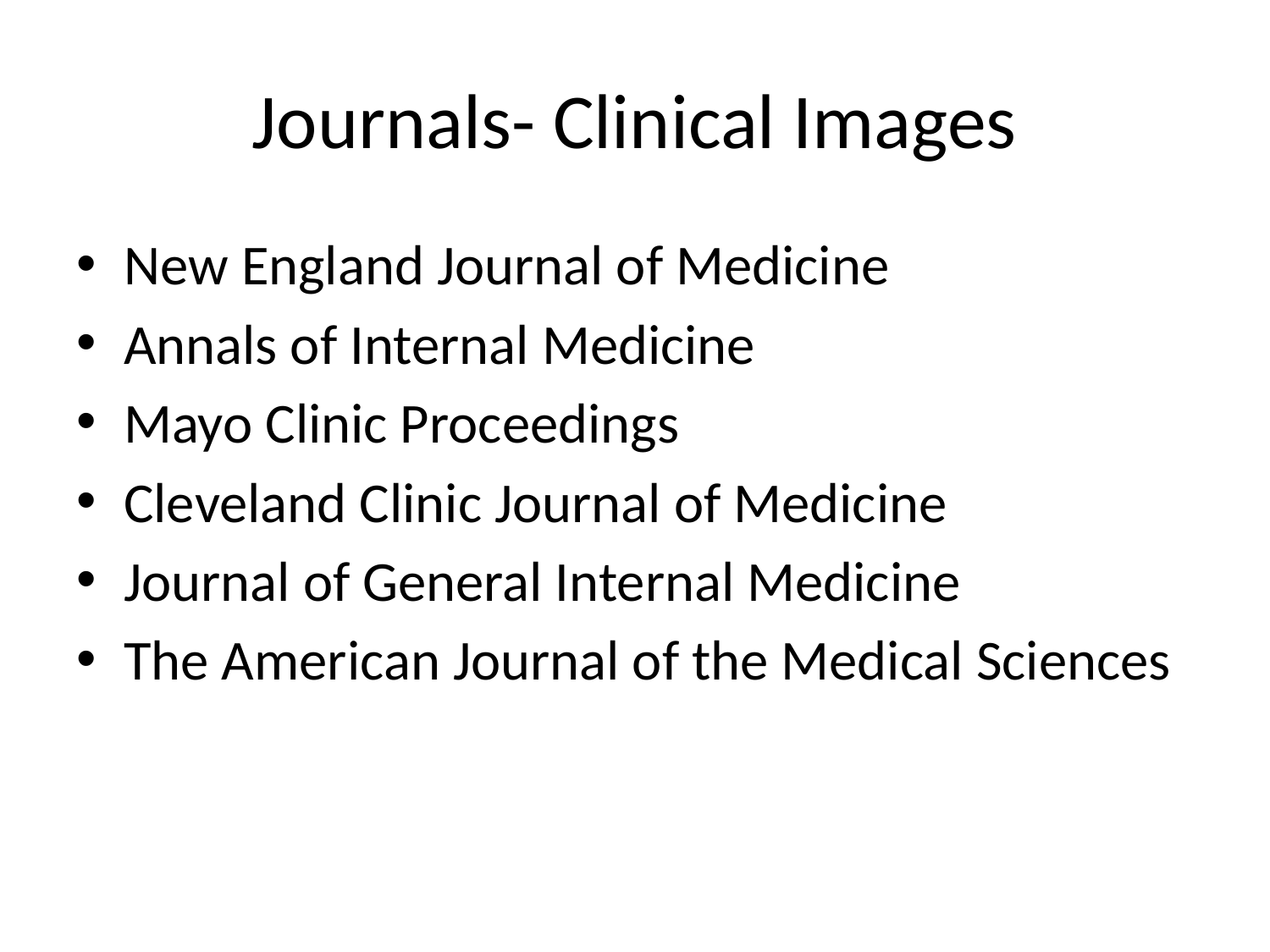

# Journals- Clinical Images
New England Journal of Medicine
Annals of Internal Medicine
Mayo Clinic Proceedings
Cleveland Clinic Journal of Medicine
Journal of General Internal Medicine
The American Journal of the Medical Sciences

## Slide 23
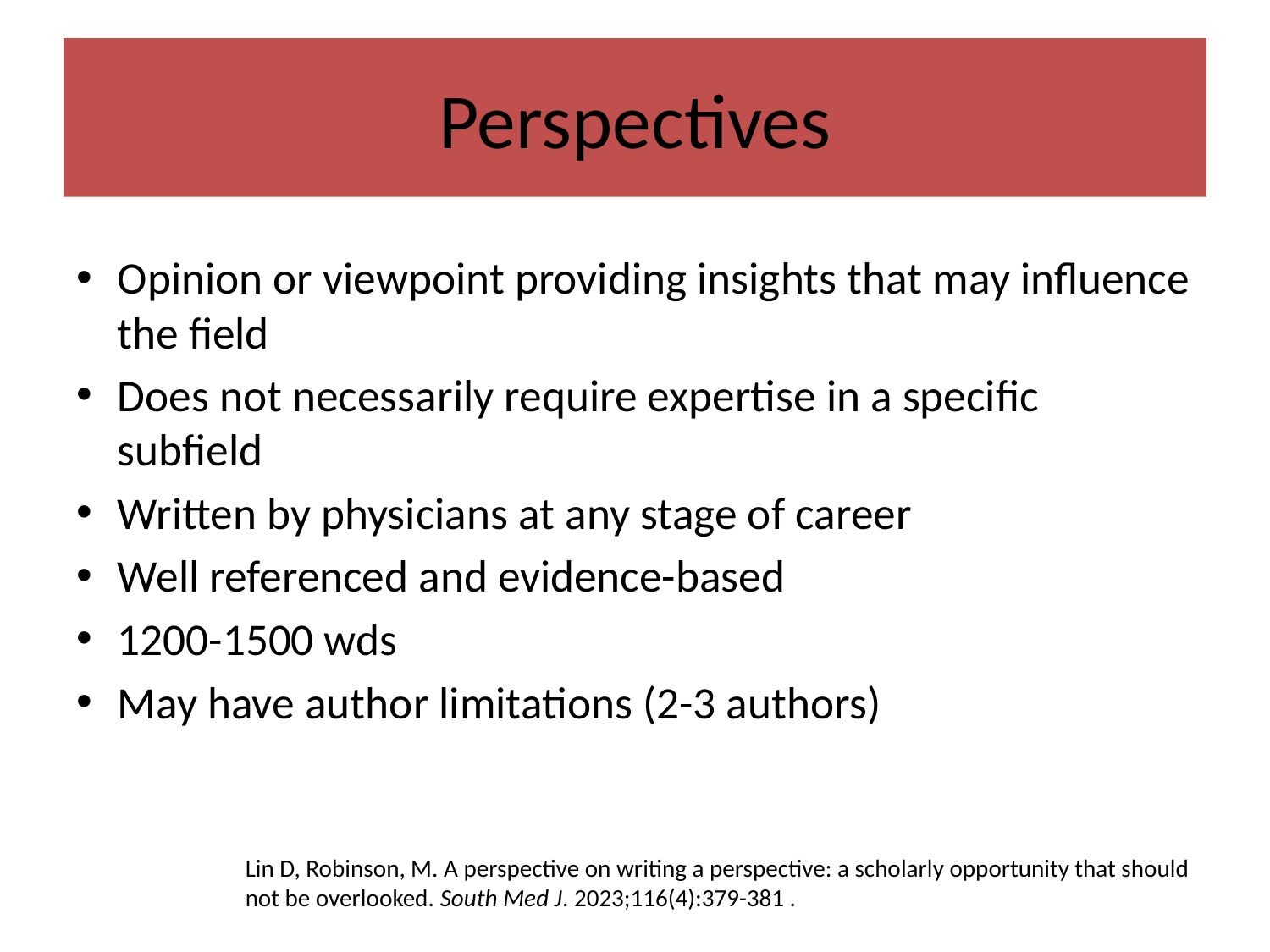

# Perspectives
Opinion or viewpoint providing insights that may influence the field
Does not necessarily require expertise in a specific subfield
Written by physicians at any stage of career
Well referenced and evidence-based
1200-1500 wds
May have author limitations (2-3 authors)
Lin D, Robinson, M. A perspective on writing a perspective: a scholarly opportunity that should not be overlooked. South Med J. 2023;116(4):379-381 .

## Slide 24
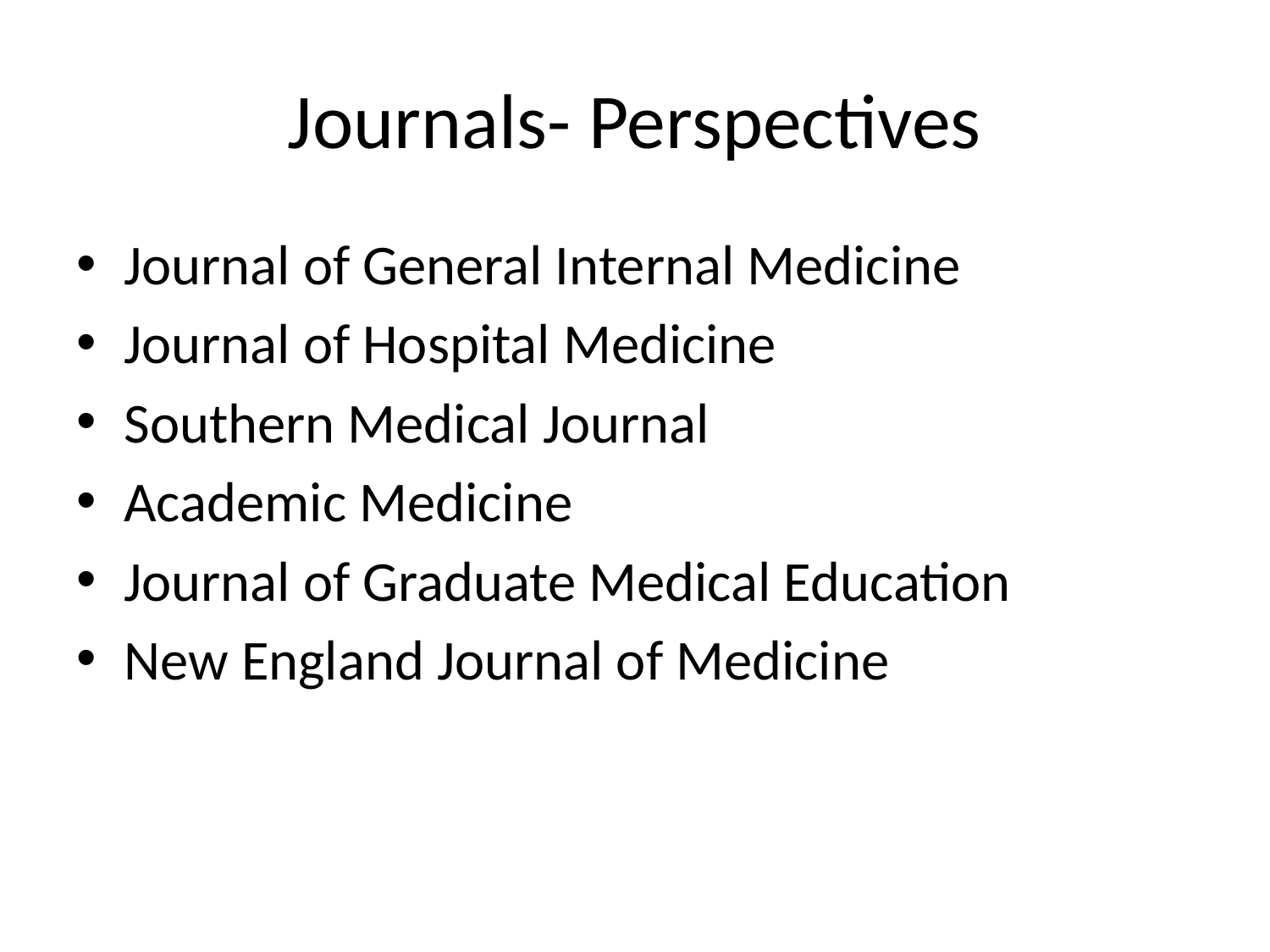

# Journals- Perspectives
Journal of General Internal Medicine
Journal of Hospital Medicine
Southern Medical Journal
Academic Medicine
Journal of Graduate Medical Education
New England Journal of Medicine

## Slide 25
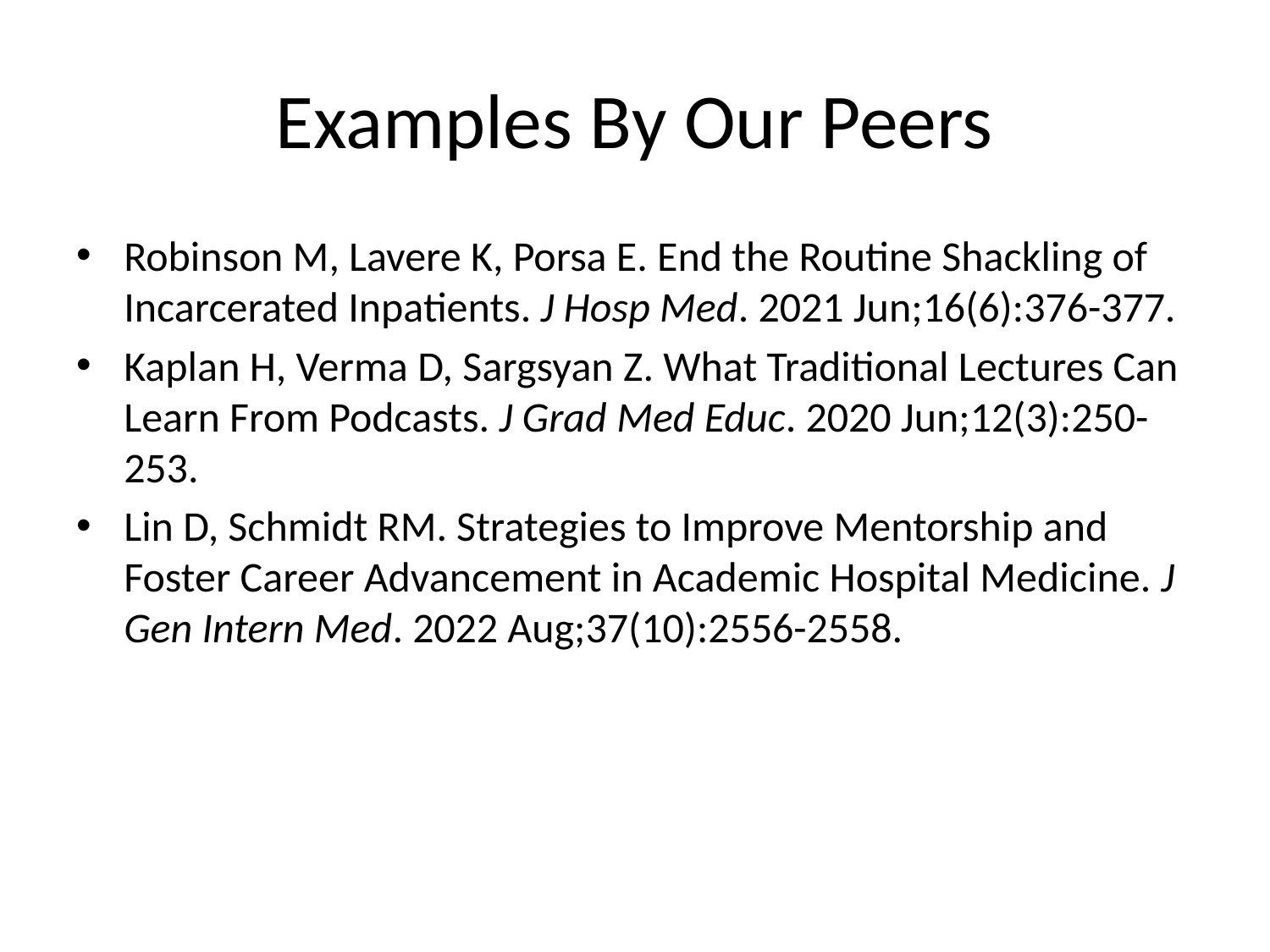

# Examples By Our Peers
Robinson M, Lavere K, Porsa E. End the Routine Shackling of Incarcerated Inpatients. J Hosp Med. 2021 Jun;16(6):376-377.
Kaplan H, Verma D, Sargsyan Z. What Traditional Lectures Can Learn From Podcasts. J Grad Med Educ. 2020 Jun;12(3):250-253.
Lin D, Schmidt RM. Strategies to Improve Mentorship and Foster Career Advancement in Academic Hospital Medicine. J Gen Intern Med. 2022 Aug;37(10):2556-2558.

## Slide 26
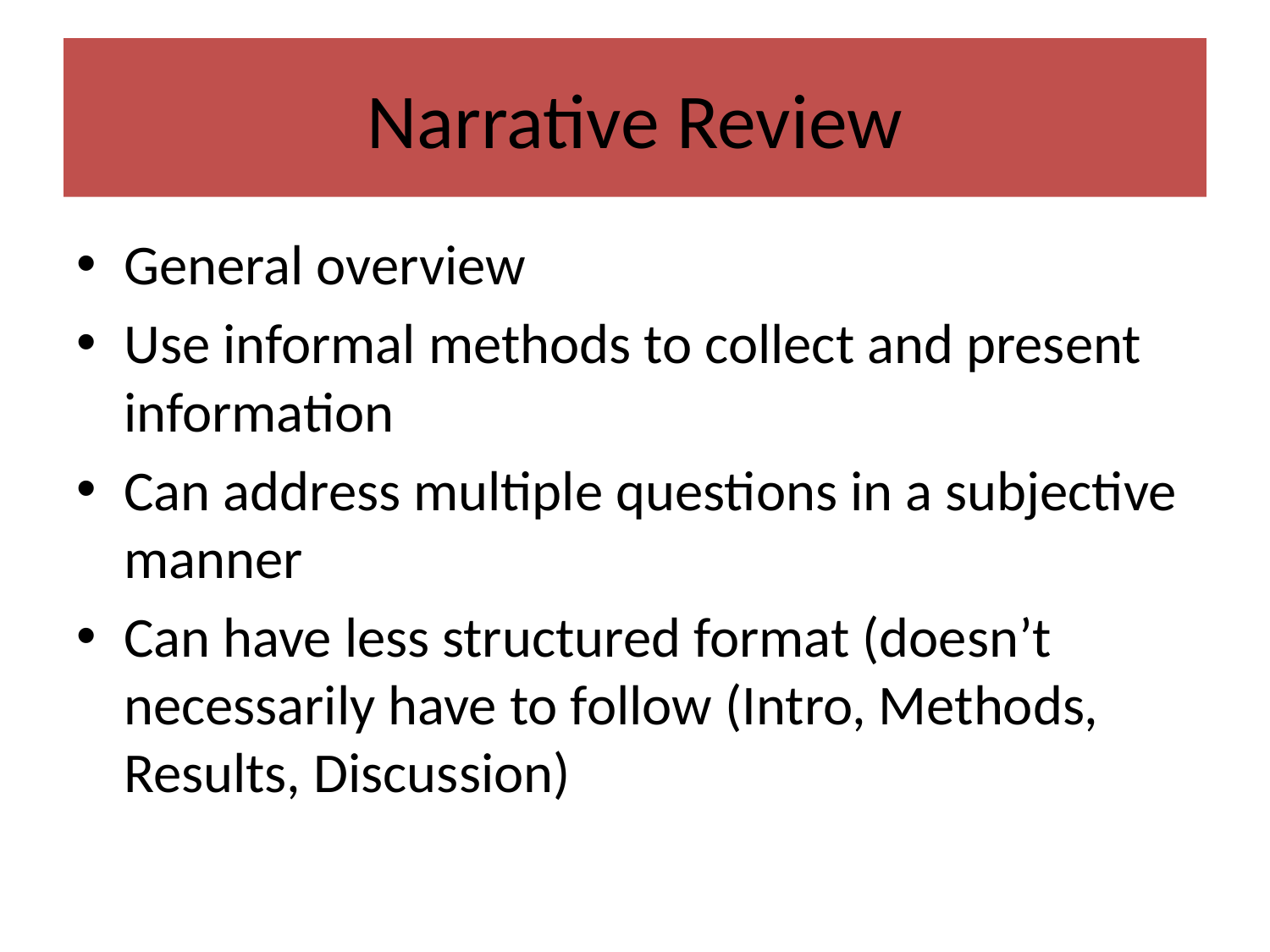

# Narrative Review
General overview
Use informal methods to collect and present information
Can address multiple questions in a subjective manner
Can have less structured format (doesn’t necessarily have to follow (Intro, Methods, Results, Discussion)

## Slide 27
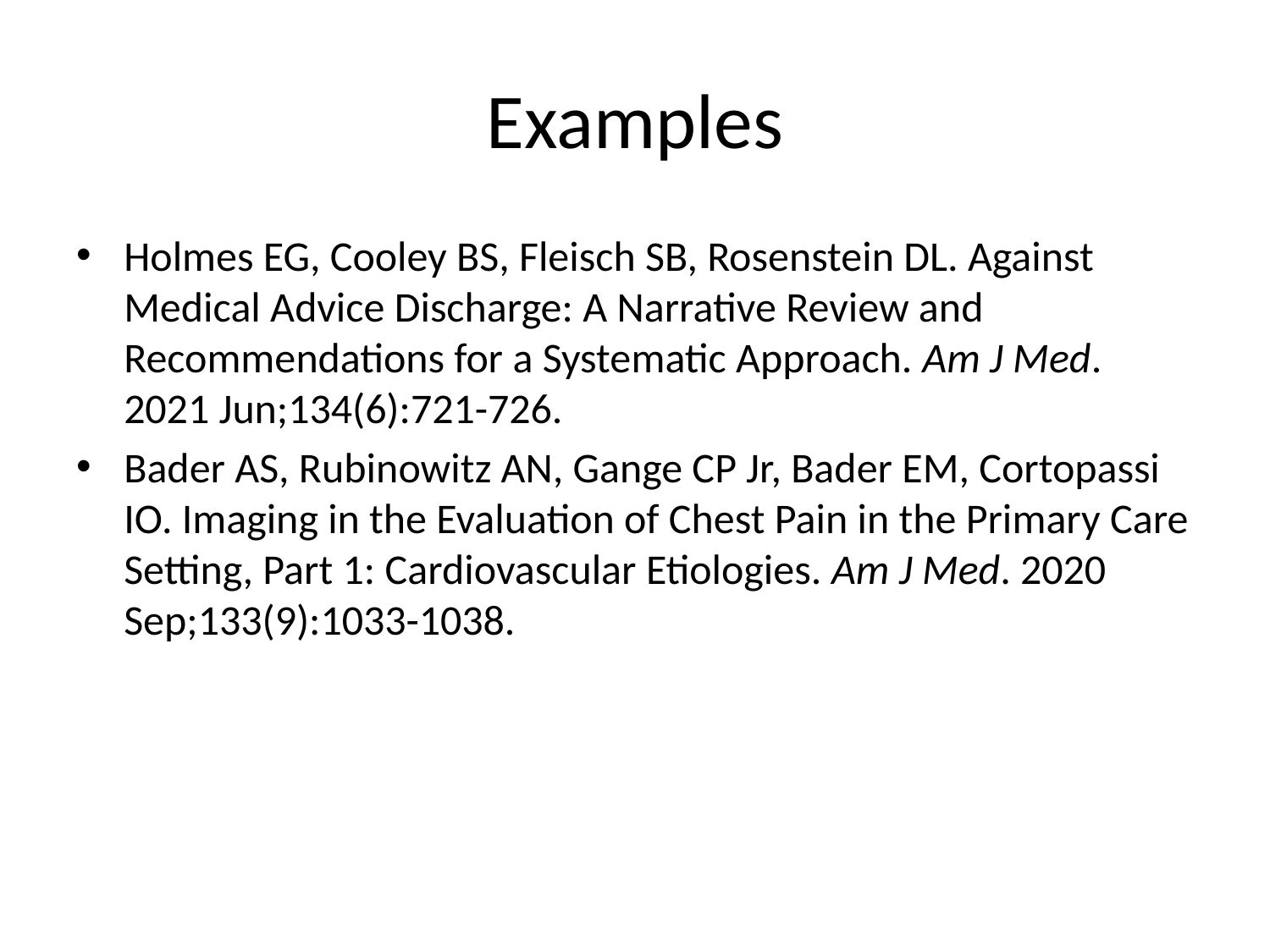

# Examples
Holmes EG, Cooley BS, Fleisch SB, Rosenstein DL. Against Medical Advice Discharge: A Narrative Review and Recommendations for a Systematic Approach. Am J Med. 2021 Jun;134(6):721-726.
Bader AS, Rubinowitz AN, Gange CP Jr, Bader EM, Cortopassi IO. Imaging in the Evaluation of Chest Pain in the Primary Care Setting, Part 1: Cardiovascular Etiologies. Am J Med. 2020 Sep;133(9):1033-1038.

## Slide 28
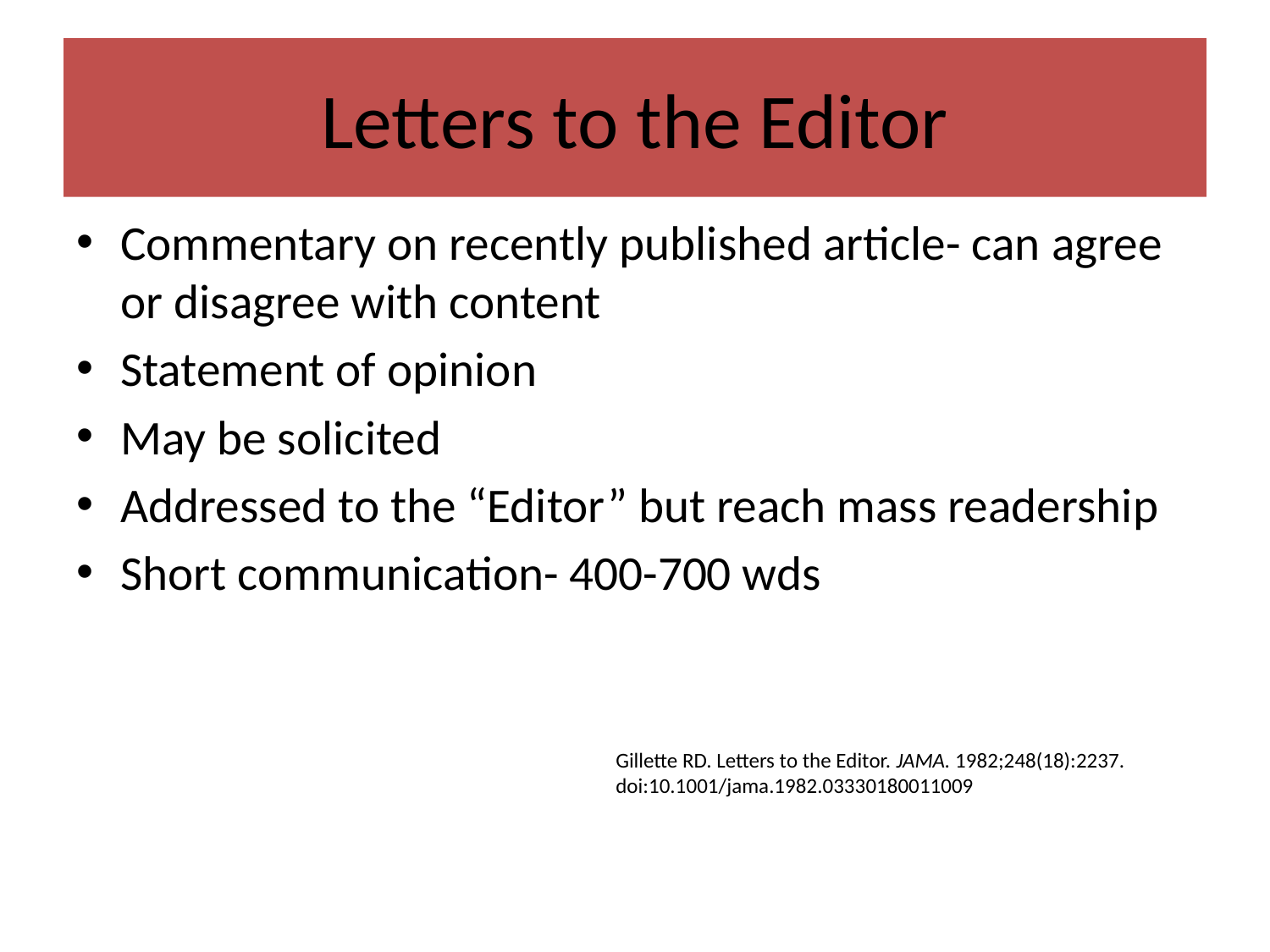

# Letters to the Editor
Commentary on recently published article- can agree or disagree with content
Statement of opinion
May be solicited
Addressed to the “Editor” but reach mass readership
Short communication- 400-700 wds
Gillette RD. Letters to the Editor. JAMA. 1982;248(18):2237. doi:10.1001/jama.1982.03330180011009

## Slide 29
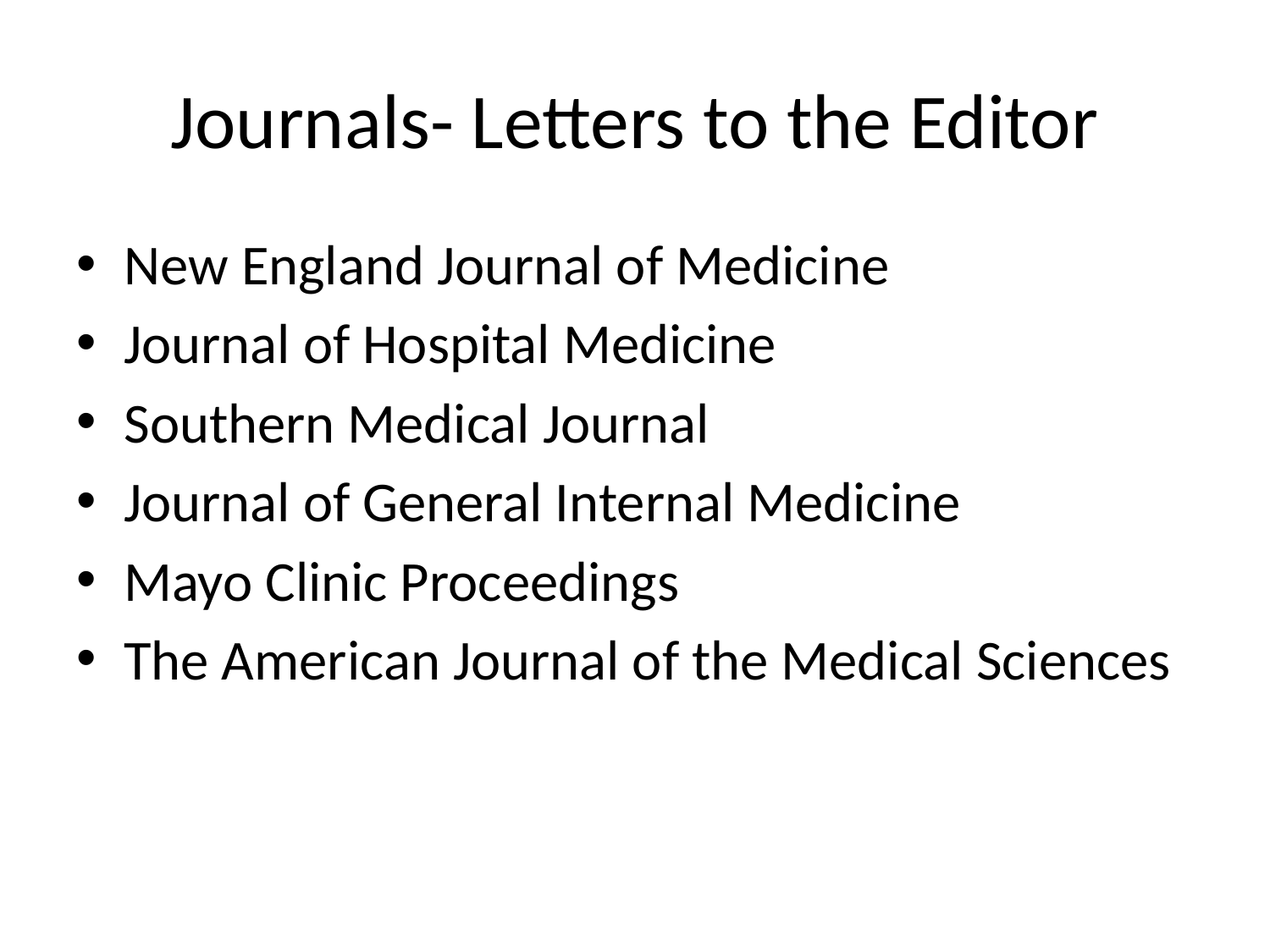

# Journals- Letters to the Editor
New England Journal of Medicine
Journal of Hospital Medicine
Southern Medical Journal
Journal of General Internal Medicine
Mayo Clinic Proceedings
The American Journal of the Medical Sciences

## Slide 30
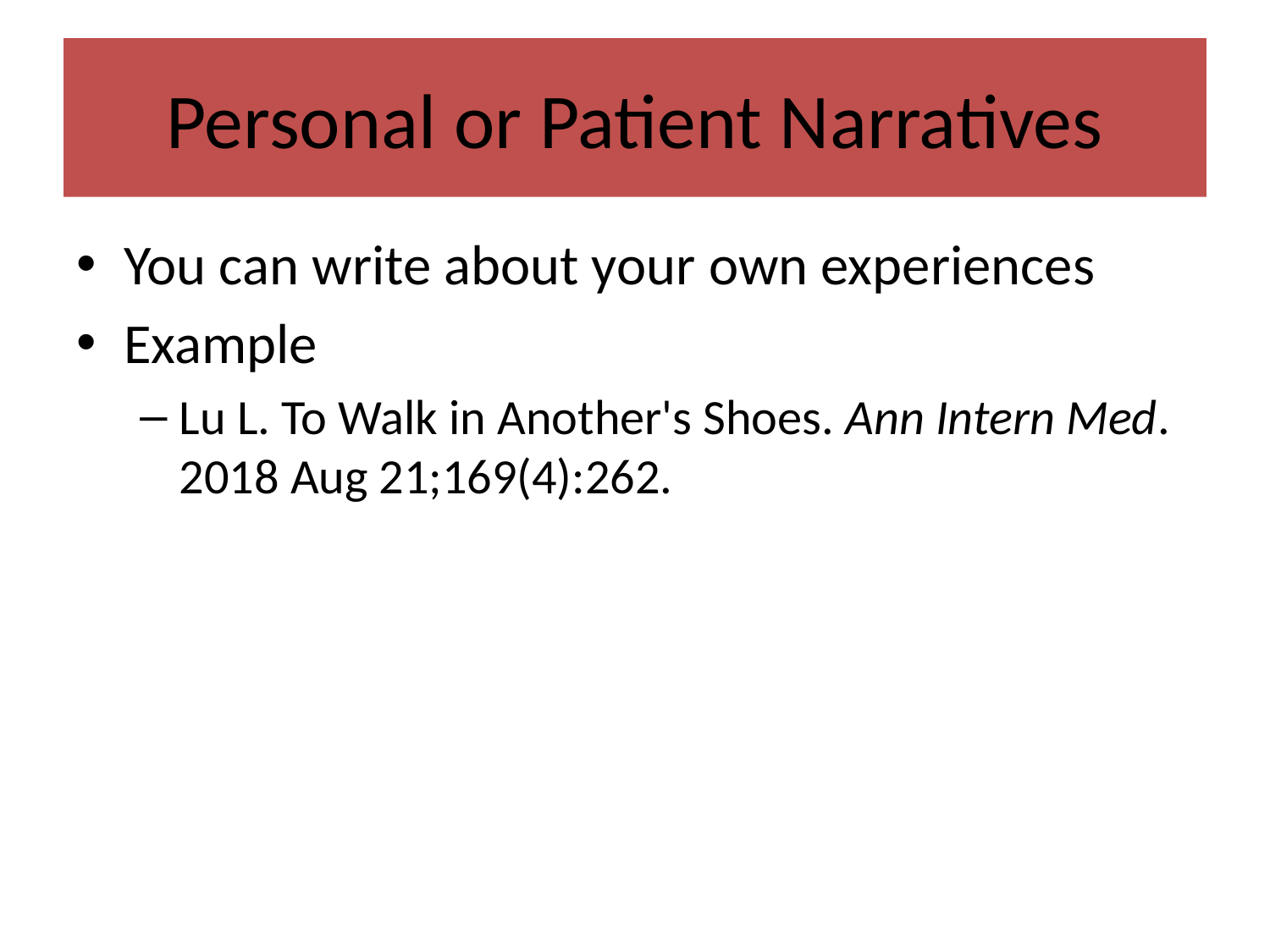

# Personal or Patient Narratives
You can write about your own experiences
Example
Lu L. To Walk in Another's Shoes. Ann Intern Med. 2018 Aug 21;169(4):262.

## Slide 31
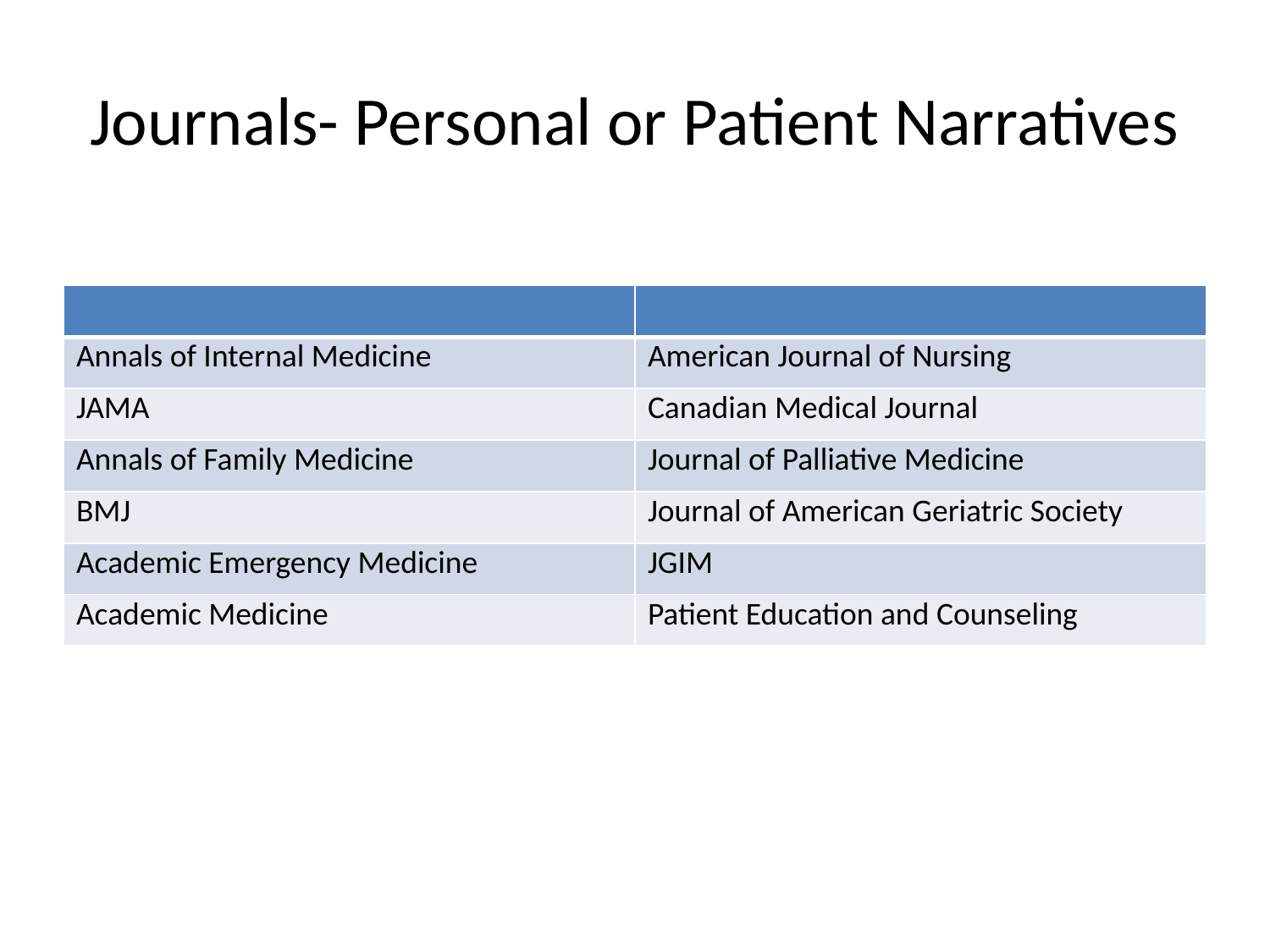

# Journals- Personal or Patient Narratives
| | |
| --- | --- |
| Annals of Internal Medicine | American Journal of Nursing |
| JAMA | Canadian Medical Journal |
| Annals of Family Medicine | Journal of Palliative Medicine |
| BMJ | Journal of American Geriatric Society |
| Academic Emergency Medicine | JGIM |
| Academic Medicine | Patient Education and Counseling |

## Slide 32
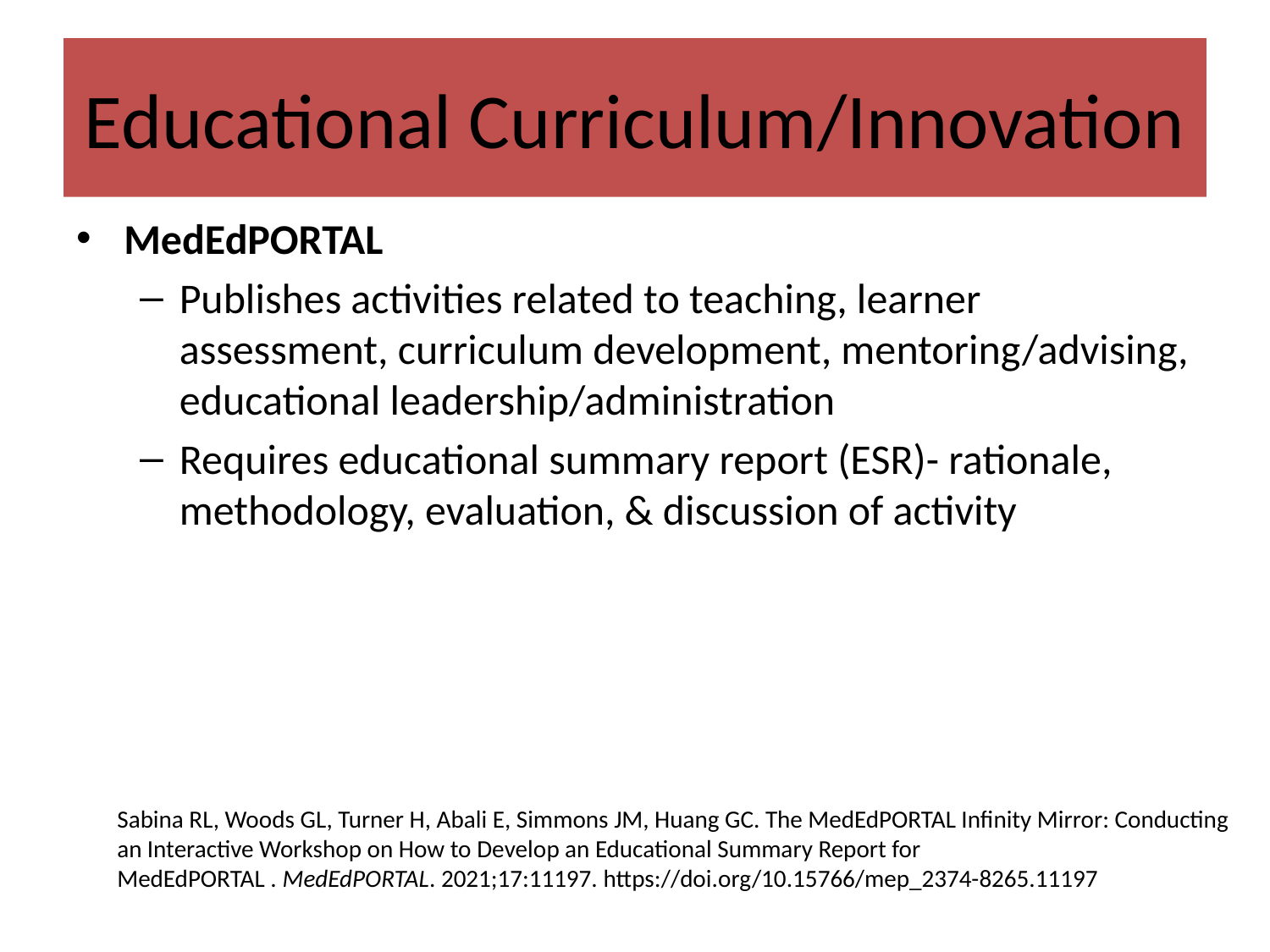

# Educational Curriculum/Innovation
MedEdPORTAL
Publishes activities related to teaching, learner assessment, curriculum development, mentoring/advising, educational leadership/administration
Requires educational summary report (ESR)- rationale, methodology, evaluation, & discussion of activity
Sabina RL, Woods GL, Turner H, Abali E, Simmons JM, Huang GC. The MedEdPORTAL Infinity Mirror: Conducting an Interactive Workshop on How to Develop an Educational Summary Report for MedEdPORTAL . MedEdPORTAL. 2021;17:11197. https://doi.org/10.15766/mep_2374-8265.11197

## Slide 33
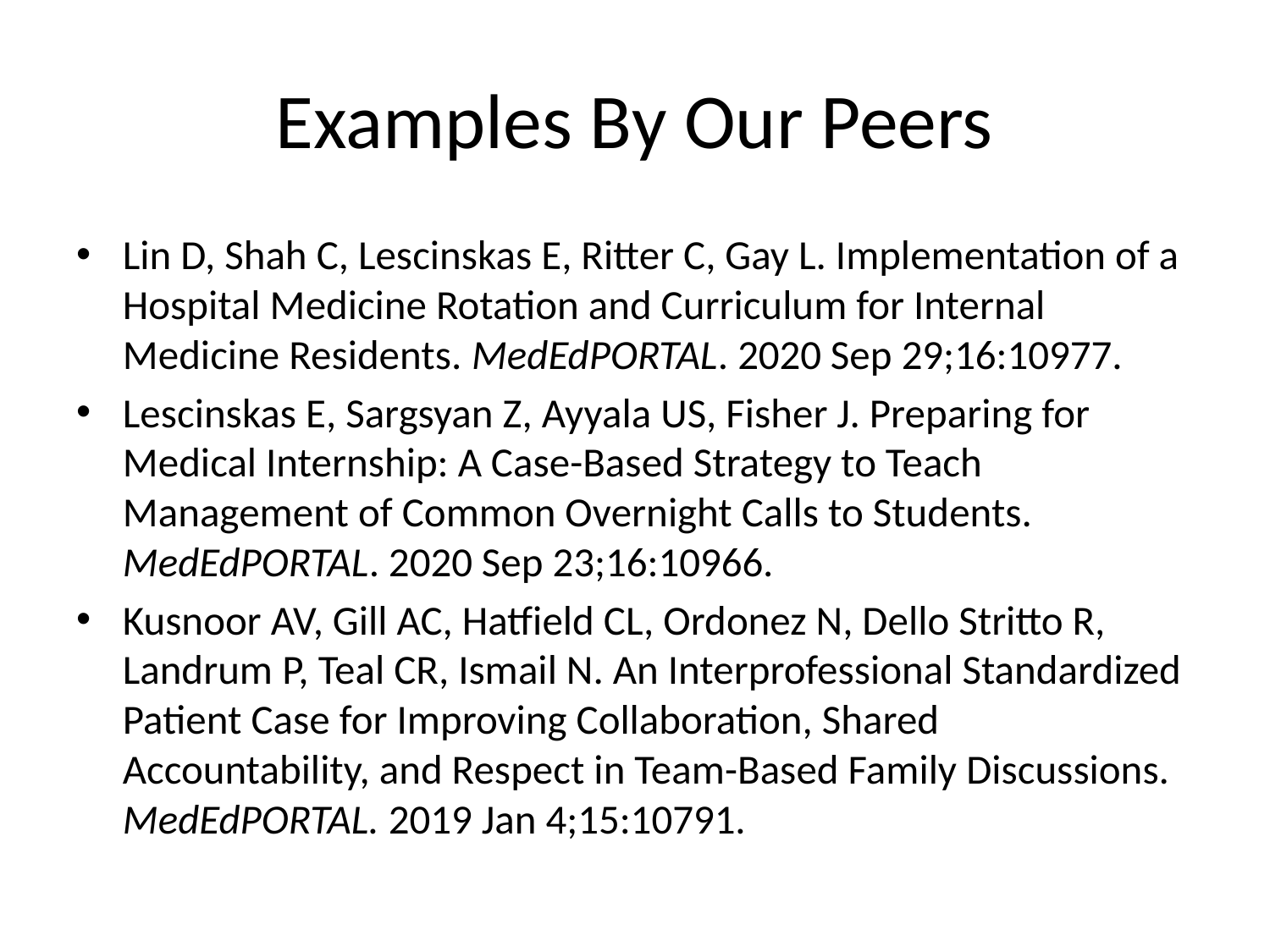

# Examples By Our Peers
Lin D, Shah C, Lescinskas E, Ritter C, Gay L. Implementation of a Hospital Medicine Rotation and Curriculum for Internal Medicine Residents. MedEdPORTAL. 2020 Sep 29;16:10977.
Lescinskas E, Sargsyan Z, Ayyala US, Fisher J. Preparing for Medical Internship: A Case-Based Strategy to Teach Management of Common Overnight Calls to Students. MedEdPORTAL. 2020 Sep 23;16:10966.
Kusnoor AV, Gill AC, Hatfield CL, Ordonez N, Dello Stritto R, Landrum P, Teal CR, Ismail N. An Interprofessional Standardized Patient Case for Improving Collaboration, Shared Accountability, and Respect in Team-Based Family Discussions. MedEdPORTAL. 2019 Jan 4;15:10791.

## Slide 34
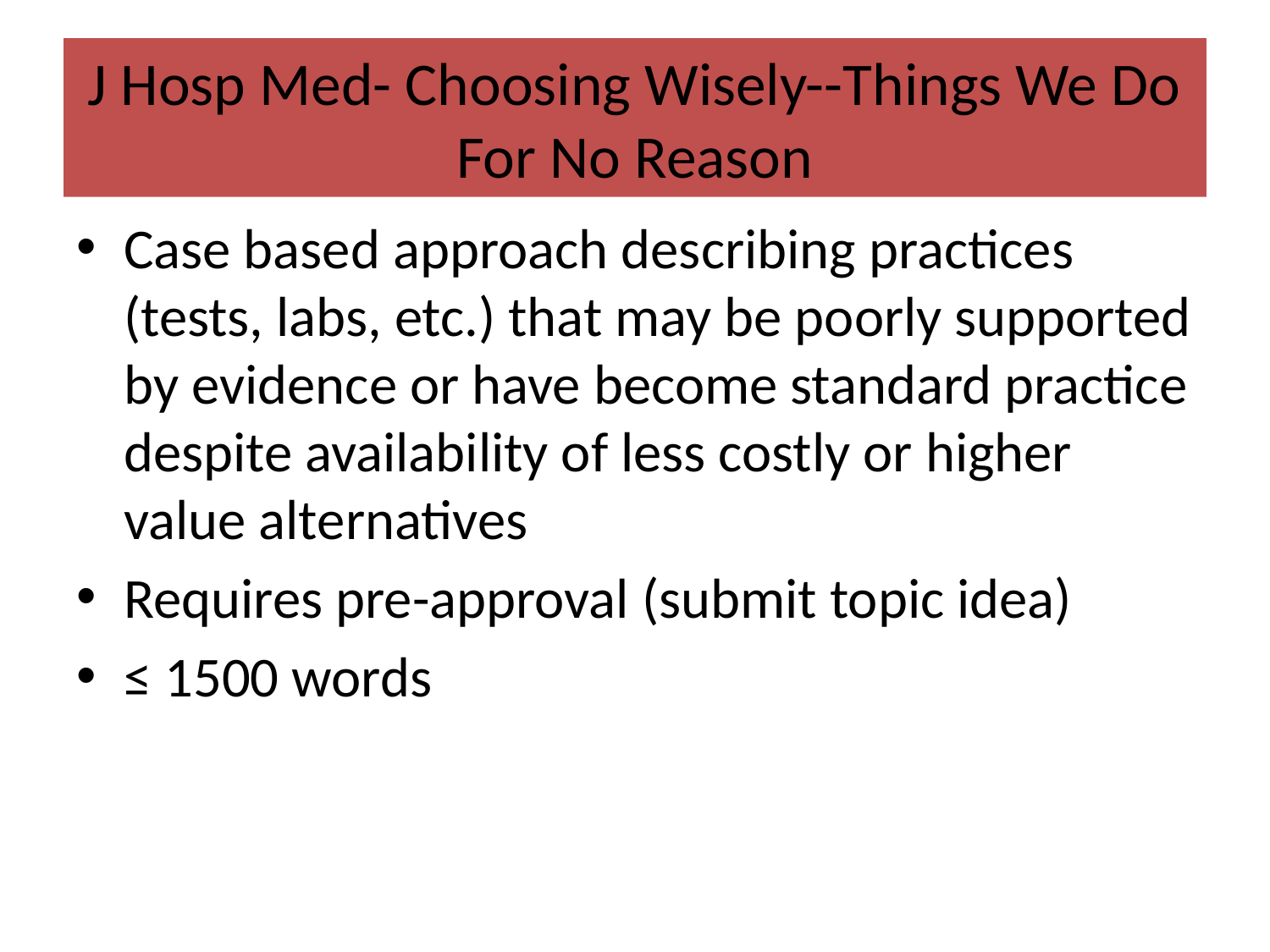

# J Hosp Med- Choosing Wisely--Things We Do For No Reason
Case based approach describing practices (tests, labs, etc.) that may be poorly supported by evidence or have become standard practice despite availability of less costly or higher value alternatives
Requires pre-approval (submit topic idea)
≤ 1500 words

## Slide 35
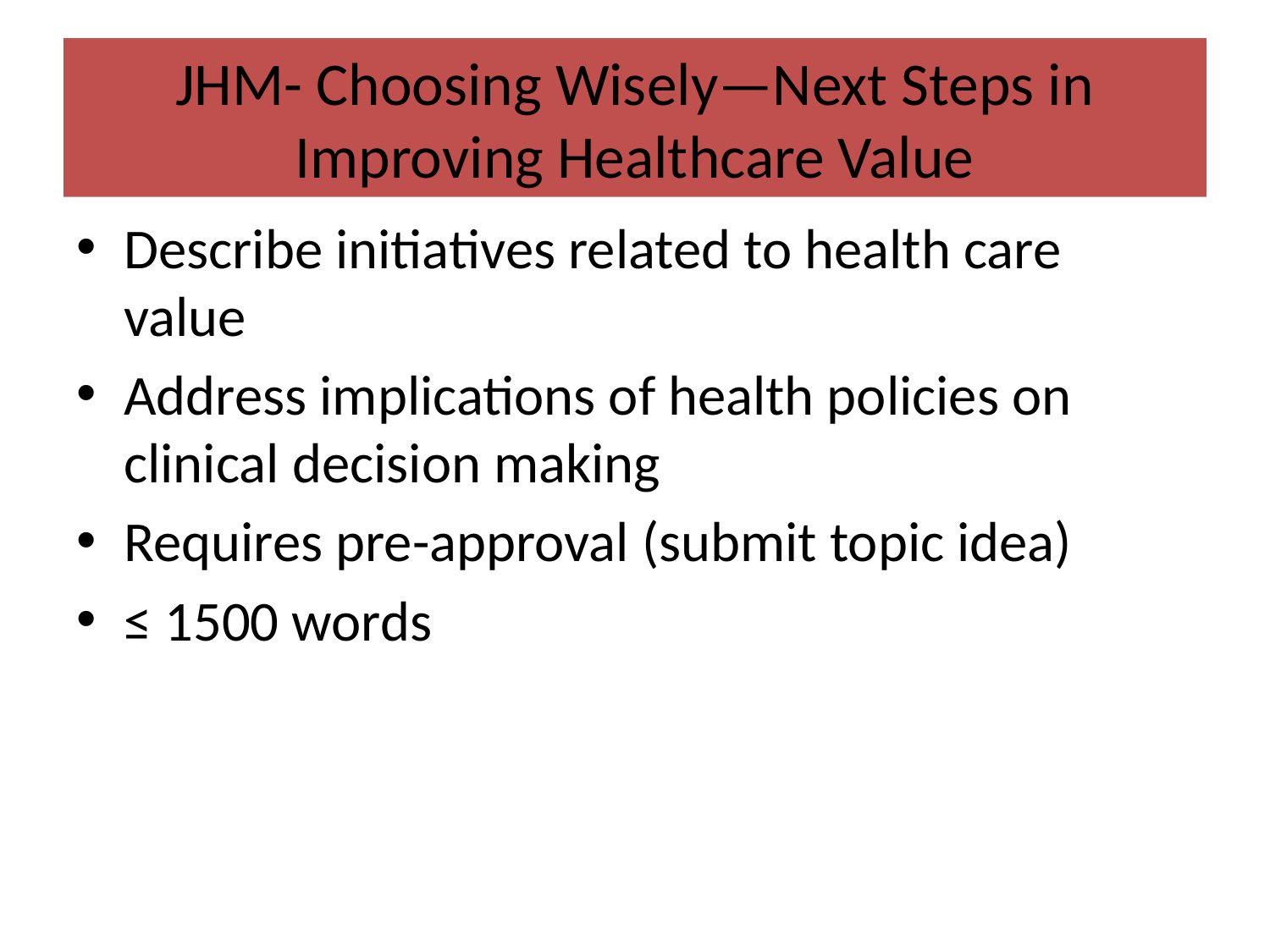

# JHM- Choosing Wisely—Next Steps in Improving Healthcare Value
Describe initiatives related to health care value
Address implications of health policies on clinical decision making
Requires pre-approval (submit topic idea)
≤ 1500 words

## Slide 36
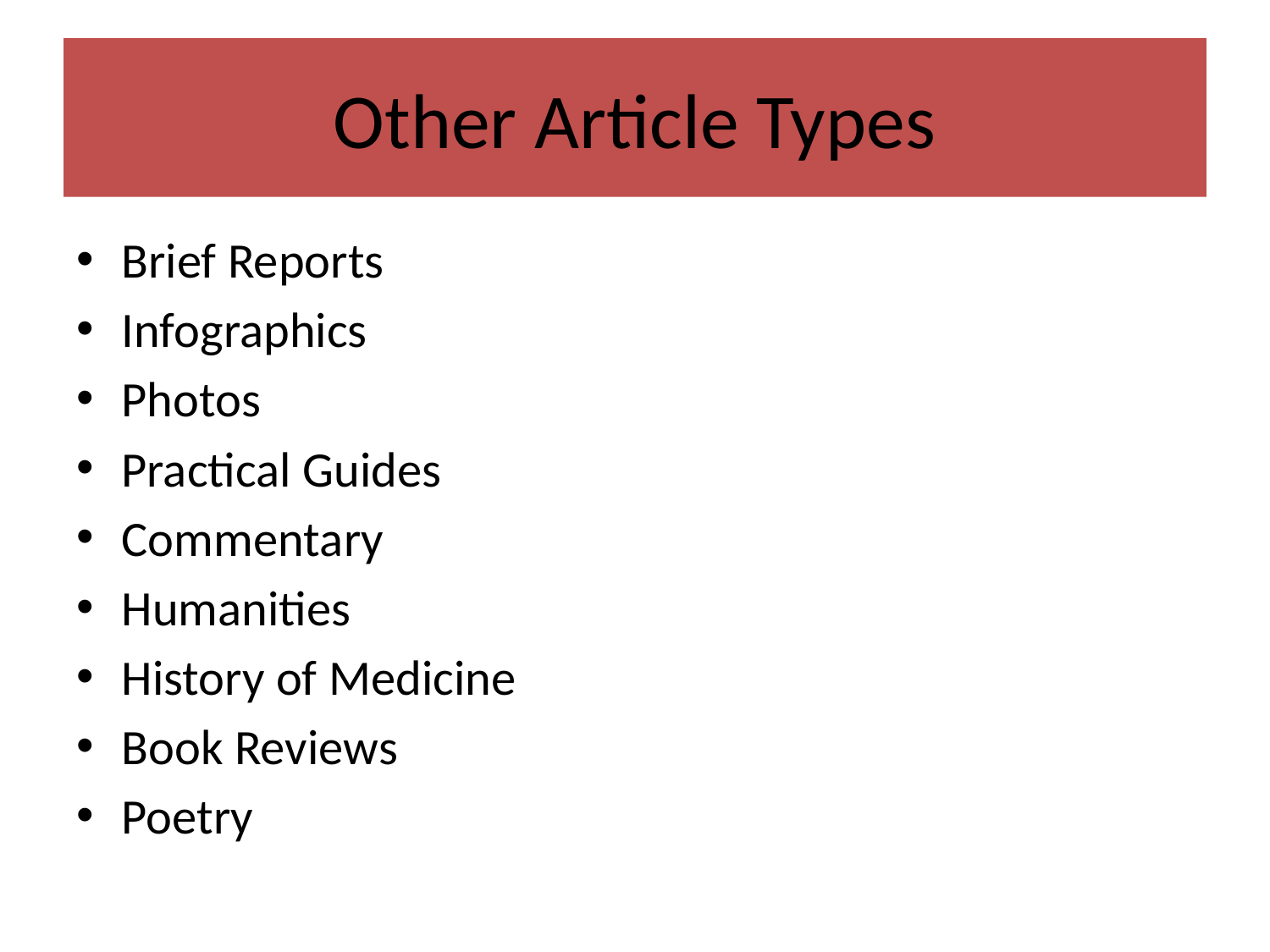

# Other Article Types
Brief Reports
Infographics
Photos
Practical Guides
Commentary
Humanities
History of Medicine
Book Reviews
Poetry

## Slide 37
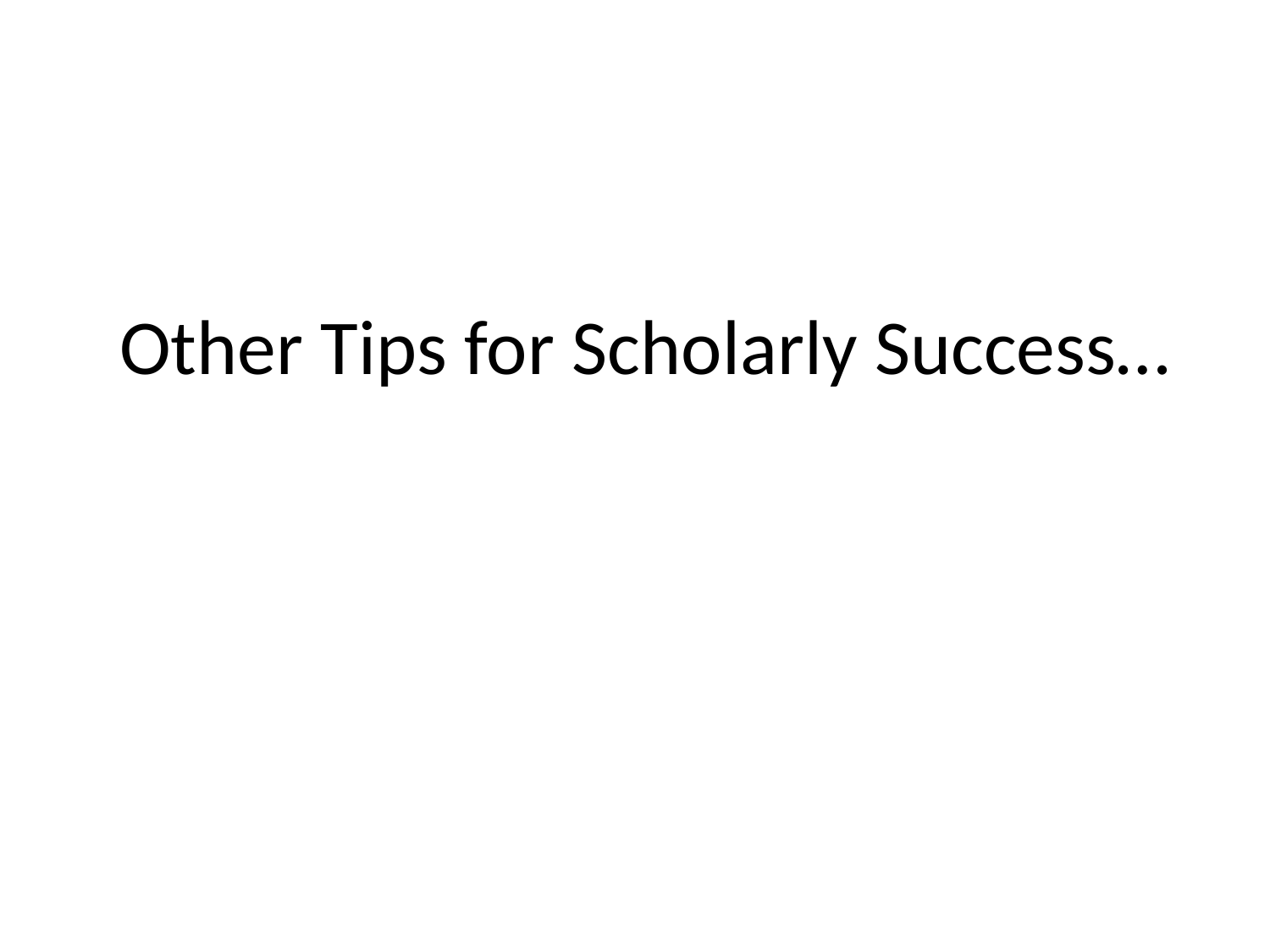

# Other Tips for Scholarly Success…

## Slide 38
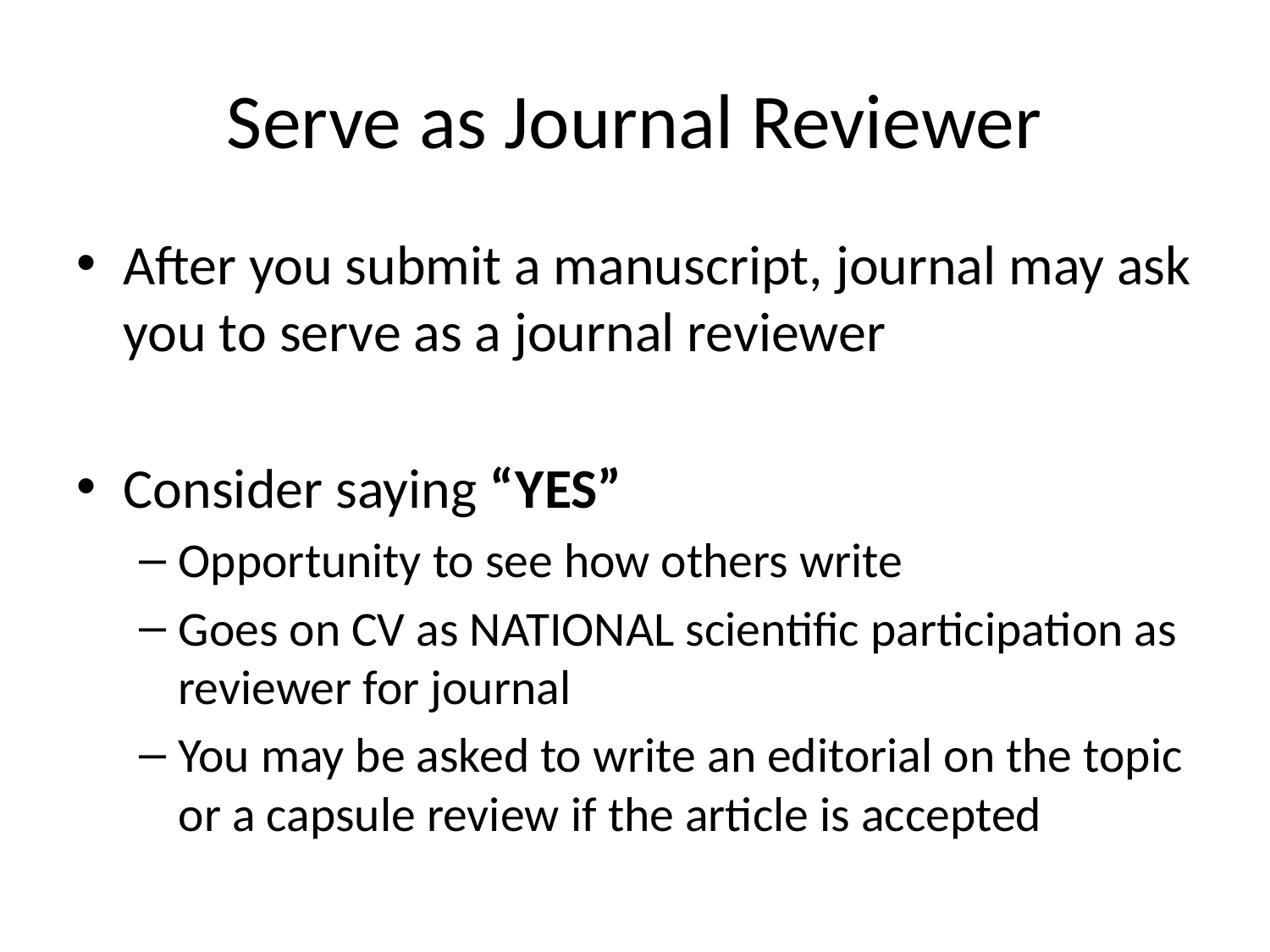

# Serve as Journal Reviewer
After you submit a manuscript, journal may ask you to serve as a journal reviewer
Consider saying “YES”
Opportunity to see how others write
Goes on CV as NATIONAL scientific participation as reviewer for journal
You may be asked to write an editorial on the topic or a capsule review if the article is accepted

## Slide 39
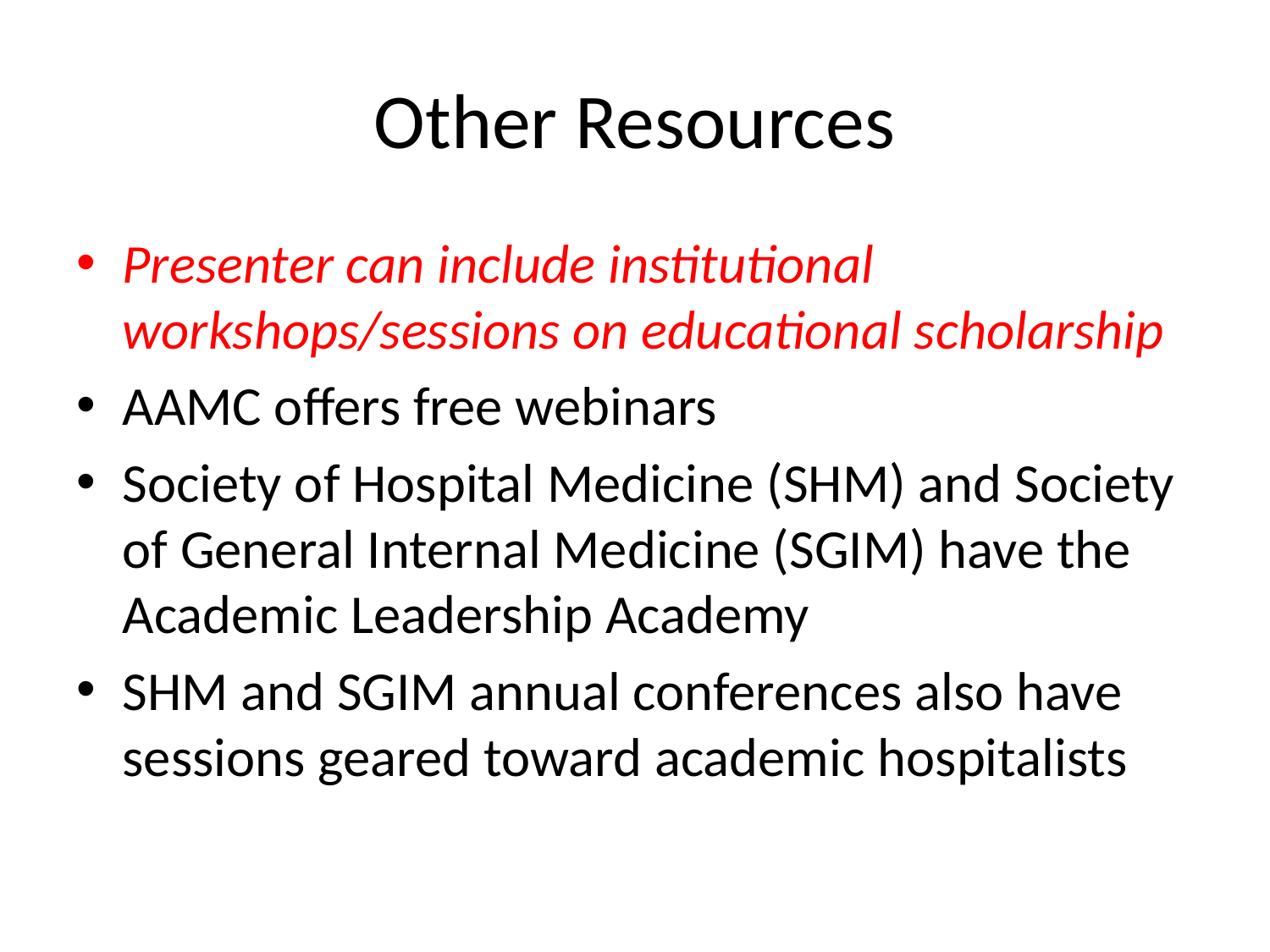

# Other Resources
Presenter can include institutional workshops/sessions on educational scholarship
AAMC offers free webinars
Society of Hospital Medicine (SHM) and Society of General Internal Medicine (SGIM) have the Academic Leadership Academy
SHM and SGIM annual conferences also have sessions geared toward academic hospitalists

## Slide 40
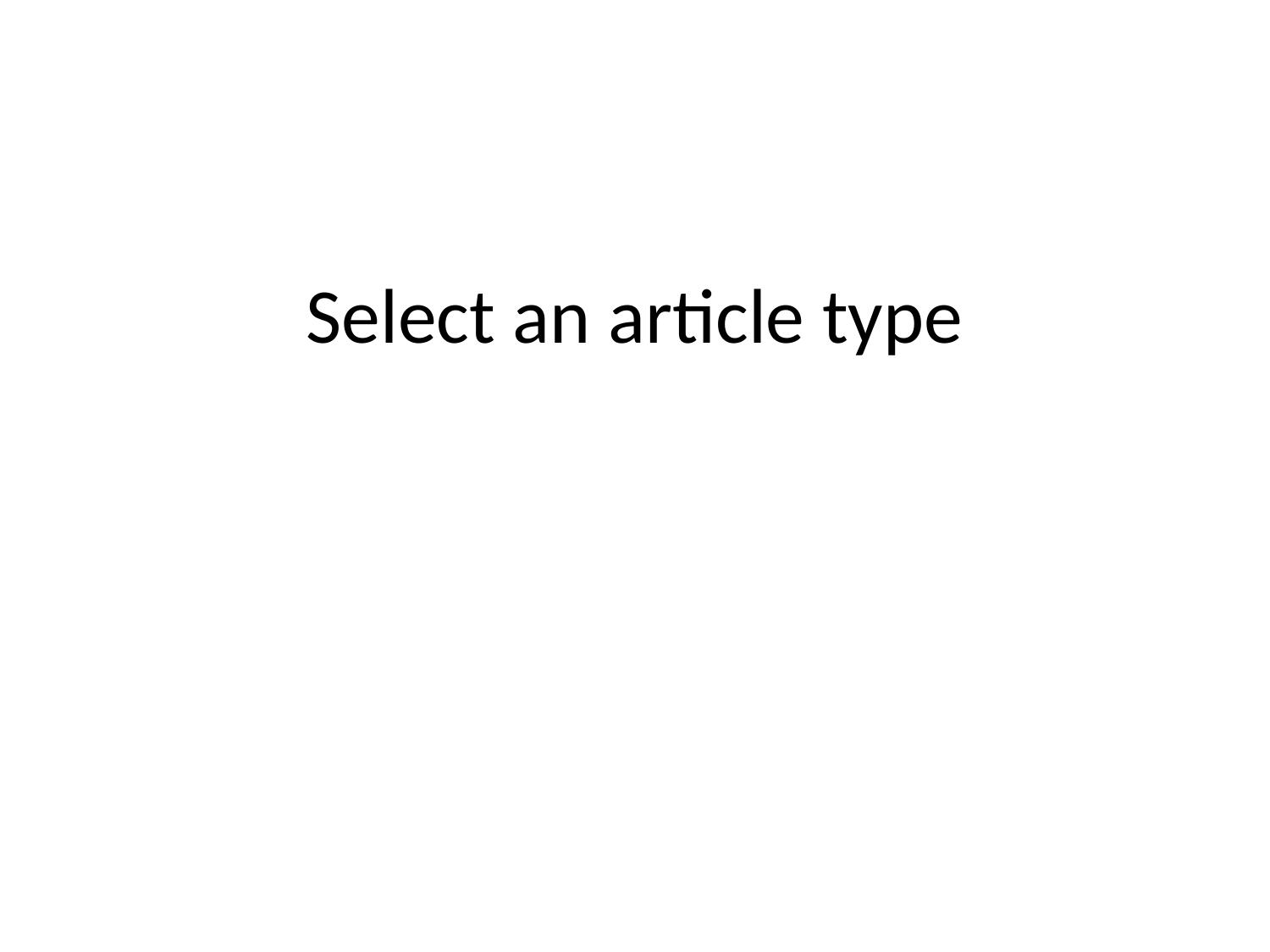

# Select an article type

## Slide 41
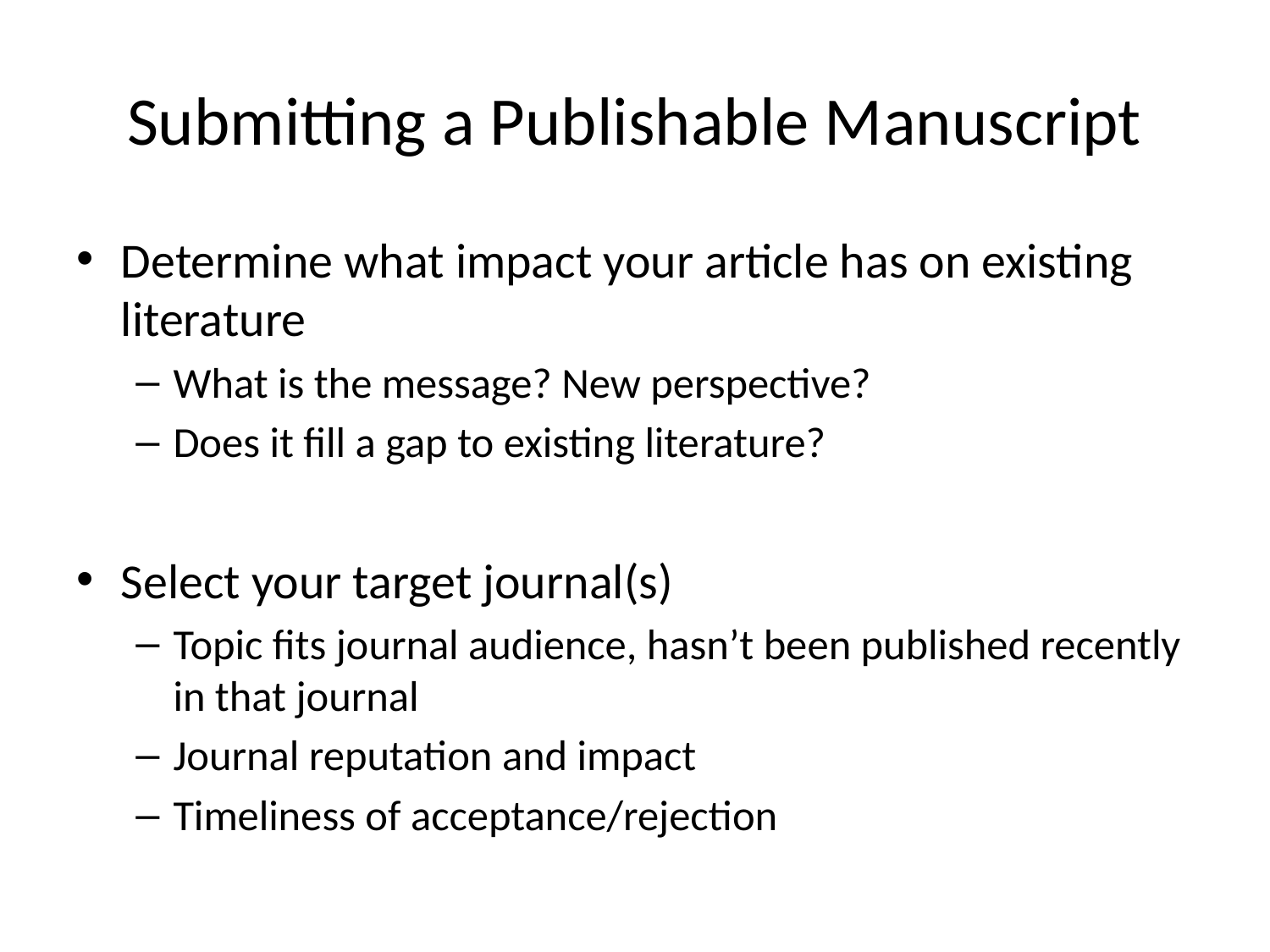

# Submitting a Publishable Manuscript
Determine what impact your article has on existing literature
What is the message? New perspective?
Does it fill a gap to existing literature?
Select your target journal(s)
Topic fits journal audience, hasn’t been published recently in that journal
Journal reputation and impact
Timeliness of acceptance/rejection

## Slide 42
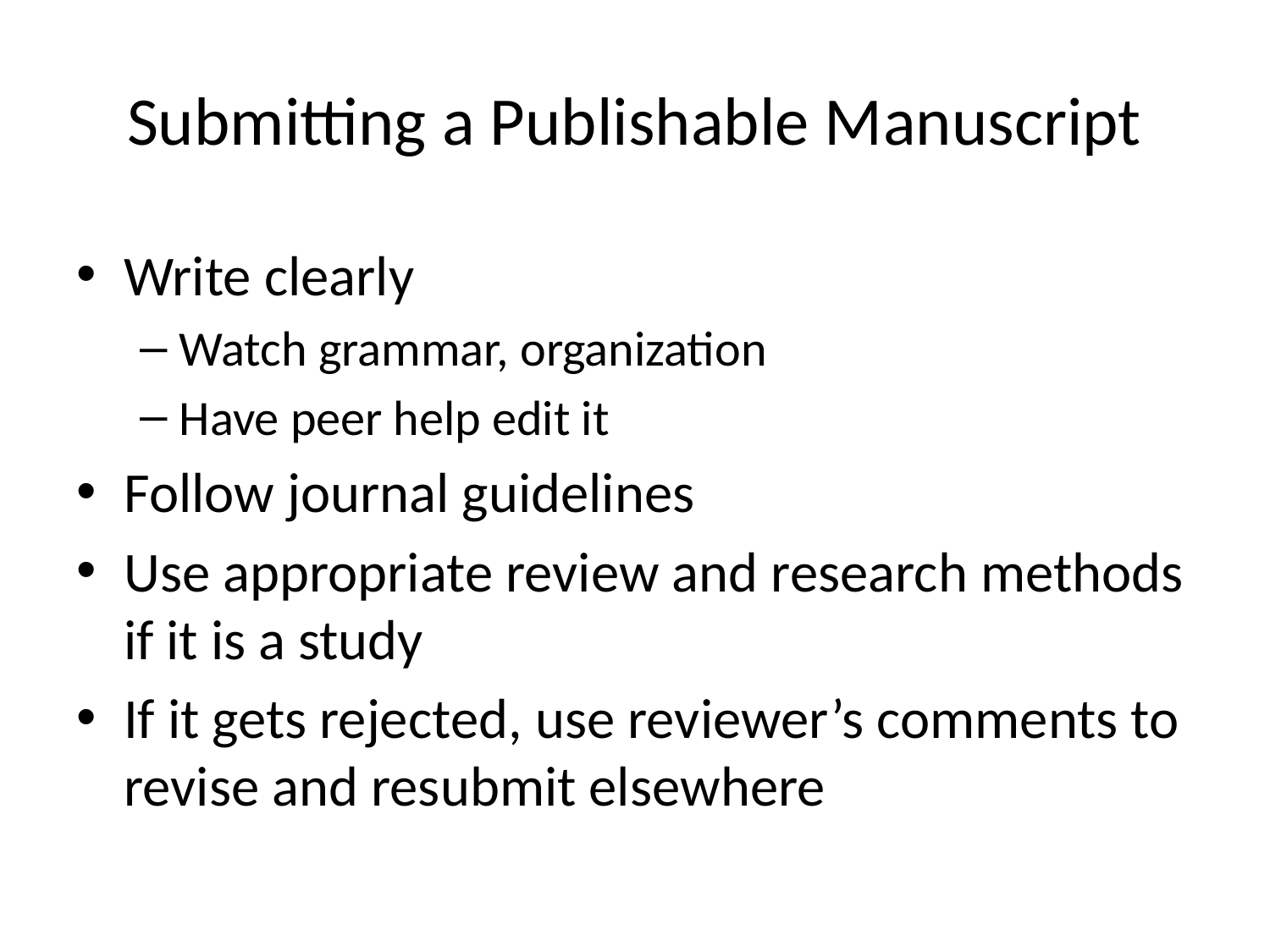

# Submitting a Publishable Manuscript
Write clearly
Watch grammar, organization
Have peer help edit it
Follow journal guidelines
Use appropriate review and research methods if it is a study
If it gets rejected, use reviewer’s comments to revise and resubmit elsewhere

## Slide 43
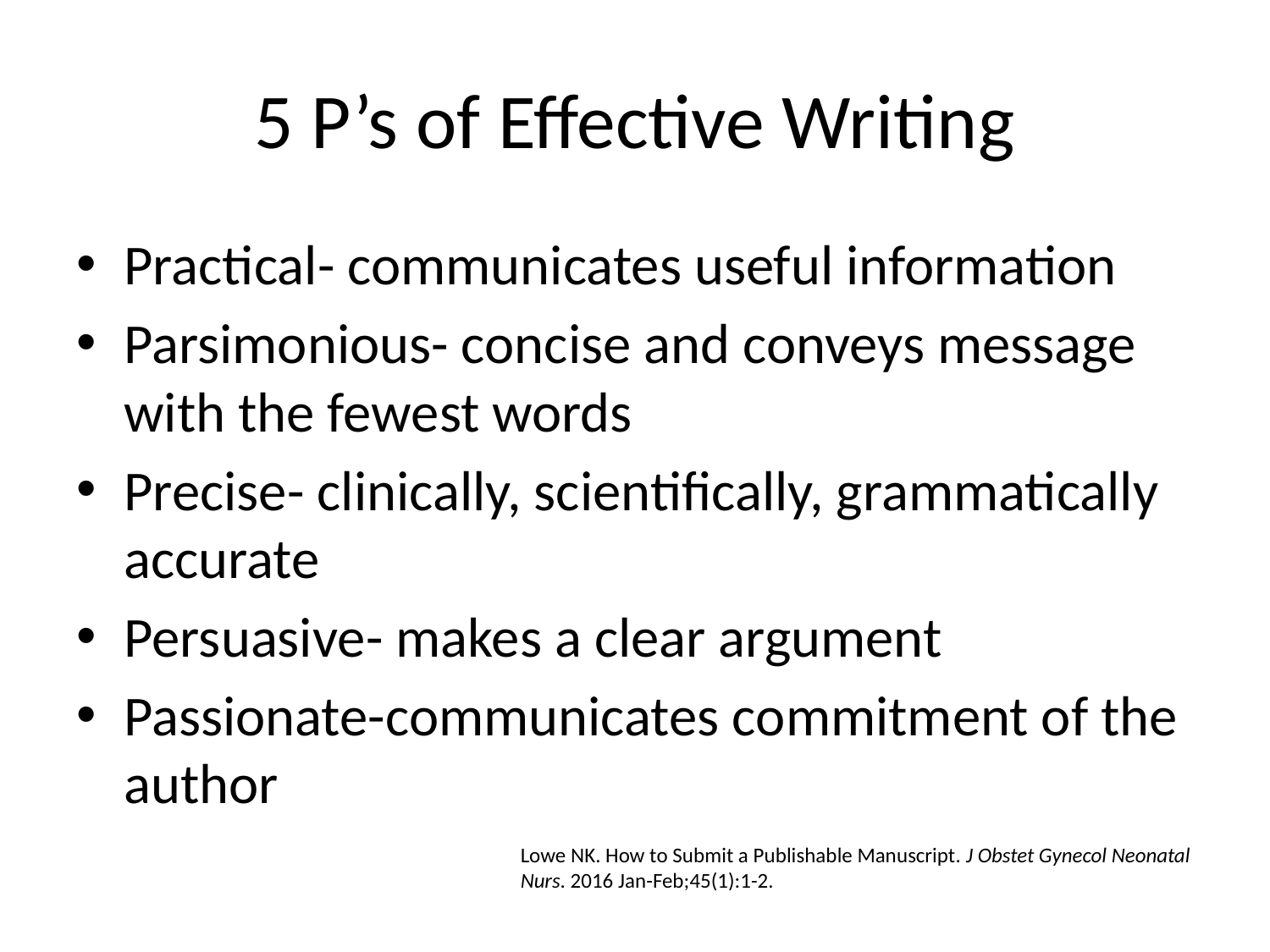

# 5 P’s of Effective Writing
Practical- communicates useful information
Parsimonious- concise and conveys message with the fewest words
Precise- clinically, scientifically, grammatically accurate
Persuasive- makes a clear argument
Passionate-communicates commitment of the author
Lowe NK. How to Submit a Publishable Manuscript. J Obstet Gynecol Neonatal Nurs. 2016 Jan-Feb;45(1):1-2.

## Slide 44
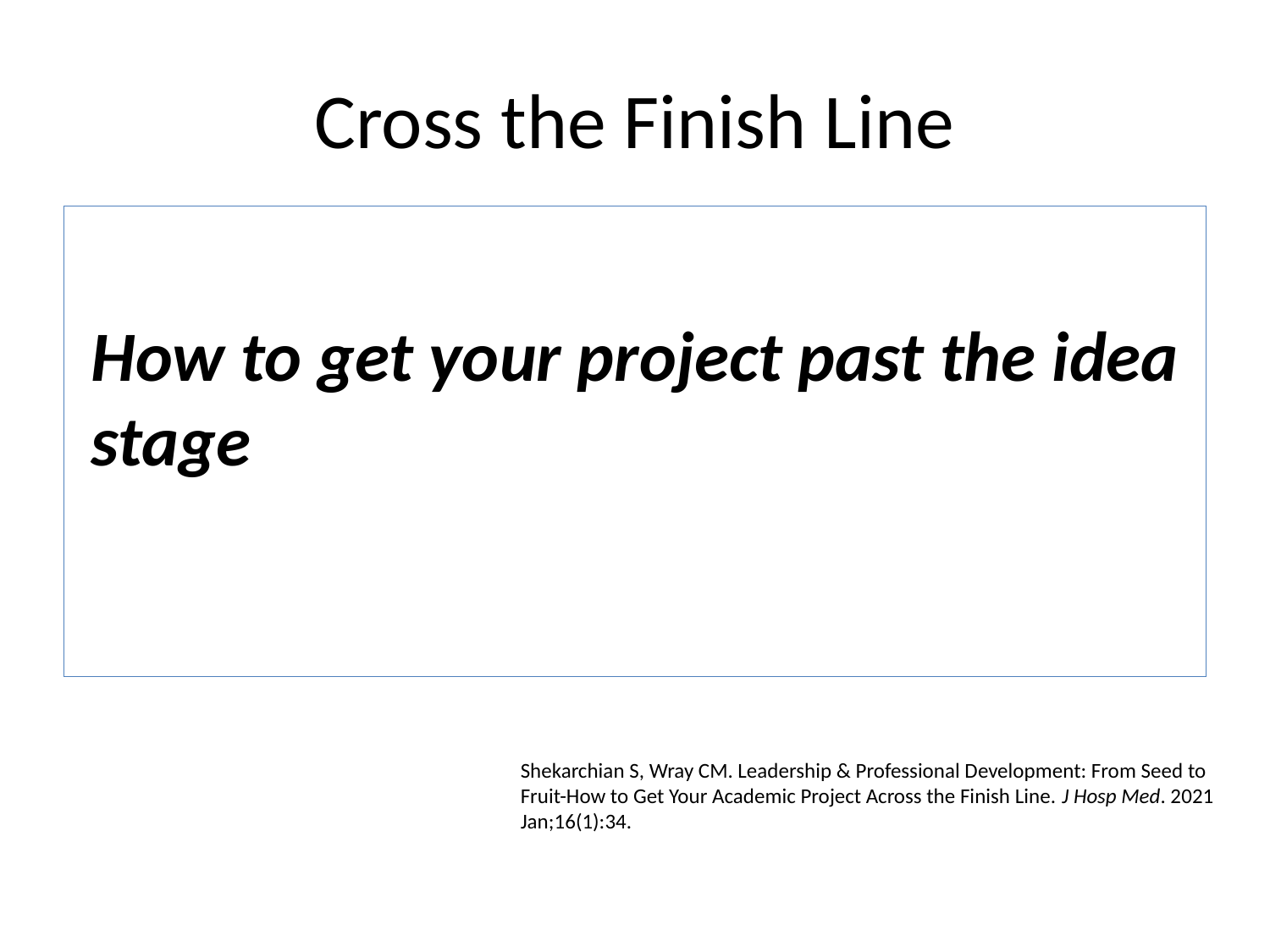

# Cross the Finish Line
How to get your project past the idea stage
Shekarchian S, Wray CM. Leadership & Professional Development: From Seed to Fruit-How to Get Your Academic Project Across the Finish Line. J Hosp Med. 2021 Jan;16(1):34.

## Slide 45
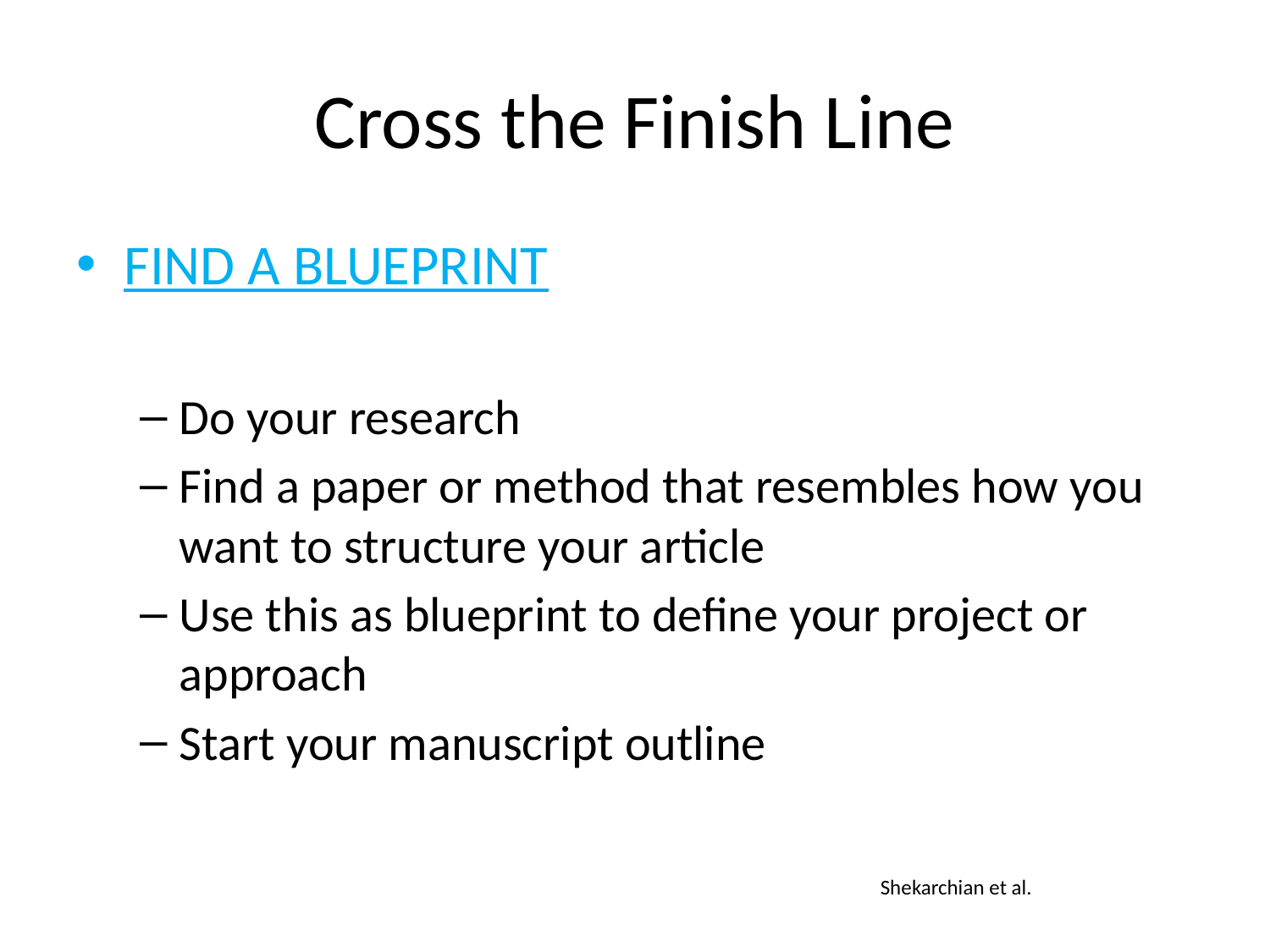

# Cross the Finish Line
FIND A BLUEPRINT
Do your research
Find a paper or method that resembles how you want to structure your article
Use this as blueprint to define your project or approach
Start your manuscript outline
Shekarchian et al.

## Slide 46
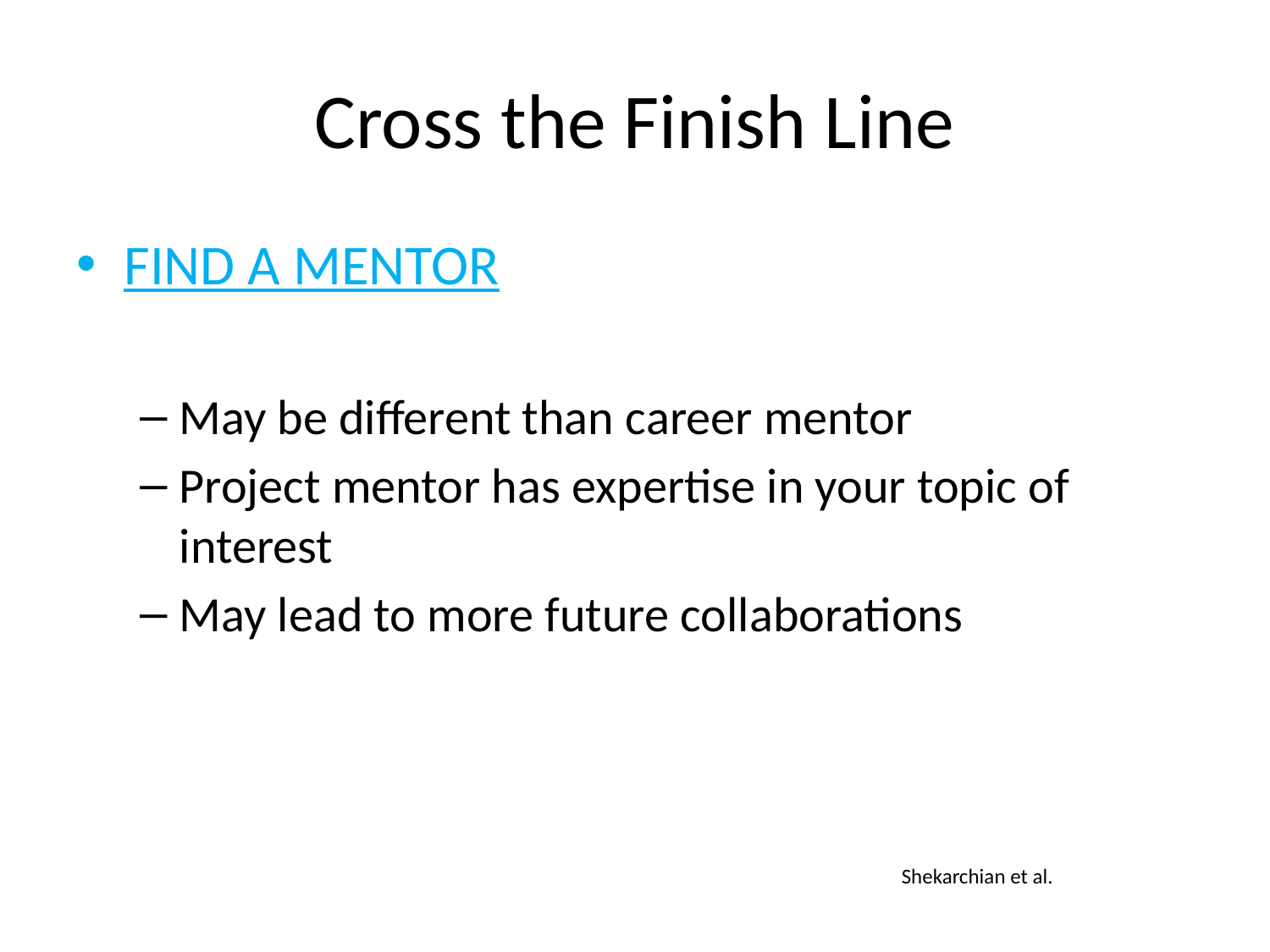

# Cross the Finish Line
FIND A MENTOR
May be different than career mentor
Project mentor has expertise in your topic of interest
May lead to more future collaborations
Shekarchian et al.

## Slide 47
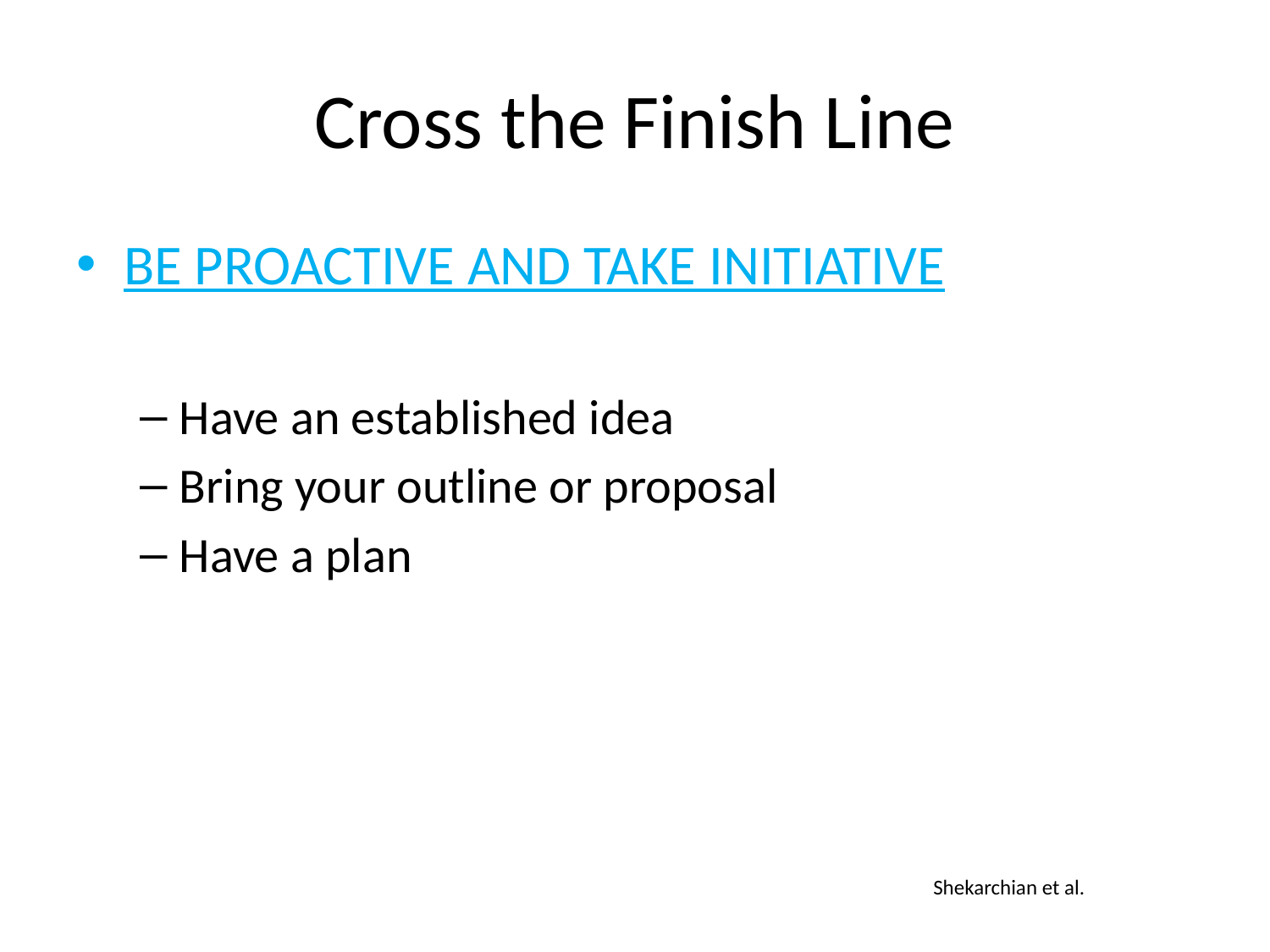

# Cross the Finish Line
BE PROACTIVE AND TAKE INITIATIVE
Have an established idea
Bring your outline or proposal
Have a plan
Shekarchian et al.

## Slide 48
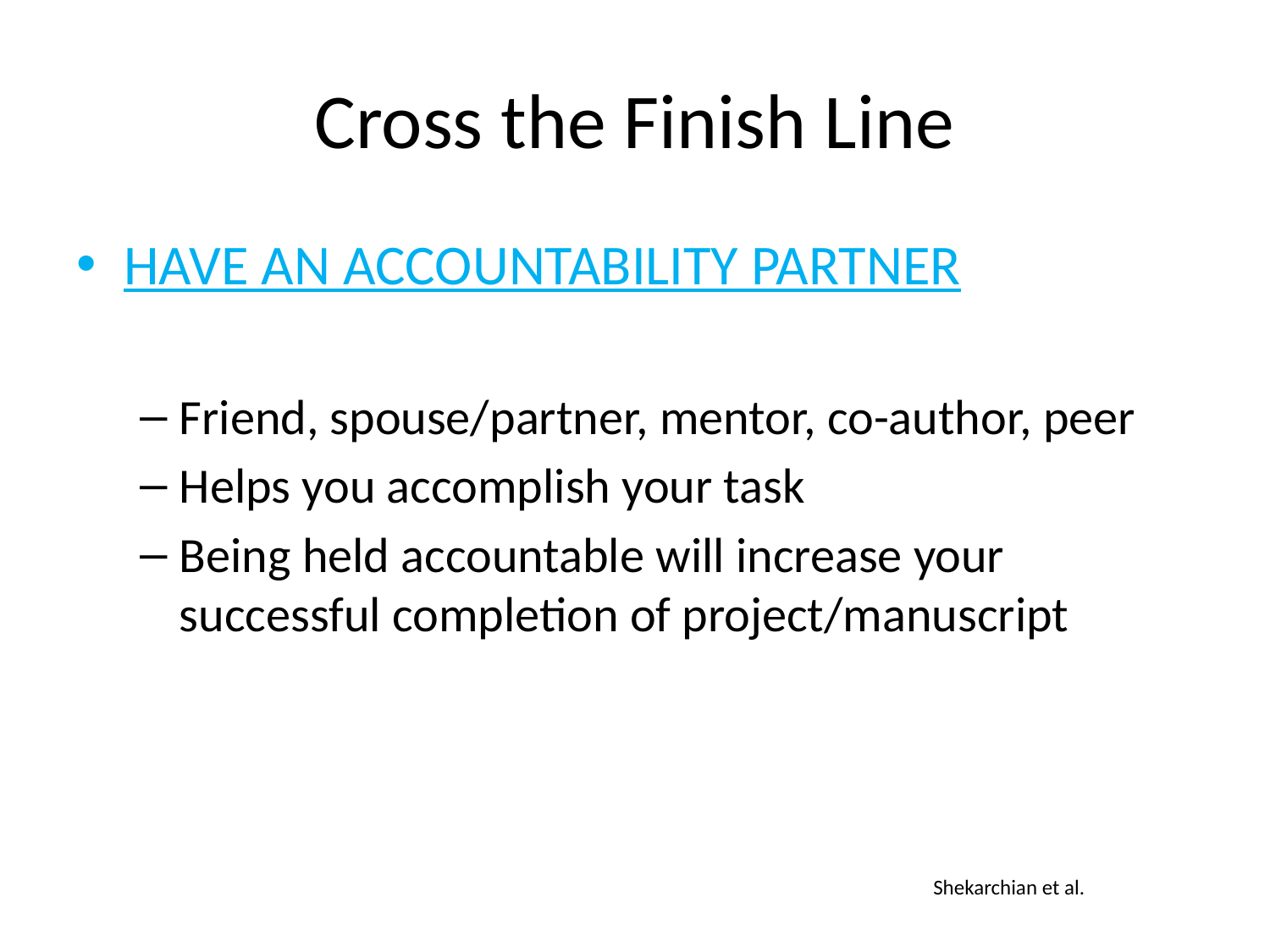

# Cross the Finish Line
HAVE AN ACCOUNTABILITY PARTNER
Friend, spouse/partner, mentor, co-author, peer
Helps you accomplish your task
Being held accountable will increase your successful completion of project/manuscript
Shekarchian et al.

## Slide 49
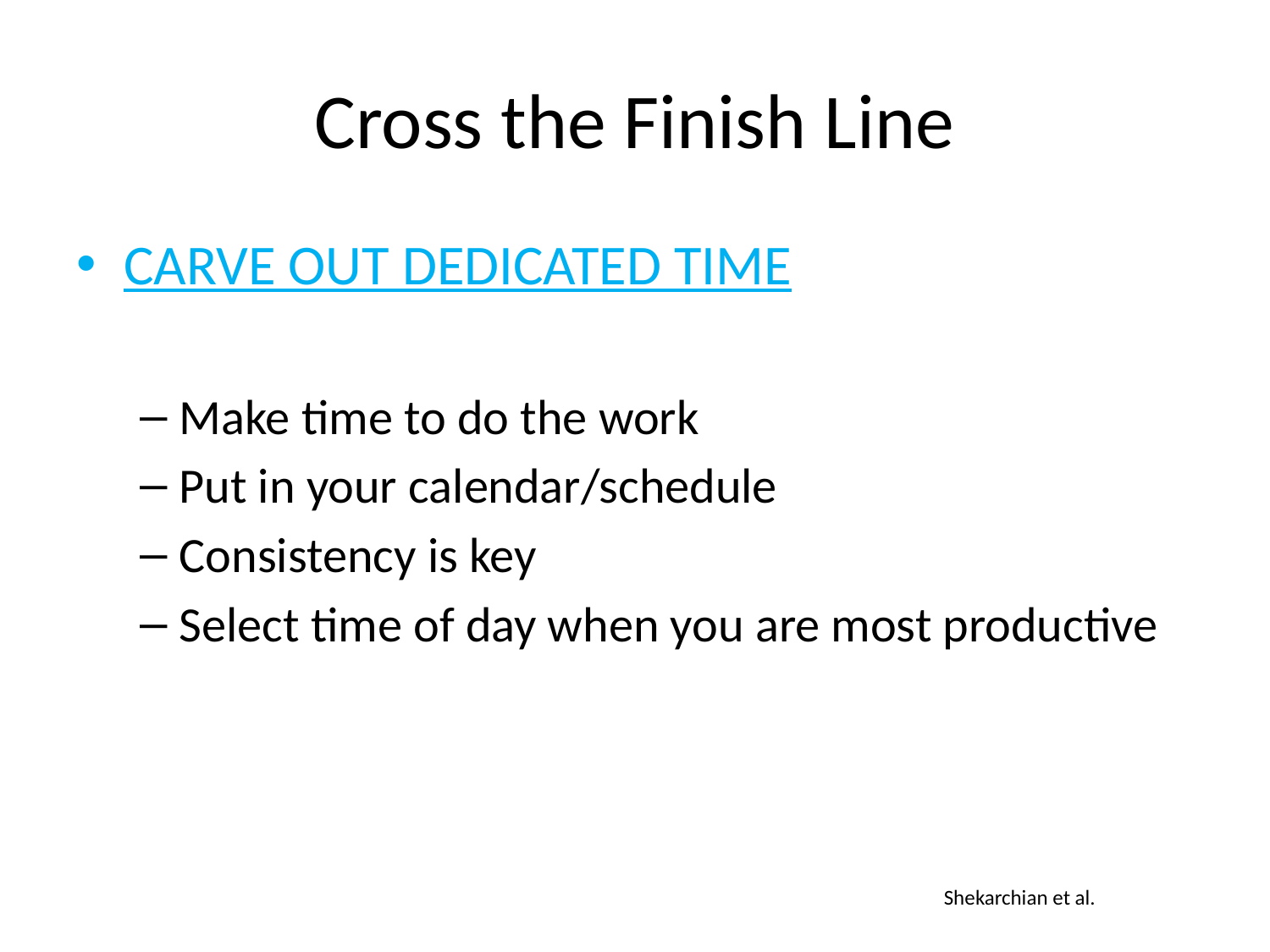

# Cross the Finish Line
CARVE OUT DEDICATED TIME
Make time to do the work
Put in your calendar/schedule
Consistency is key
Select time of day when you are most productive
Shekarchian et al.

## Slide 50
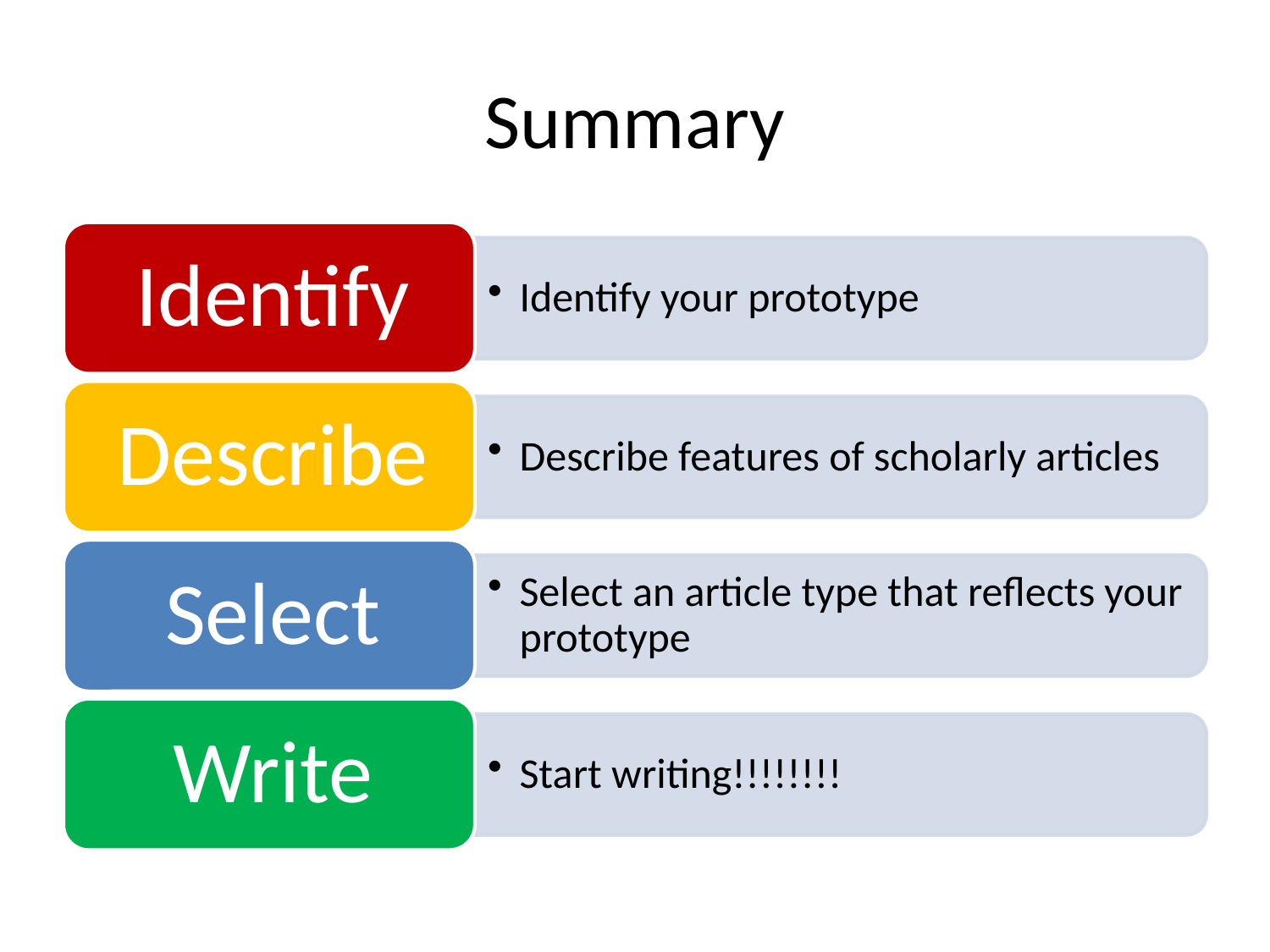

# Summary

## Slide 51
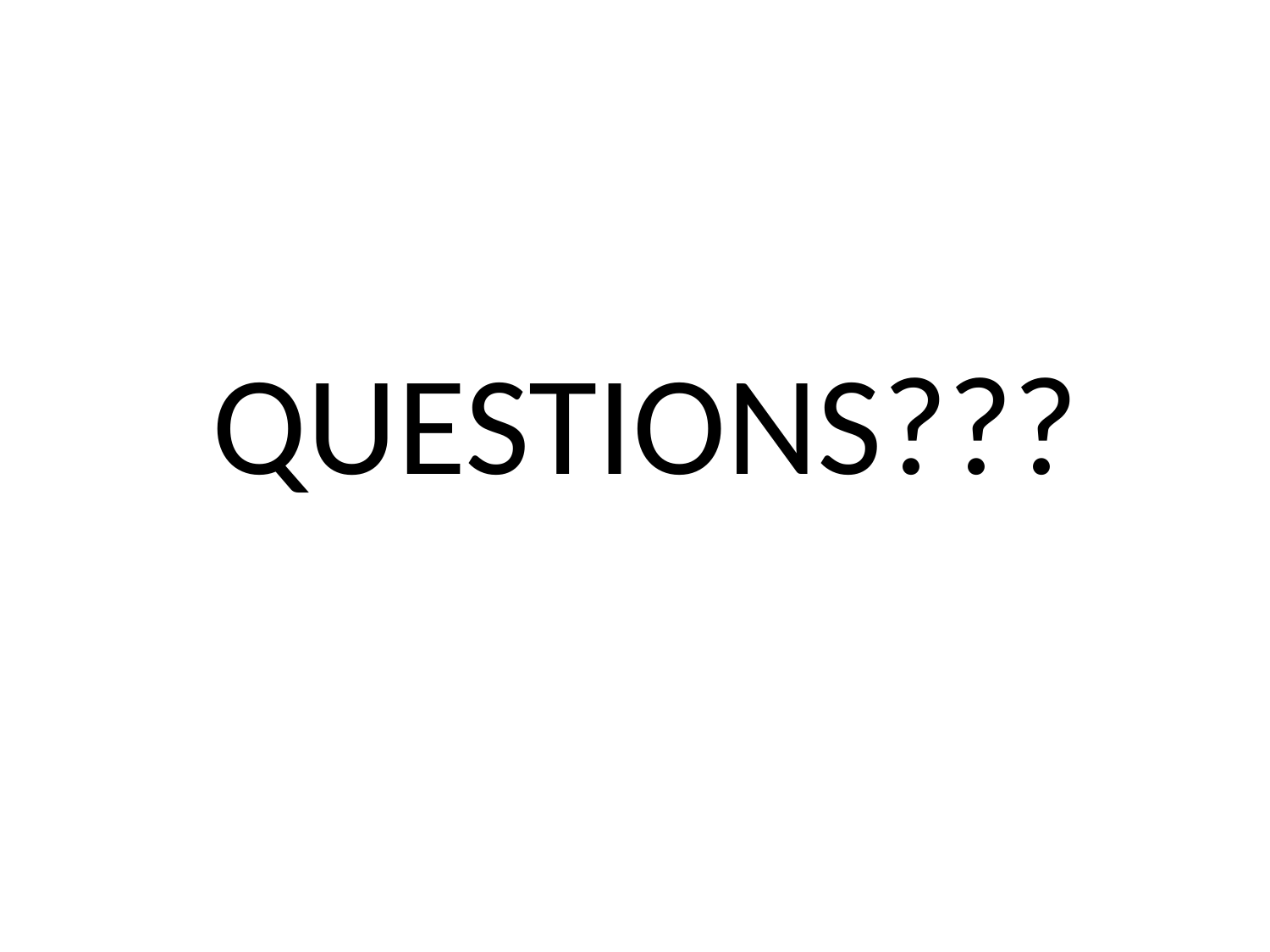

# QUESTIONS???
