# Supplementary material for: A Facilitated Peer Mentoring Program With a Dedicated Curriculum to Foster Career Advancement of Academic Hospitalists
Source: MedEdPORTAL. 2023 Dec 8;19:11366. doi: 10.15766/mep_2374-8265.11366 (PMC10704005; doi:10.15766/mep_2374-8265.11366)
Supplement: Supplementary file 1 — Preprogram Survey.docxPostprogram Survey.docxLarge-Group Session 1.pptxLarge-Group Session 2.pptxLarge-Group Session 3.pptxLarge-Group Session 4.pptxSmall-Group Session 1 Facilitator Guide.docxSmall-Group Session 2 Facilitator Guide.docxSmall-Group Session 3 Facilitator Guide.docx [file mep_2374-8265.11366-s001.zip › C. Large-Group Session 1.pptx]

## Slide 1
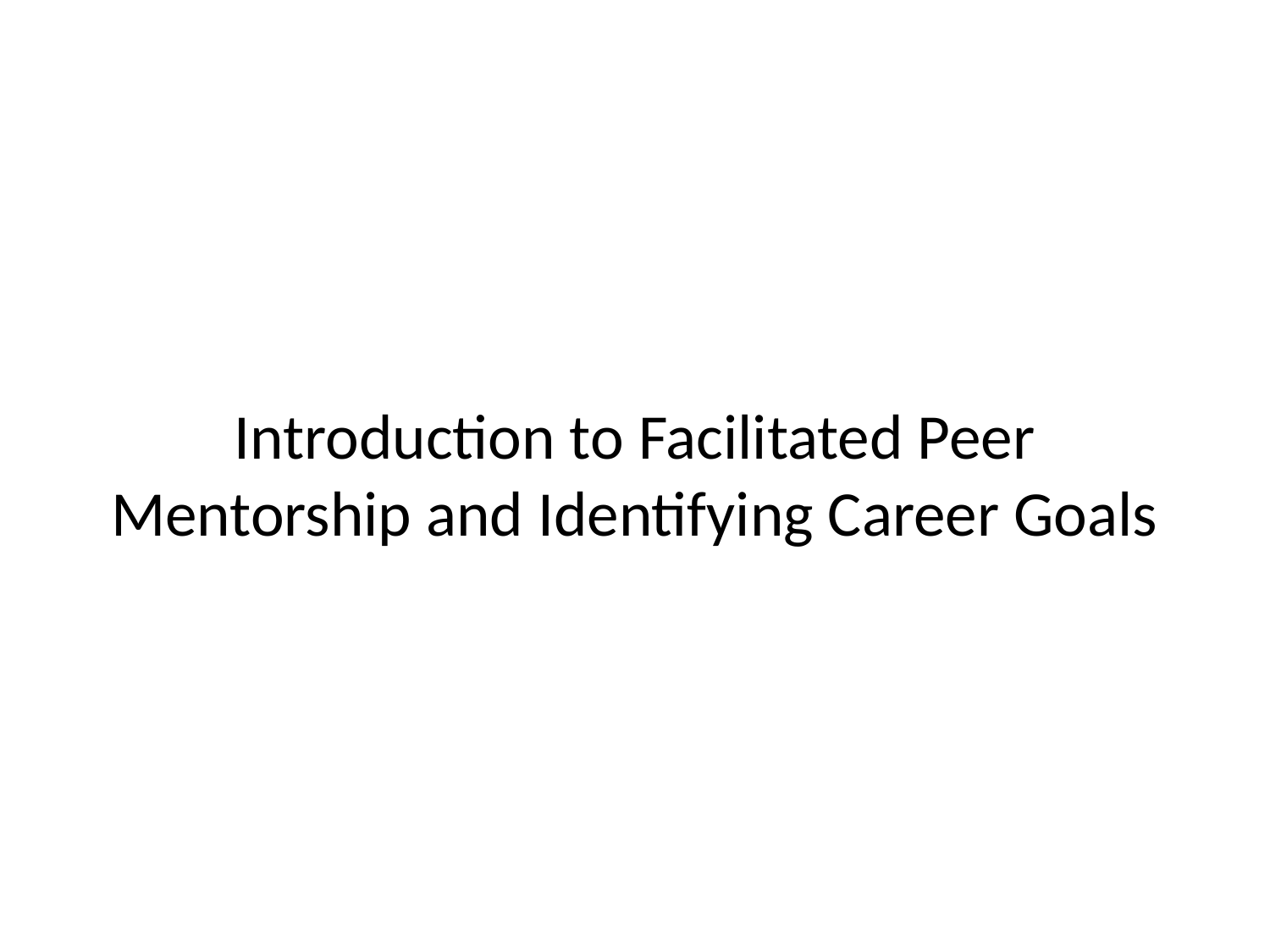

# Introduction to Facilitated Peer Mentorship and Identifying Career Goals

## Slide 2
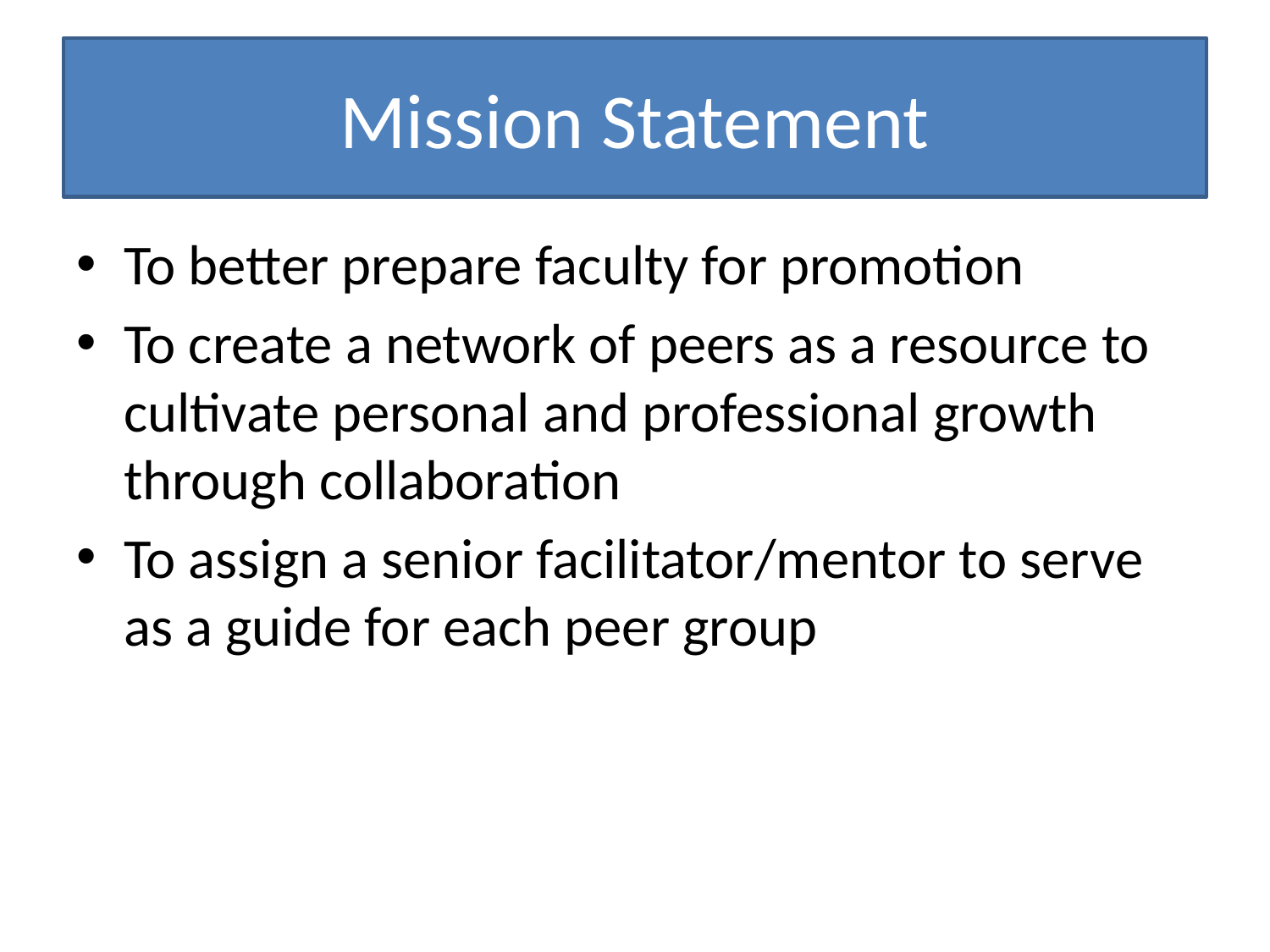

# Mission Statement
To better prepare faculty for promotion
To create a network of peers as a resource to cultivate personal and professional growth through collaboration
To assign a senior facilitator/mentor to serve as a guide for each peer group

## Slide 3
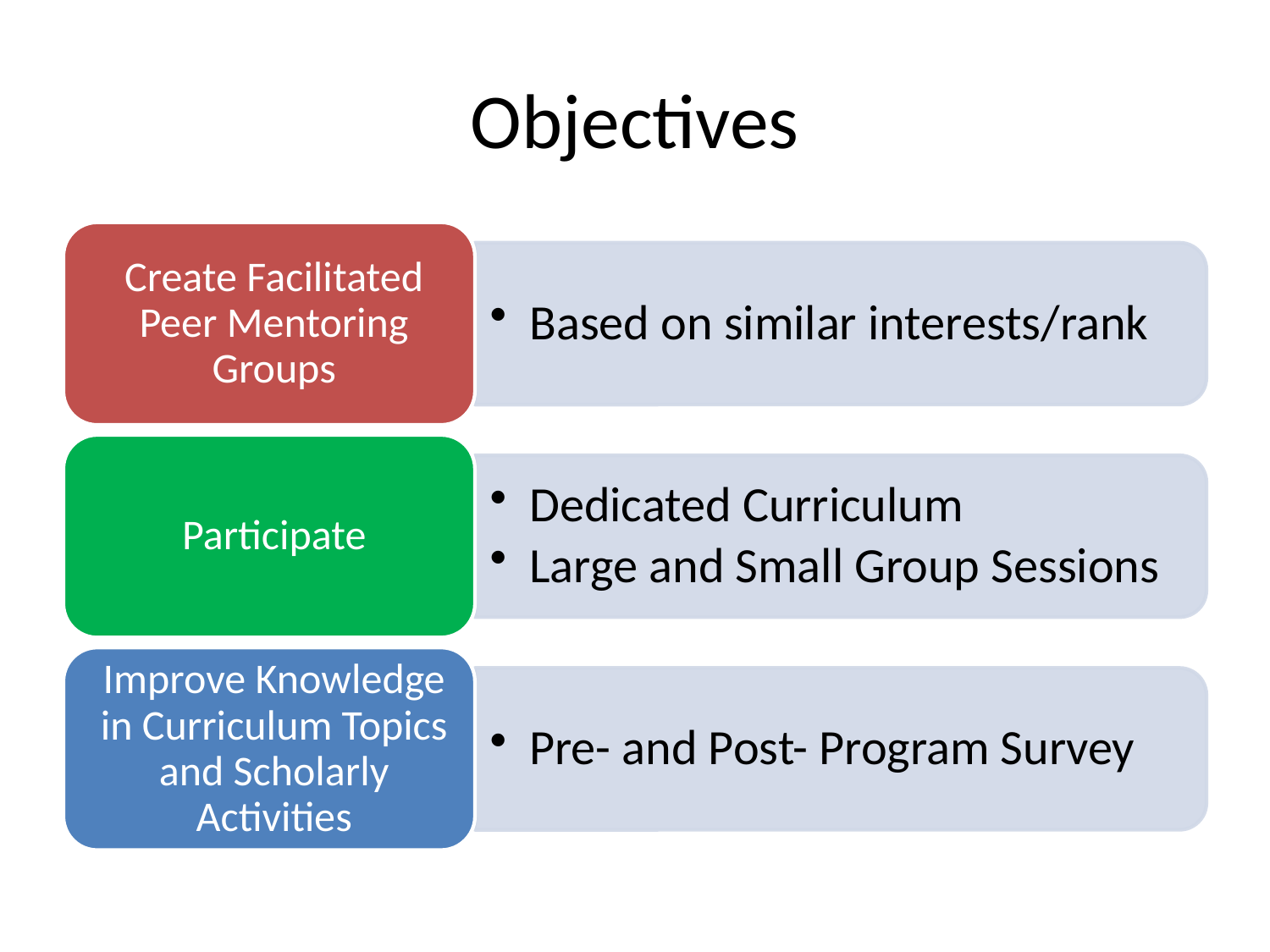

# Objectives

## Slide 4
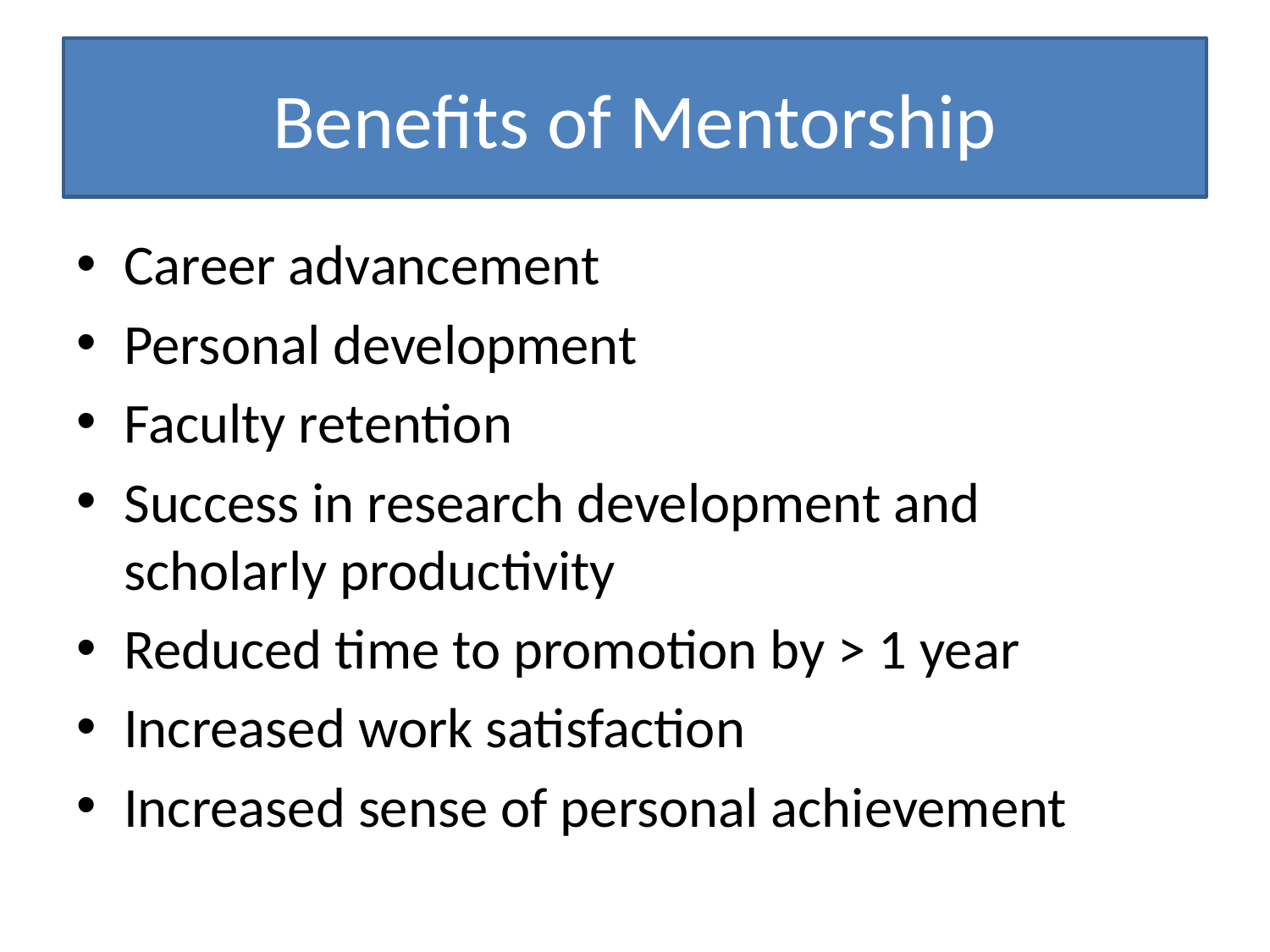

# Benefits of Mentorship
Career advancement
Personal development
Faculty retention
Success in research development and scholarly productivity
Reduced time to promotion by > 1 year
Increased work satisfaction
Increased sense of personal achievement

## Slide 5
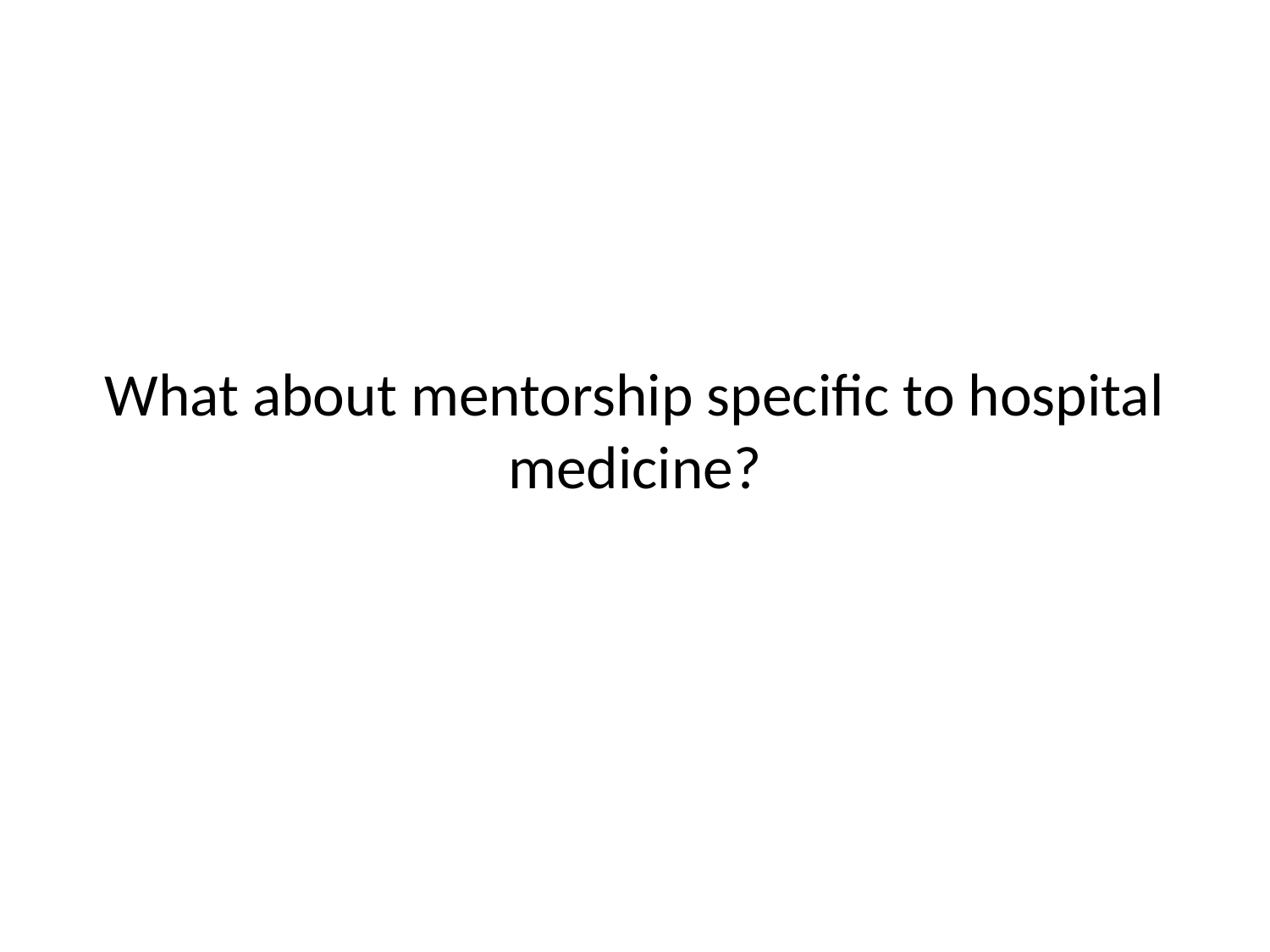

# What about mentorship specific to hospital medicine?

## Slide 6
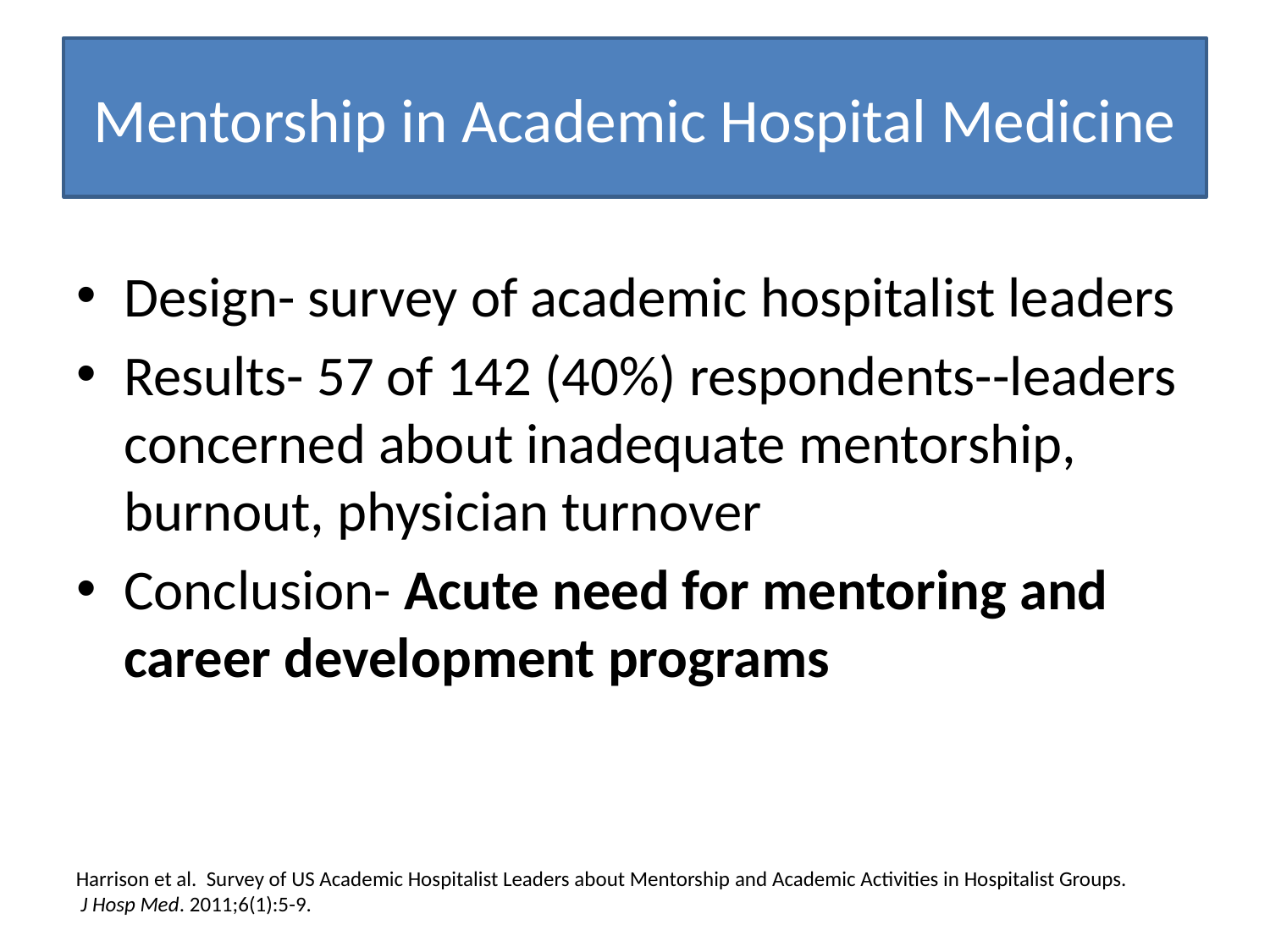

# Mentorship in Academic Hospital Medicine
Design- survey of academic hospitalist leaders
Results- 57 of 142 (40%) respondents--leaders concerned about inadequate mentorship, burnout, physician turnover
Conclusion- Acute need for mentoring and career development programs
Harrison et al. Survey of US Academic Hospitalist Leaders about Mentorship and Academic Activities in Hospitalist Groups.
 J Hosp Med. 2011;6(1):5-9.

## Slide 7
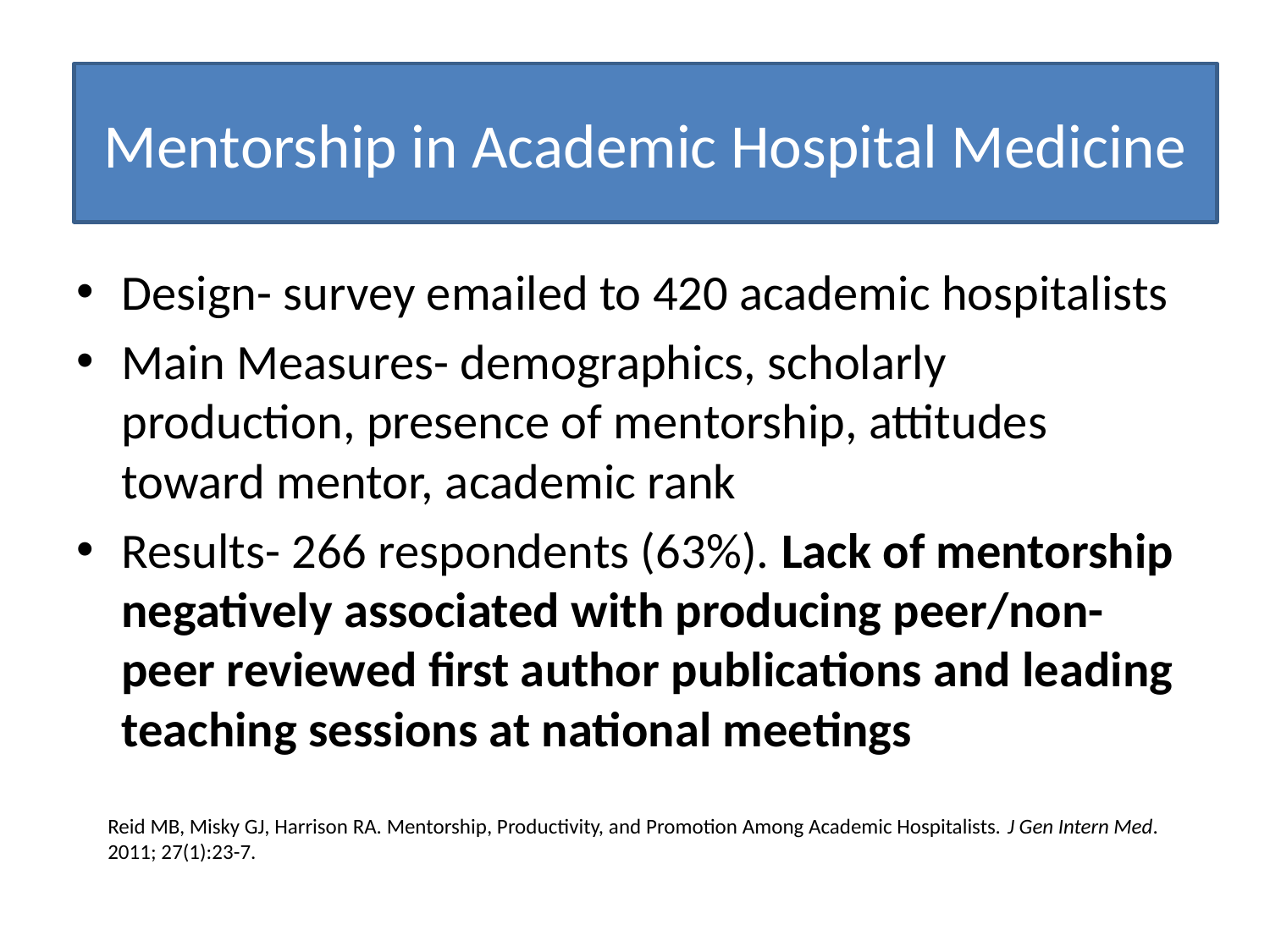

# Mentorship in Academic Hospital Medicine
Design- survey emailed to 420 academic hospitalists
Main Measures- demographics, scholarly production, presence of mentorship, attitudes toward mentor, academic rank
Results- 266 respondents (63%). Lack of mentorship negatively associated with producing peer/non-peer reviewed first author publications and leading teaching sessions at national meetings
Reid MB, Misky GJ, Harrison RA. Mentorship, Productivity, and Promotion Among Academic Hospitalists. J Gen Intern Med. 2011; 27(1):23-7.

## Slide 8
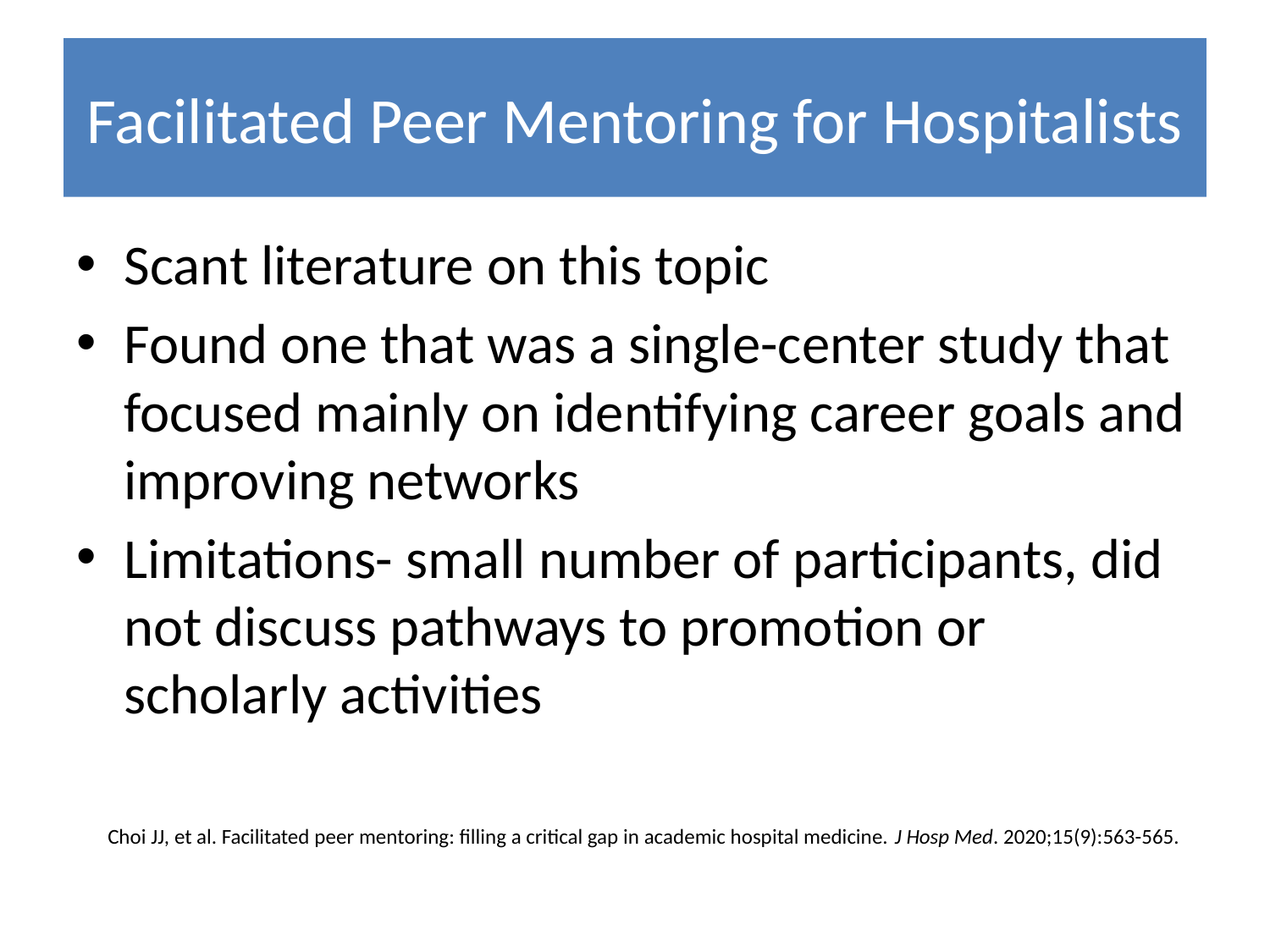

# Facilitated Peer Mentoring for Hospitalists
Scant literature on this topic
Found one that was a single-center study that focused mainly on identifying career goals and improving networks
Limitations- small number of participants, did not discuss pathways to promotion or scholarly activities
Choi JJ, et al. Facilitated peer mentoring: filling a critical gap in academic hospital medicine. J Hosp Med. 2020;15(9):563-565.

## Slide 9
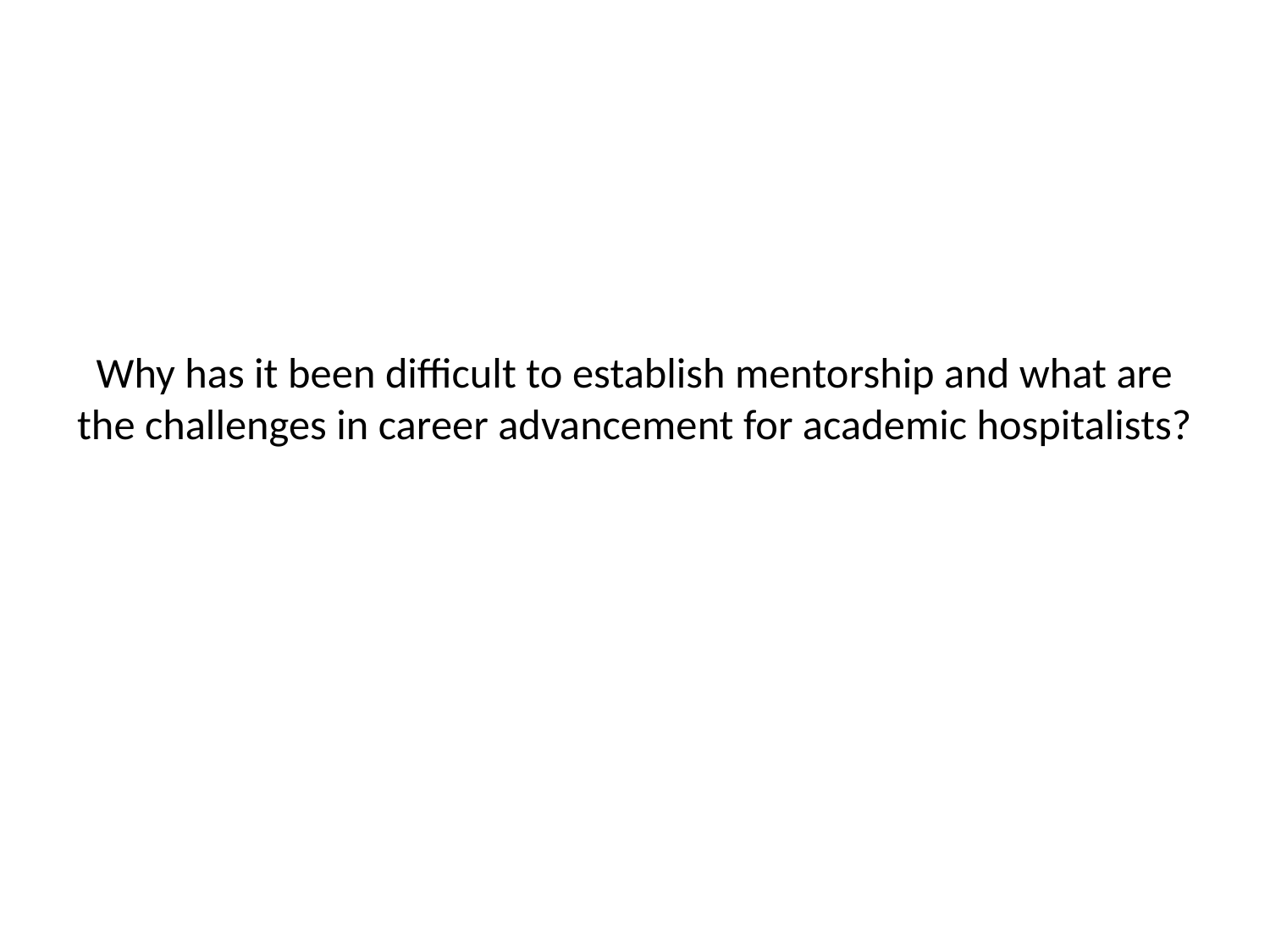

# Why has it been difficult to establish mentorship and what are the challenges in career advancement for academic hospitalists?

## Slide 10
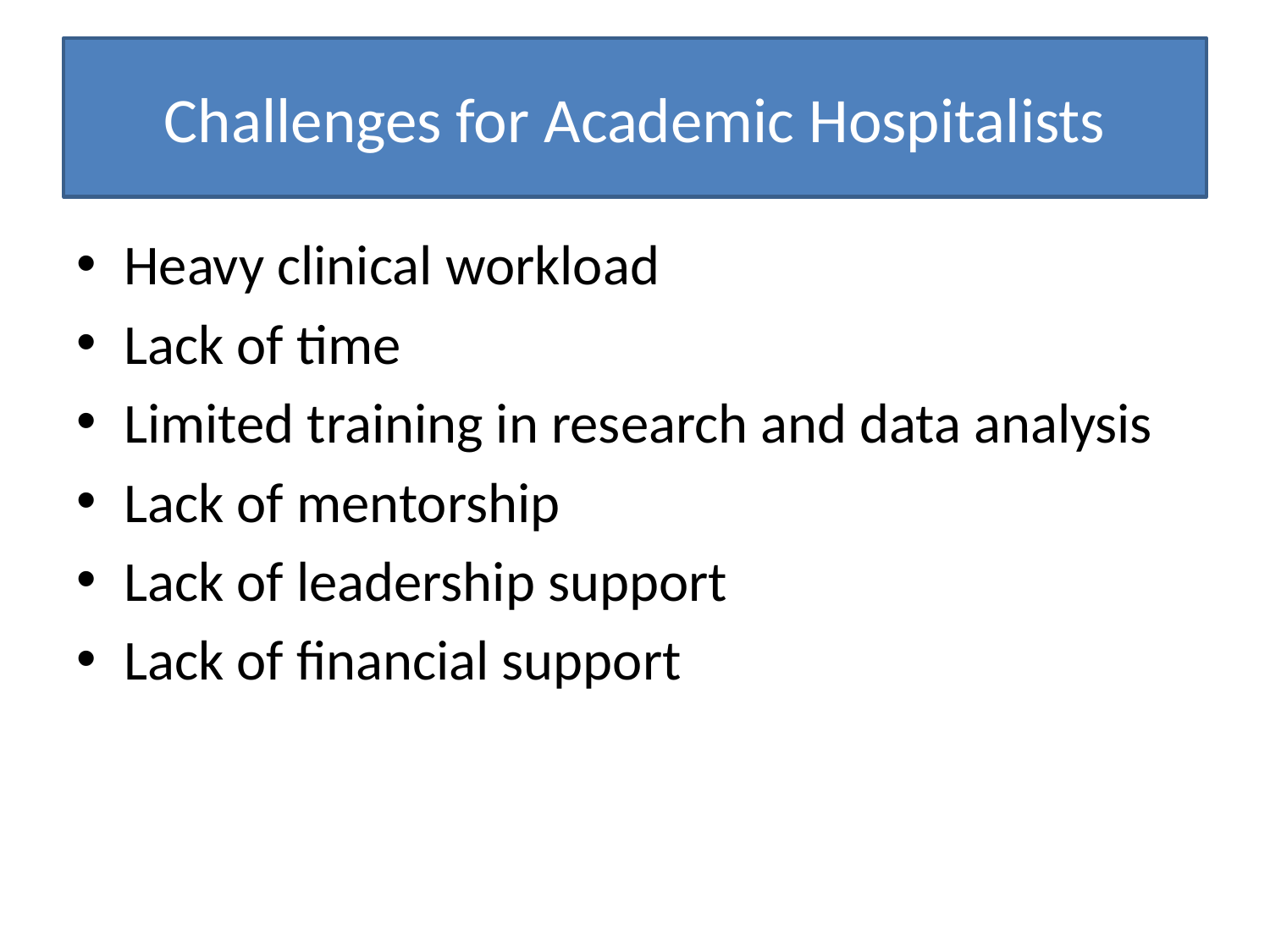

# Challenges for Academic Hospitalists
Heavy clinical workload
Lack of time
Limited training in research and data analysis
Lack of mentorship
Lack of leadership support
Lack of financial support

## Slide 11
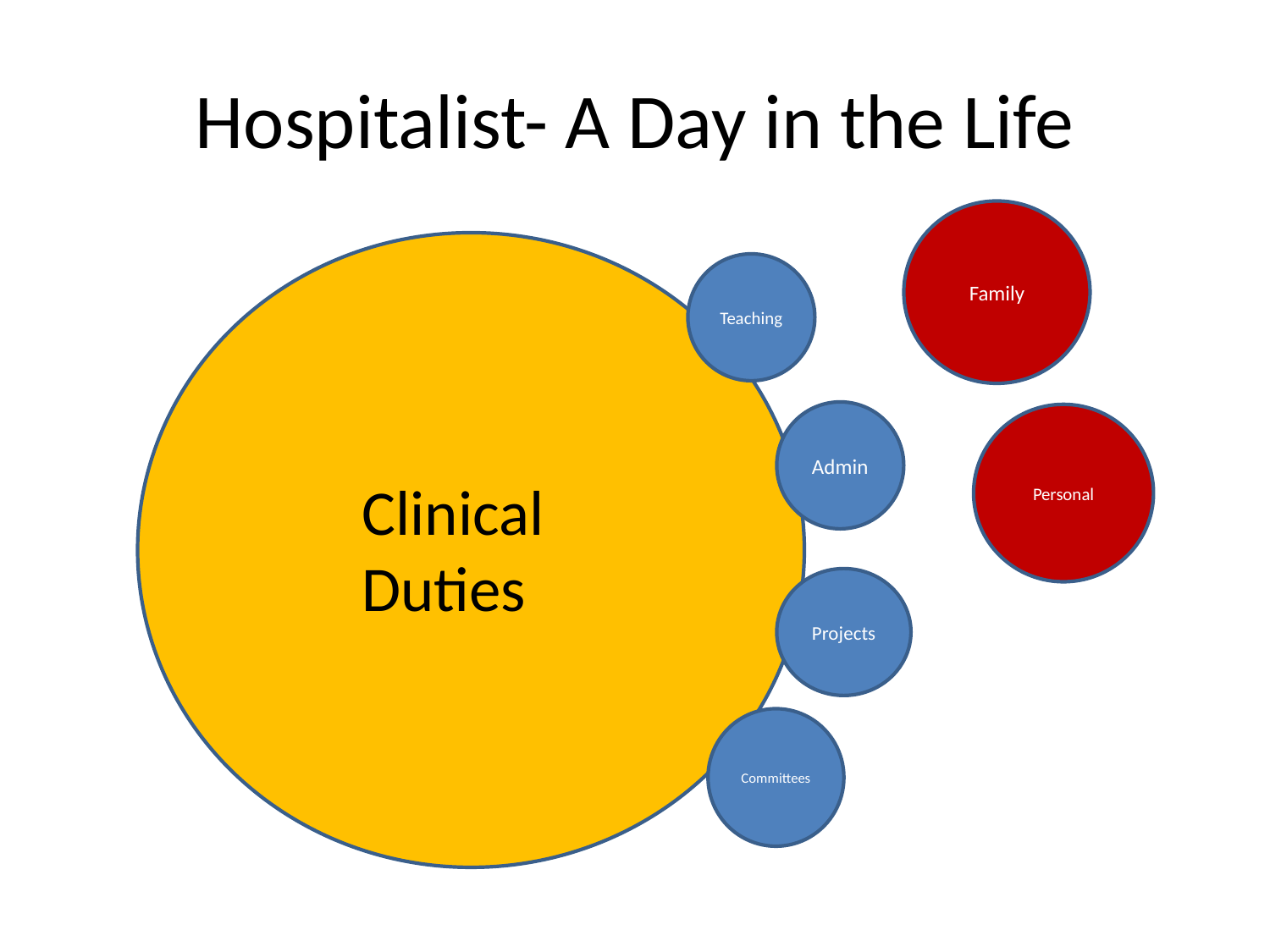

# Hospitalist- A Day in the Life
Family
Teaching
Admin
Personal
Clinical Duties
Projects
Committees

## Slide 12
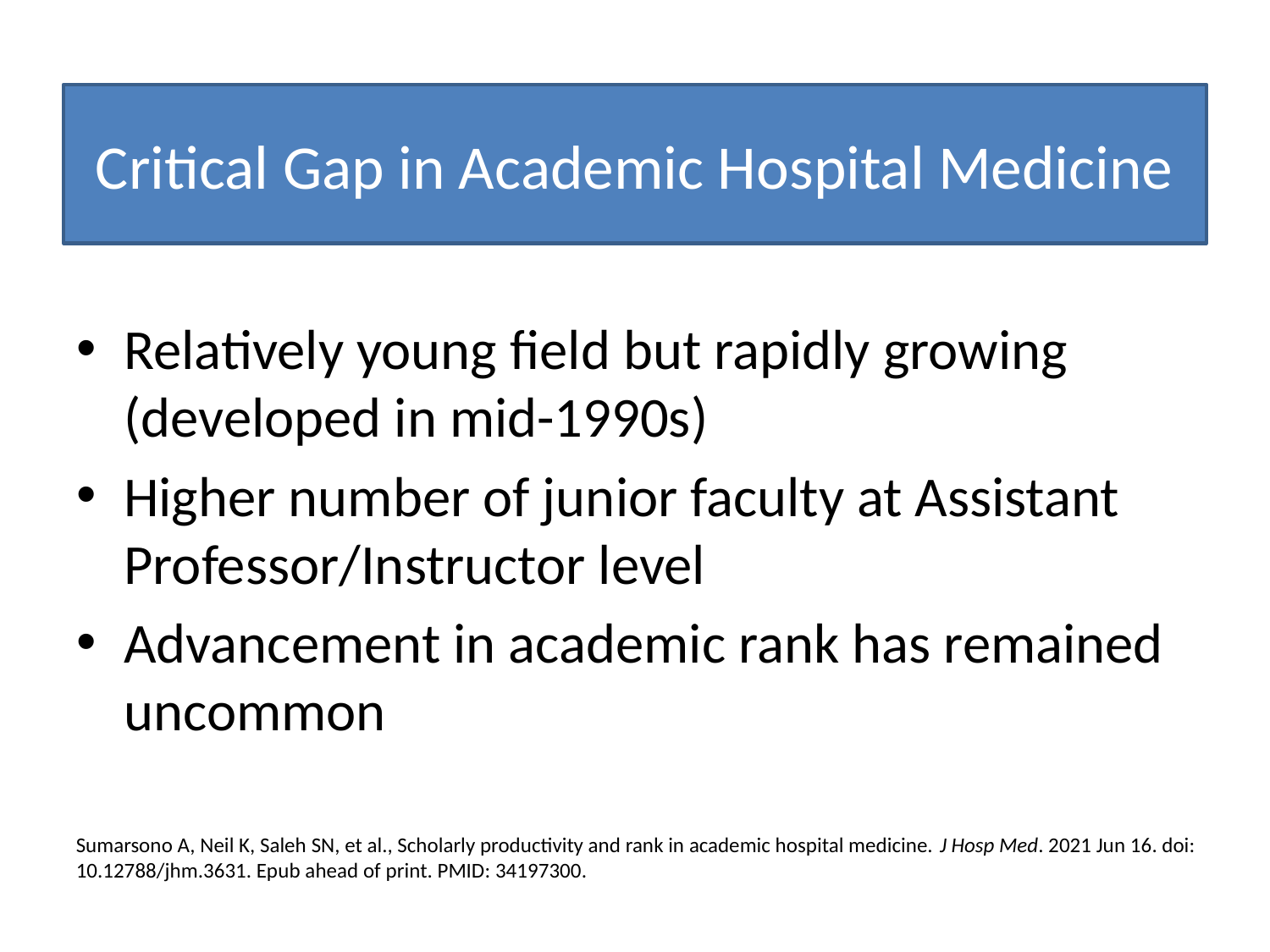

# Critical Gap in Academic Hospital Medicine
Relatively young field but rapidly growing (developed in mid-1990s)
Higher number of junior faculty at Assistant Professor/Instructor level
Advancement in academic rank has remained uncommon
Sumarsono A, Neil K, Saleh SN, et al., Scholarly productivity and rank in academic hospital medicine. J Hosp Med. 2021 Jun 16. doi: 10.12788/jhm.3631. Epub ahead of print. PMID: 34197300.

## Slide 13
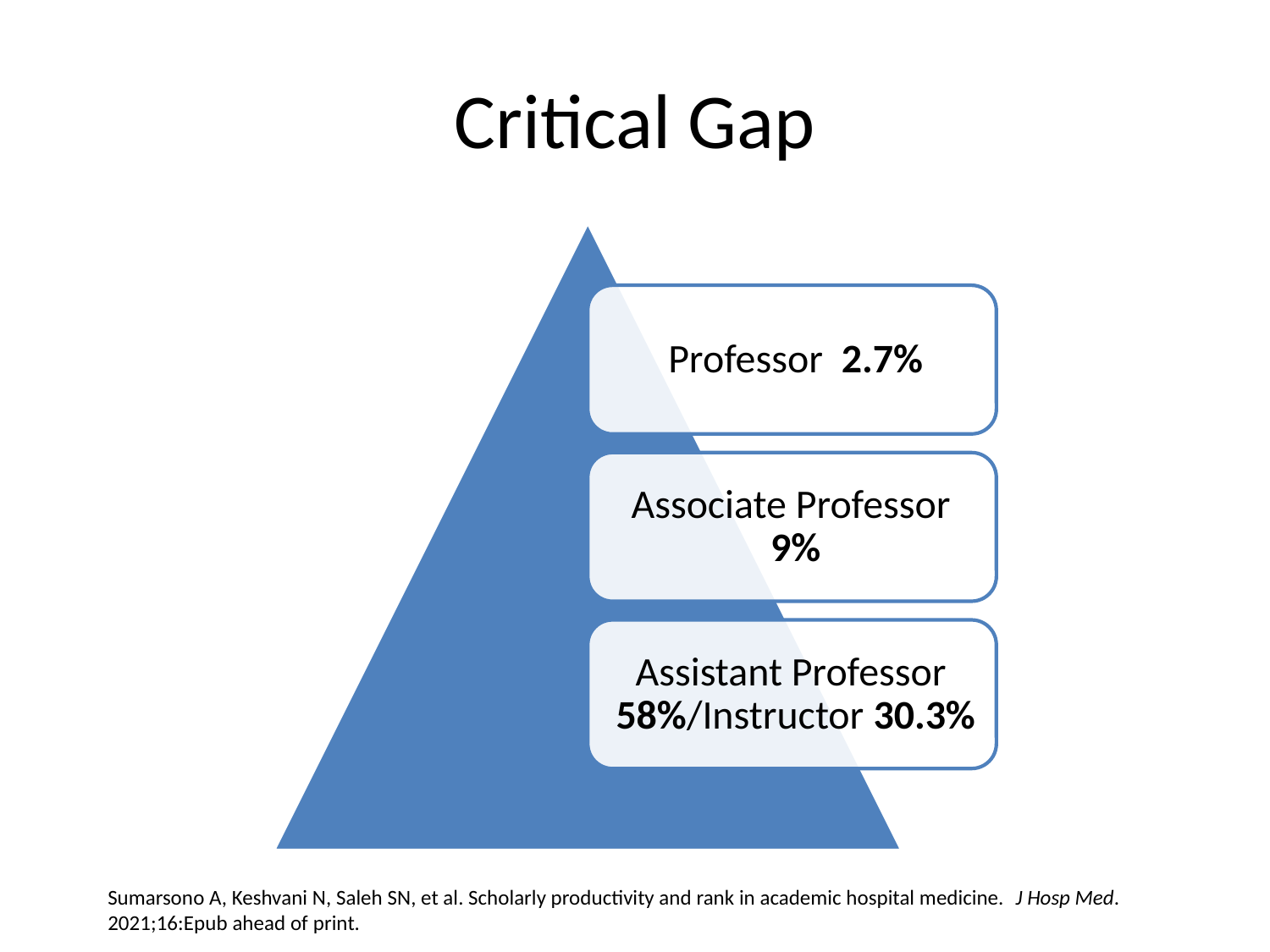

# Critical Gap
Sumarsono A, Keshvani N, Saleh SN, et al. Scholarly productivity and rank in academic hospital medicine. J Hosp Med. 2021;16:Epub ahead of print.

## Slide 14
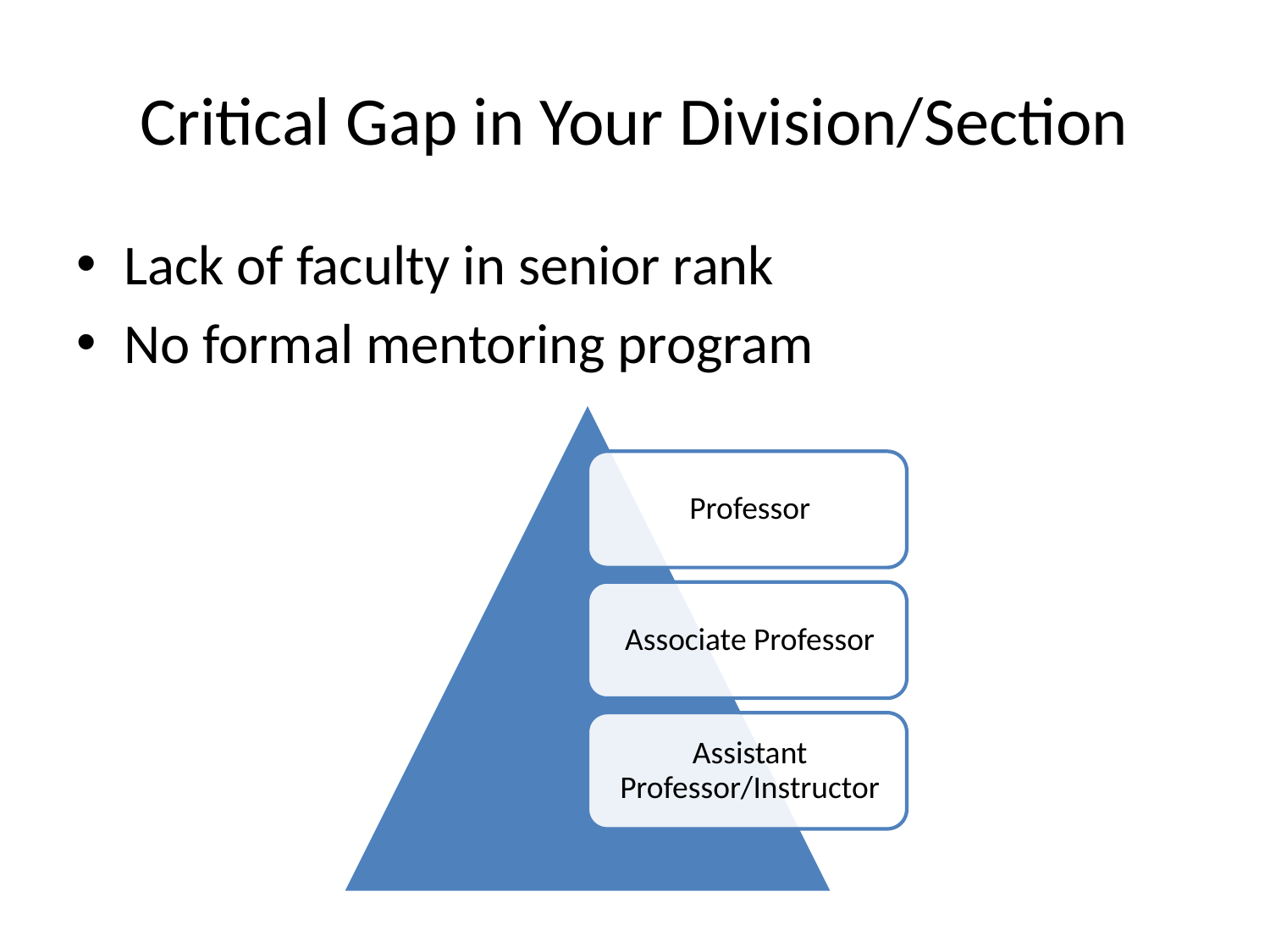

# Critical Gap in Your Division/Section
Lack of faculty in senior rank
No formal mentoring program

## Slide 15
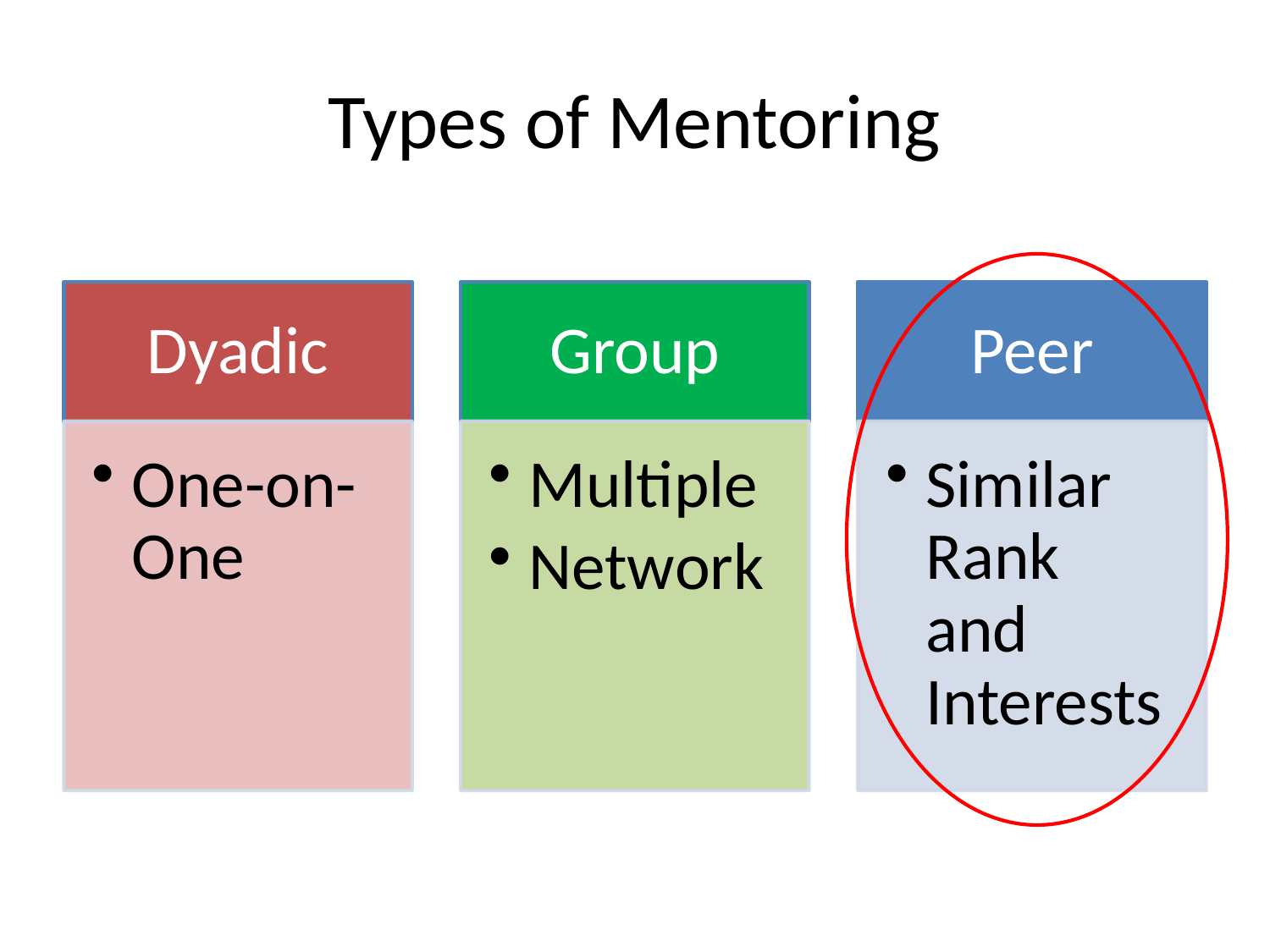

# Types of Mentoring

## Slide 16
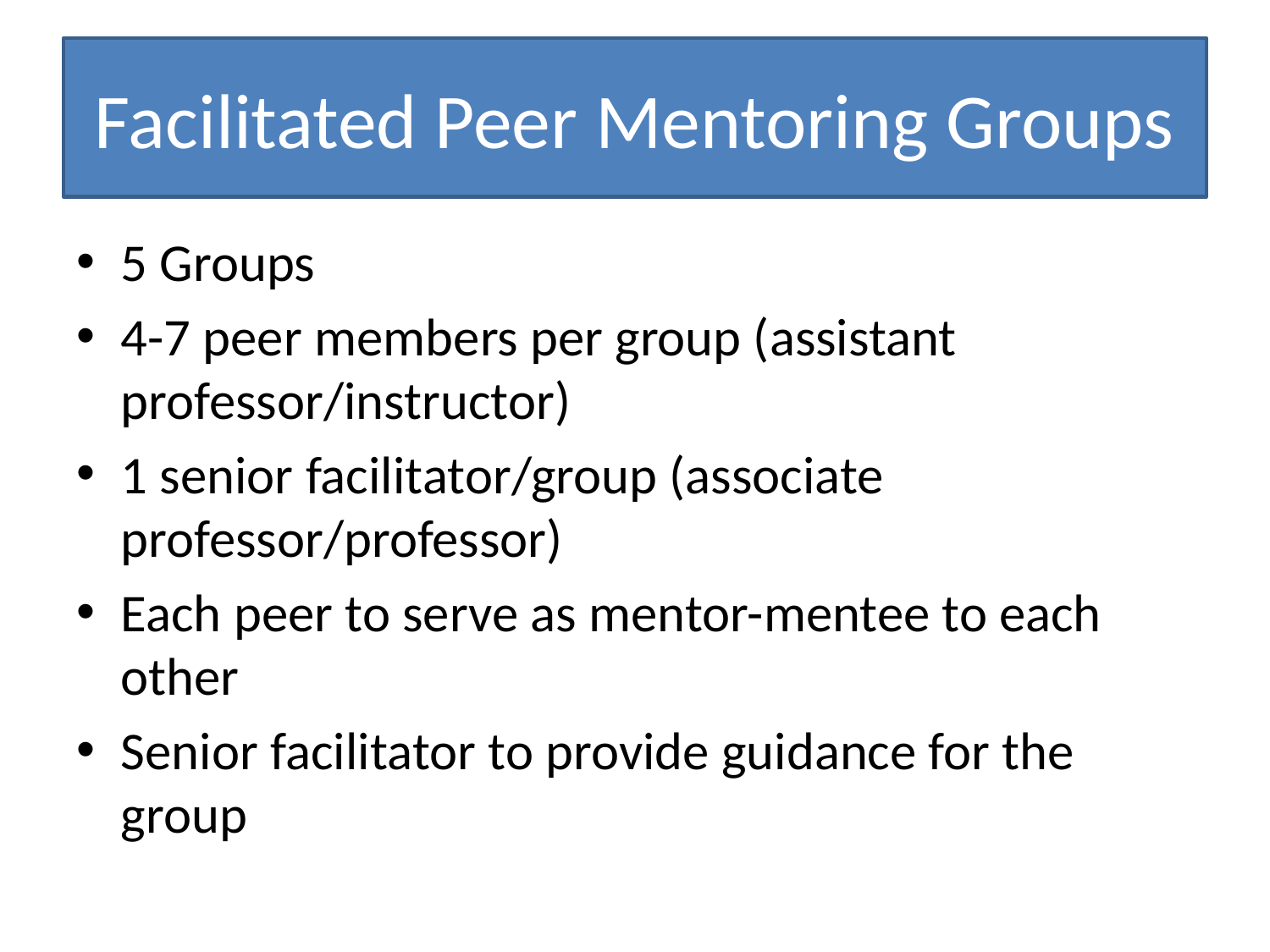

# Facilitated Peer Mentoring Groups
5 Groups
4-7 peer members per group (assistant professor/instructor)
1 senior facilitator/group (associate professor/professor)
Each peer to serve as mentor-mentee to each other
Senior facilitator to provide guidance for the group

## Slide 17
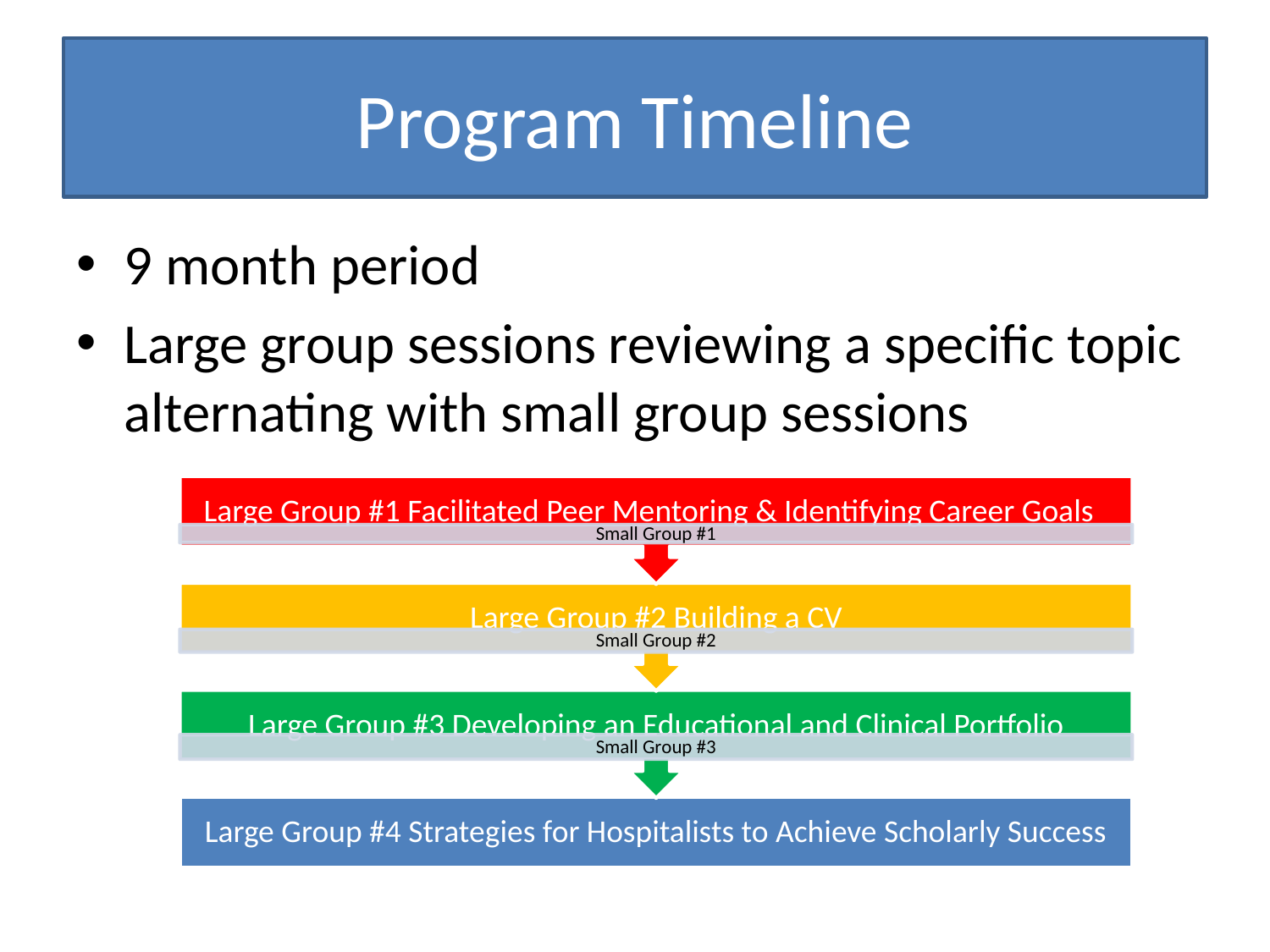

# Program Timeline
9 month period
Large group sessions reviewing a specific topic alternating with small group sessions

## Slide 18
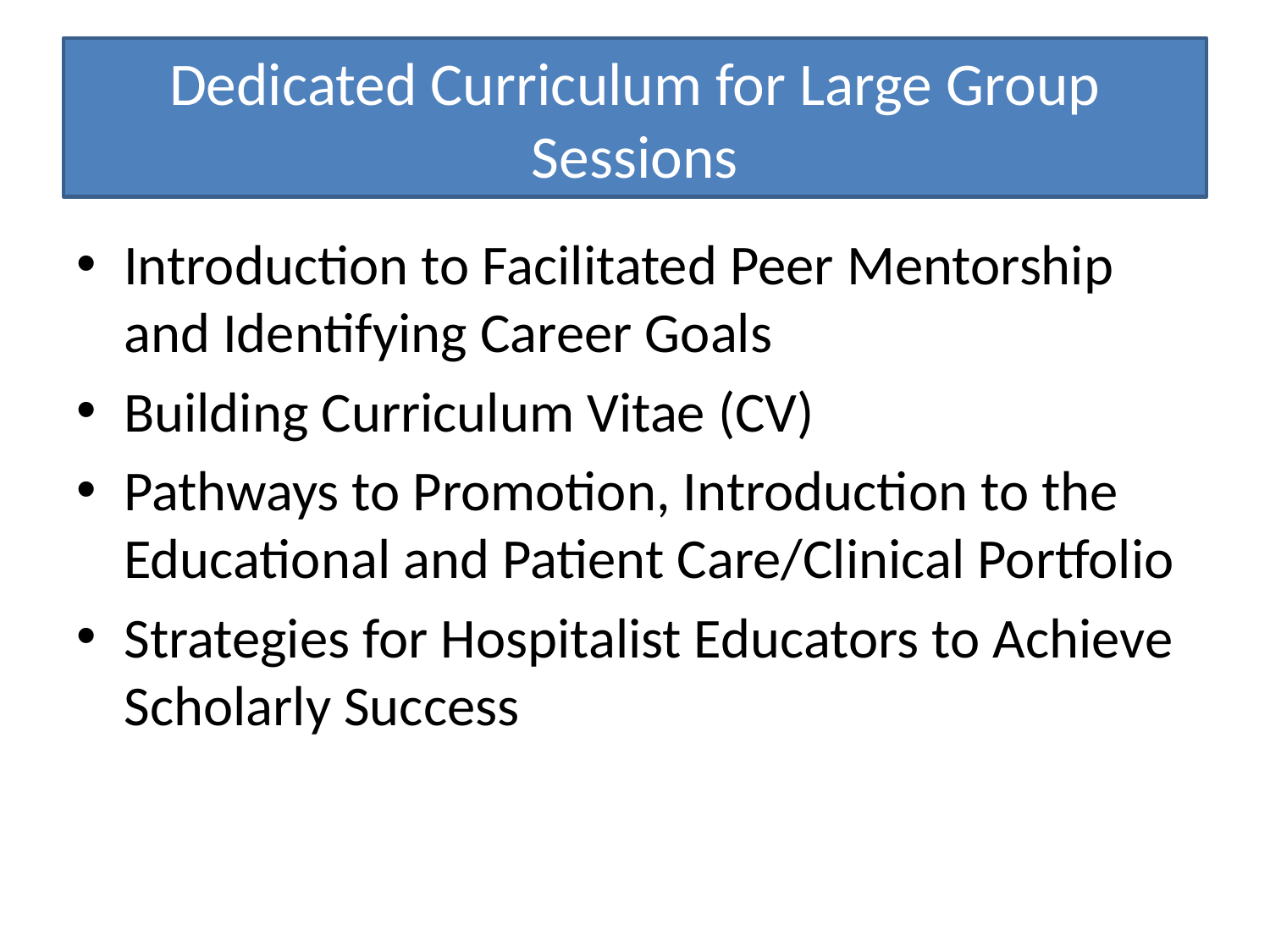

# Dedicated Curriculum for Large Group Sessions
Introduction to Facilitated Peer Mentorship and Identifying Career Goals
Building Curriculum Vitae (CV)
Pathways to Promotion, Introduction to the Educational and Patient Care/Clinical Portfolio
Strategies for Hospitalist Educators to Achieve Scholarly Success

## Slide 19
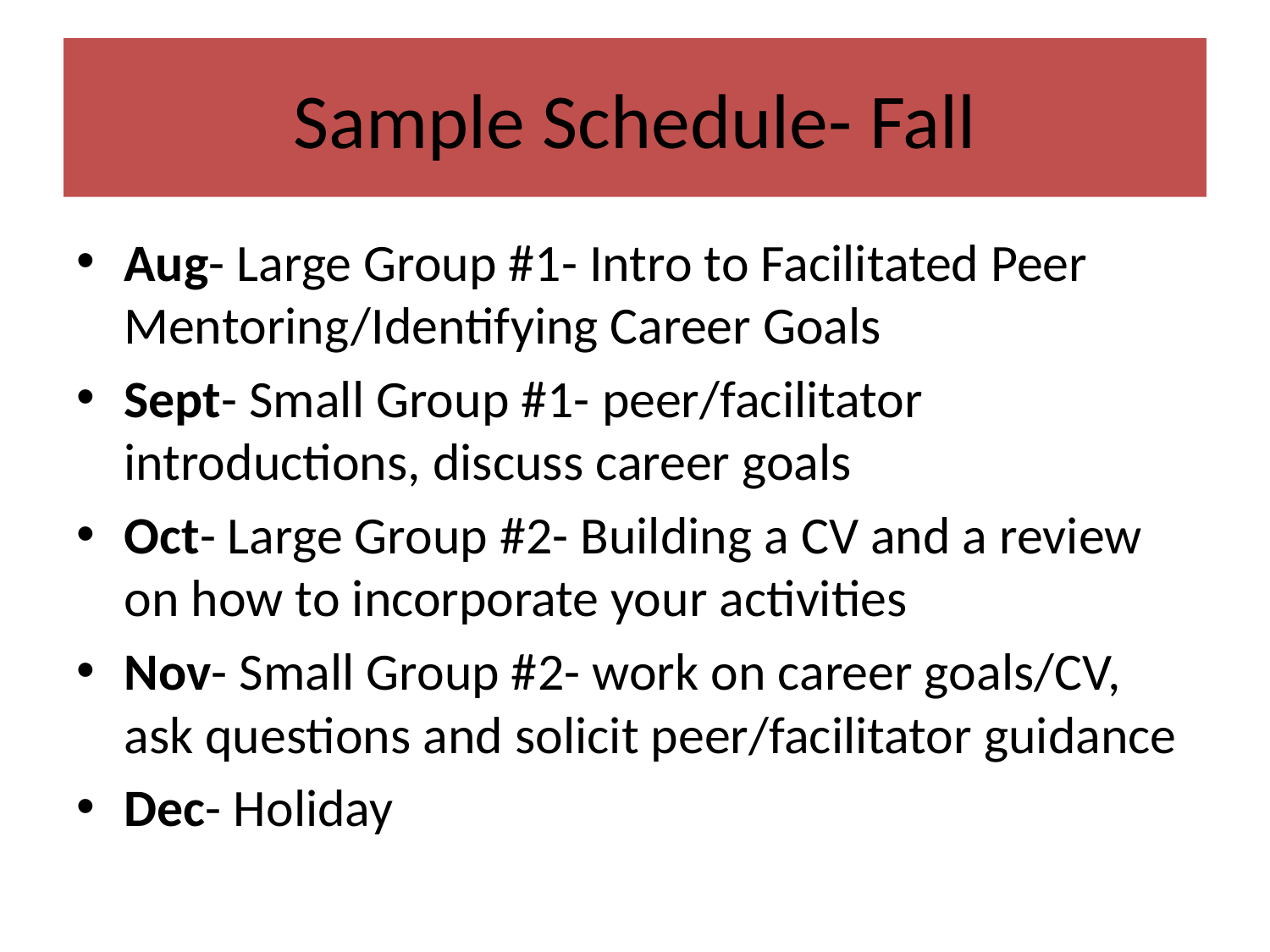

# Sample Schedule- Fall
Aug- Large Group #1- Intro to Facilitated Peer Mentoring/Identifying Career Goals
Sept- Small Group #1- peer/facilitator introductions, discuss career goals
Oct- Large Group #2- Building a CV and a review on how to incorporate your activities
Nov- Small Group #2- work on career goals/CV, ask questions and solicit peer/facilitator guidance
Dec- Holiday

## Slide 20
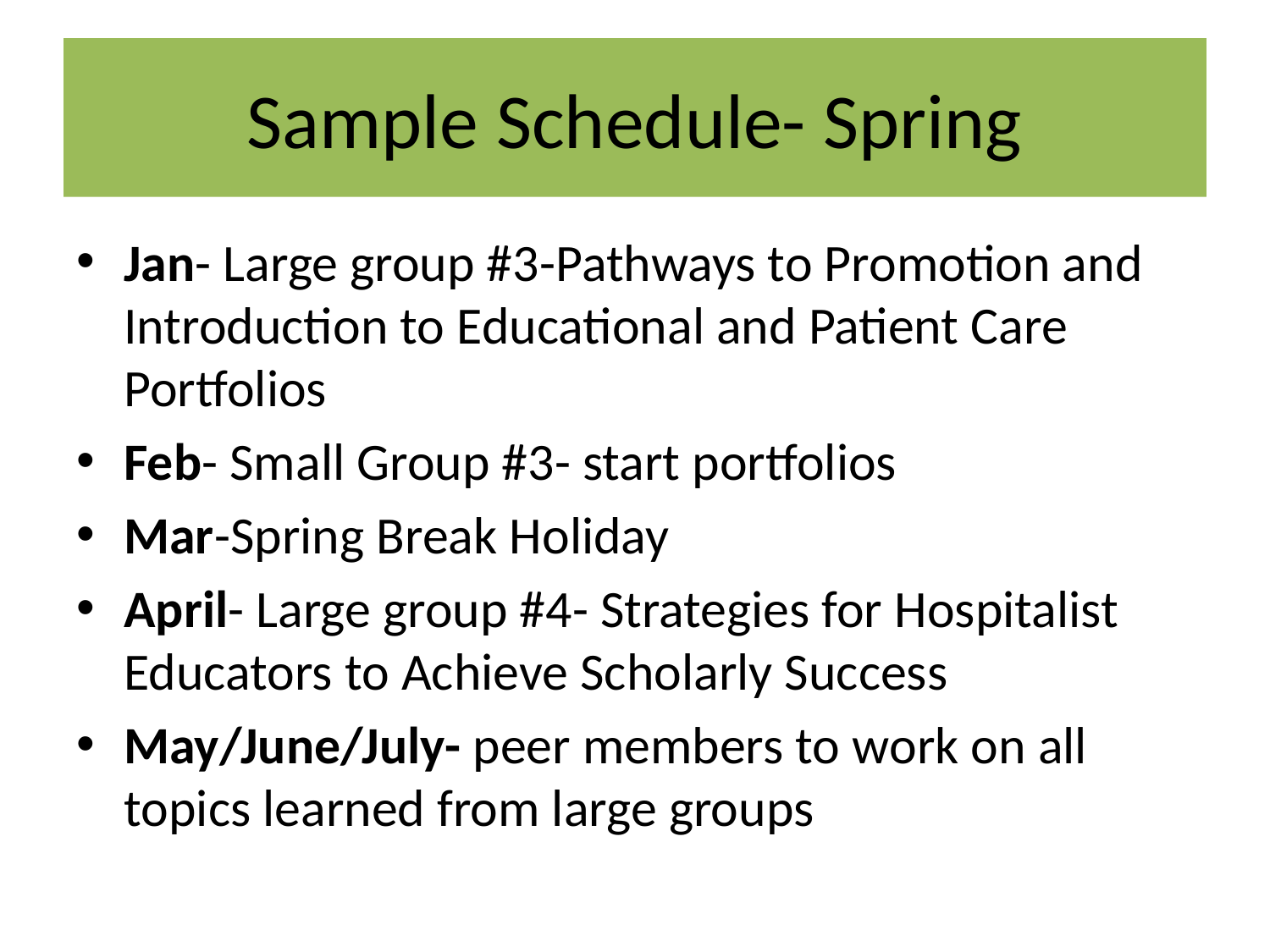

# Sample Schedule- Spring
Jan- Large group #3-Pathways to Promotion and Introduction to Educational and Patient Care Portfolios
Feb- Small Group #3- start portfolios
Mar-Spring Break Holiday
April- Large group #4- Strategies for Hospitalist Educators to Achieve Scholarly Success
May/June/July- peer members to work on all topics learned from large groups

## Slide 21
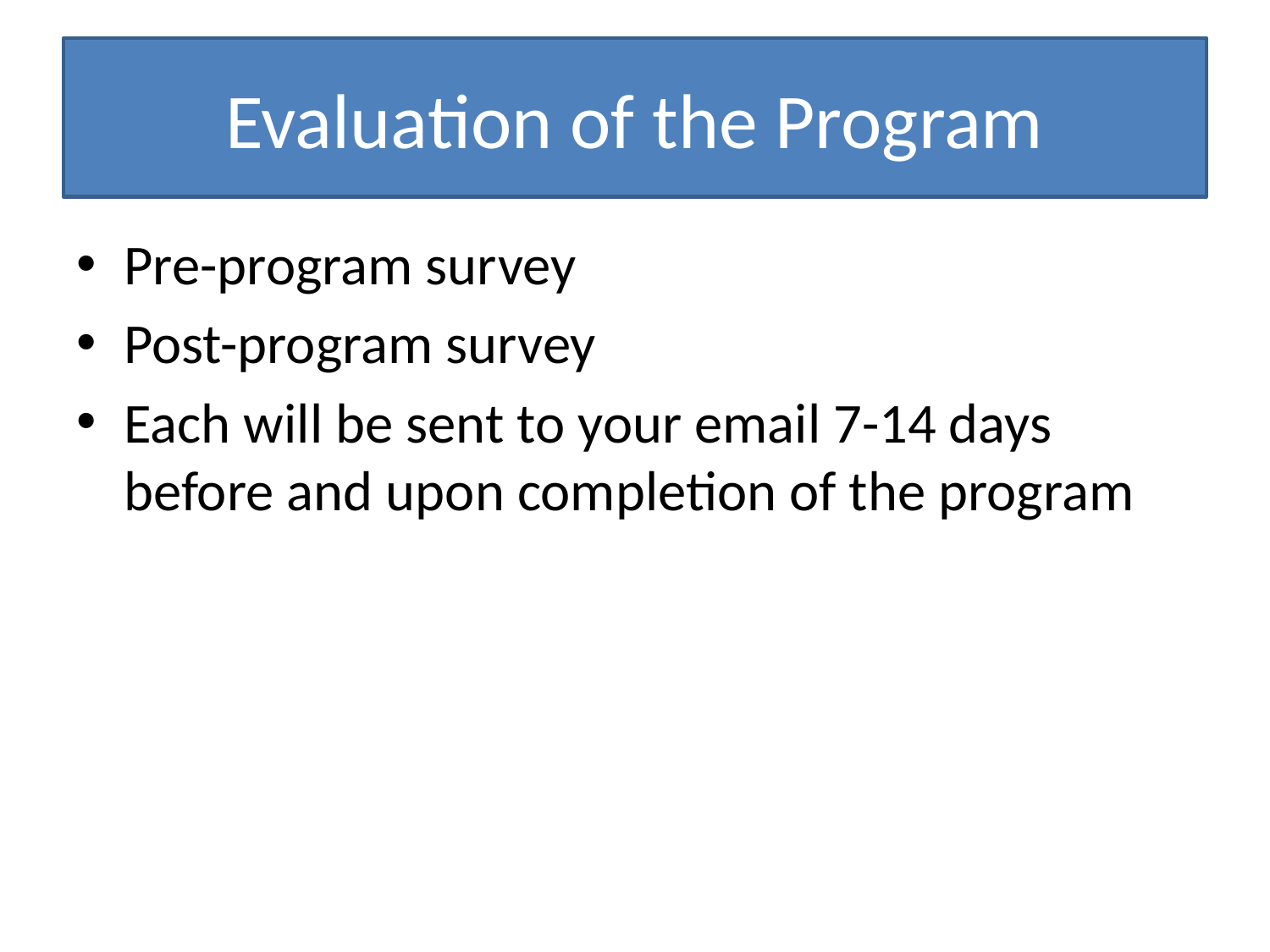

# Evaluation of the Program
Pre-program survey
Post-program survey
Each will be sent to your email 7-14 days before and upon completion of the program

## Slide 22
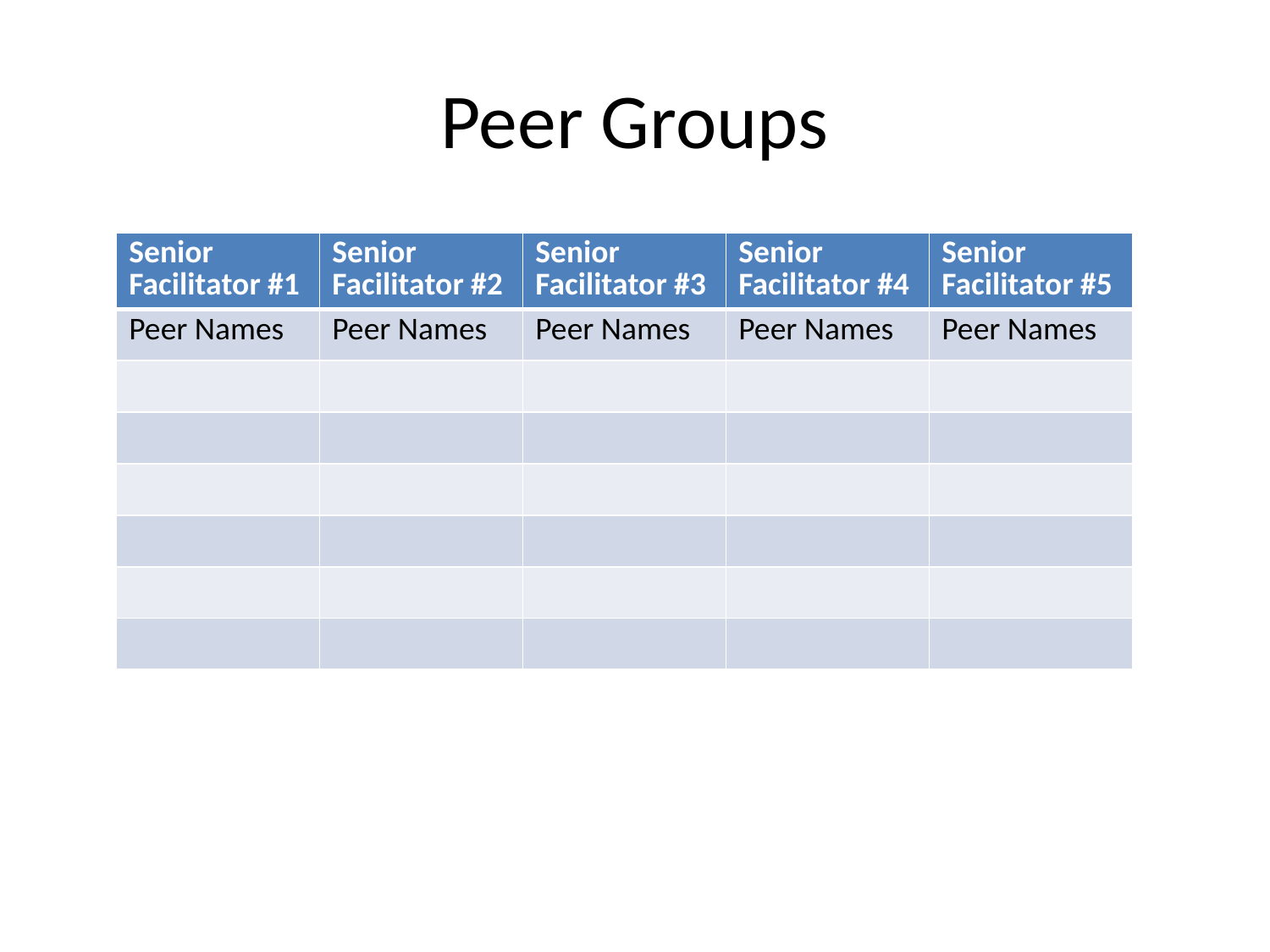

# Peer Groups
| Senior Facilitator #1 | Senior Facilitator #2 | Senior Facilitator #3 | Senior Facilitator #4 | Senior Facilitator #5 |
| --- | --- | --- | --- | --- |
| Peer Names | Peer Names | Peer Names | Peer Names | Peer Names |
| | | | | |
| | | | | |
| | | | | |
| | | | | |
| | | | | |
| | | | | |

## Slide 23
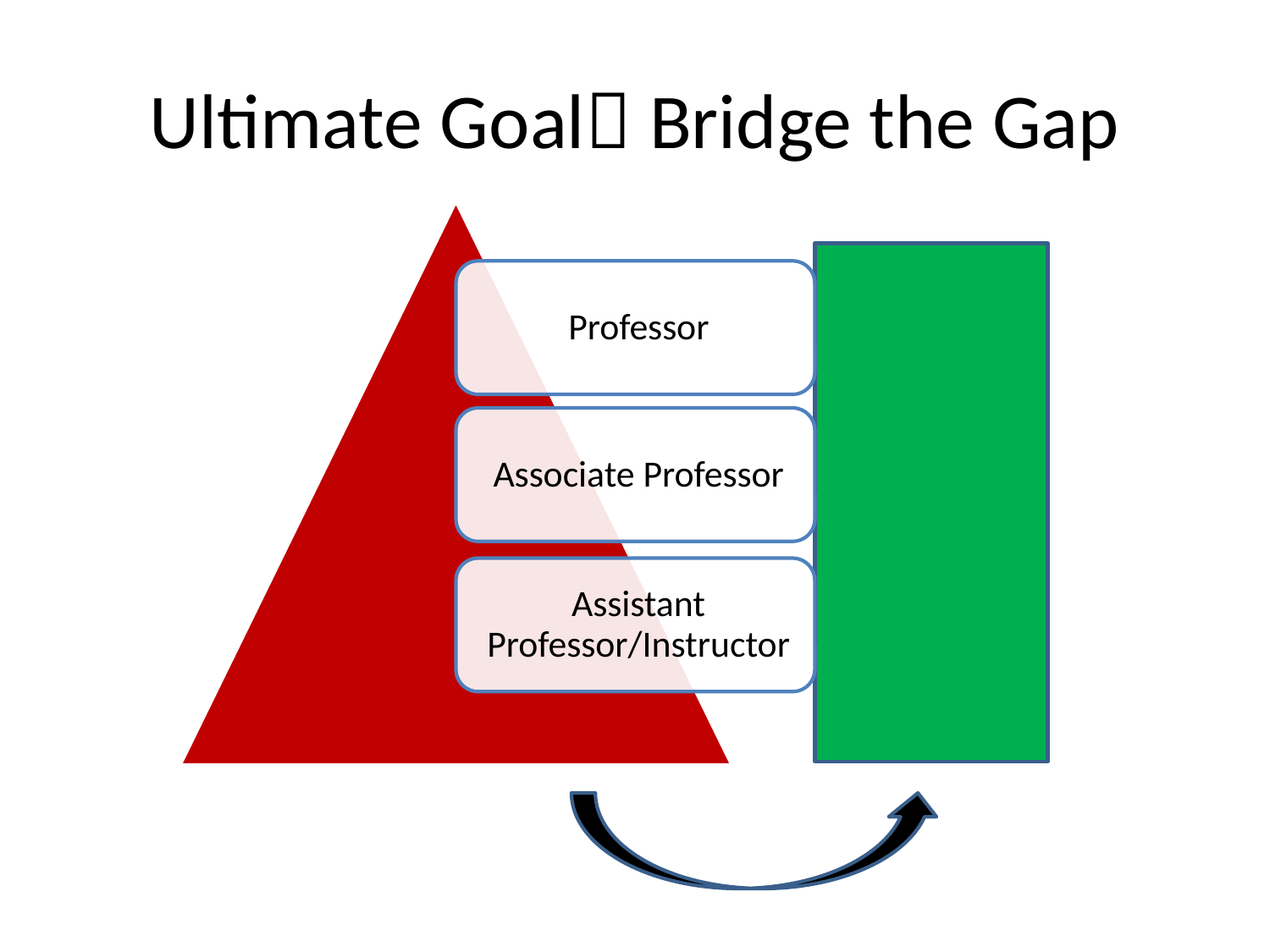

# Ultimate Goal Bridge the Gap

## Slide 24
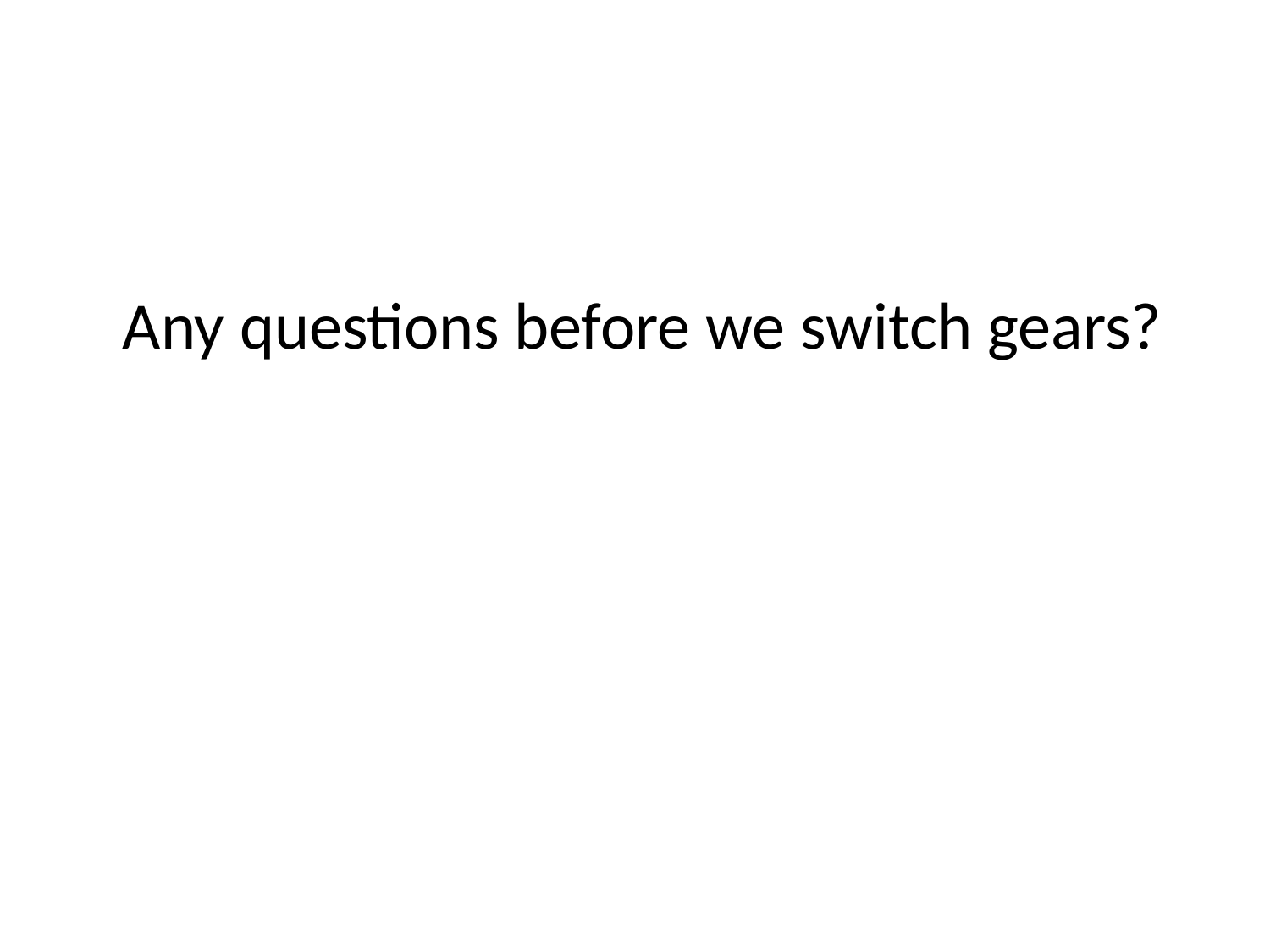

# Any questions before we switch gears?

## Slide 25
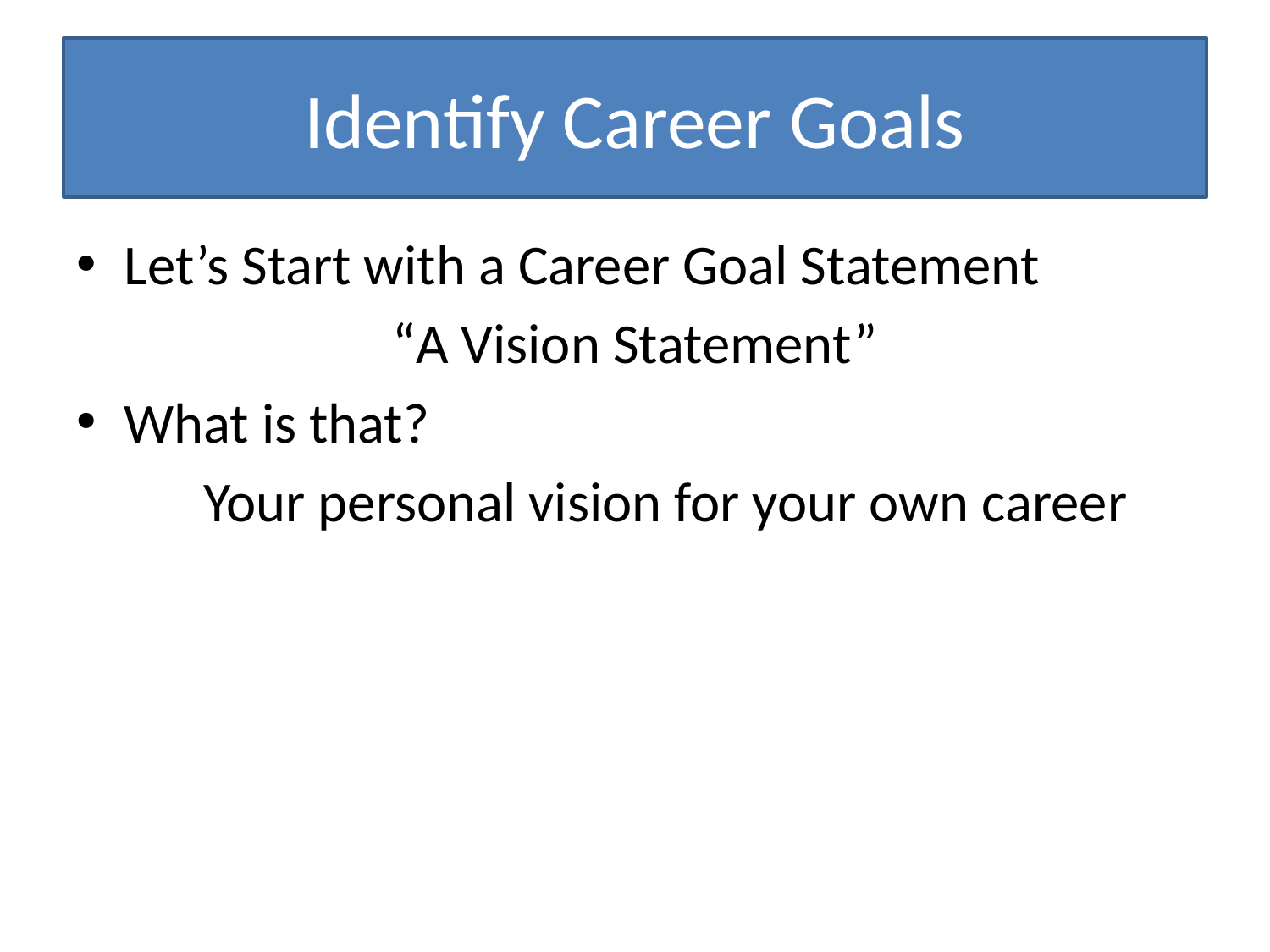

# Identify Career Goals
Let’s Start with a Career Goal Statement
“A Vision Statement”
What is that?
	Your personal vision for your own career

## Slide 26
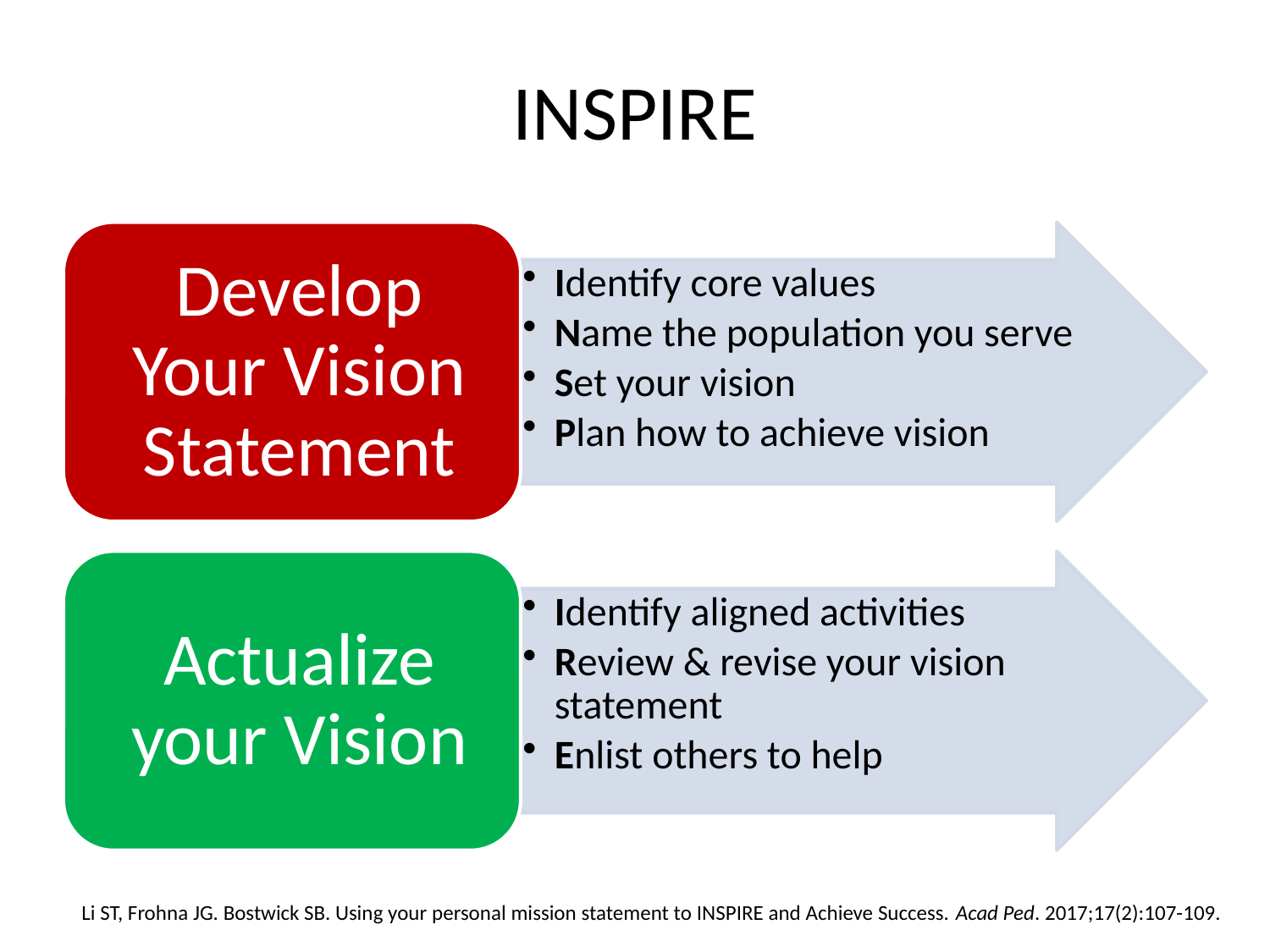

# INSPIRE
Li ST, Frohna JG. Bostwick SB. Using your personal mission statement to INSPIRE and Achieve Success. Acad Ped. 2017;17(2):107-109.

## Slide 27
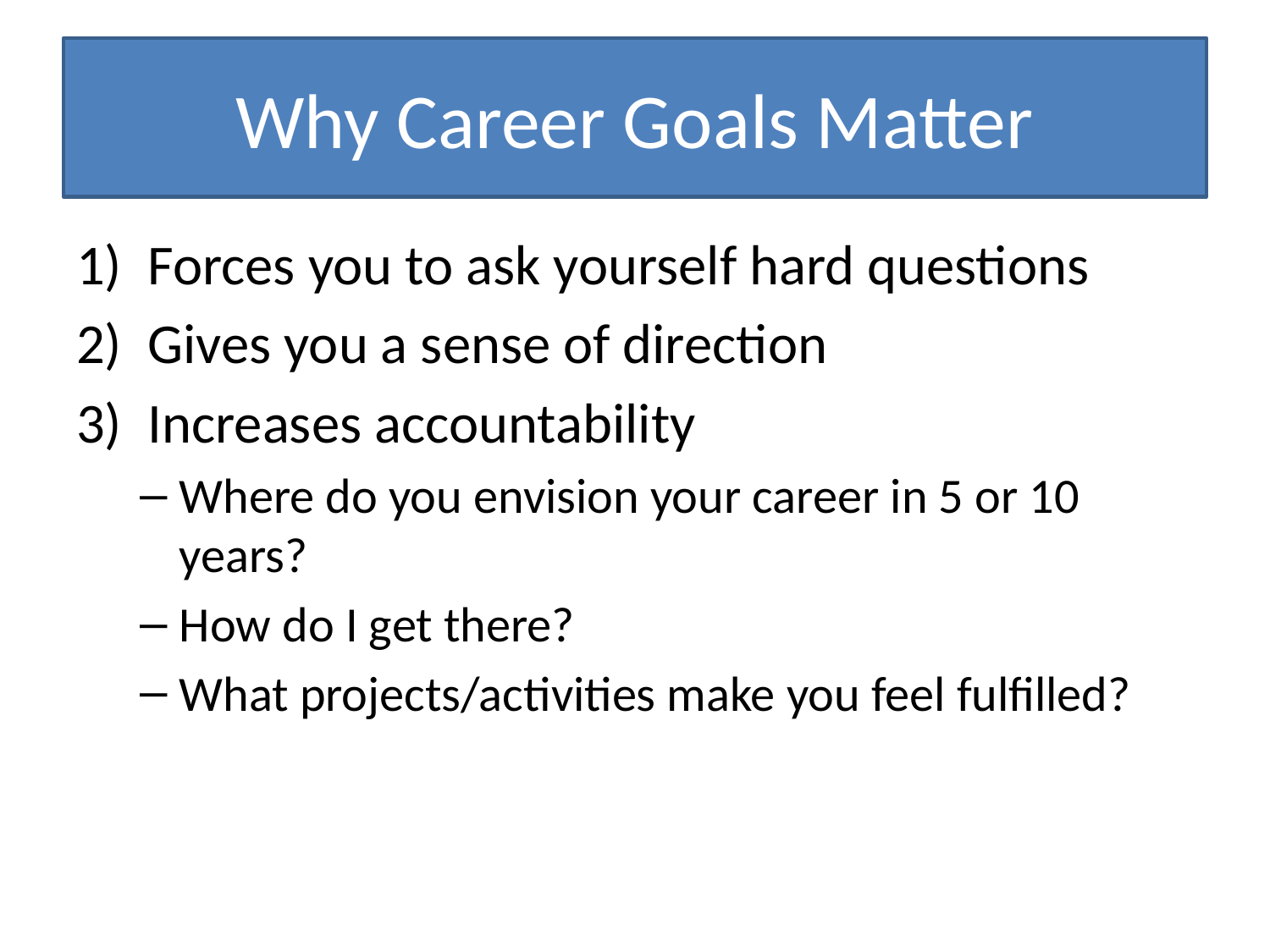

# Why Career Goals Matter
Forces you to ask yourself hard questions
Gives you a sense of direction
Increases accountability
Where do you envision your career in 5 or 10 years?
How do I get there?
What projects/activities make you feel fulfilled?

## Slide 28
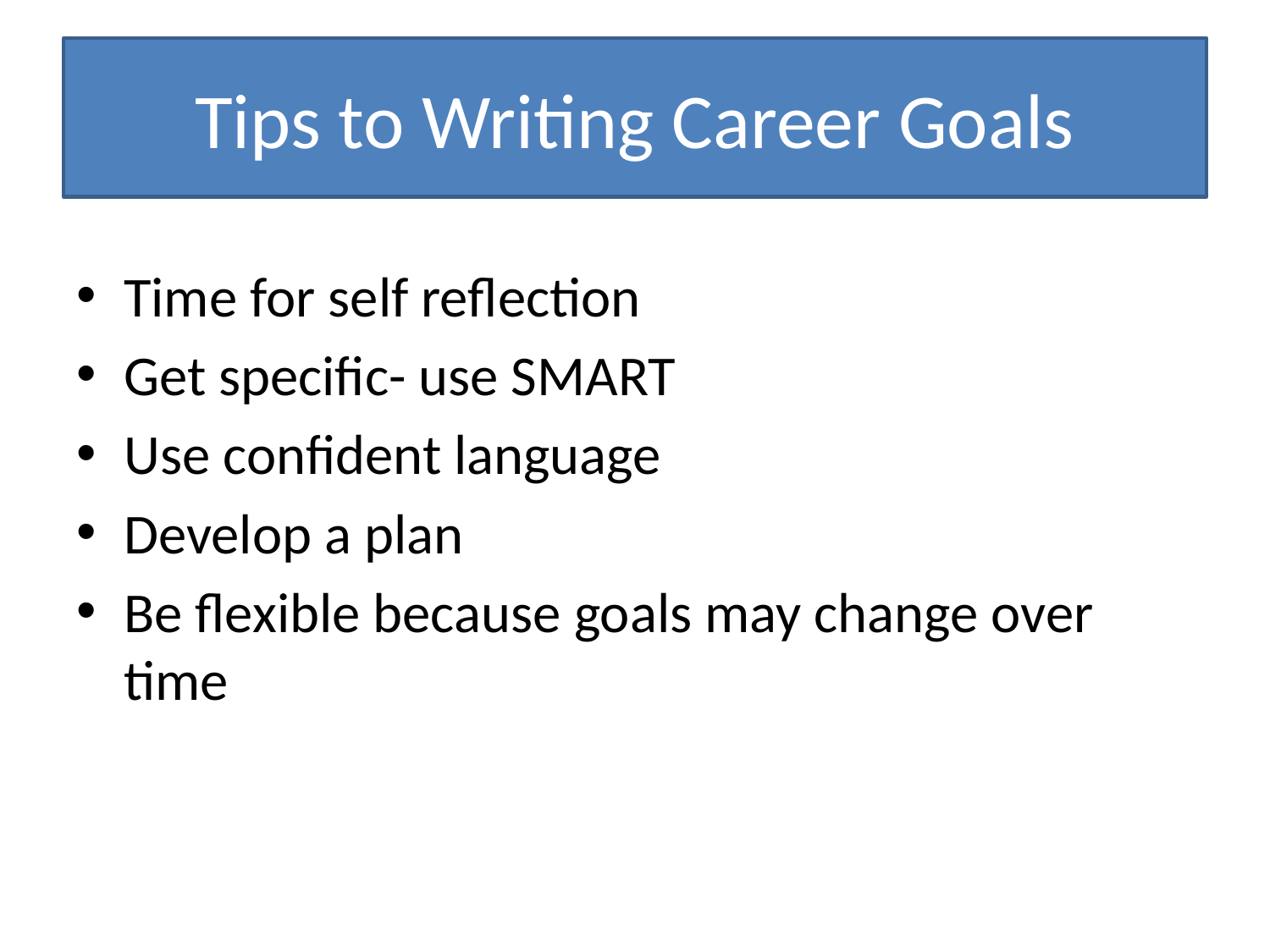

# Tips to Writing Career Goals
Time for self reflection
Get specific- use SMART
Use confident language
Develop a plan
Be flexible because goals may change over time

## Slide 29
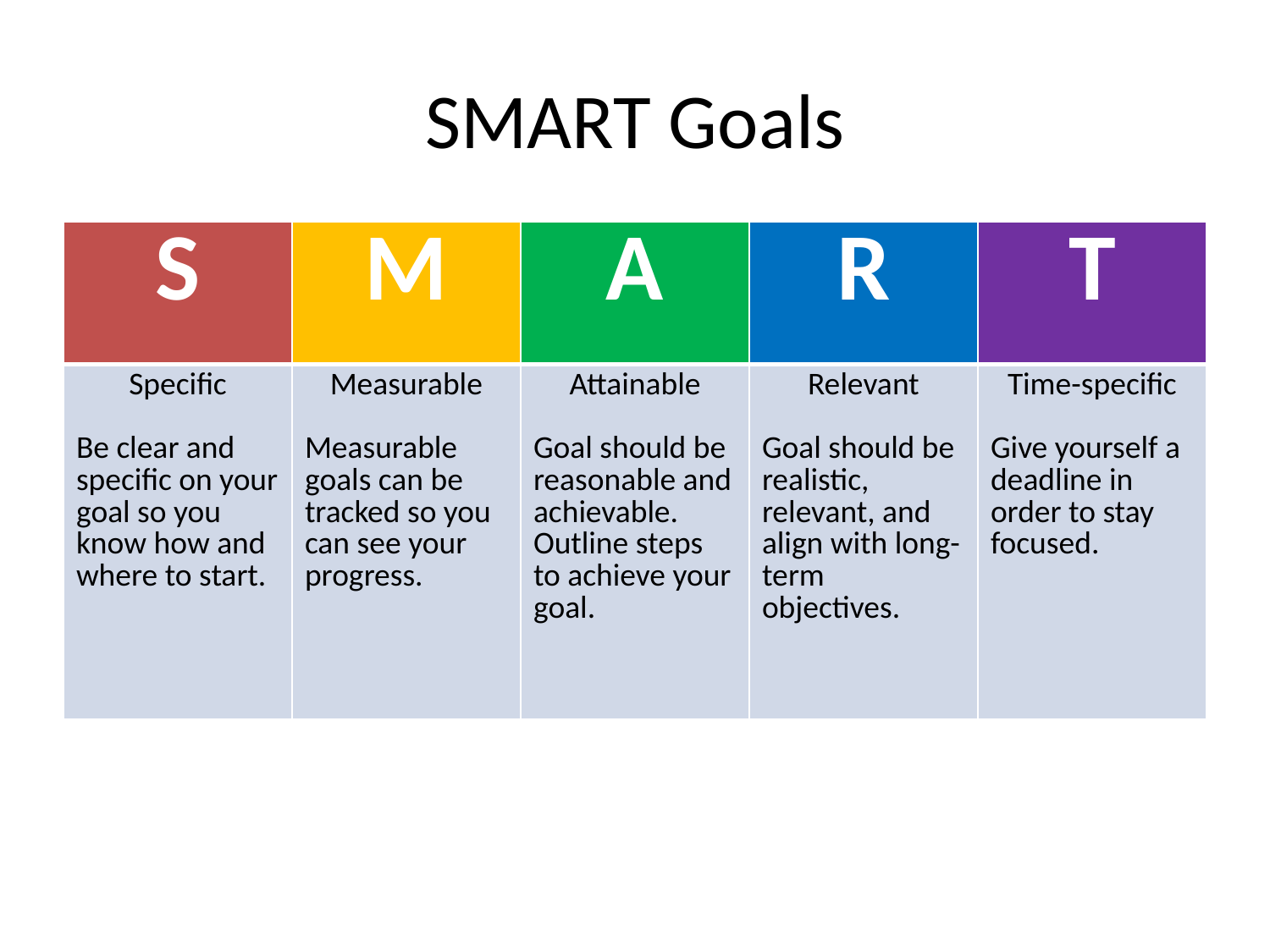

# SMART Goals
| S | M | A | R | T |
| --- | --- | --- | --- | --- |
| Specific Be clear and specific on your goal so you know how and where to start. | Measurable Measurable goals can be tracked so you can see your progress. | Attainable Goal should be reasonable and achievable. Outline steps to achieve your goal. | Relevant Goal should be realistic, relevant, and align with long-term objectives. | Time-specific Give yourself a deadline in order to stay focused. |

## Slide 30
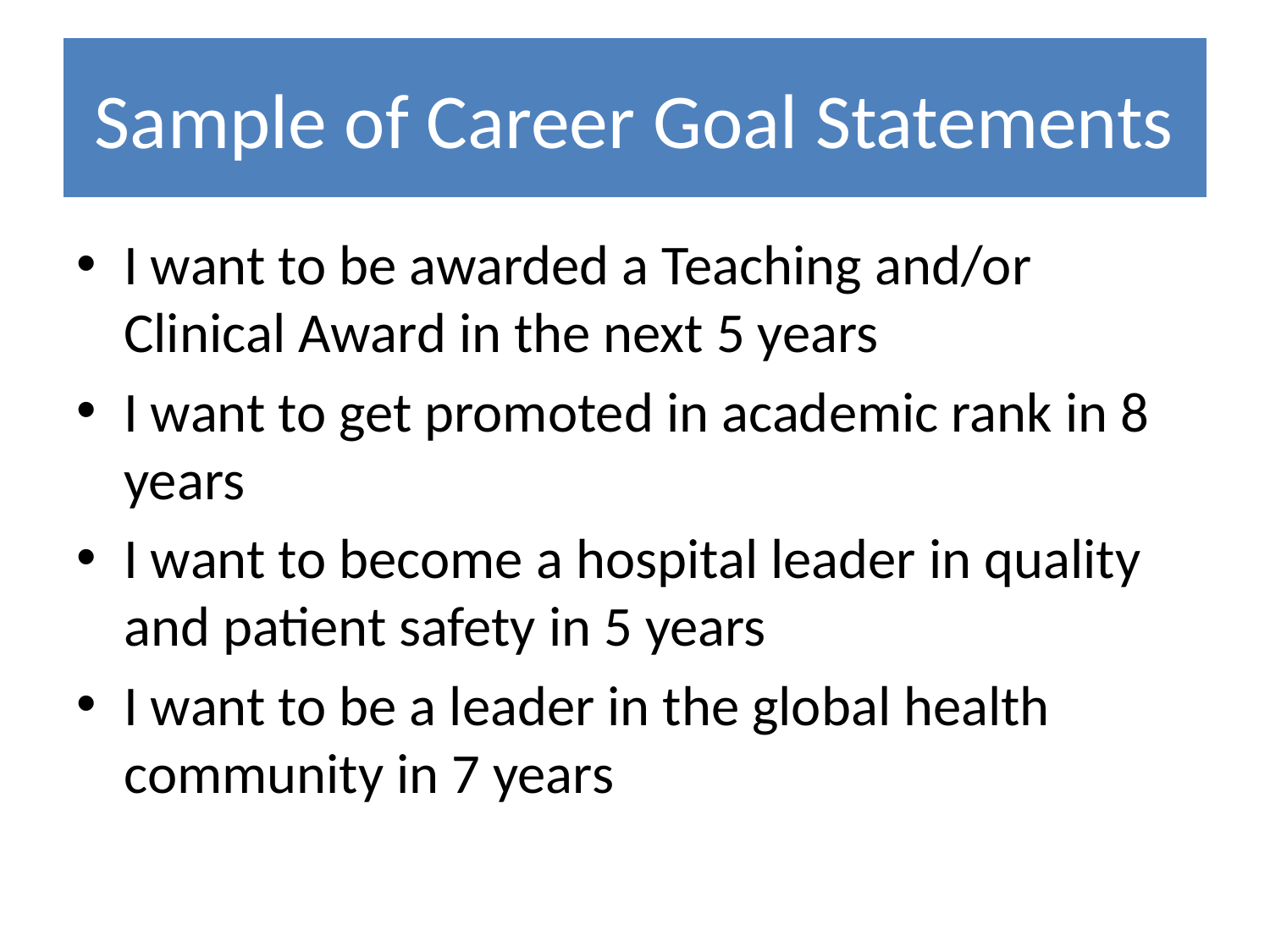

# Sample of Career Goal Statements
I want to be awarded a Teaching and/or Clinical Award in the next 5 years
I want to get promoted in academic rank in 8 years
I want to become a hospital leader in quality and patient safety in 5 years
I want to be a leader in the global health community in 7 years

## Slide 31
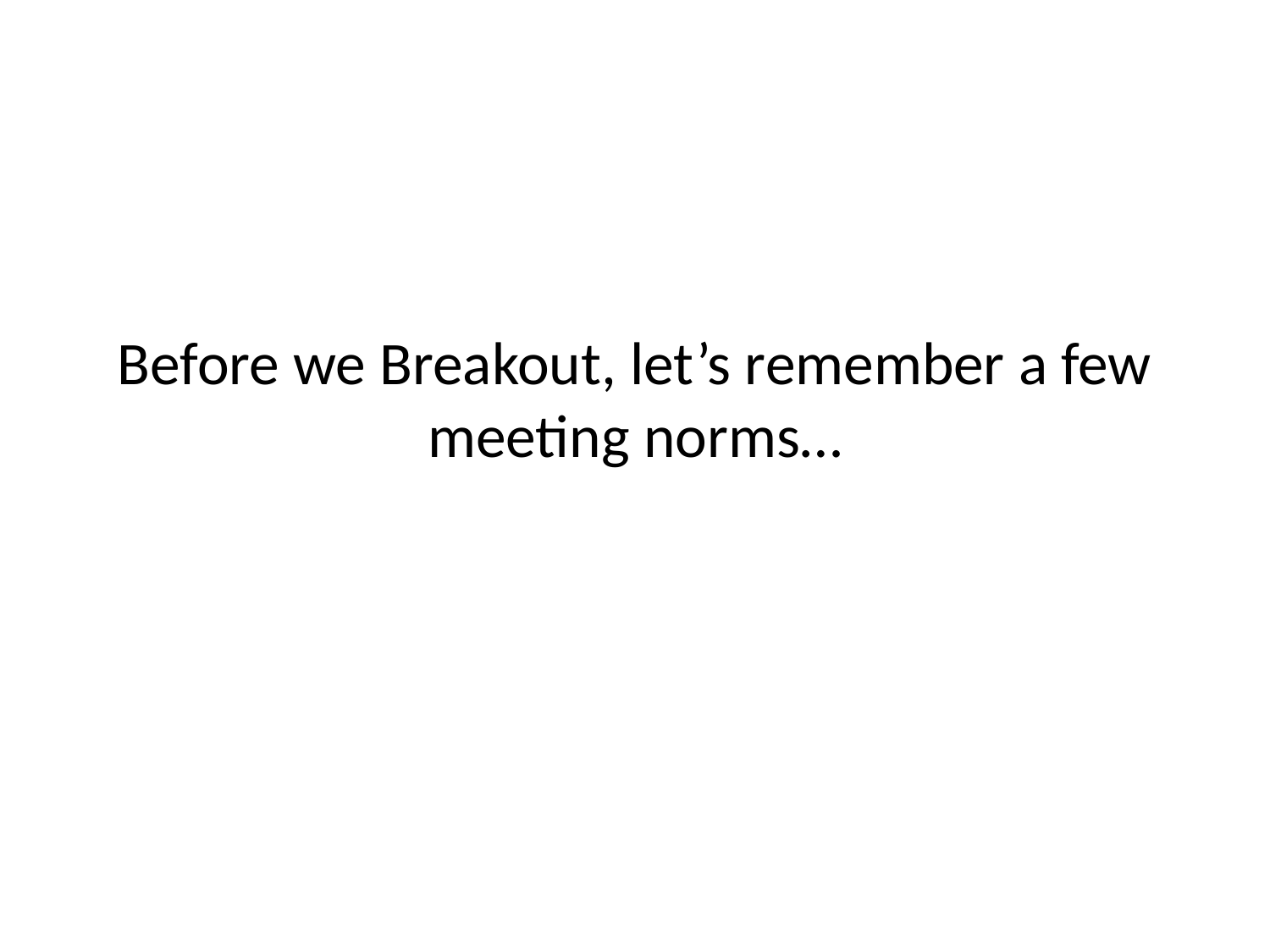

# Before we Breakout, let’s remember a few meeting norms…

## Slide 32
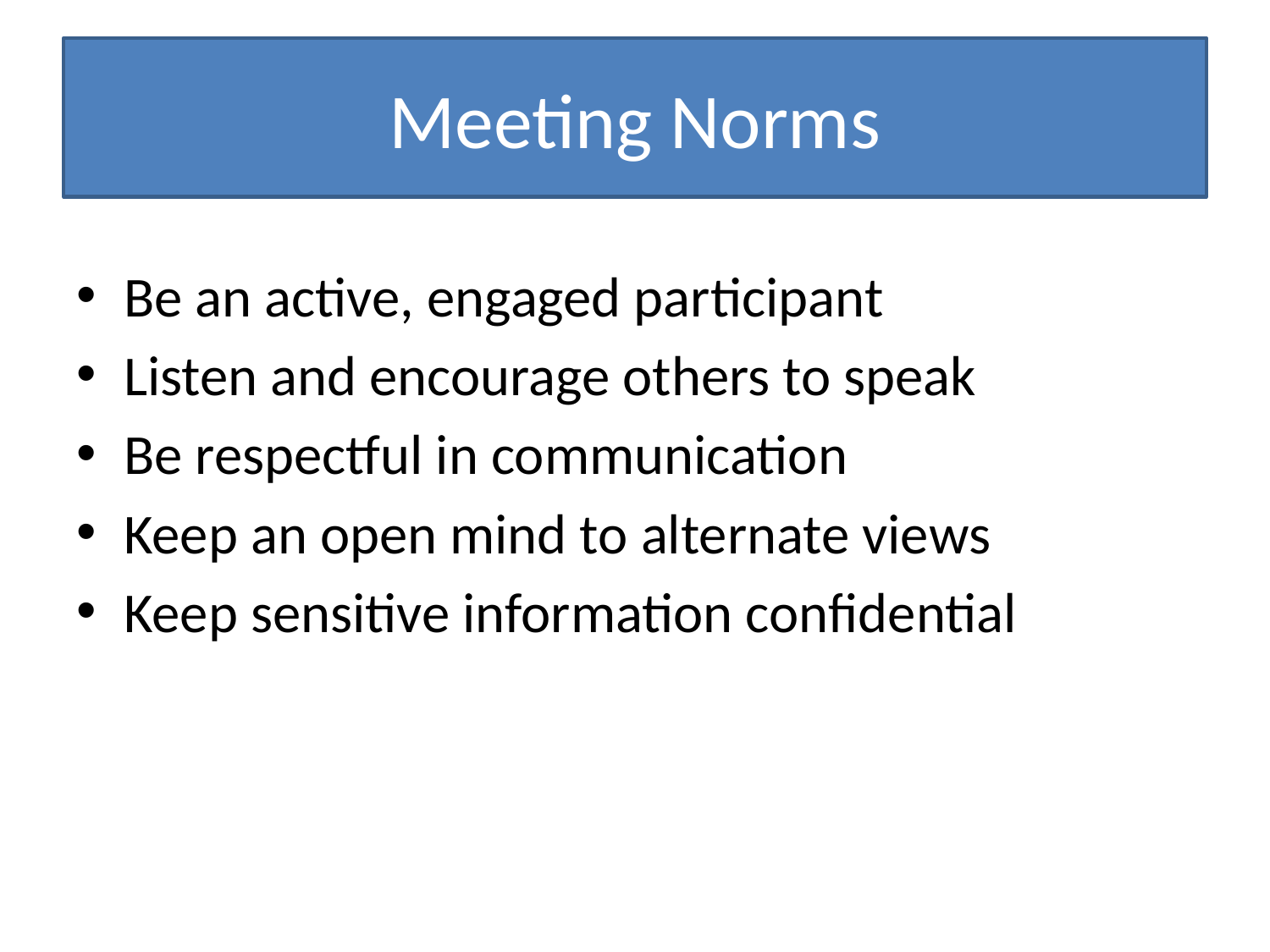

# Meeting Norms
Be an active, engaged participant
Listen and encourage others to speak
Be respectful in communication
Keep an open mind to alternate views
Keep sensitive information confidential

## Slide 33
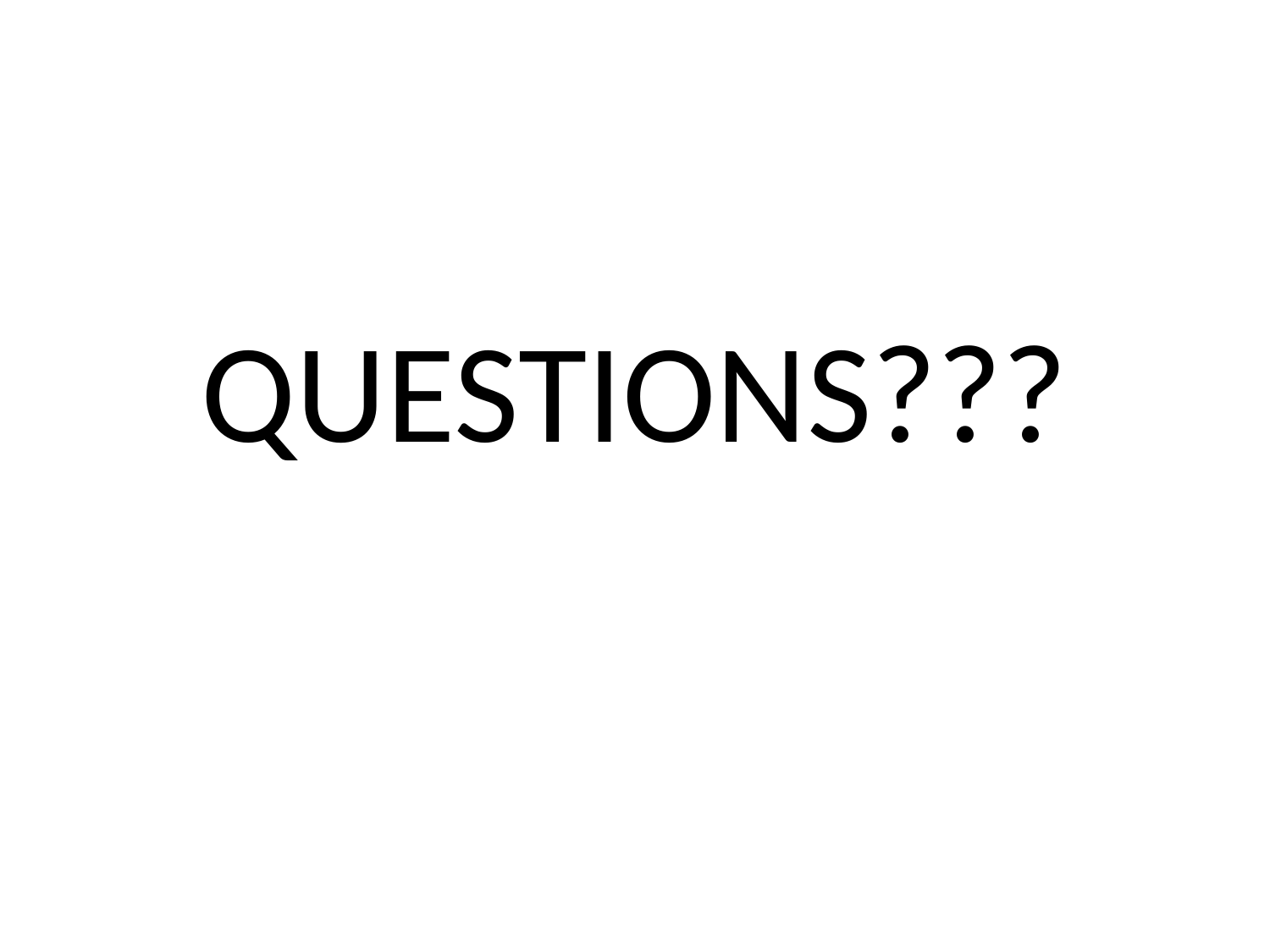

# QUESTIONS???

## Slide 34
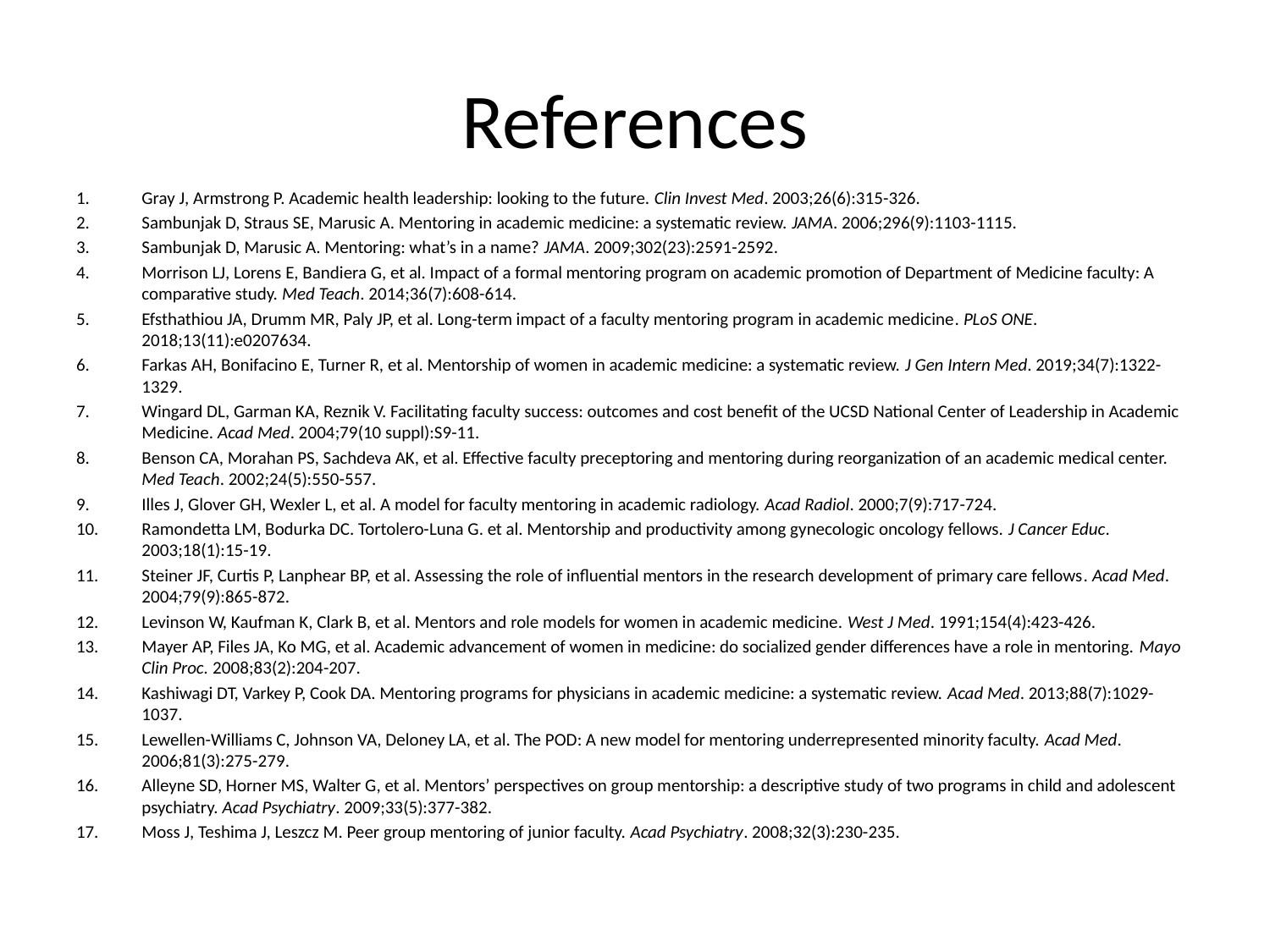

# References
Gray J, Armstrong P. Academic health leadership: looking to the future. Clin Invest Med. 2003;26(6):315-326.
Sambunjak D, Straus SE, Marusic A. Mentoring in academic medicine: a systematic review. JAMA. 2006;296(9):1103-1115.
Sambunjak D, Marusic A. Mentoring: what’s in a name? JAMA. 2009;302(23):2591-2592.
Morrison LJ, Lorens E, Bandiera G, et al. Impact of a formal mentoring program on academic promotion of Department of Medicine faculty: A comparative study. Med Teach. 2014;36(7):608-614.
Efsthathiou JA, Drumm MR, Paly JP, et al. Long-term impact of a faculty mentoring program in academic medicine. PLoS ONE. 2018;13(11):e0207634.
Farkas AH, Bonifacino E, Turner R, et al. Mentorship of women in academic medicine: a systematic review. J Gen Intern Med. 2019;34(7):1322-1329.
Wingard DL, Garman KA, Reznik V. Facilitating faculty success: outcomes and cost benefit of the UCSD National Center of Leadership in Academic Medicine. Acad Med. 2004;79(10 suppl):S9-11.
Benson CA, Morahan PS, Sachdeva AK, et al. Effective faculty preceptoring and mentoring during reorganization of an academic medical center. Med Teach. 2002;24(5):550-557.
Illes J, Glover GH, Wexler L, et al. A model for faculty mentoring in academic radiology. Acad Radiol. 2000;7(9):717-724.
Ramondetta LM, Bodurka DC. Tortolero-Luna G. et al. Mentorship and productivity among gynecologic oncology fellows. J Cancer Educ. 2003;18(1):15-19.
Steiner JF, Curtis P, Lanphear BP, et al. Assessing the role of influential mentors in the research development of primary care fellows. Acad Med. 2004;79(9):865-872.
Levinson W, Kaufman K, Clark B, et al. Mentors and role models for women in academic medicine. West J Med. 1991;154(4):423-426.
Mayer AP, Files JA, Ko MG, et al. Academic advancement of women in medicine: do socialized gender differences have a role in mentoring. Mayo Clin Proc. 2008;83(2):204-207.
Kashiwagi DT, Varkey P, Cook DA. Mentoring programs for physicians in academic medicine: a systematic review. Acad Med. 2013;88(7):1029-1037.
Lewellen-Williams C, Johnson VA, Deloney LA, et al. The POD: A new model for mentoring underrepresented minority faculty. Acad Med. 2006;81(3):275-279.
Alleyne SD, Horner MS, Walter G, et al. Mentors’ perspectives on group mentorship: a descriptive study of two programs in child and adolescent psychiatry. Acad Psychiatry. 2009;33(5):377-382.
Moss J, Teshima J, Leszcz M. Peer group mentoring of junior faculty. Acad Psychiatry. 2008;32(3):230-235.
