# Supplementary material for: A Facilitated Peer Mentoring Program With a Dedicated Curriculum to Foster Career Advancement of Academic Hospitalists
Source: MedEdPORTAL. 2023 Dec 8;19:11366. doi: 10.15766/mep_2374-8265.11366 (PMC10704005; doi:10.15766/mep_2374-8265.11366)
Supplement: Supplementary file 1 — Preprogram Survey.docxPostprogram Survey.docxLarge-Group Session 1.pptxLarge-Group Session 2.pptxLarge-Group Session 3.pptxLarge-Group Session 4.pptxSmall-Group Session 1 Facilitator Guide.docxSmall-Group Session 2 Facilitator Guide.docxSmall-Group Session 3 Facilitator Guide.docx [file mep_2374-8265.11366-s001.zip › D. Large-Group Session 2.pptx]

## Slide 1
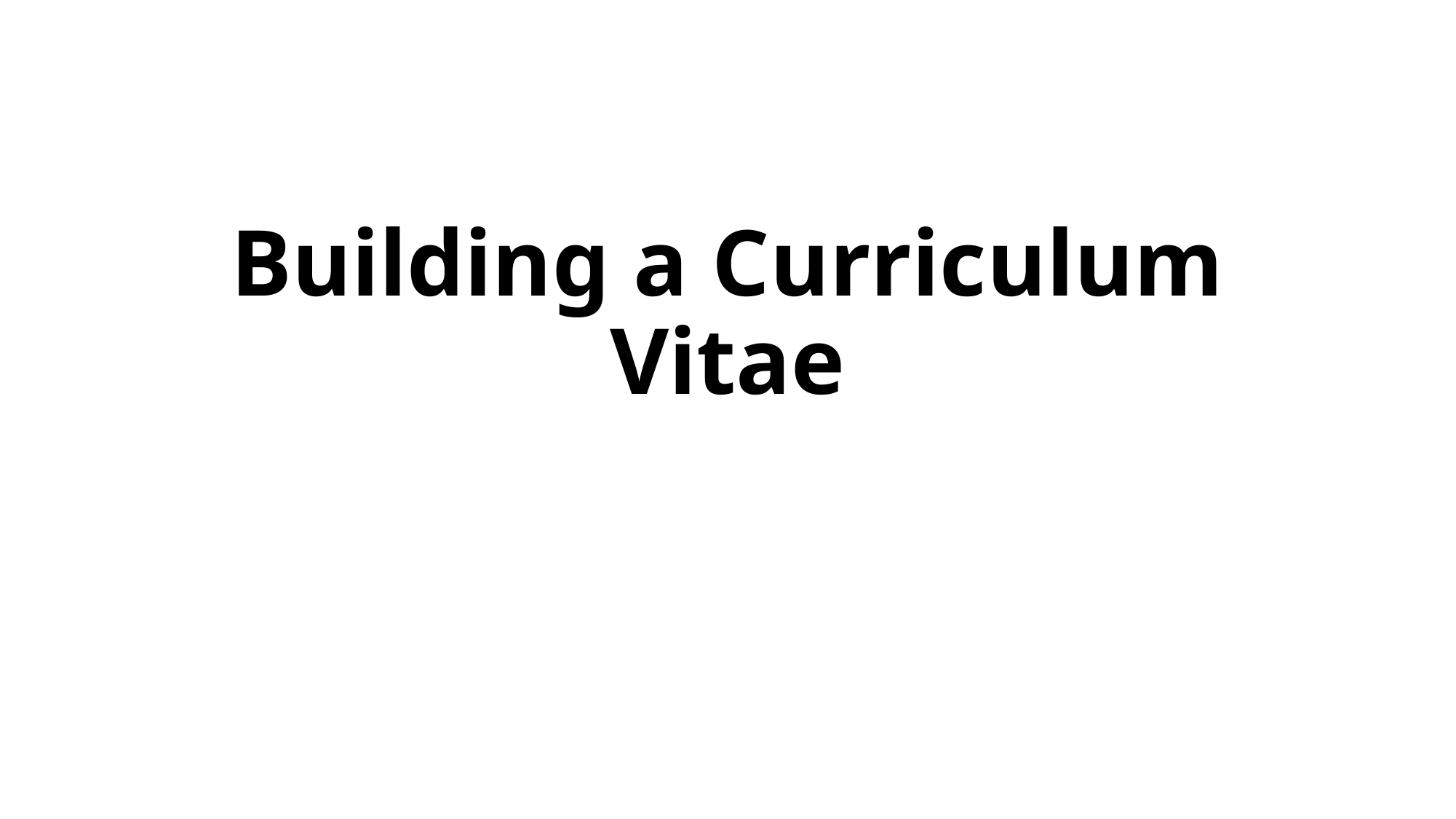

# Building a Curriculum Vitae

## Slide 2
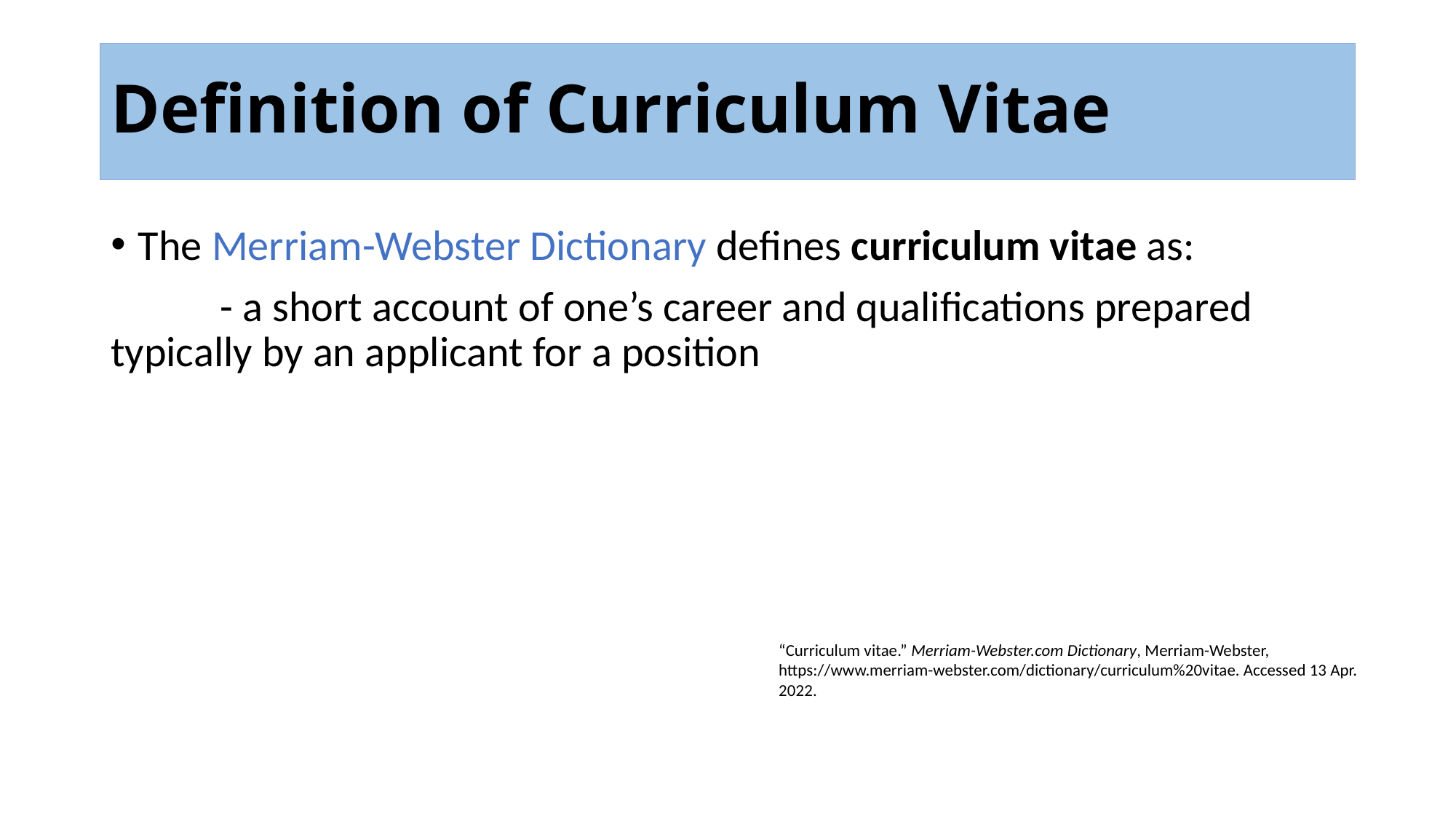

# Definition of Curriculum Vitae
The Merriam-Webster Dictionary defines curriculum vitae as:
	- a short account of one’s career and qualifications prepared 	typically by an applicant for a position
“Curriculum vitae.” Merriam-Webster.com Dictionary, Merriam-Webster, https://www.merriam-webster.com/dictionary/curriculum%20vitae. Accessed 13 Apr. 2022.

## Slide 3
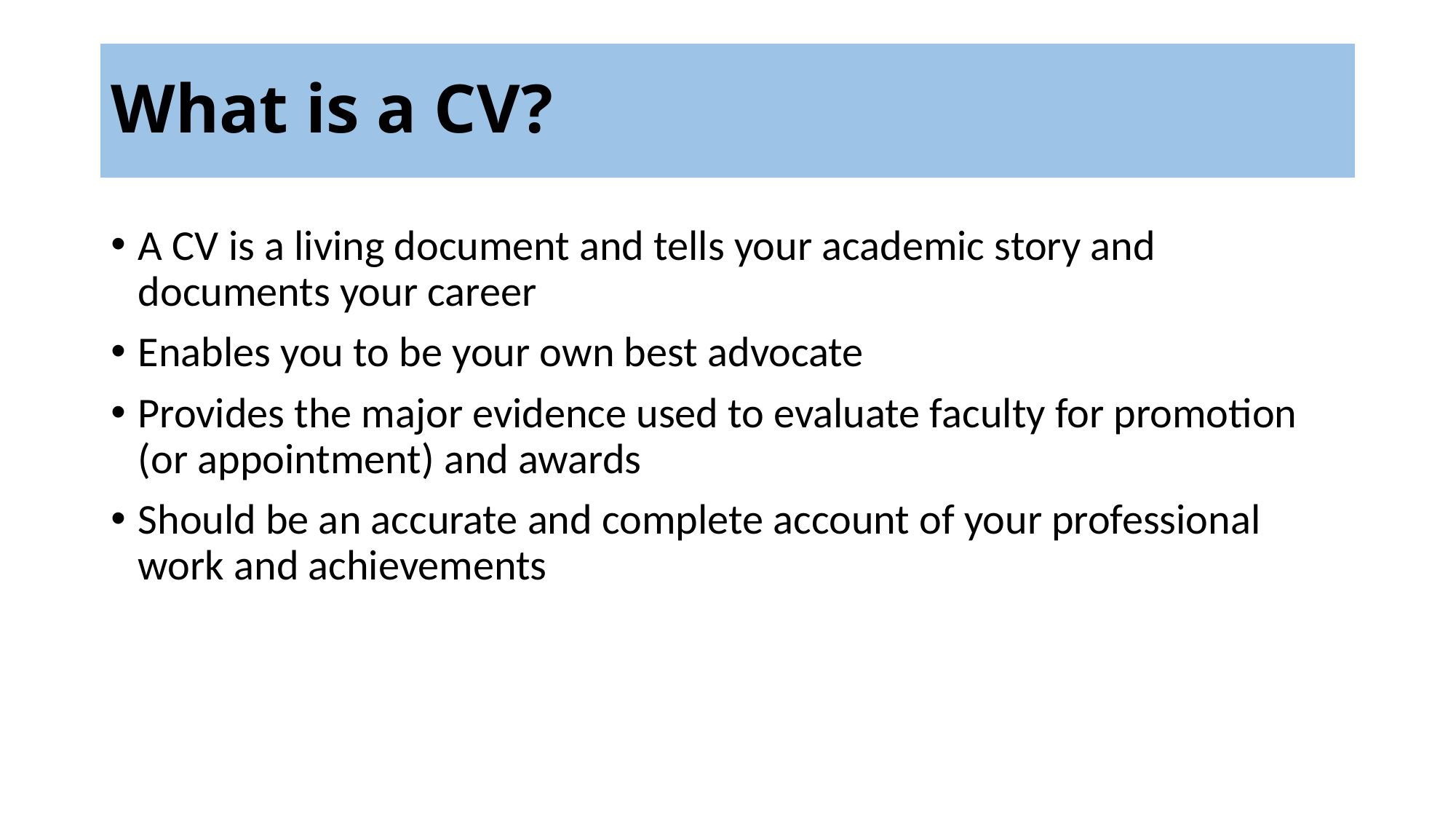

# What is a CV?
A CV is a living document and tells your academic story and documents your career
Enables you to be your own best advocate
Provides the major evidence used to evaluate faculty for promotion (or appointment) and awards
Should be an accurate and complete account of your professional work and achievements

## Slide 4
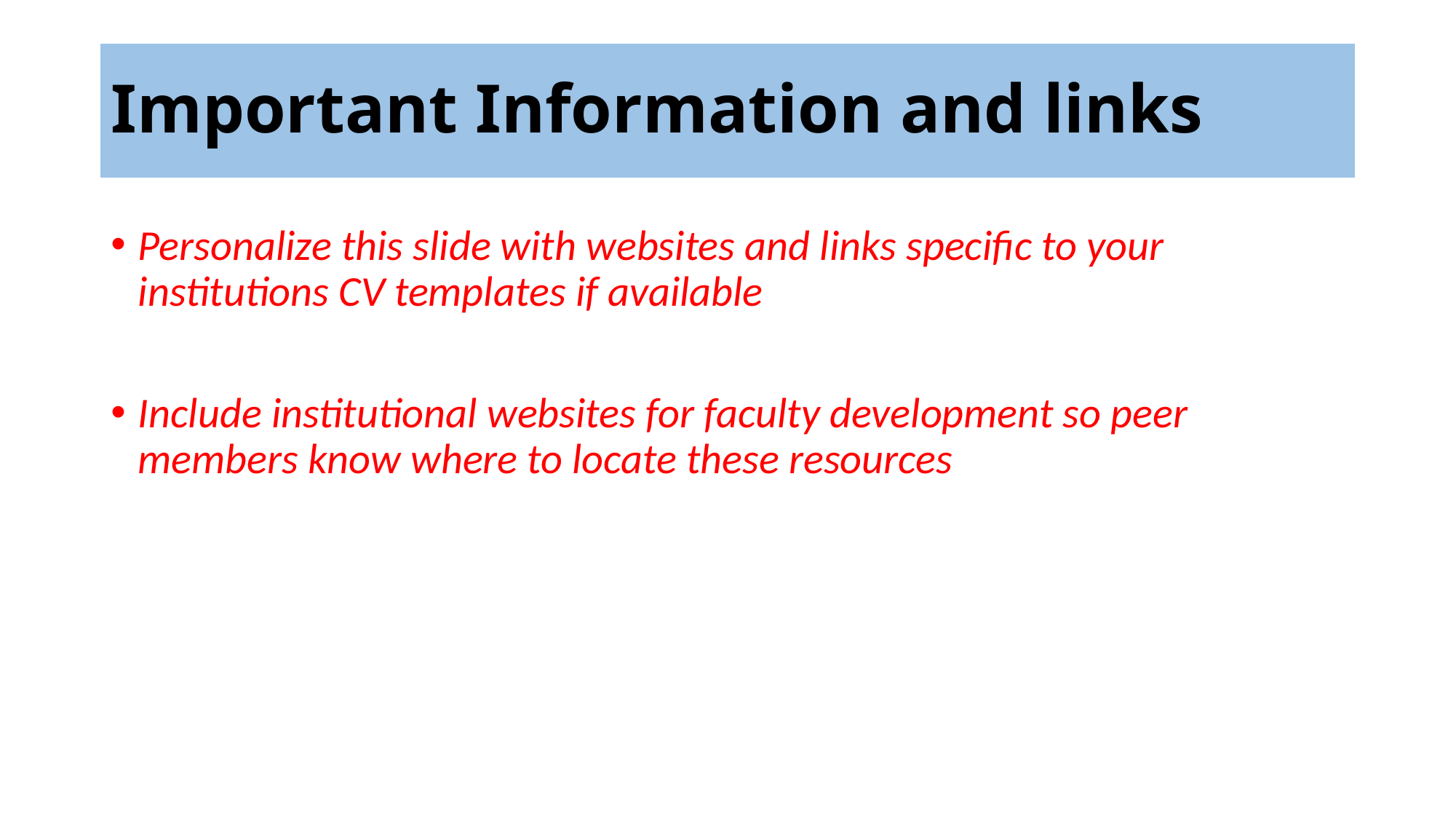

# Important Information and links
Personalize this slide with websites and links specific to your institutions CV templates if available
Include institutional websites for faculty development so peer members know where to locate these resources

## Slide 5
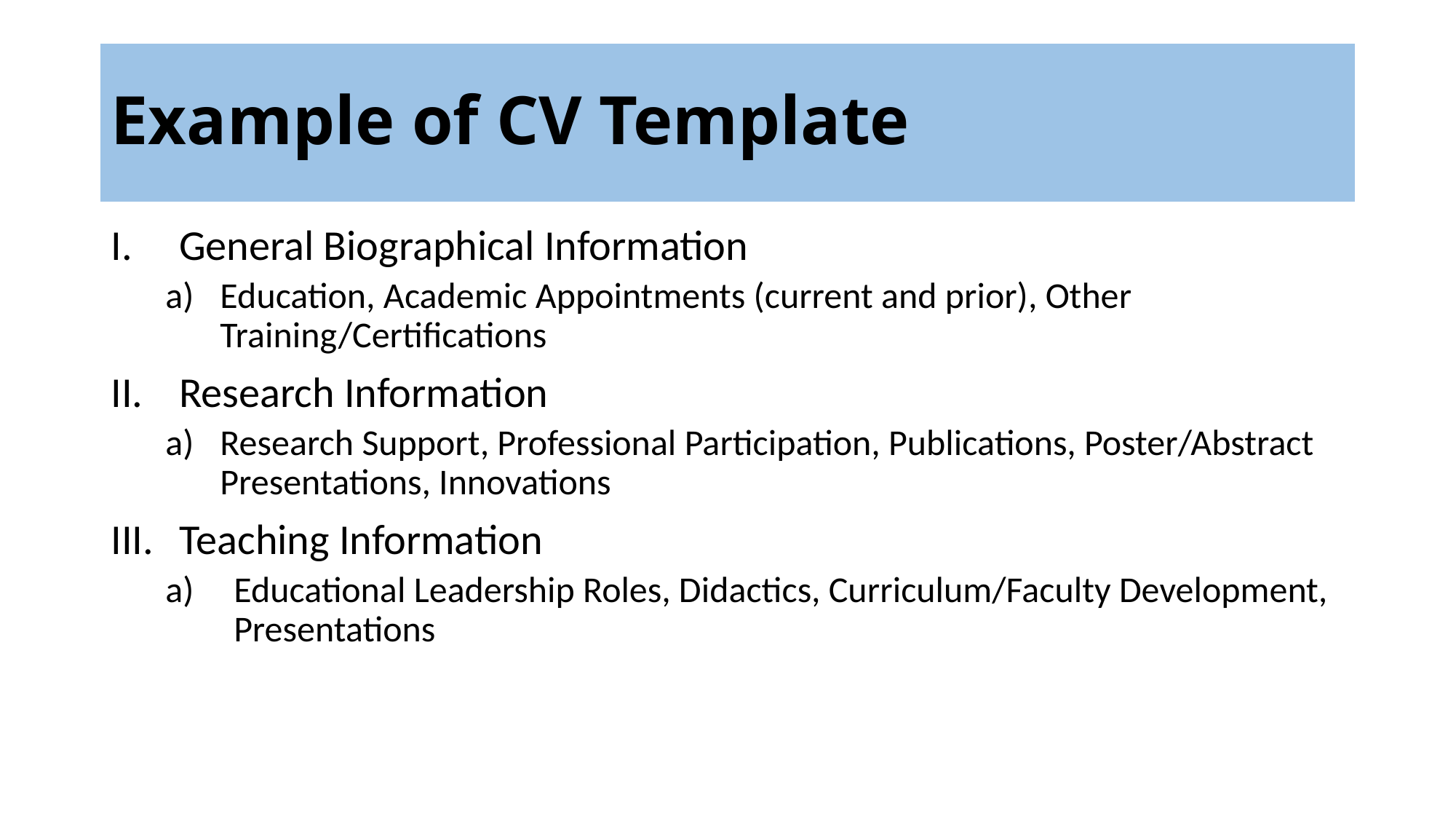

# Example of CV Template
General Biographical Information
Education, Academic Appointments (current and prior), Other Training/Certifications
Research Information
Research Support, Professional Participation, Publications, Poster/Abstract Presentations, Innovations
Teaching Information
Educational Leadership Roles, Didactics, Curriculum/Faculty Development, Presentations

## Slide 6
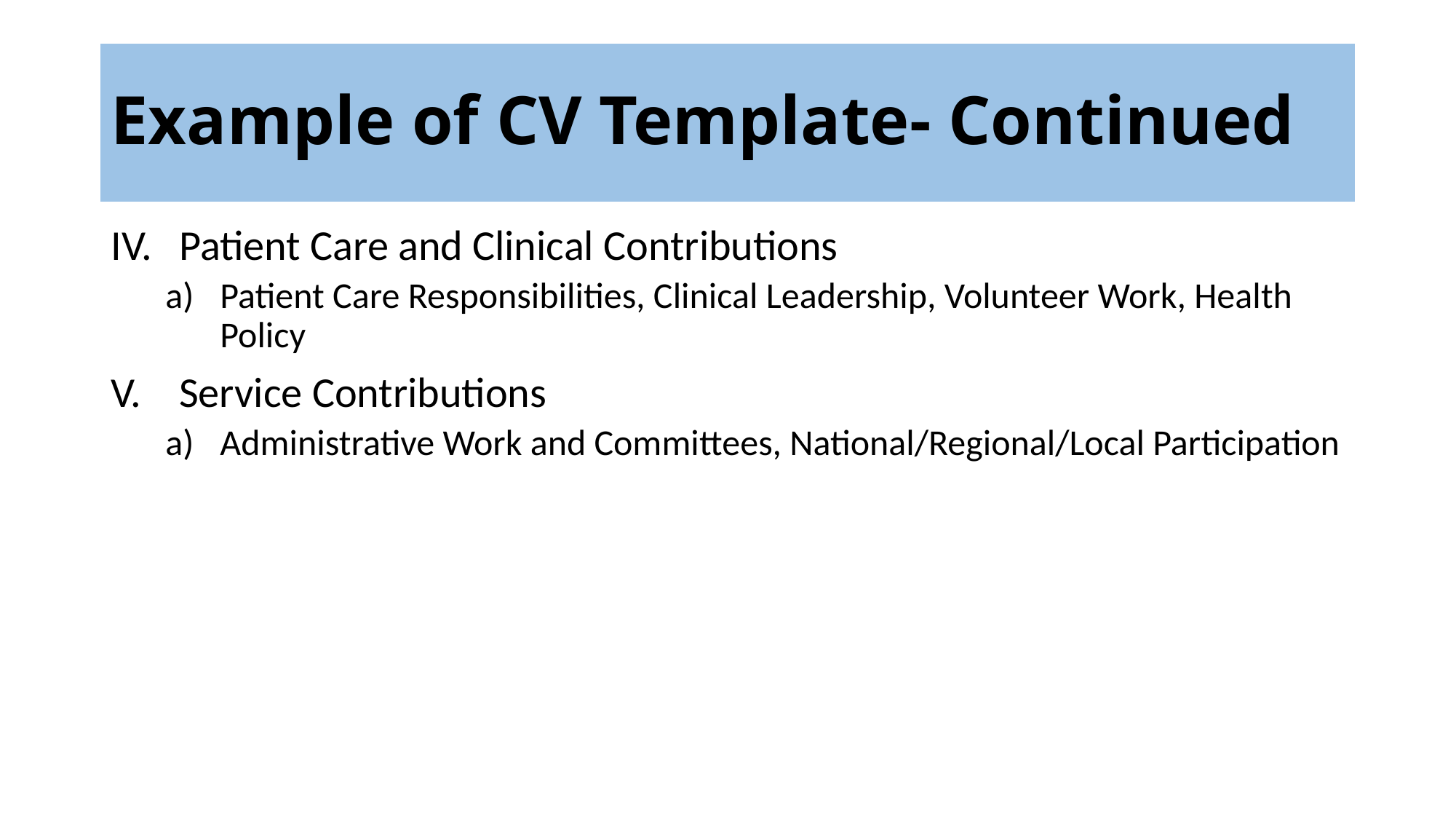

# Example of CV Template- Continued
Patient Care and Clinical Contributions
Patient Care Responsibilities, Clinical Leadership, Volunteer Work, Health Policy
Service Contributions
Administrative Work and Committees, National/Regional/Local Participation

## Slide 7
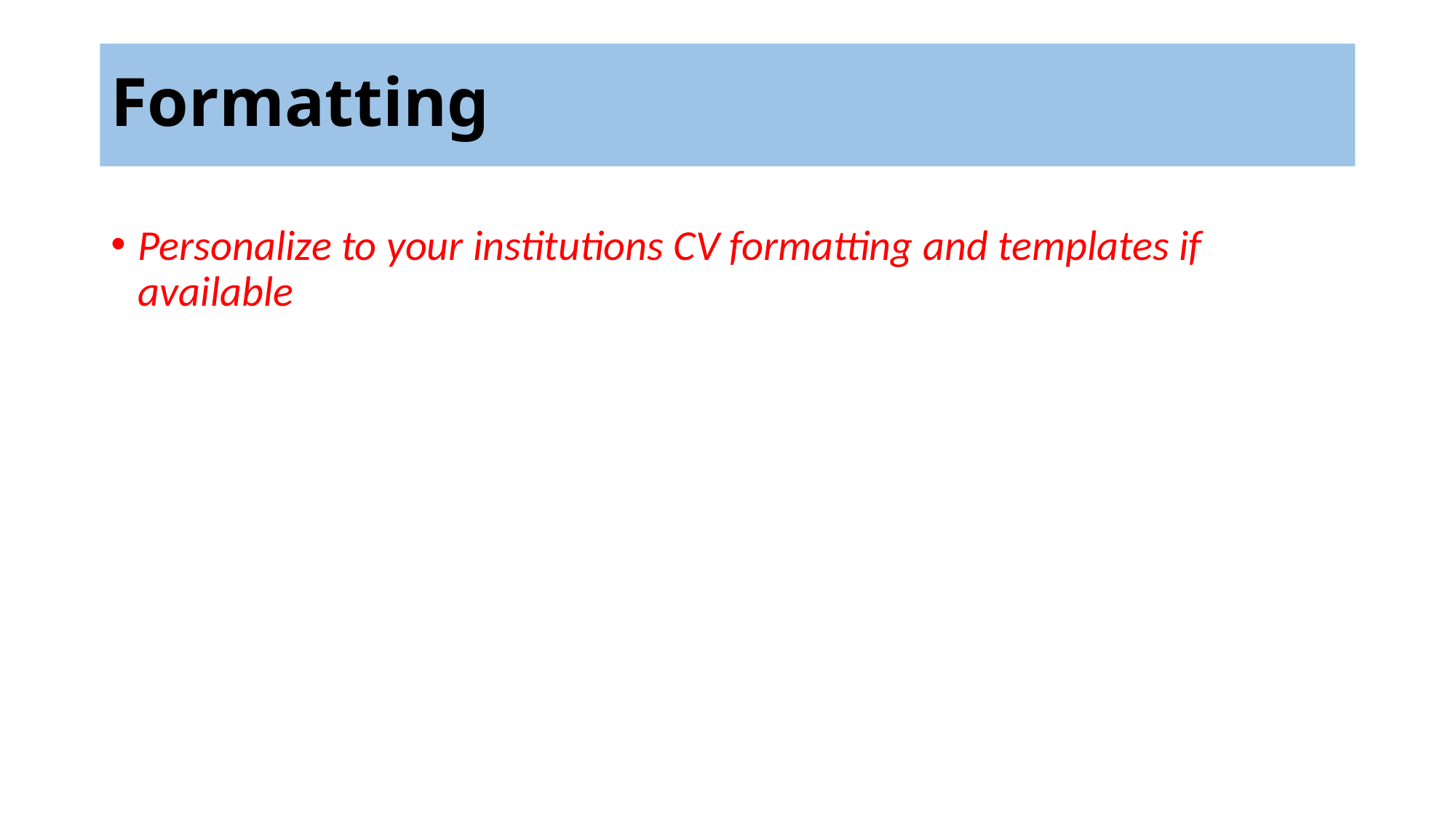

# Formatting
Personalize to your institutions CV formatting and templates if available

## Slide 8
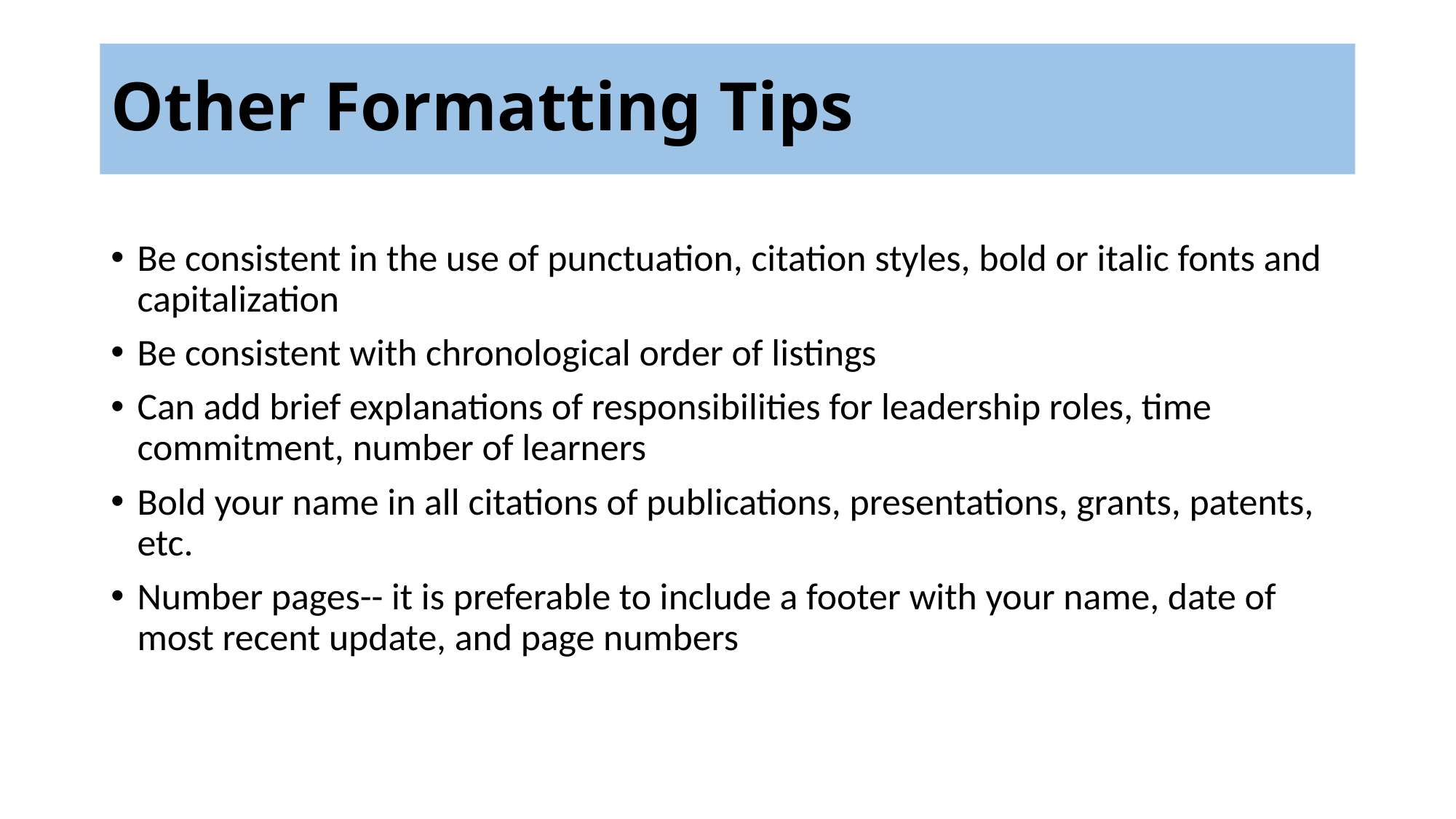

# Other Formatting Tips
Be consistent in the use of punctuation, citation styles, bold or italic fonts and capitalization
Be consistent with chronological order of listings
Can add brief explanations of responsibilities for leadership roles, time commitment, number of learners
Bold your name in all citations of publications, presentations, grants, patents, etc.
Number pages-- it is preferable to include a footer with your name, date of most recent update, and page numbers

## Slide 9
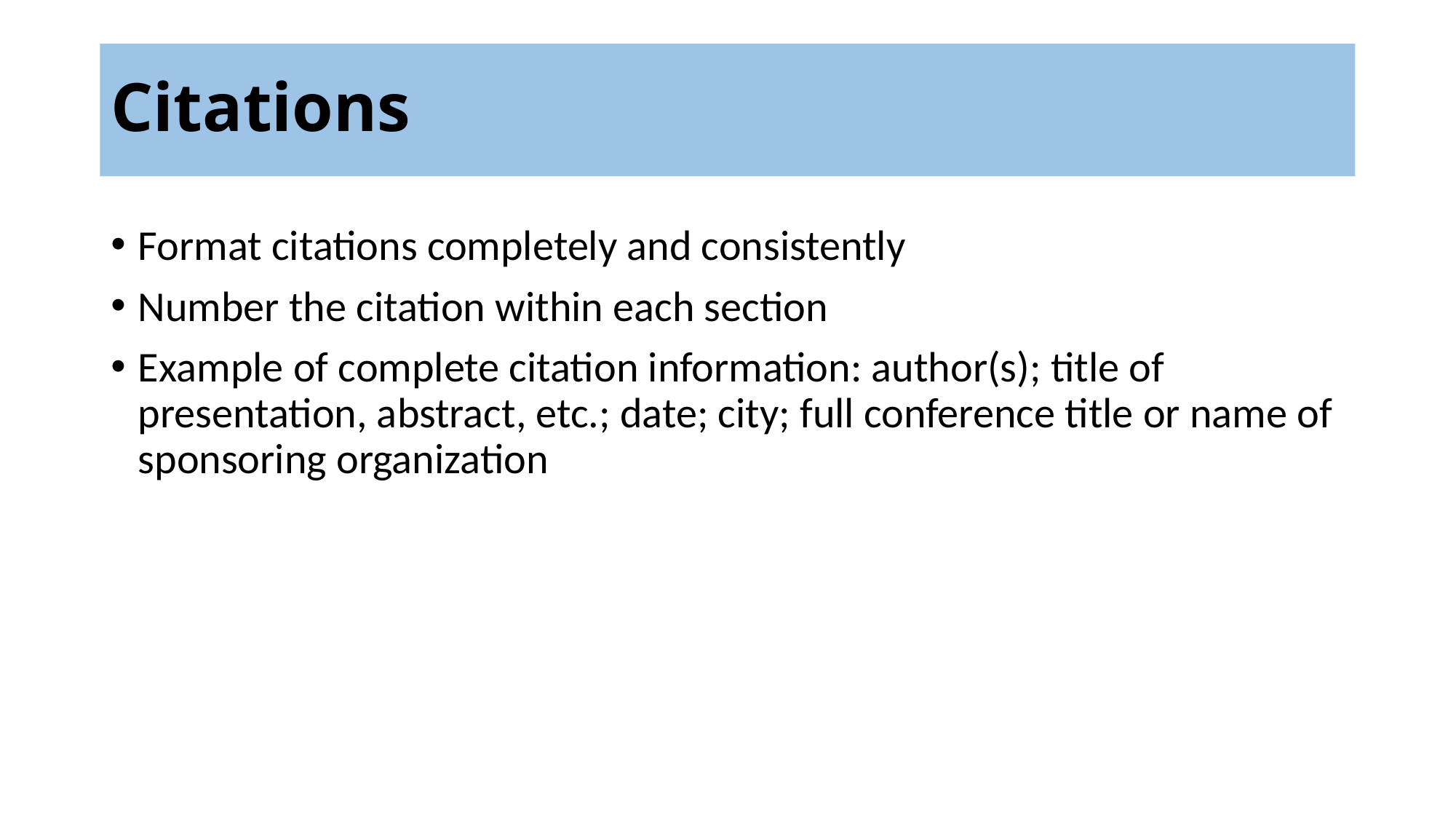

# Citations
Format citations completely and consistently
Number the citation within each section
Example of complete citation information: author(s); title of presentation, abstract, etc.; date; city; full conference title or name of sponsoring organization

## Slide 10
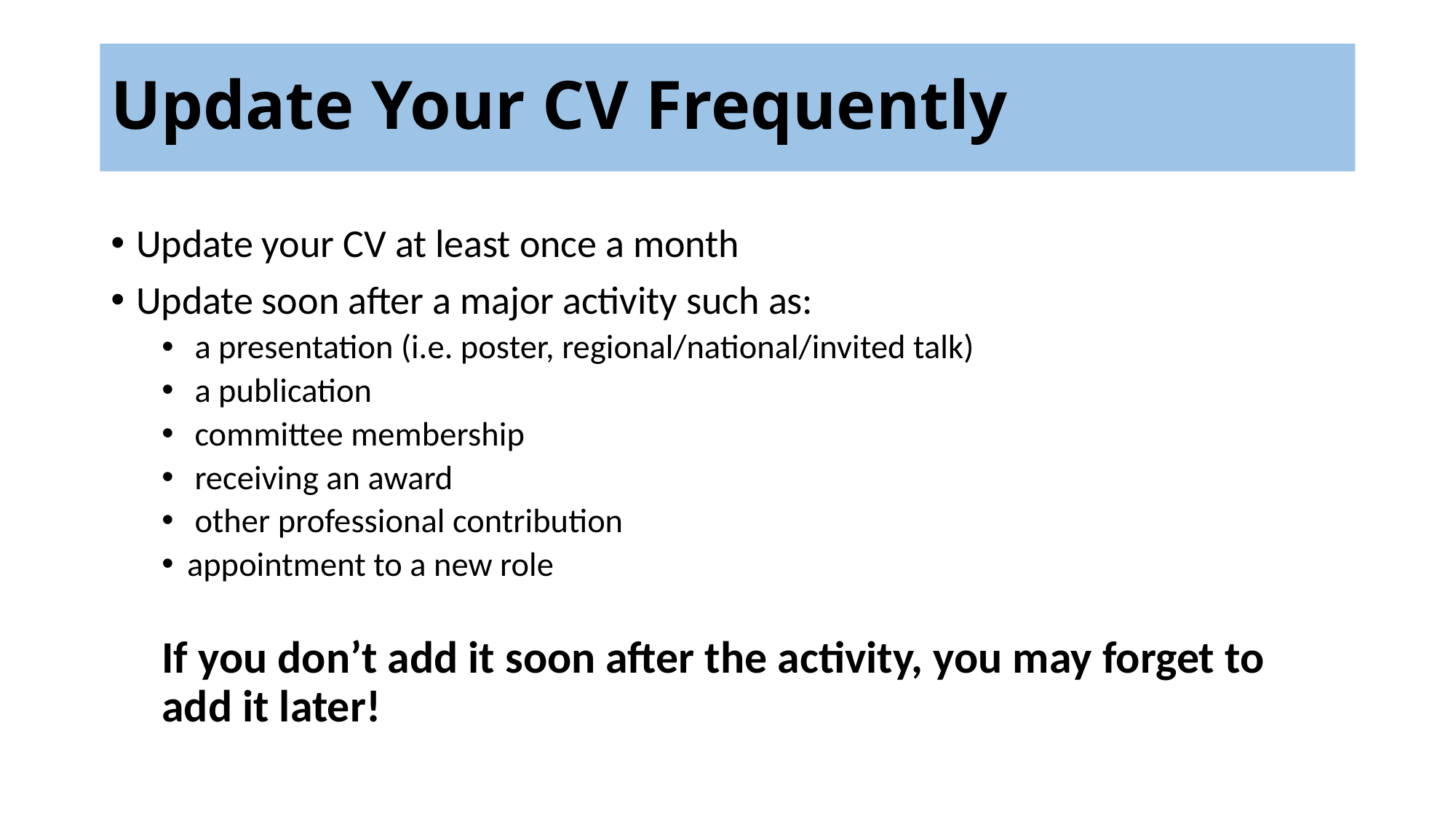

# Update Your CV Frequently
Update your CV at least once a month
Update soon after a major activity such as:
 a presentation (i.e. poster, regional/national/invited talk)
 a publication
 committee membership
 receiving an award
 other professional contribution
appointment to a new role
If you don’t add it soon after the activity, you may forget to add it later!

## Slide 11
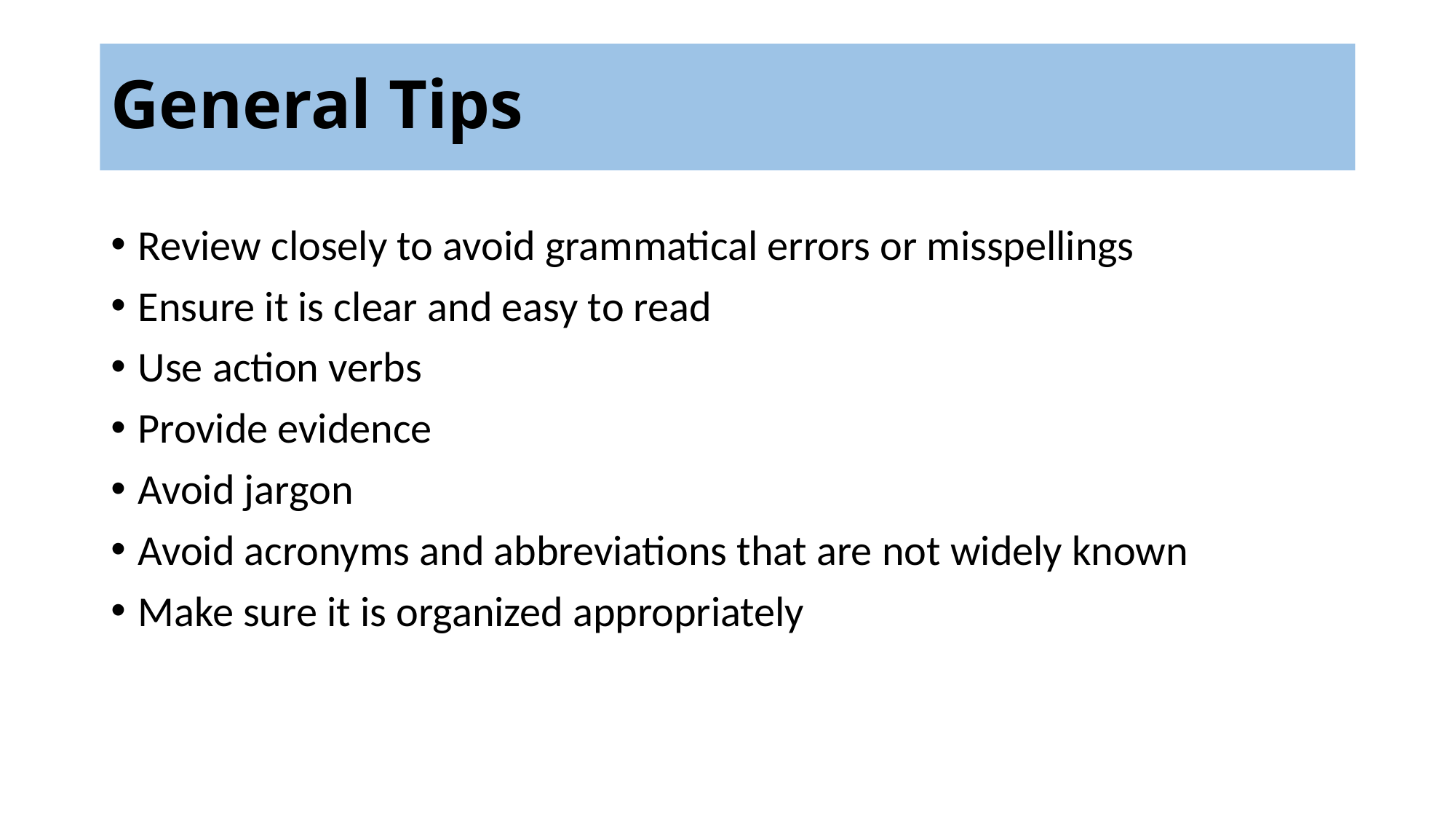

# General Tips
Review closely to avoid grammatical errors or misspellings
Ensure it is clear and easy to read
Use action verbs
Provide evidence
Avoid jargon
Avoid acronyms and abbreviations that are not widely known
Make sure it is organized appropriately

## Slide 12
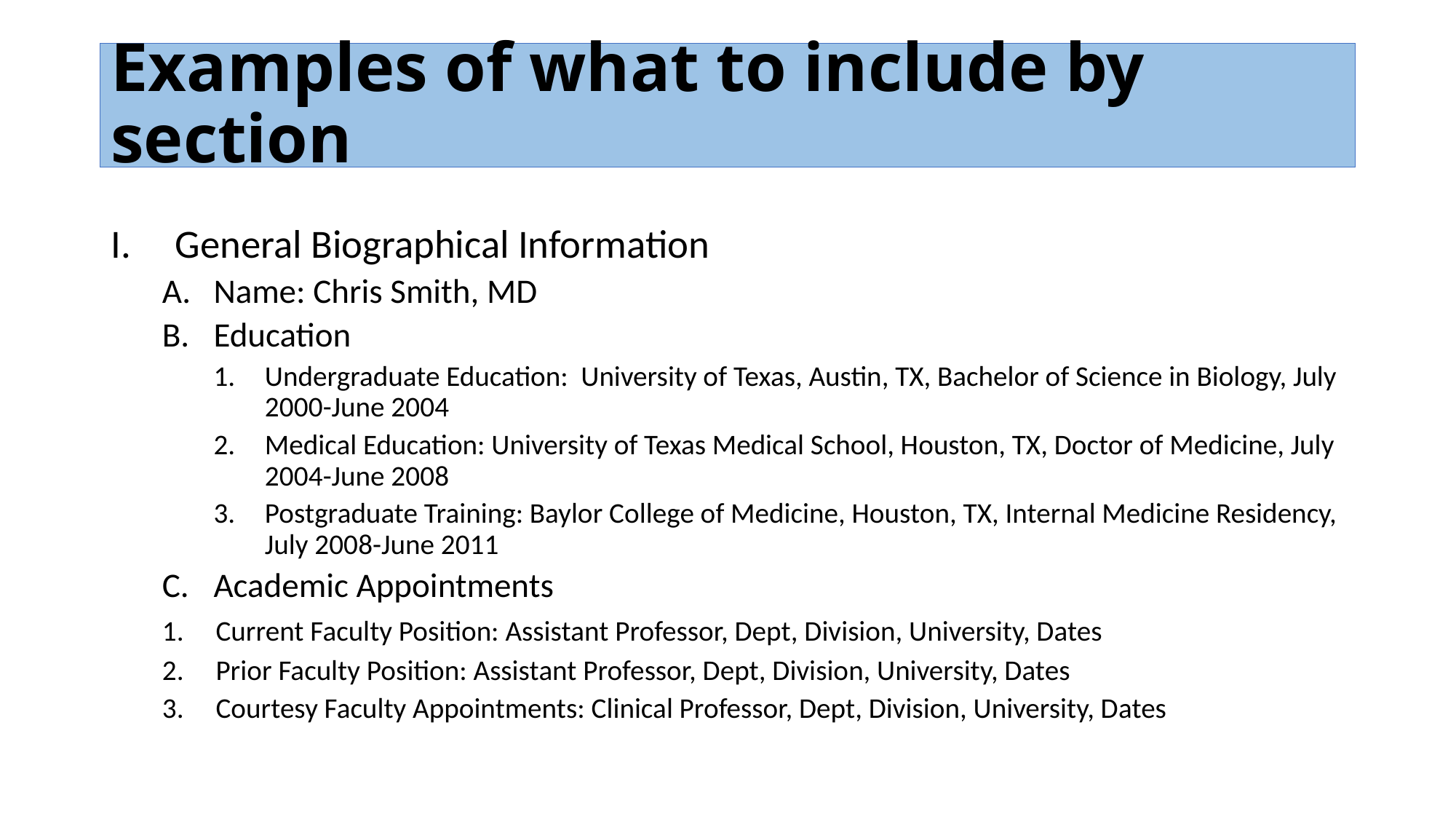

# Examples of what to include by section
General Biographical Information
Name: Chris Smith, MD
Education
Undergraduate Education: University of Texas, Austin, TX, Bachelor of Science in Biology, July 2000-June 2004
Medical Education: University of Texas Medical School, Houston, TX, Doctor of Medicine, July 2004-June 2008
Postgraduate Training: Baylor College of Medicine, Houston, TX, Internal Medicine Residency, July 2008-June 2011
Academic Appointments
	1. Current Faculty Position: Assistant Professor, Dept, Division, University, Dates
	2. Prior Faculty Position: Assistant Professor, Dept, Division, University, Dates
	3. Courtesy Faculty Appointments: Clinical Professor, Dept, Division, University, Dates

## Slide 13
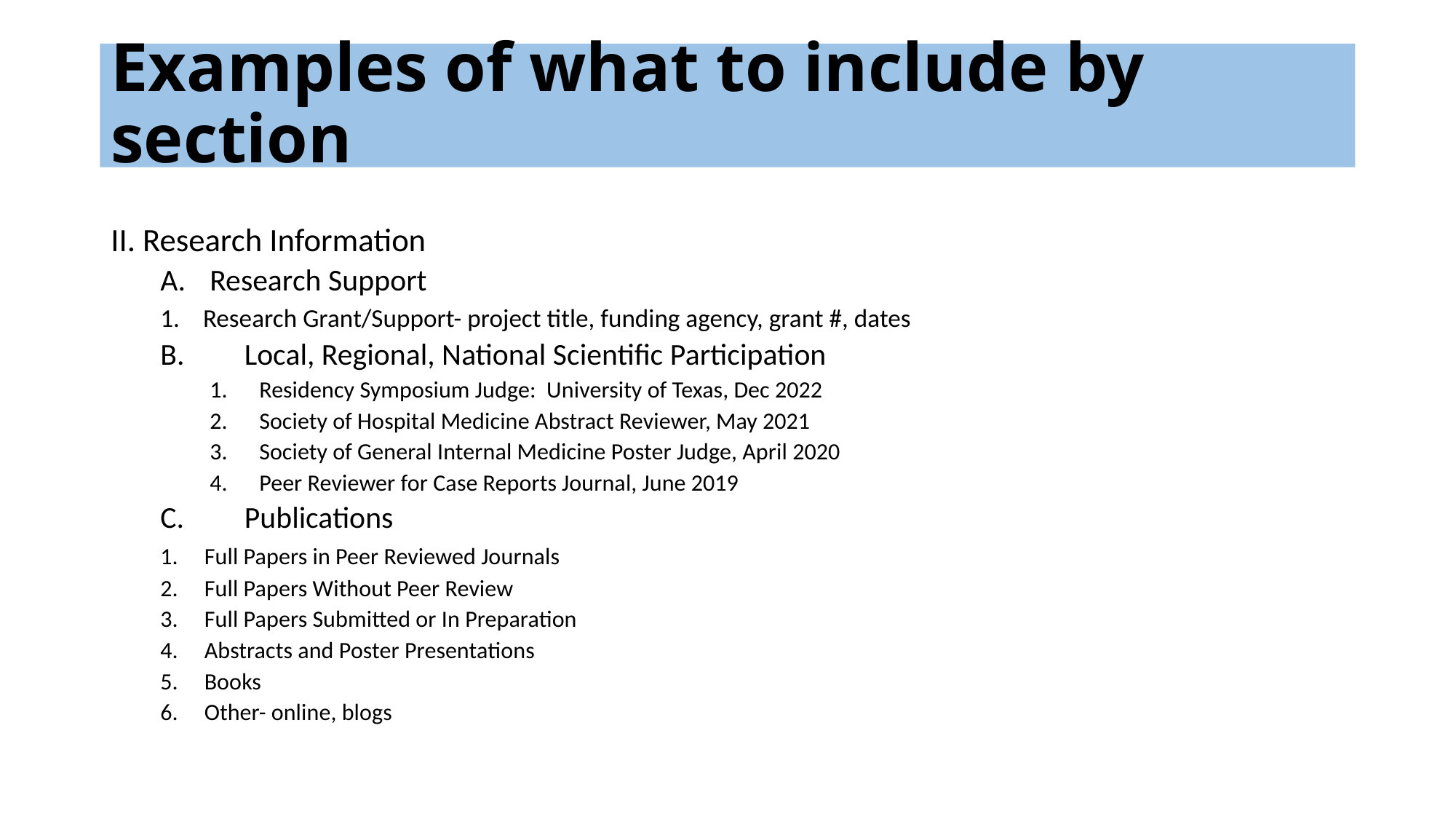

# Examples of what to include by section
II. Research Information
Research Support
	1. Research Grant/Support- project title, funding agency, grant #, dates
B.	Local, Regional, National Scientific Participation
Residency Symposium Judge: University of Texas, Dec 2022
Society of Hospital Medicine Abstract Reviewer, May 2021
Society of General Internal Medicine Poster Judge, April 2020
Peer Reviewer for Case Reports Journal, June 2019
C. 	Publications
	1. Full Papers in Peer Reviewed Journals
	2. Full Papers Without Peer Review
	3. Full Papers Submitted or In Preparation
	4. Abstracts and Poster Presentations
	5. Books
	6. Other- online, blogs

## Slide 14
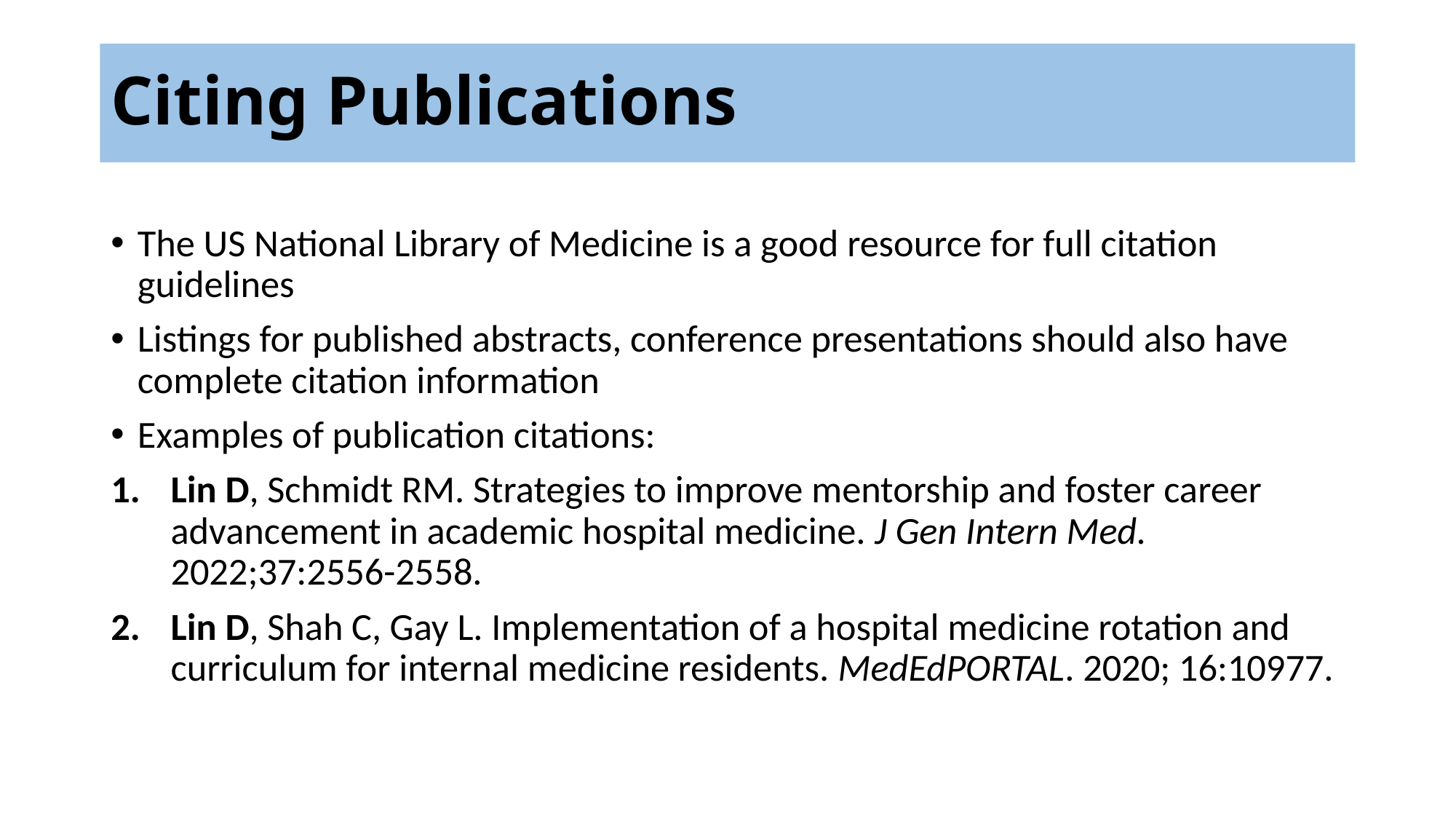

# Citing Publications
The US National Library of Medicine is a good resource for full citation guidelines
Listings for published abstracts, conference presentations should also have complete citation information
Examples of publication citations:
Lin D, Schmidt RM. Strategies to improve mentorship and foster career advancement in academic hospital medicine. J Gen Intern Med. 2022;37:2556-2558.
Lin D, Shah C, Gay L. Implementation of a hospital medicine rotation and curriculum for internal medicine residents. MedEdPORTAL. 2020; 16:10977.

## Slide 15
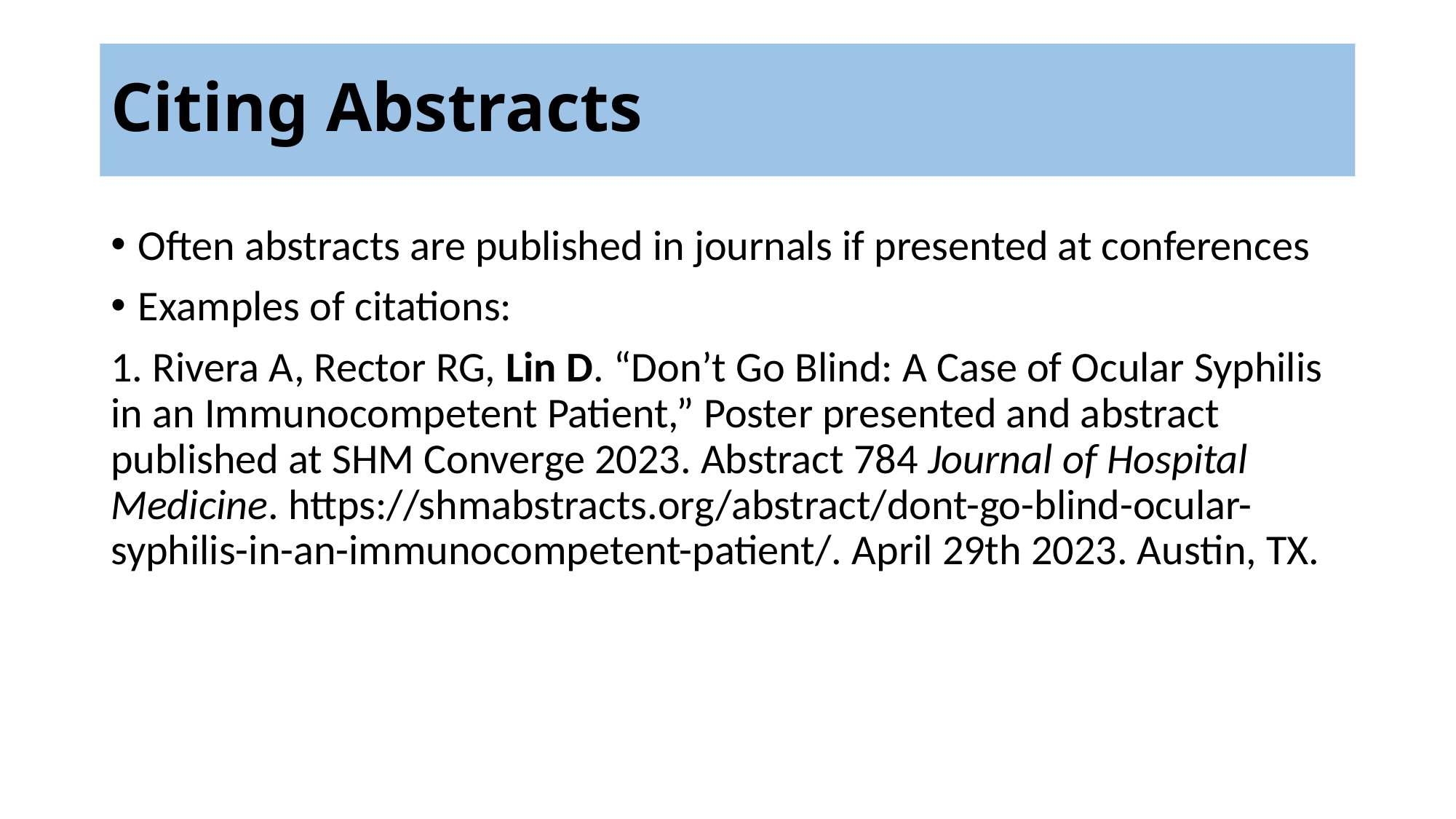

# Citing Abstracts
Often abstracts are published in journals if presented at conferences
Examples of citations:
1. Rivera A, Rector RG, Lin D. “Don’t Go Blind: A Case of Ocular Syphilis in an Immunocompetent Patient,” Poster presented and abstract published at SHM Converge 2023. Abstract 784 Journal of Hospital Medicine. https://shmabstracts.org/abstract/dont-go-blind-ocular-syphilis-in-an-immunocompetent-patient/. April 29th 2023. Austin, TX.

## Slide 16
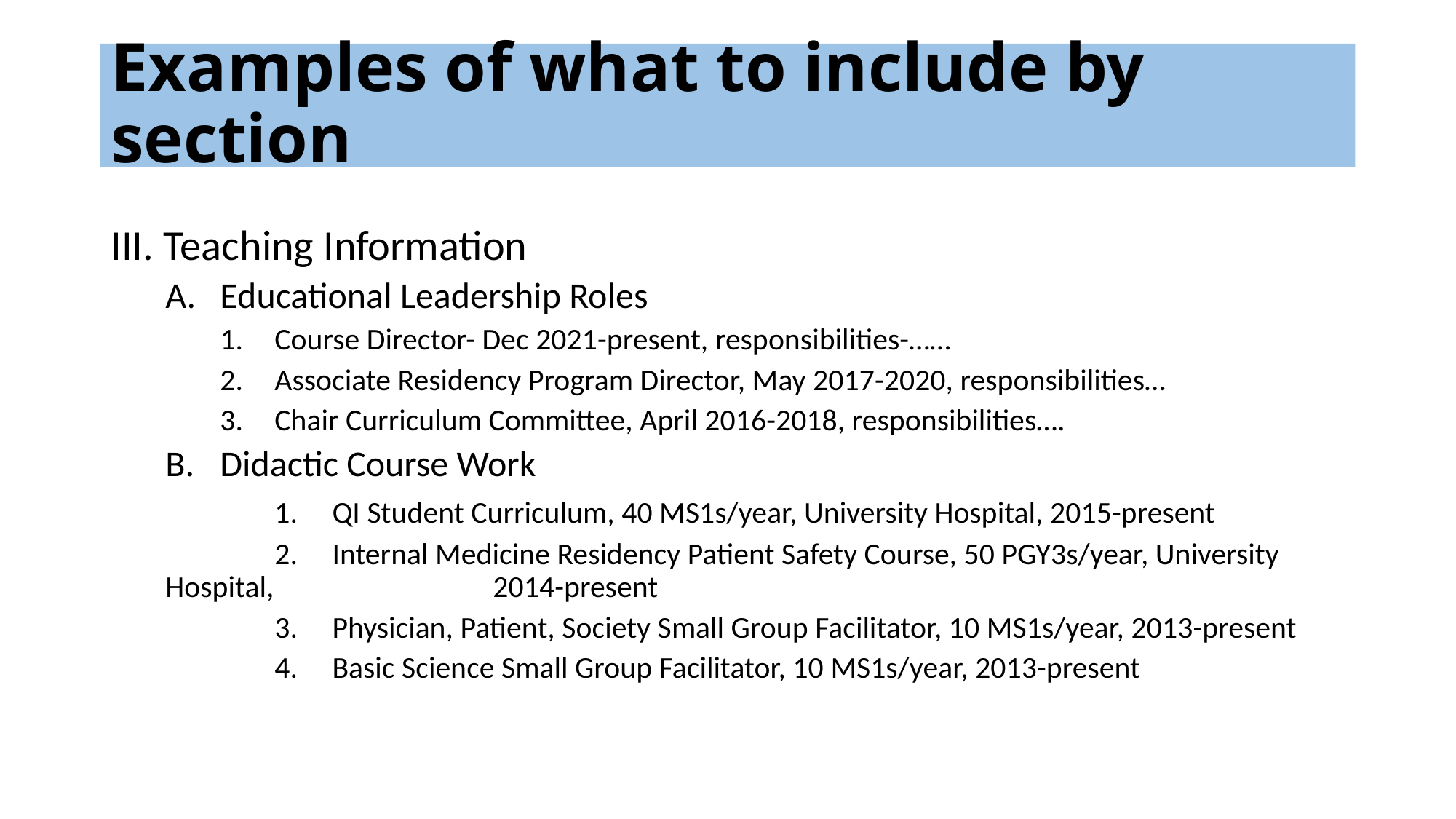

# Examples of what to include by section
III. Teaching Information
Educational Leadership Roles
Course Director- Dec 2021-present, responsibilities-……
Associate Residency Program Director, May 2017-2020, responsibilities…
Chair Curriculum Committee, April 2016-2018, responsibilities….
Didactic Course Work
	1. QI Student Curriculum, 40 MS1s/year, University Hospital, 2015-present
	2. Internal Medicine Residency Patient Safety Course, 50 PGY3s/year, University Hospital, 		2014-present
	3. Physician, Patient, Society Small Group Facilitator, 10 MS1s/year, 2013-present
	4. Basic Science Small Group Facilitator, 10 MS1s/year, 2013-present

## Slide 17
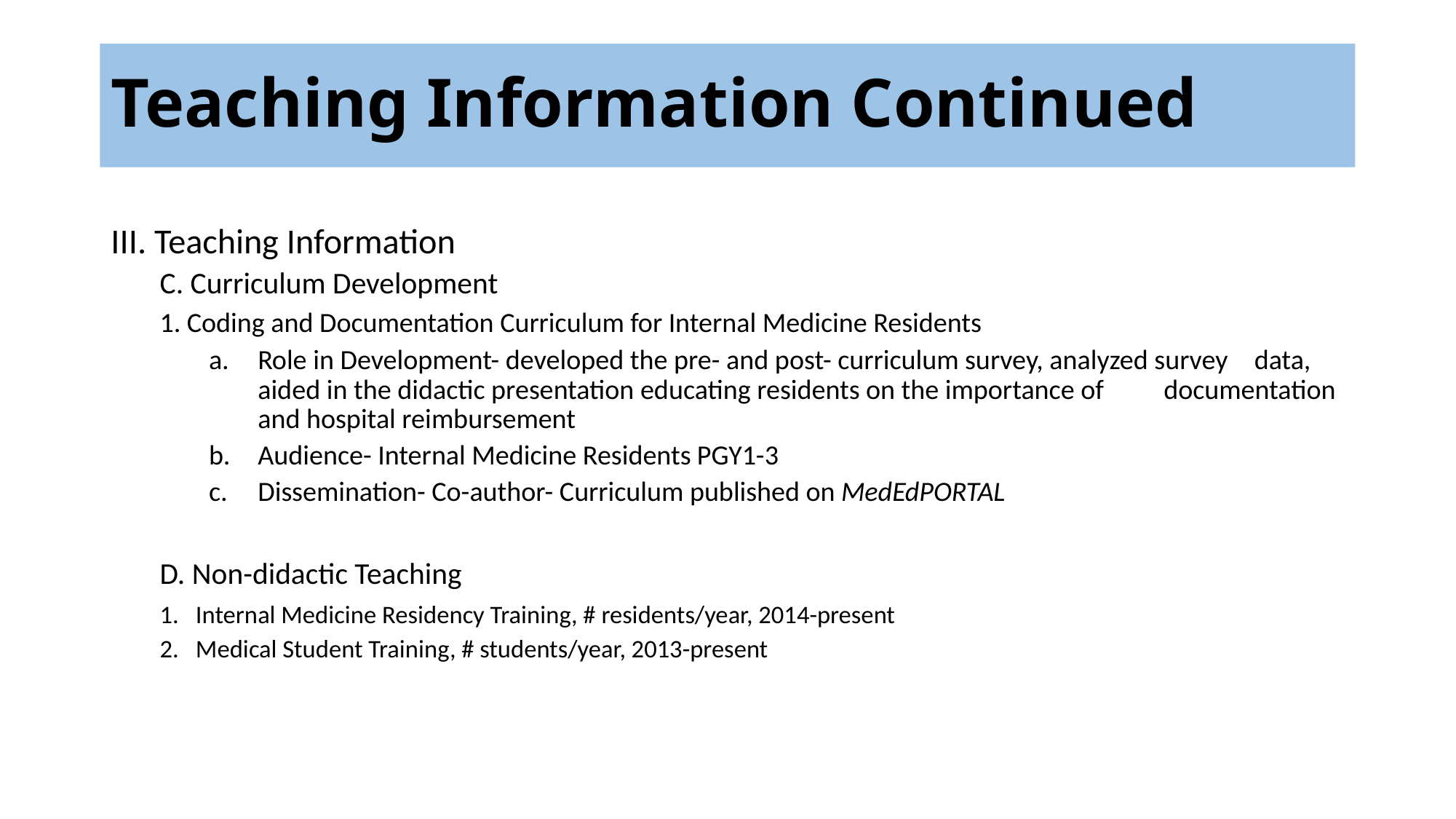

# Teaching Information Continued
III. Teaching Information
C. Curriculum Development
	1. Coding and Documentation Curriculum for Internal Medicine Residents
Role in Development- developed the pre- and post- curriculum survey, analyzed survey 	data, aided in the didactic presentation educating residents on the importance of 	documentation and hospital reimbursement
Audience- Internal Medicine Residents PGY1-3
Dissemination- Co-author- Curriculum published on MedEdPORTAL
D. Non-didactic Teaching
	1. Internal Medicine Residency Training, # residents/year, 2014-present
	2. Medical Student Training, # students/year, 2013-present

## Slide 18
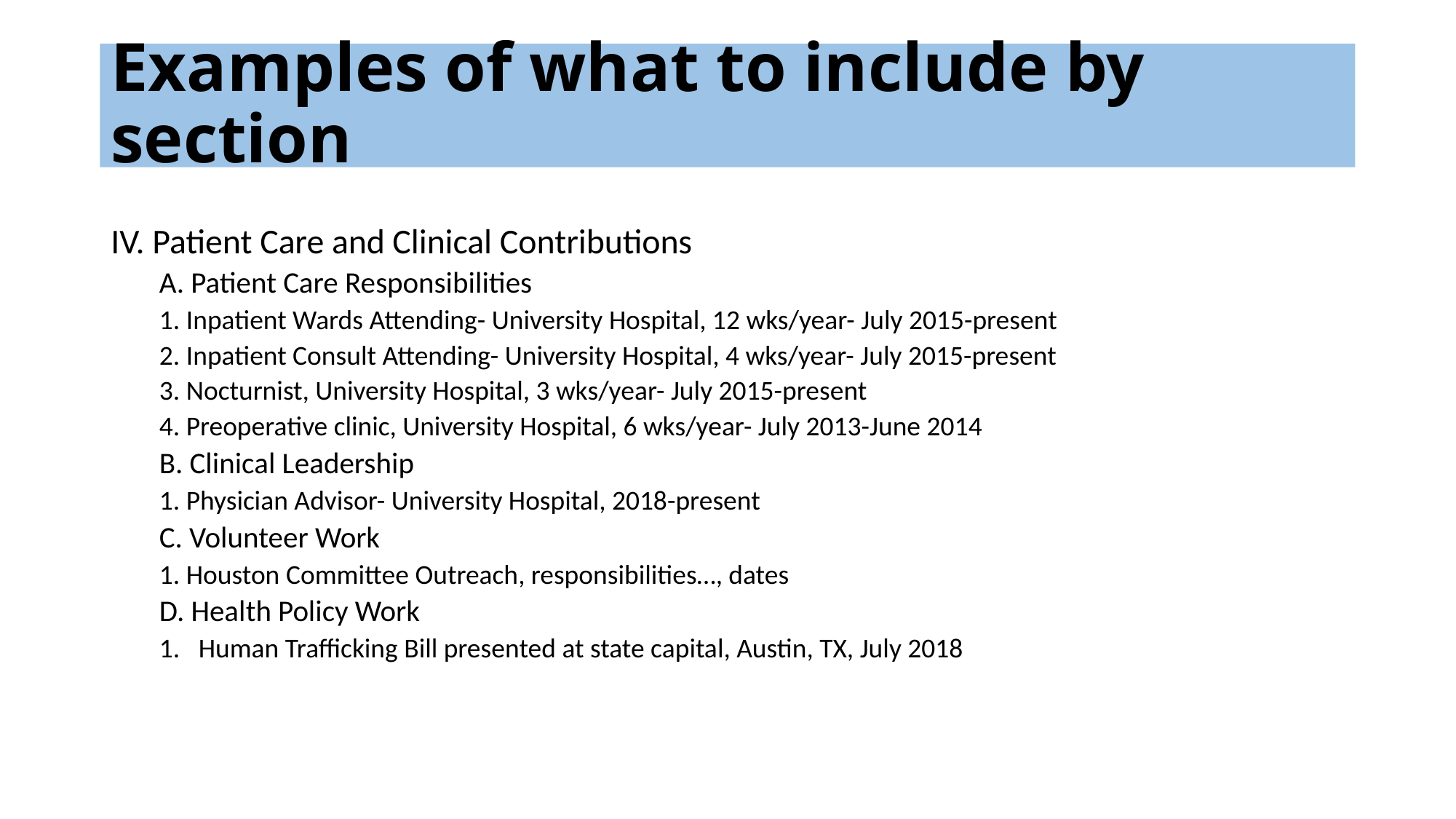

# Examples of what to include by section
IV. Patient Care and Clinical Contributions
A. Patient Care Responsibilities
	1. Inpatient Wards Attending- University Hospital, 12 wks/year- July 2015-present
	2. Inpatient Consult Attending- University Hospital, 4 wks/year- July 2015-present
	3. Nocturnist, University Hospital, 3 wks/year- July 2015-present
	4. Preoperative clinic, University Hospital, 6 wks/year- July 2013-June 2014
B. Clinical Leadership
	1. Physician Advisor- University Hospital, 2018-present
C. Volunteer Work
	1. Houston Committee Outreach, responsibilities…, dates
D. Health Policy Work
	1. Human Trafficking Bill presented at state capital, Austin, TX, July 2018

## Slide 19
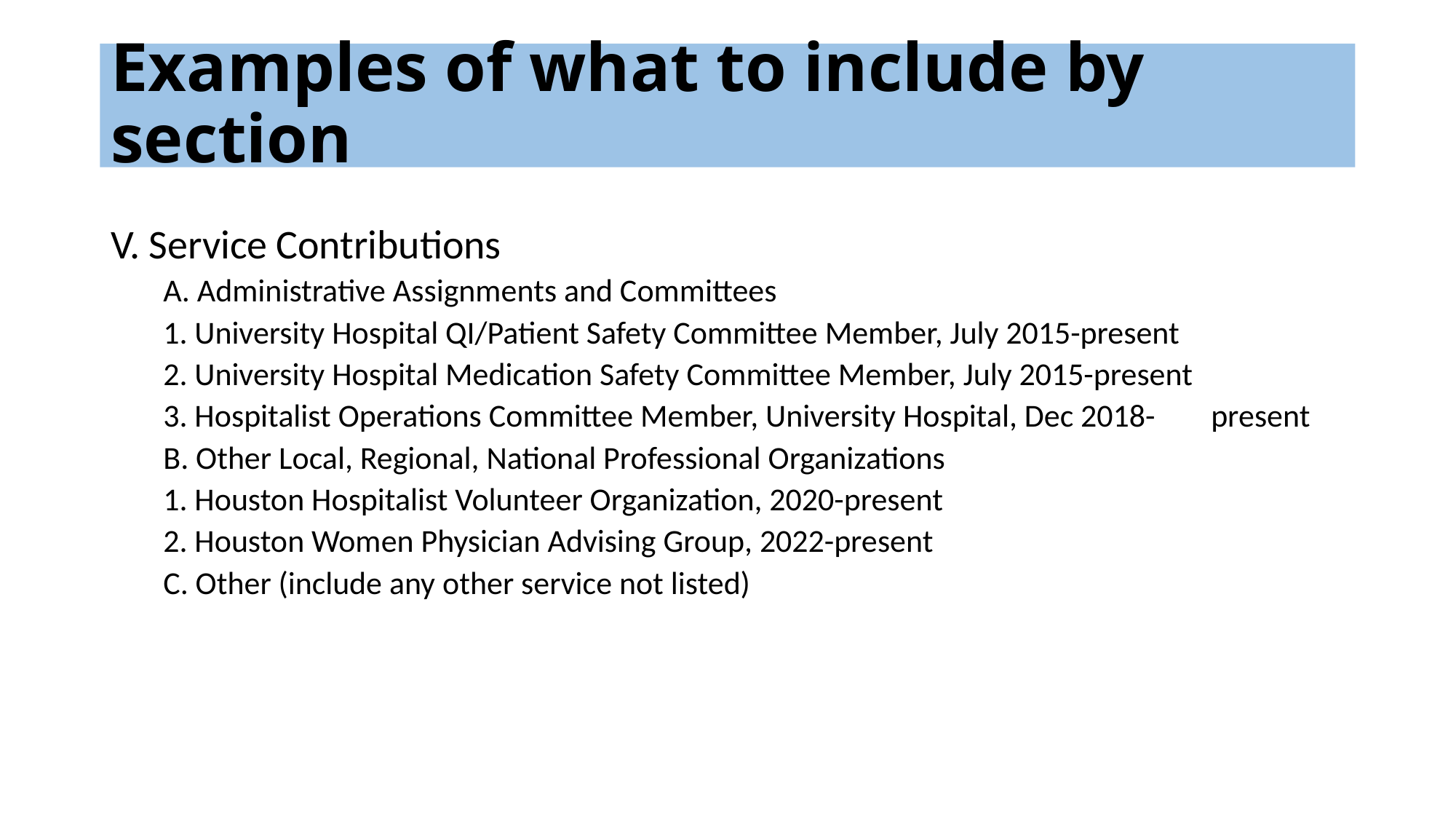

# Examples of what to include by section
V. Service Contributions
A. Administrative Assignments and Committees
	1. University Hospital QI/Patient Safety Committee Member, July 2015-present
	2. University Hospital Medication Safety Committee Member, July 2015-present
	3. Hospitalist Operations Committee Member, University Hospital, Dec 2018-	present
B. Other Local, Regional, National Professional Organizations
	1. Houston Hospitalist Volunteer Organization, 2020-present
	2. Houston Women Physician Advising Group, 2022-present
C. Other (include any other service not listed)

## Slide 20
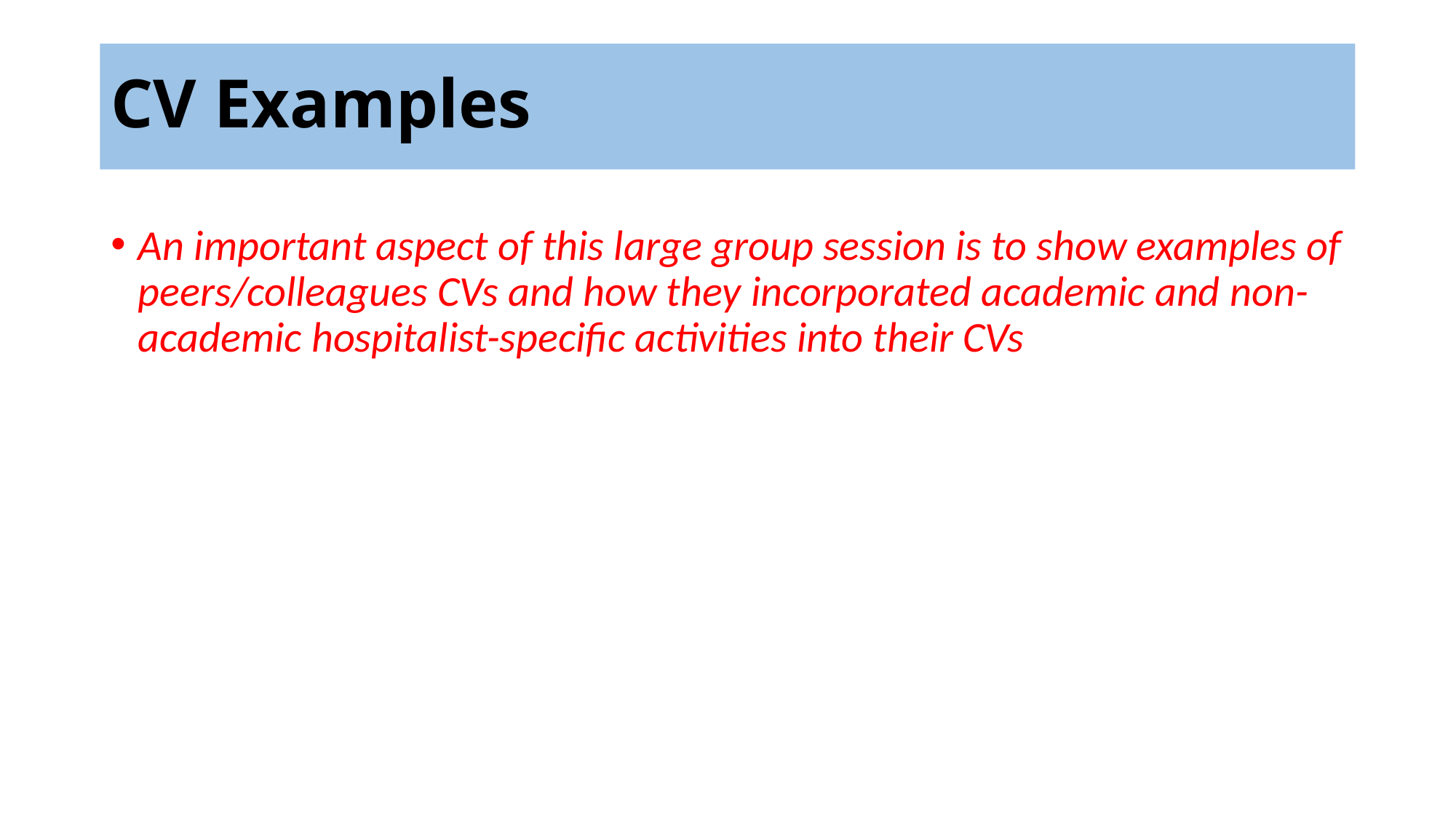

# CV Examples
An important aspect of this large group session is to show examples of peers/colleagues CVs and how they incorporated academic and non-academic hospitalist-specific activities into their CVs

## Slide 21
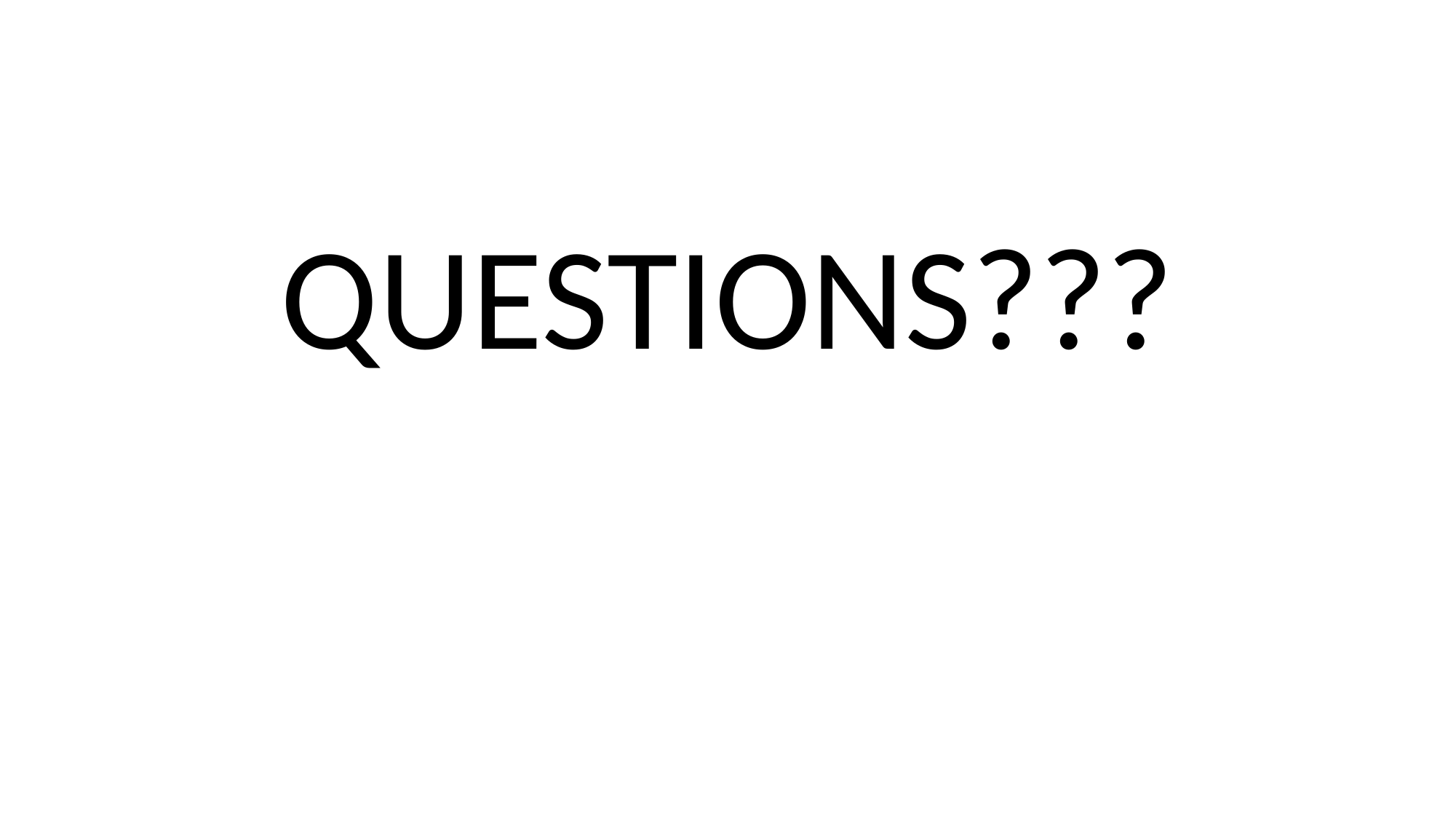

QUESTIONS???

## Slide 22
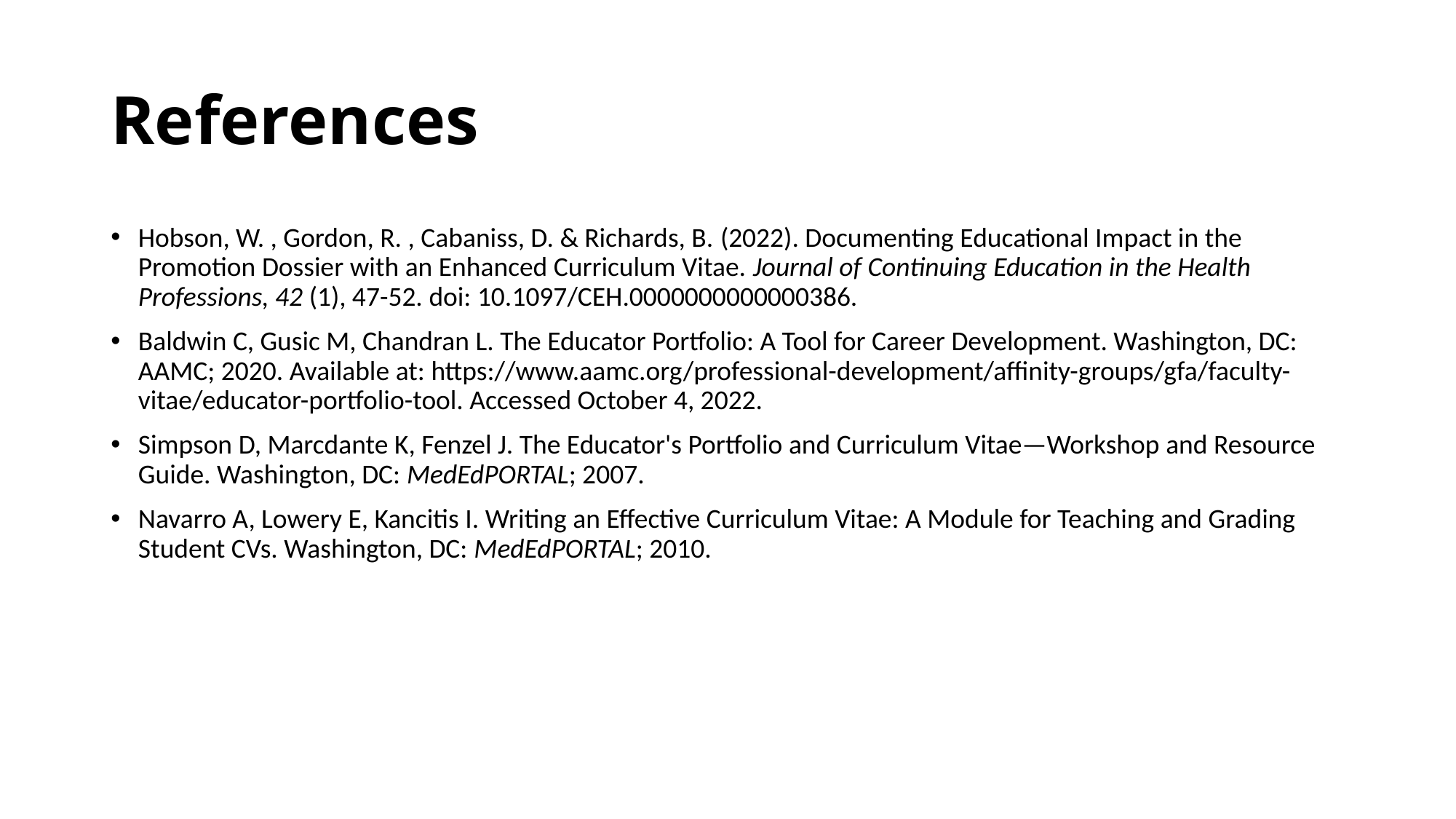

# References
Hobson, W. , Gordon, R. , Cabaniss, D. & Richards, B. (2022). Documenting Educational Impact in the Promotion Dossier with an Enhanced Curriculum Vitae. Journal of Continuing Education in the Health Professions, 42 (1), 47-52. doi: 10.1097/CEH.0000000000000386.
Baldwin C, Gusic M, Chandran L. The Educator Portfolio: A Tool for Career Development. Washington, DC: AAMC; 2020. Available at: https://www.aamc.org/professional-development/affinity-groups/gfa/faculty-vitae/educator-portfolio-tool. Accessed October 4, 2022.
Simpson D, Marcdante K, Fenzel J. The Educator's Portfolio and Curriculum Vitae—Workshop and Resource Guide. Washington, DC: MedEdPORTAL; 2007.
Navarro A, Lowery E, Kancitis I. Writing an Effective Curriculum Vitae: A Module for Teaching and Grading Student CVs. Washington, DC: MedEdPORTAL; 2010.
