# Supplementary material for: A Facilitated Peer Mentoring Program With a Dedicated Curriculum to Foster Career Advancement of Academic Hospitalists
Source: MedEdPORTAL. 2023 Dec 8;19:11366. doi: 10.15766/mep_2374-8265.11366 (PMC10704005; doi:10.15766/mep_2374-8265.11366)
Supplement: Supplementary file 1 — Preprogram Survey.docxPostprogram Survey.docxLarge-Group Session 1.pptxLarge-Group Session 2.pptxLarge-Group Session 3.pptxLarge-Group Session 4.pptxSmall-Group Session 1 Facilitator Guide.docxSmall-Group Session 2 Facilitator Guide.docxSmall-Group Session 3 Facilitator Guide.docx [file mep_2374-8265.11366-s001.zip › E. Large-Group Session 3.pptx]

## Slide 1
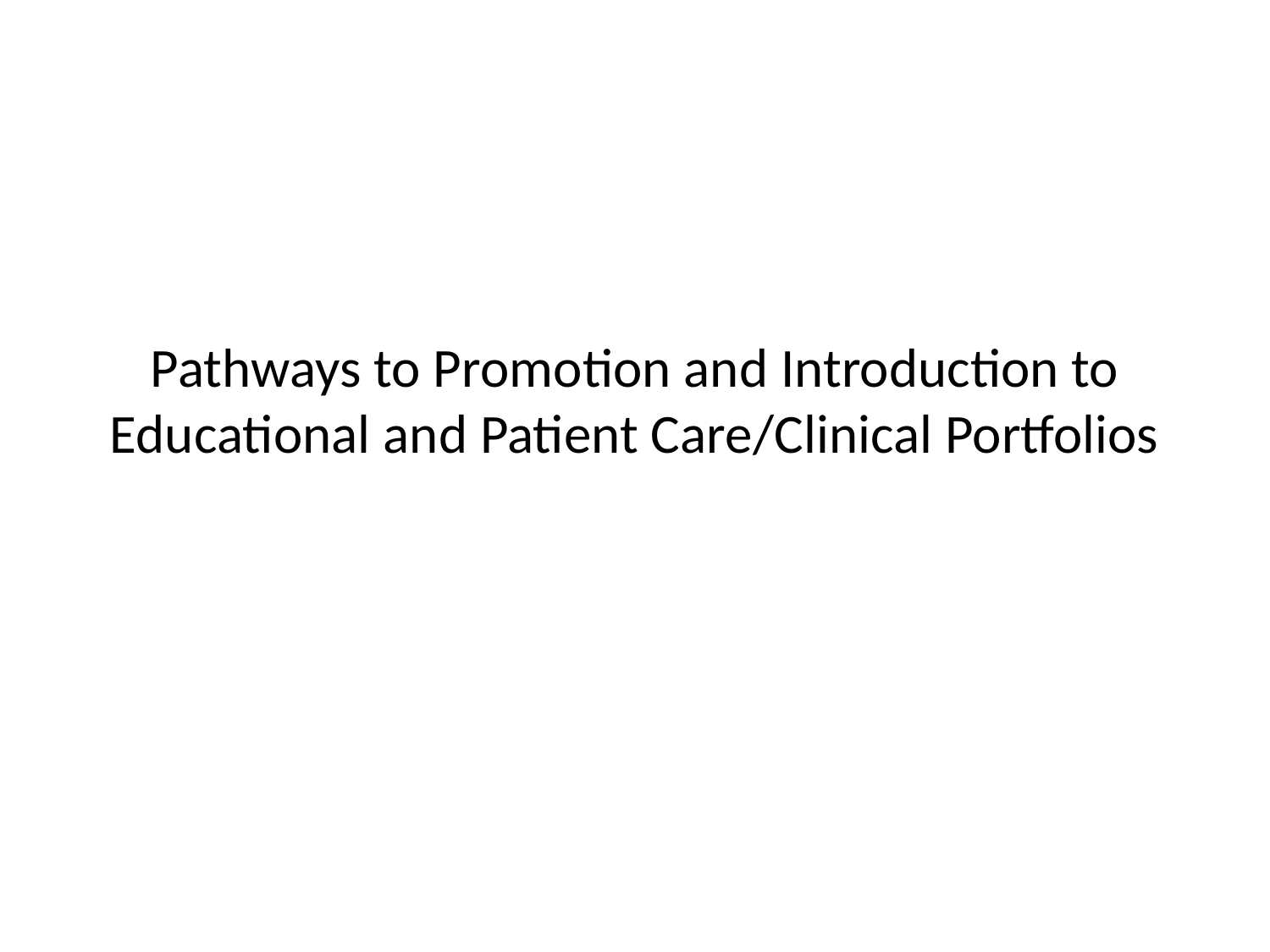

# Pathways to Promotion and Introduction to Educational and Patient Care/Clinical Portfolios

## Slide 2
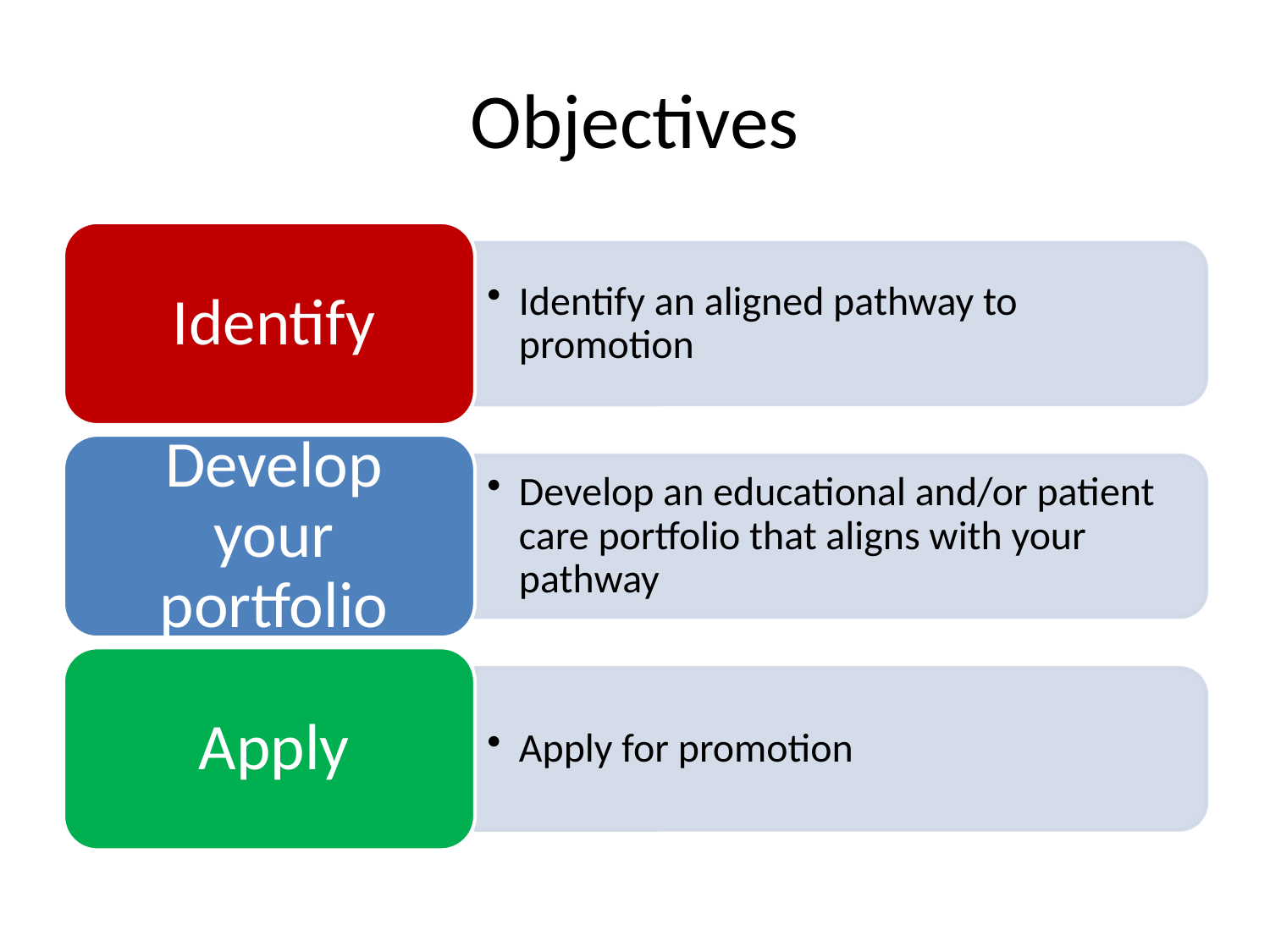

# Objectives

## Slide 3
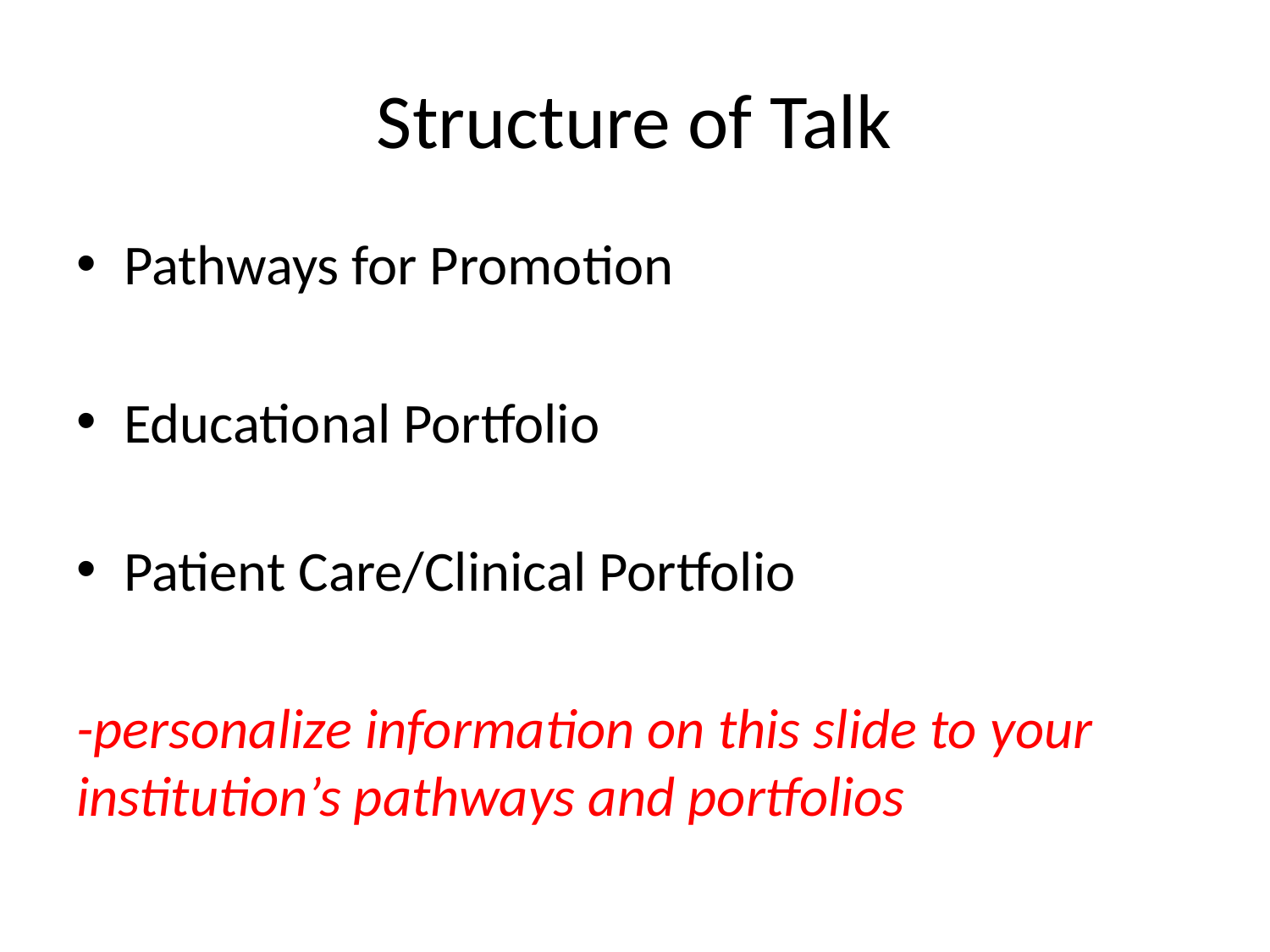

# Structure of Talk
Pathways for Promotion
Educational Portfolio
Patient Care/Clinical Portfolio
-personalize information on this slide to your institution’s pathways and portfolios

## Slide 4
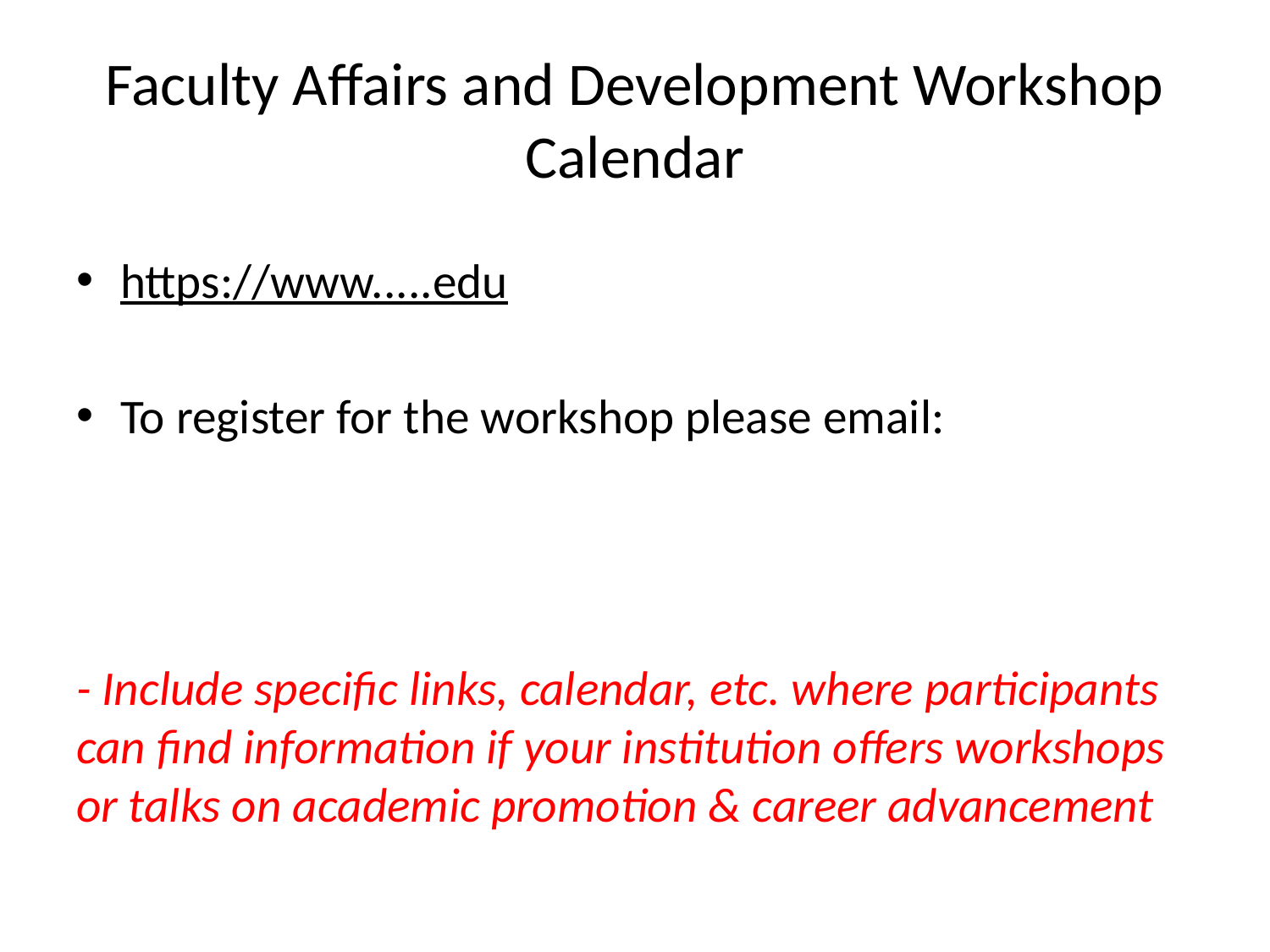

# Faculty Affairs and Development Workshop Calendar
https://www.....edu
To register for the workshop please email:
- Include specific links, calendar, etc. where participants can find information if your institution offers workshops or talks on academic promotion & career advancement

## Slide 5
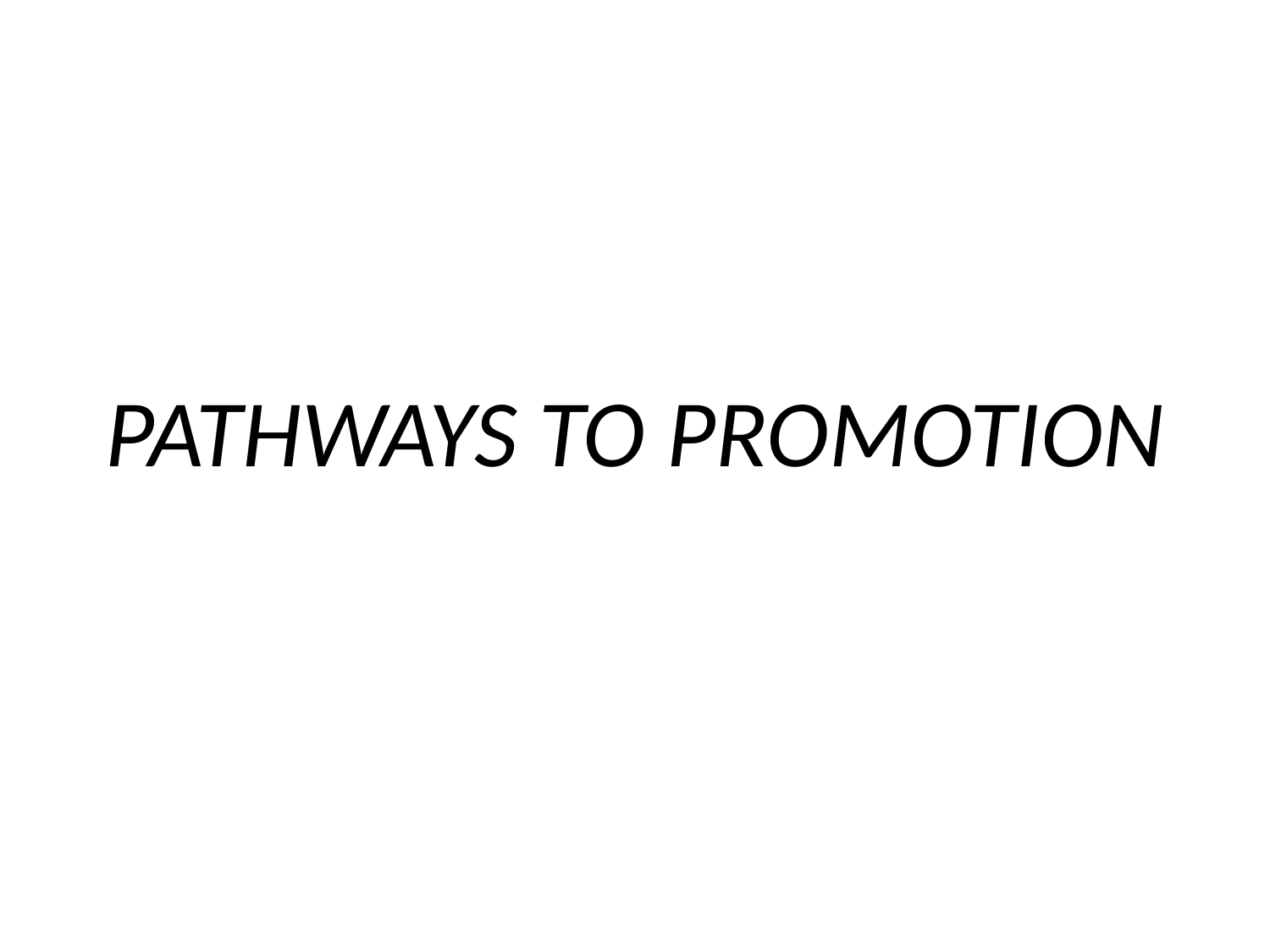

# PATHWAYS TO PROMOTION

## Slide 6
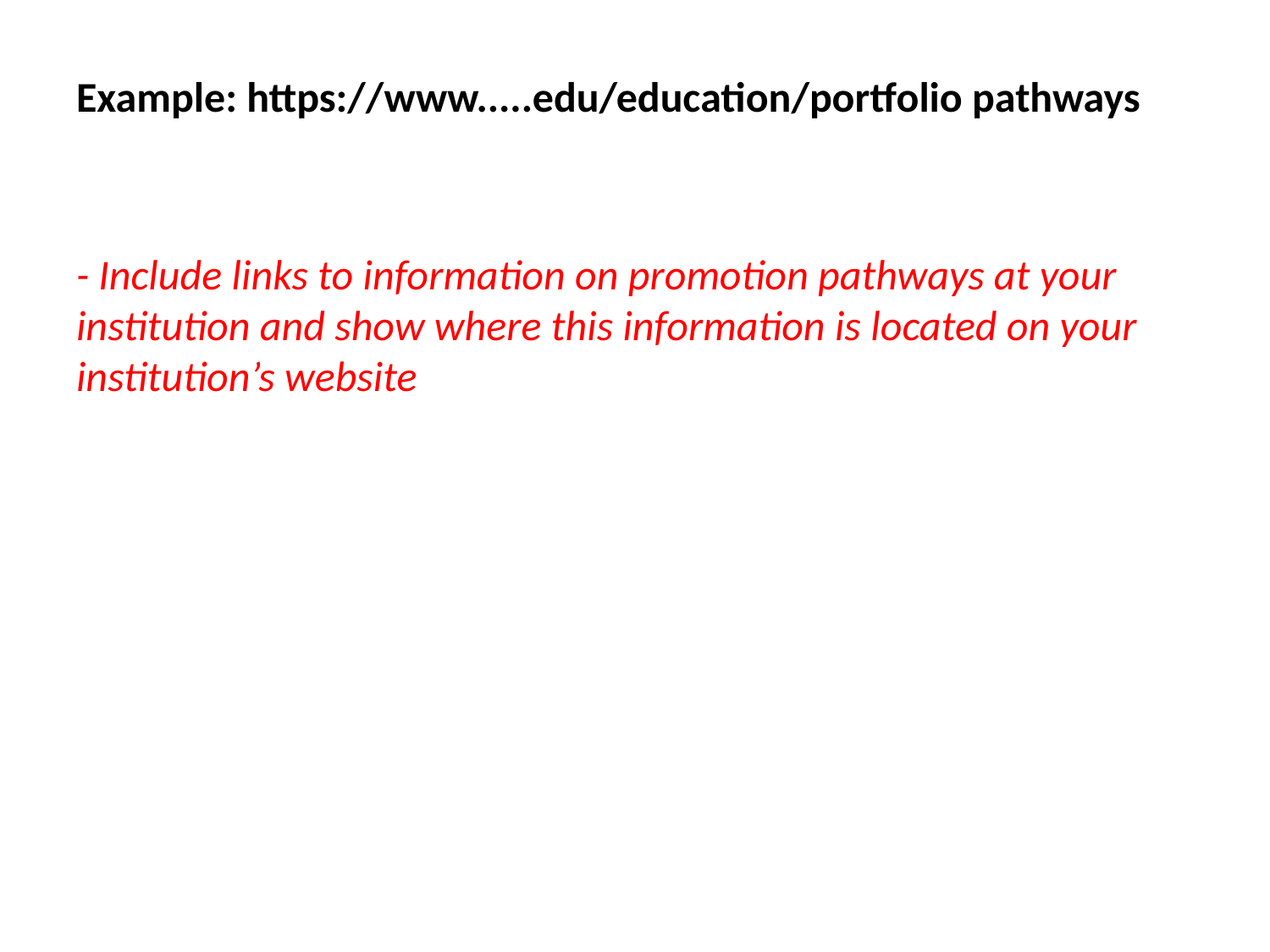

Example: https://www.....edu/education/portfolio pathways
- Include links to information on promotion pathways at your institution and show where this information is located on your institution’s website

## Slide 7
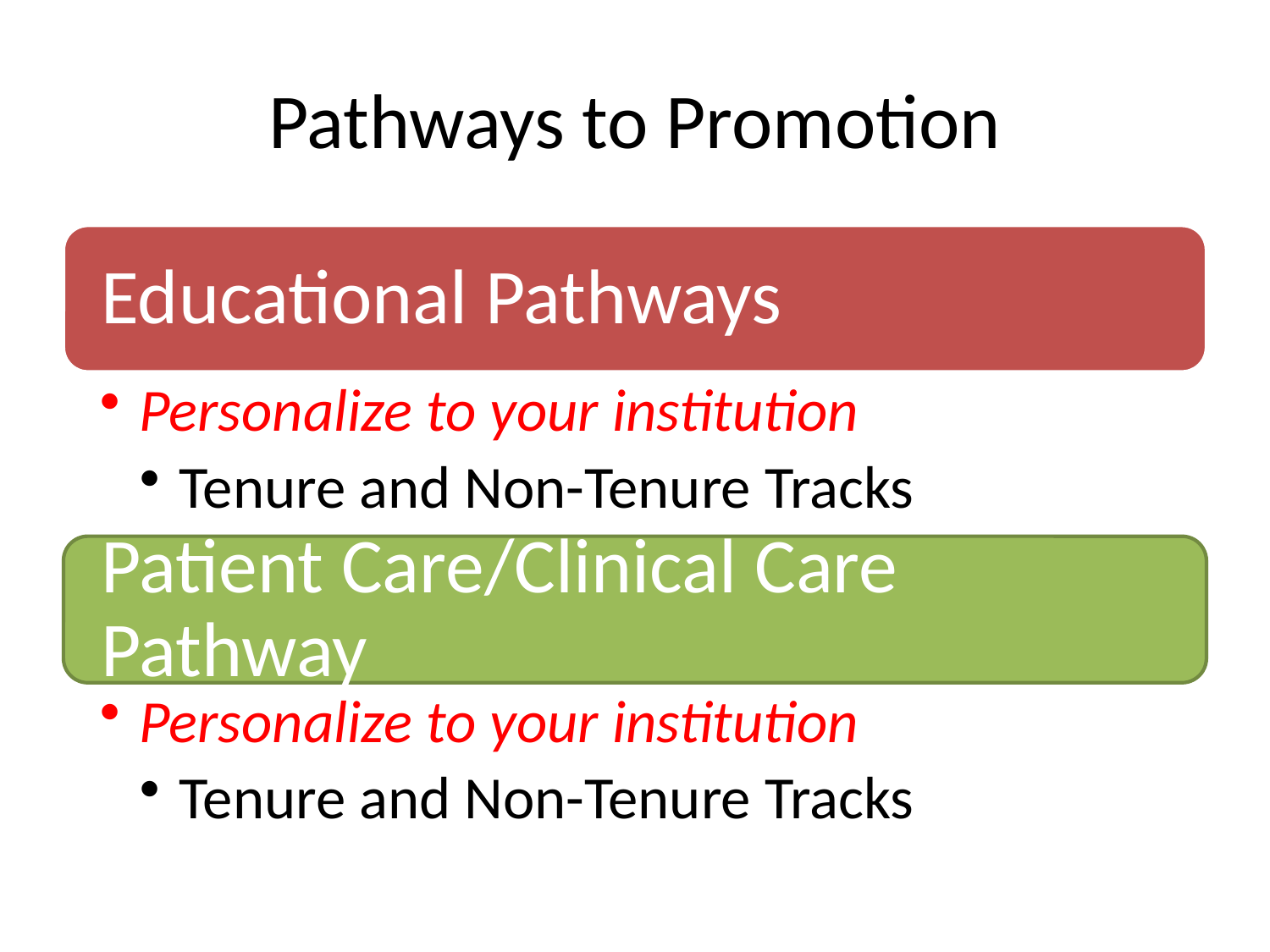

# Pathways to Promotion

## Slide 8
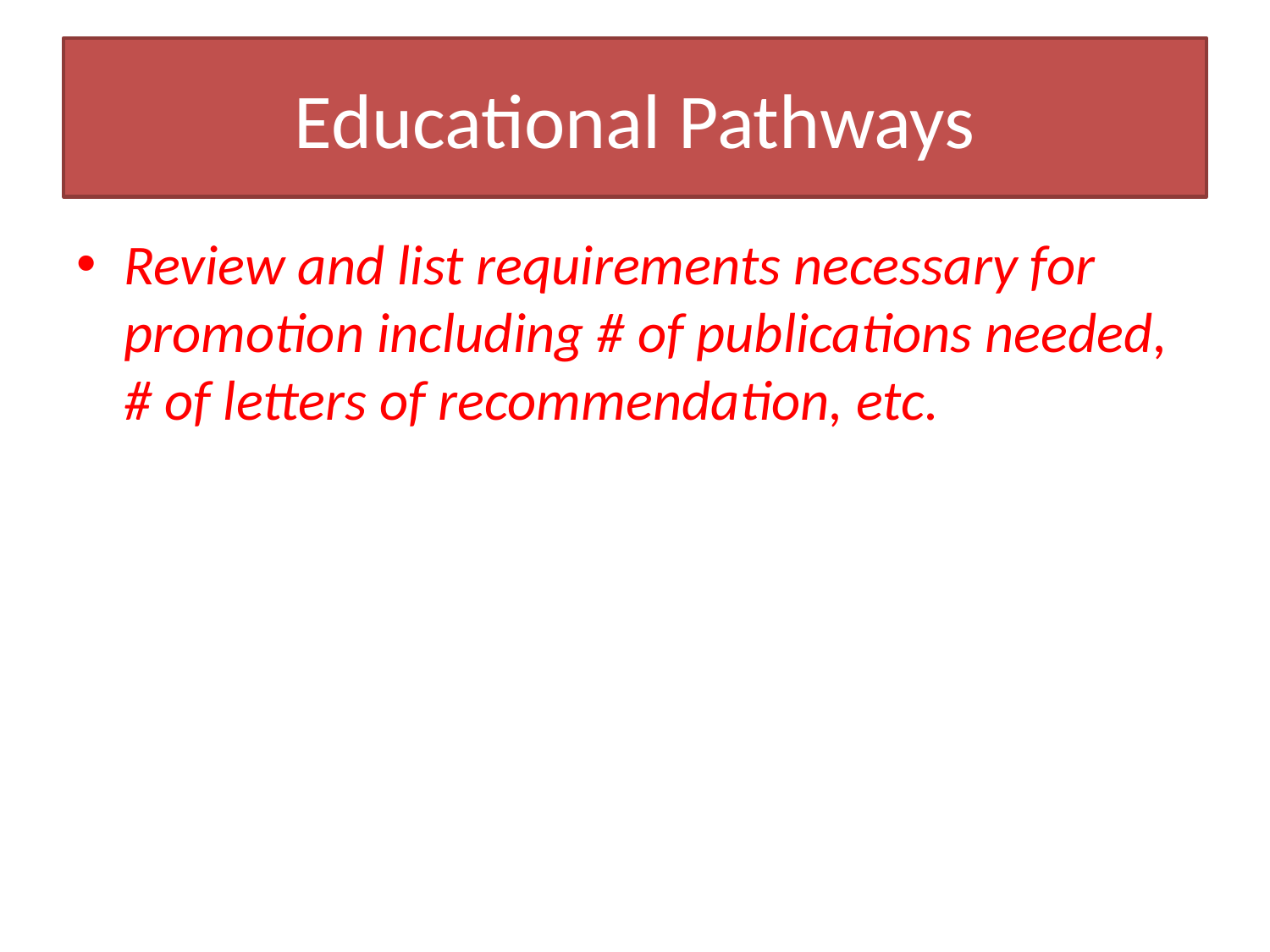

# Educational Pathways
Review and list requirements necessary for promotion including # of publications needed, # of letters of recommendation, etc.

## Slide 9
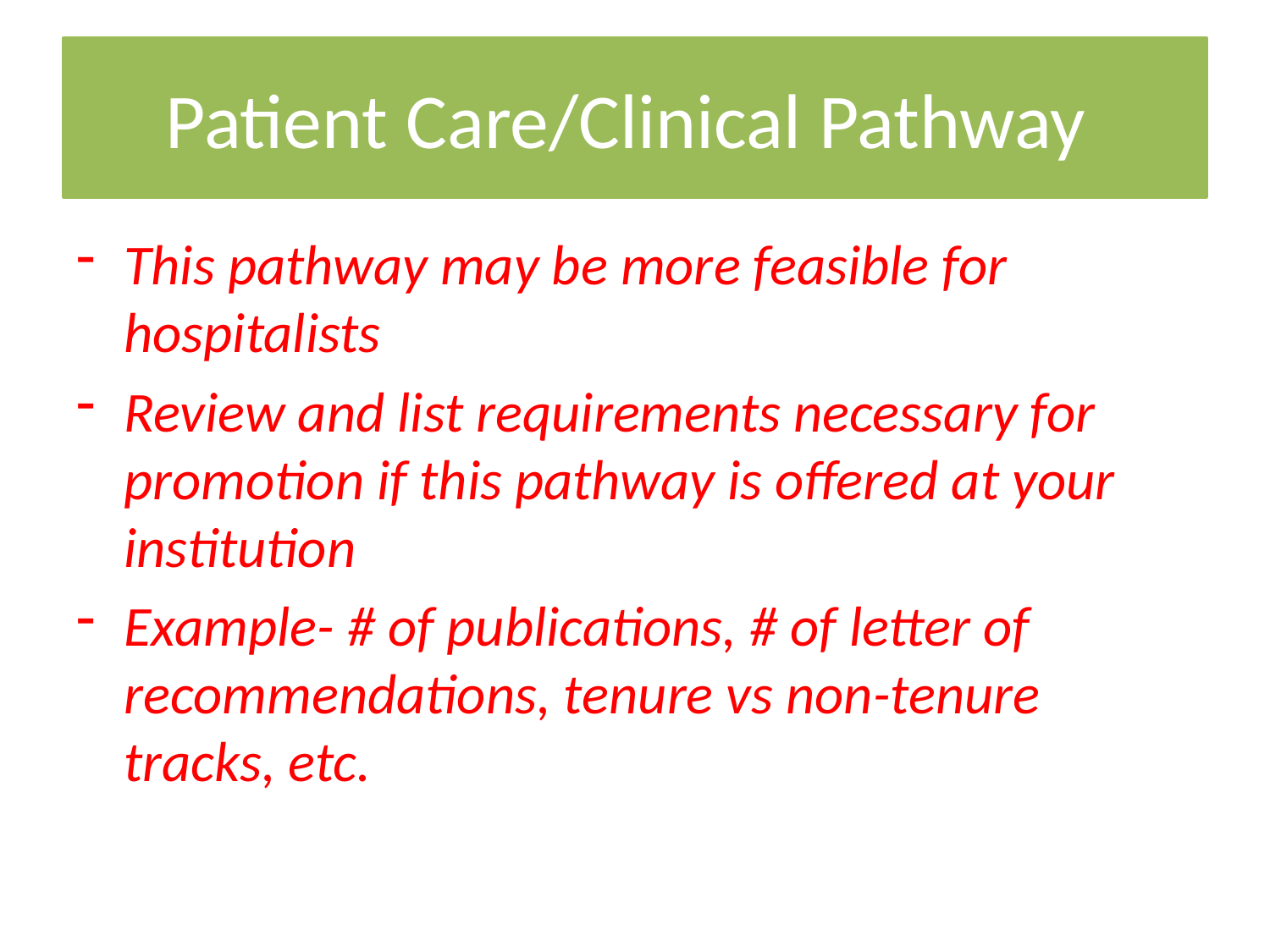

# Patient Care/Clinical Pathway
This pathway may be more feasible for hospitalists
Review and list requirements necessary for promotion if this pathway is offered at your institution
Example- # of publications, # of letter of recommendations, tenure vs non-tenure tracks, etc.

## Slide 10
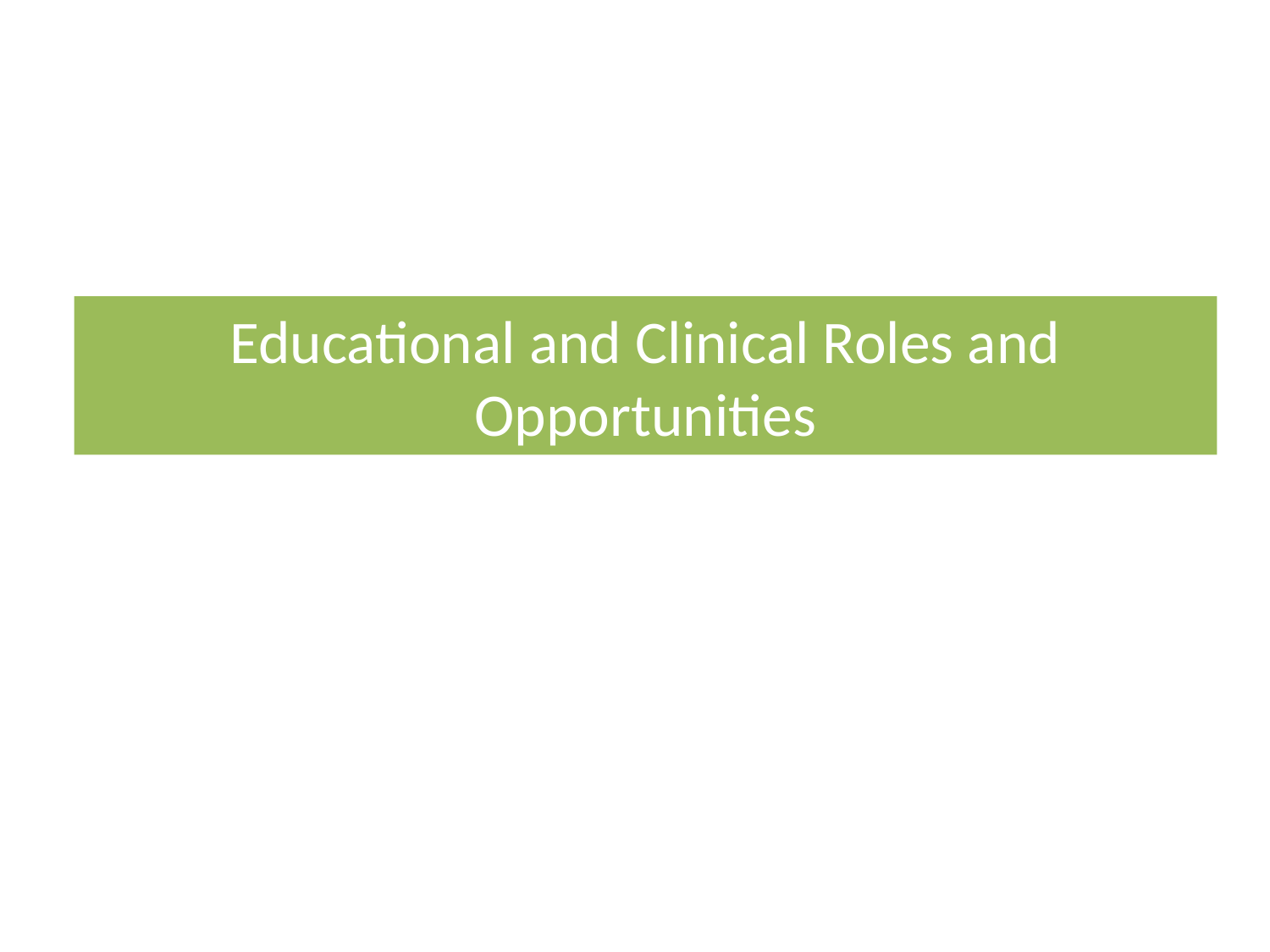

# Educational and Clinical Roles and Opportunities

## Slide 11
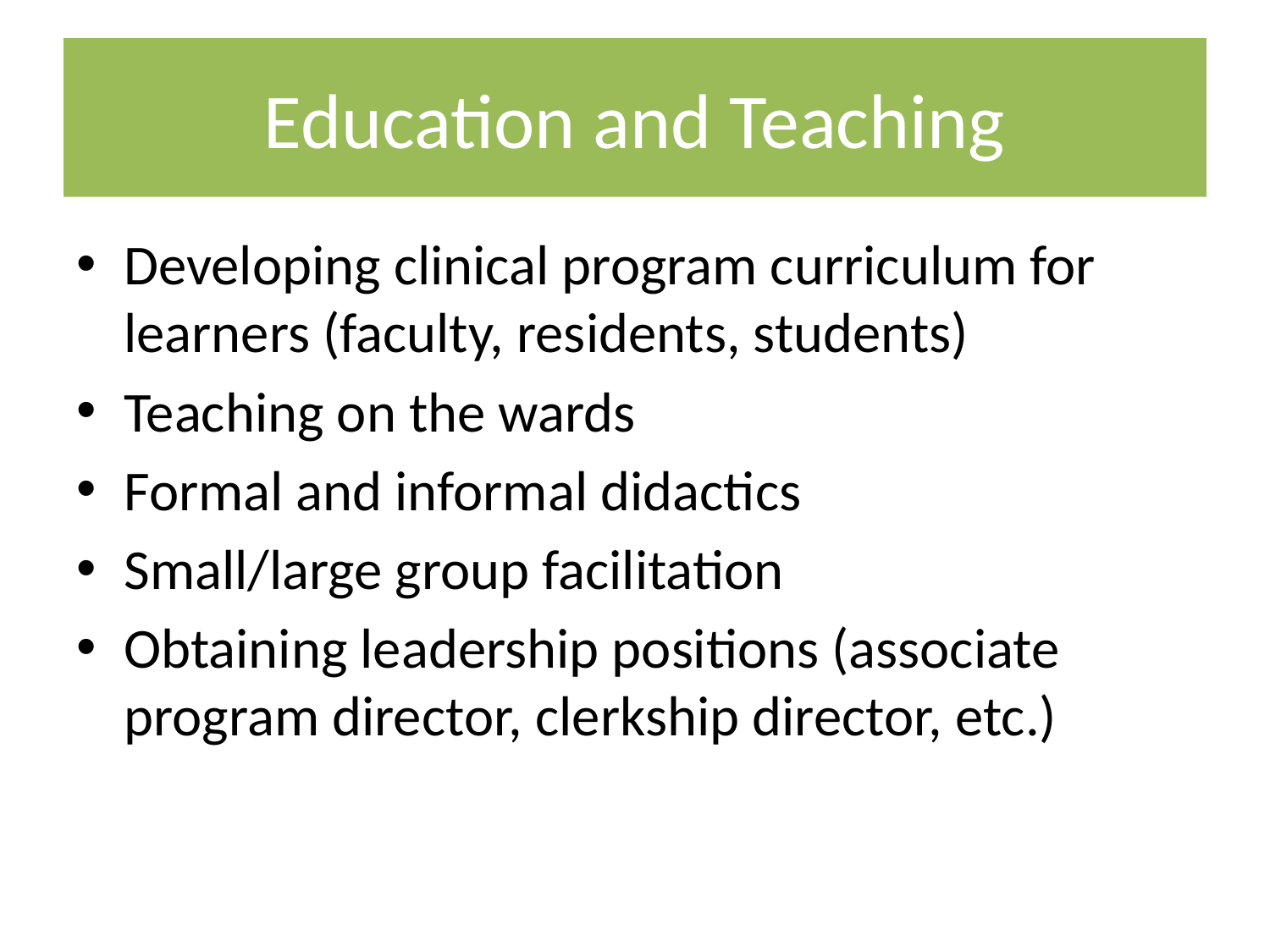

# Education and Teaching
Developing clinical program curriculum for learners (faculty, residents, students)
Teaching on the wards
Formal and informal didactics
Small/large group facilitation
Obtaining leadership positions (associate program director, clerkship director, etc.)

## Slide 12
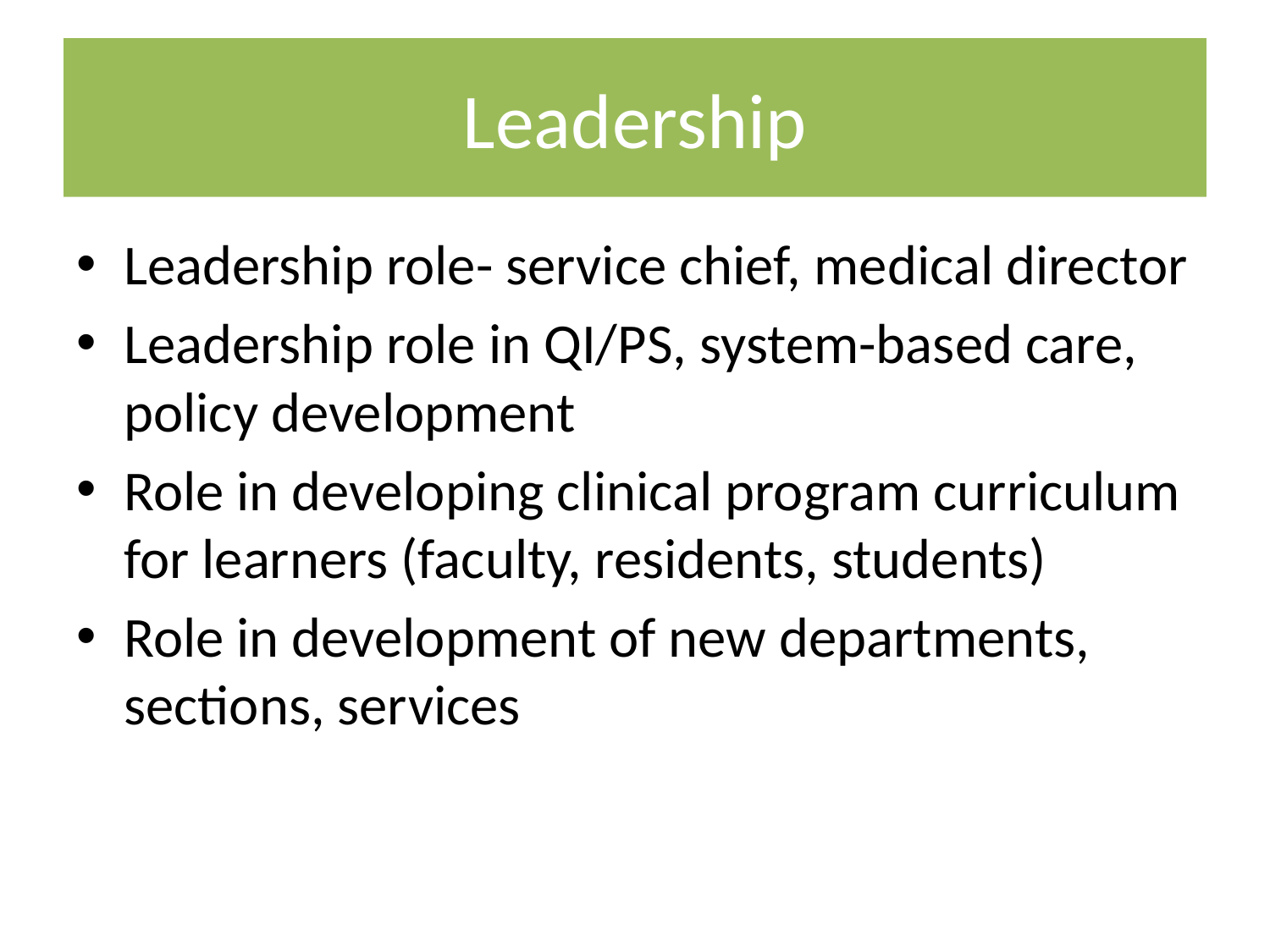

# Leadership
Leadership role- service chief, medical director
Leadership role in QI/PS, system-based care, policy development
Role in developing clinical program curriculum for learners (faculty, residents, students)
Role in development of new departments, sections, services

## Slide 13
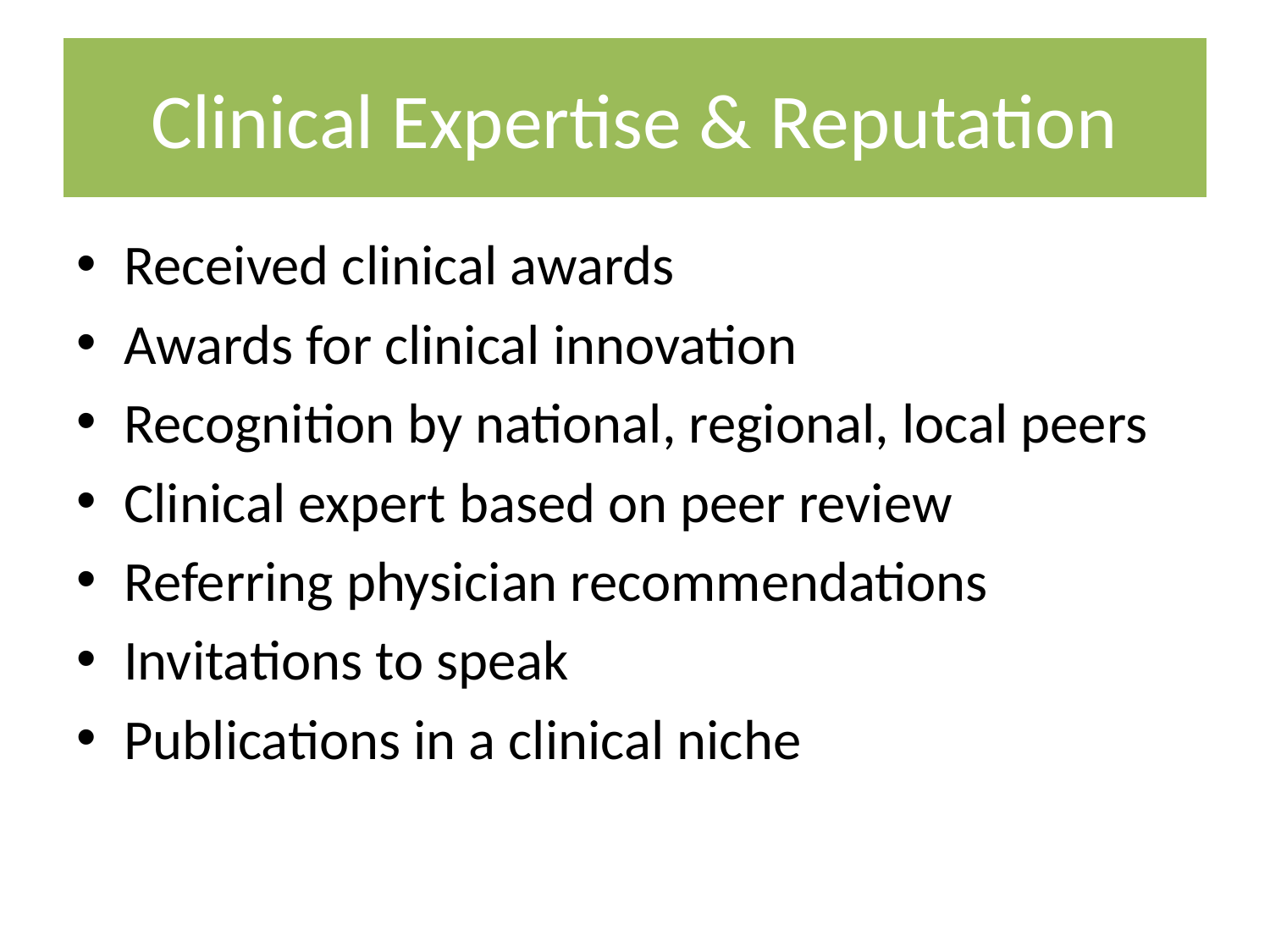

# Clinical Expertise & Reputation
Received clinical awards
Awards for clinical innovation
Recognition by national, regional, local peers
Clinical expert based on peer review
Referring physician recommendations
Invitations to speak
Publications in a clinical niche

## Slide 14
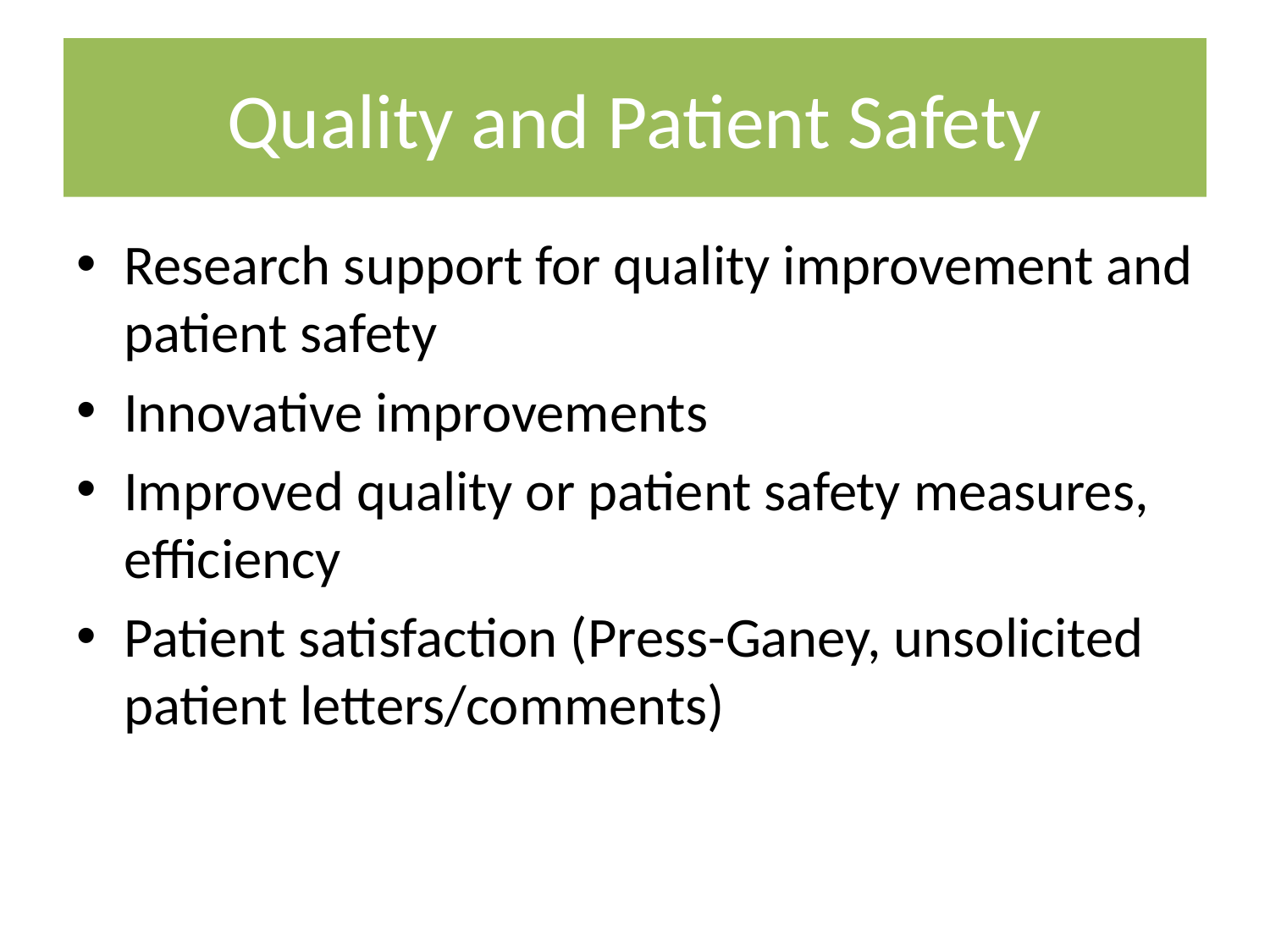

# Quality and Patient Safety
Research support for quality improvement and patient safety
Innovative improvements
Improved quality or patient safety measures, efficiency
Patient satisfaction (Press-Ganey, unsolicited patient letters/comments)

## Slide 15
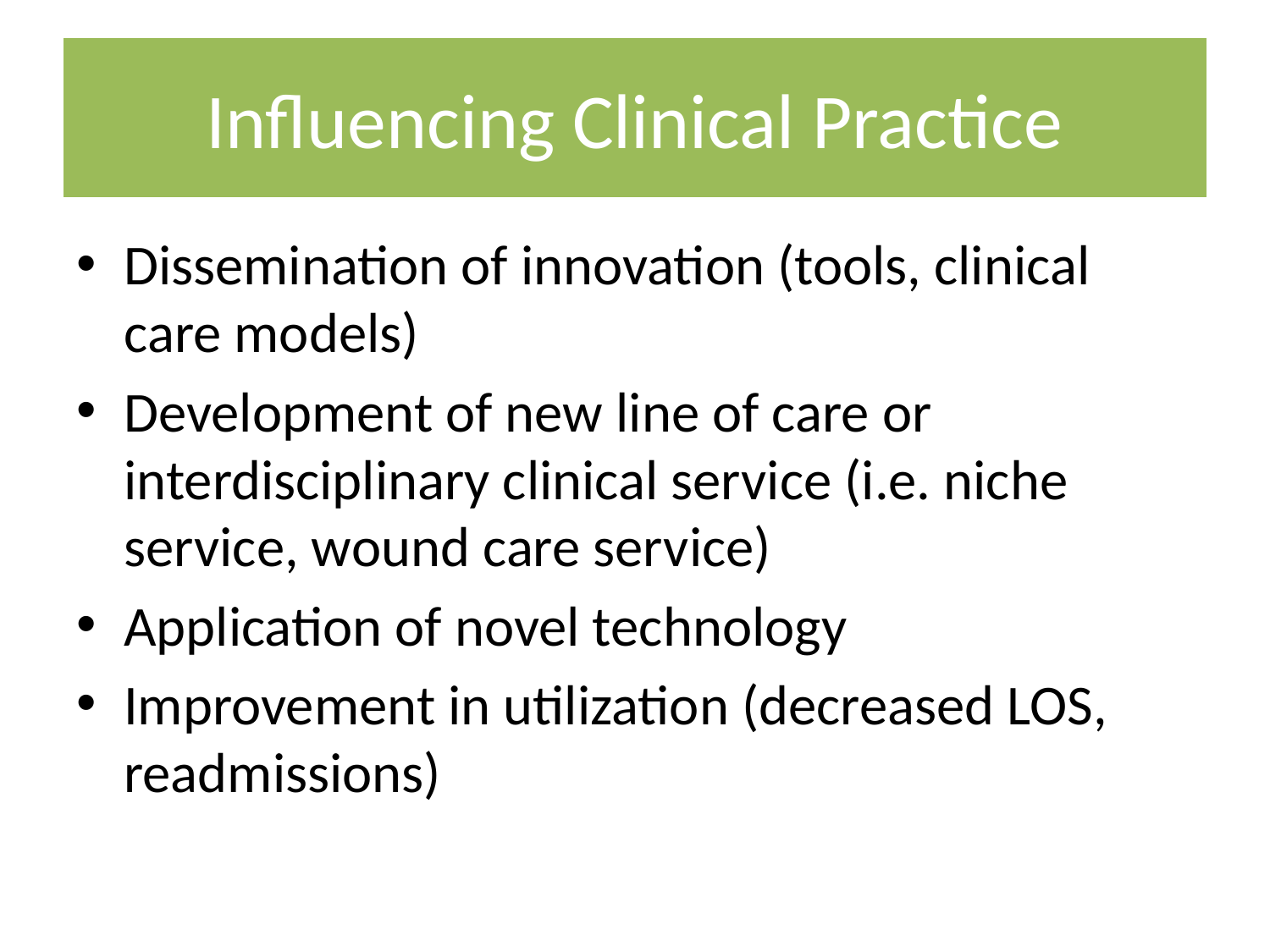

# Influencing Clinical Practice
Dissemination of innovation (tools, clinical care models)
Development of new line of care or interdisciplinary clinical service (i.e. niche service, wound care service)
Application of novel technology
Improvement in utilization (decreased LOS, readmissions)

## Slide 16
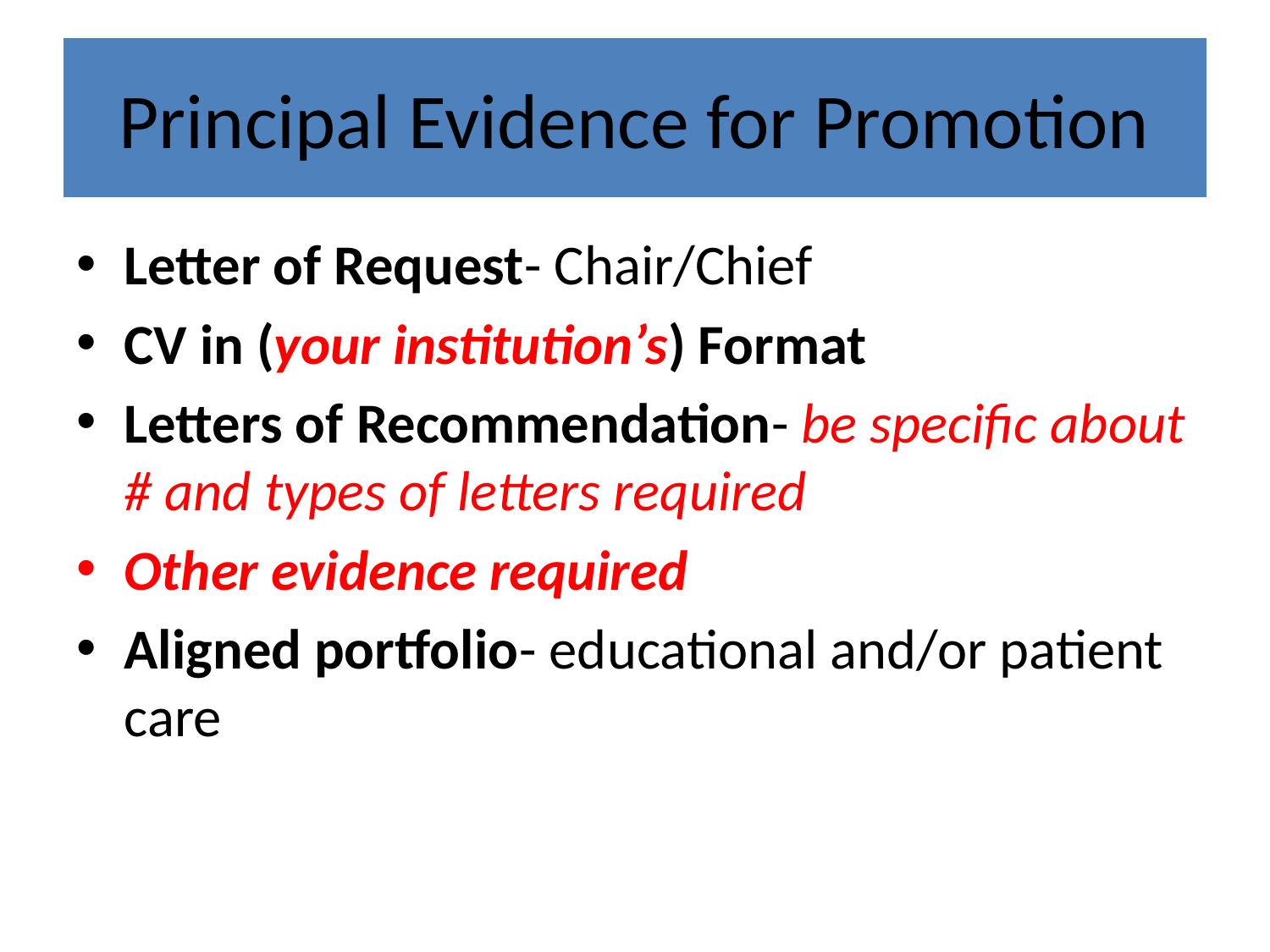

# Principal Evidence for Promotion
Letter of Request- Chair/Chief
CV in (your institution’s) Format
Letters of Recommendation- be specific about # and types of letters required
Other evidence required
Aligned portfolio- educational and/or patient care

## Slide 17
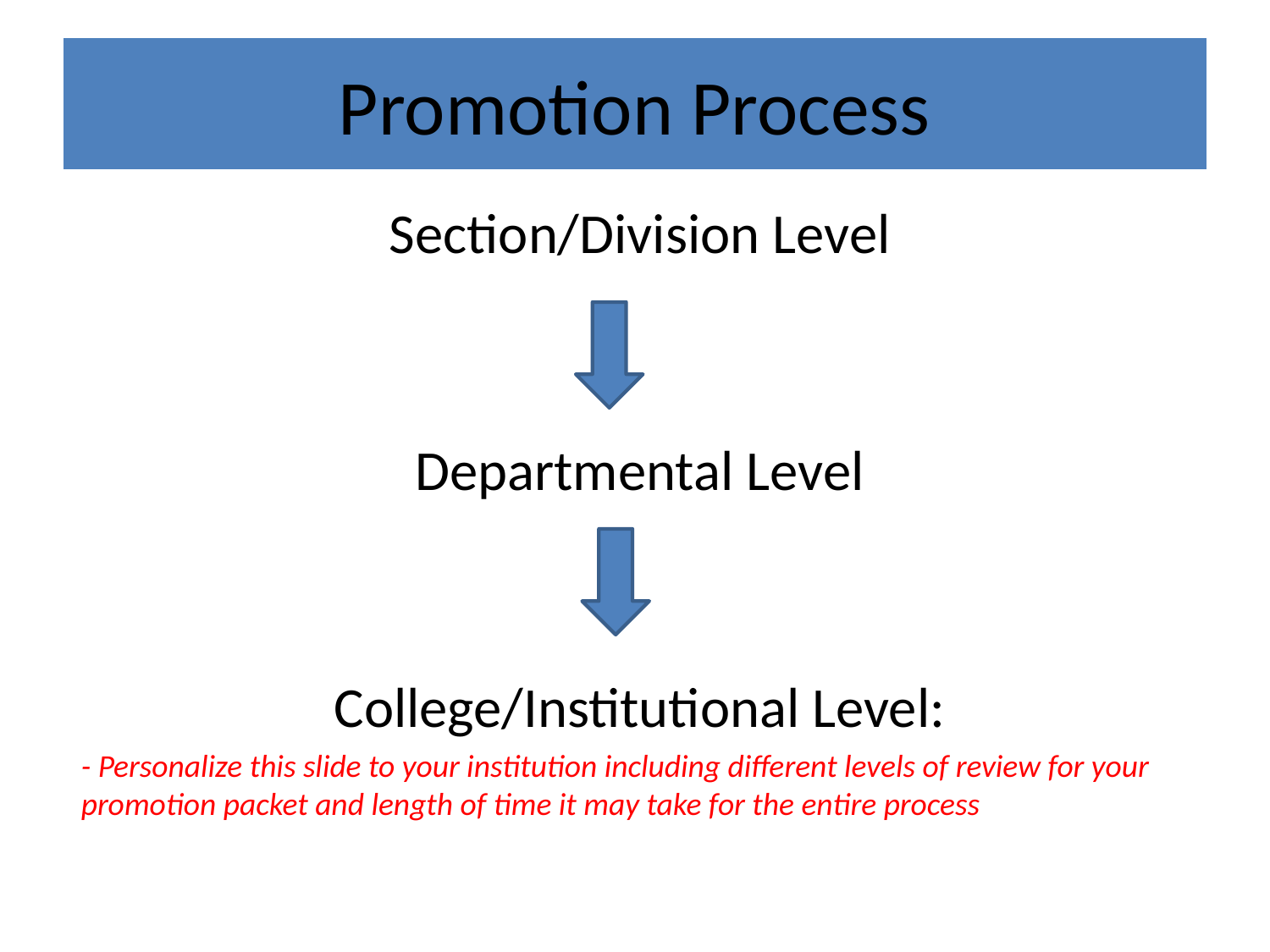

# Promotion Process
Section/Division Level
Departmental Level
College/Institutional Level:
- Personalize this slide to your institution including different levels of review for your promotion packet and length of time it may take for the entire process

## Slide 18
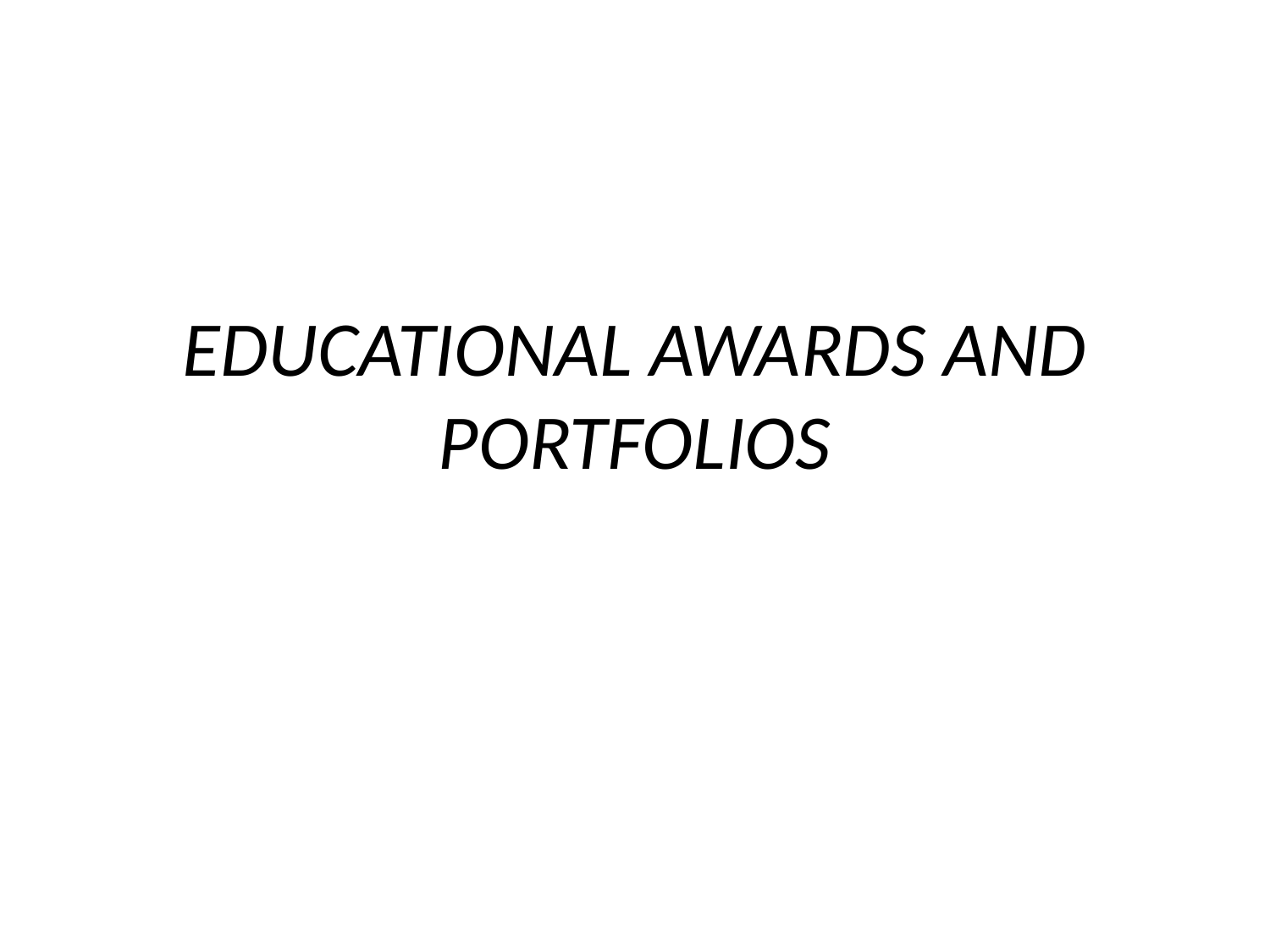

# EDUCATIONAL AWARDS AND PORTFOLIOS

## Slide 19
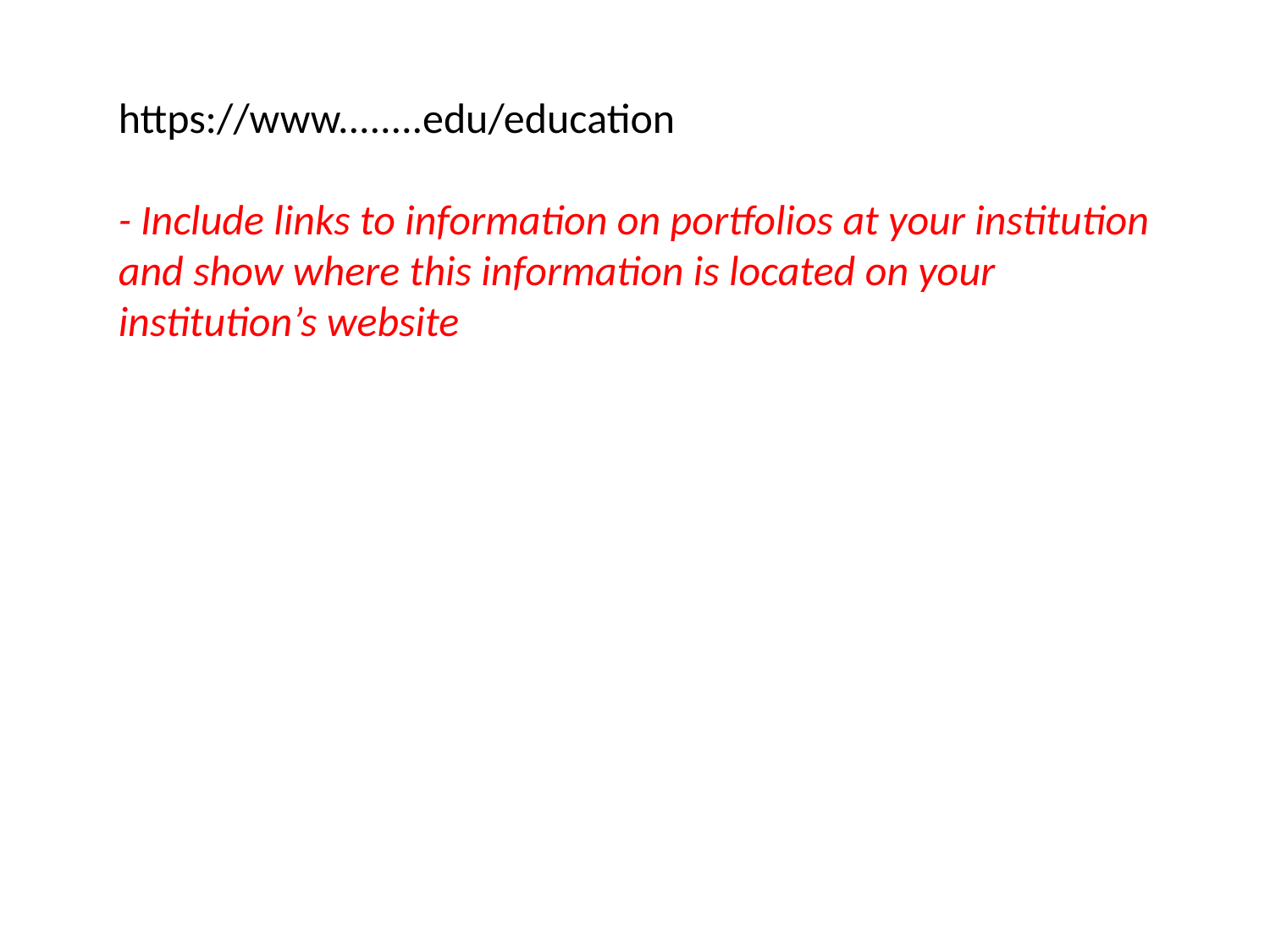

https://www........edu/education
- Include links to information on portfolios at your institution and show where this information is located on your institution’s website

## Slide 20
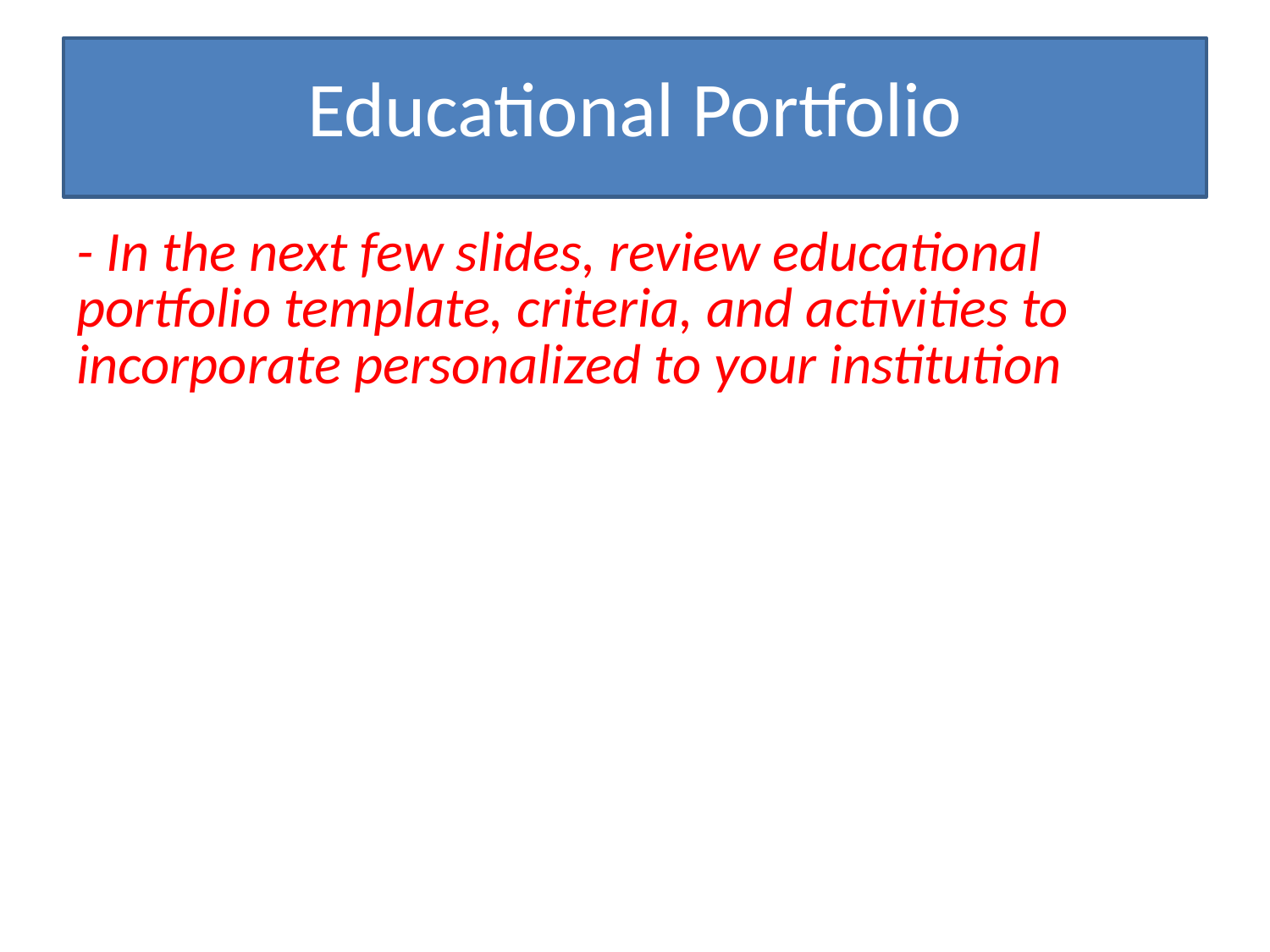

# Educational Portfolio
- In the next few slides, review educational portfolio template, criteria, and activities to incorporate personalized to your institution

## Slide 21
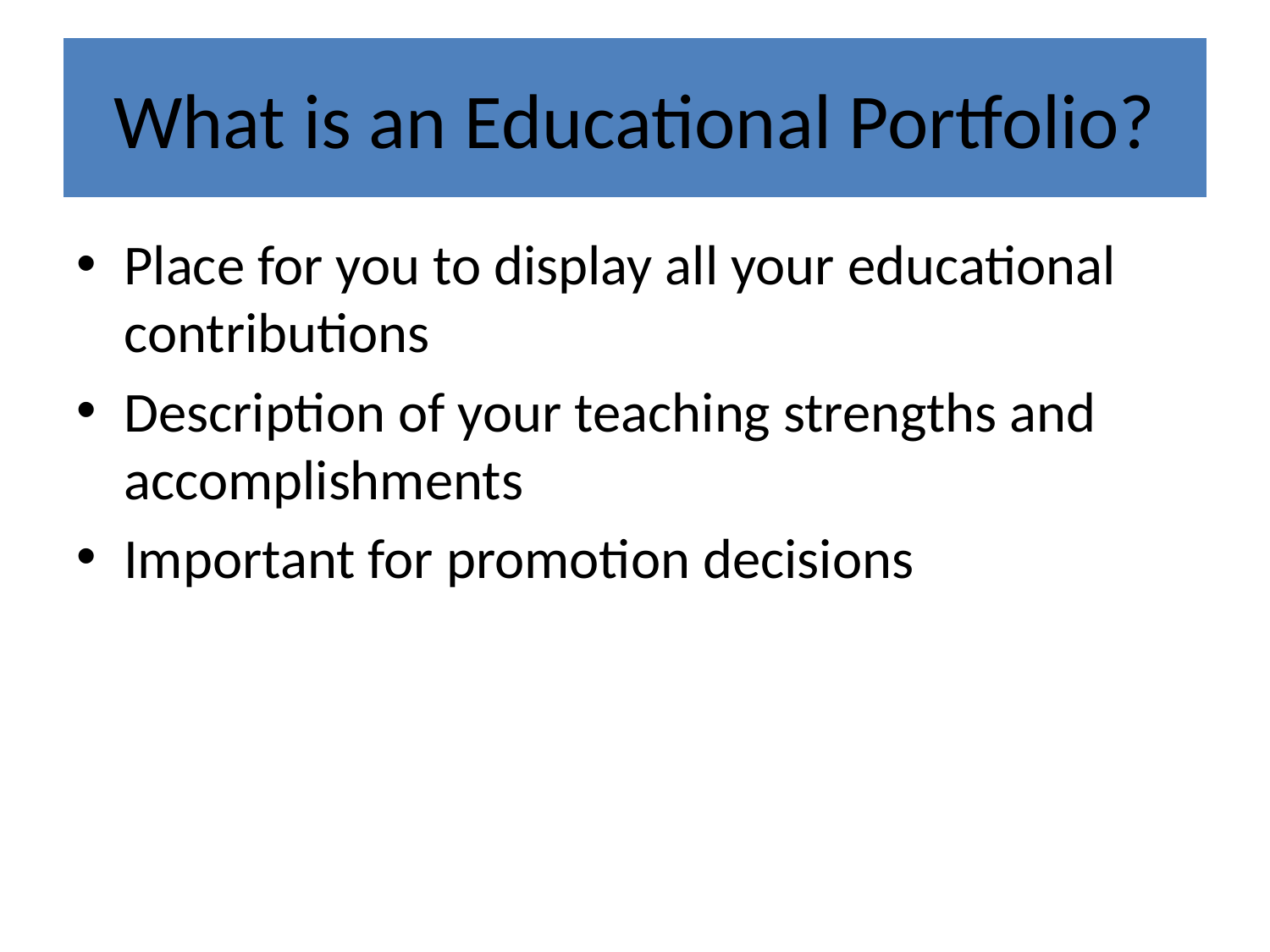

# What is an Educational Portfolio?
Place for you to display all your educational contributions
Description of your teaching strengths and accomplishments
Important for promotion decisions

## Slide 22
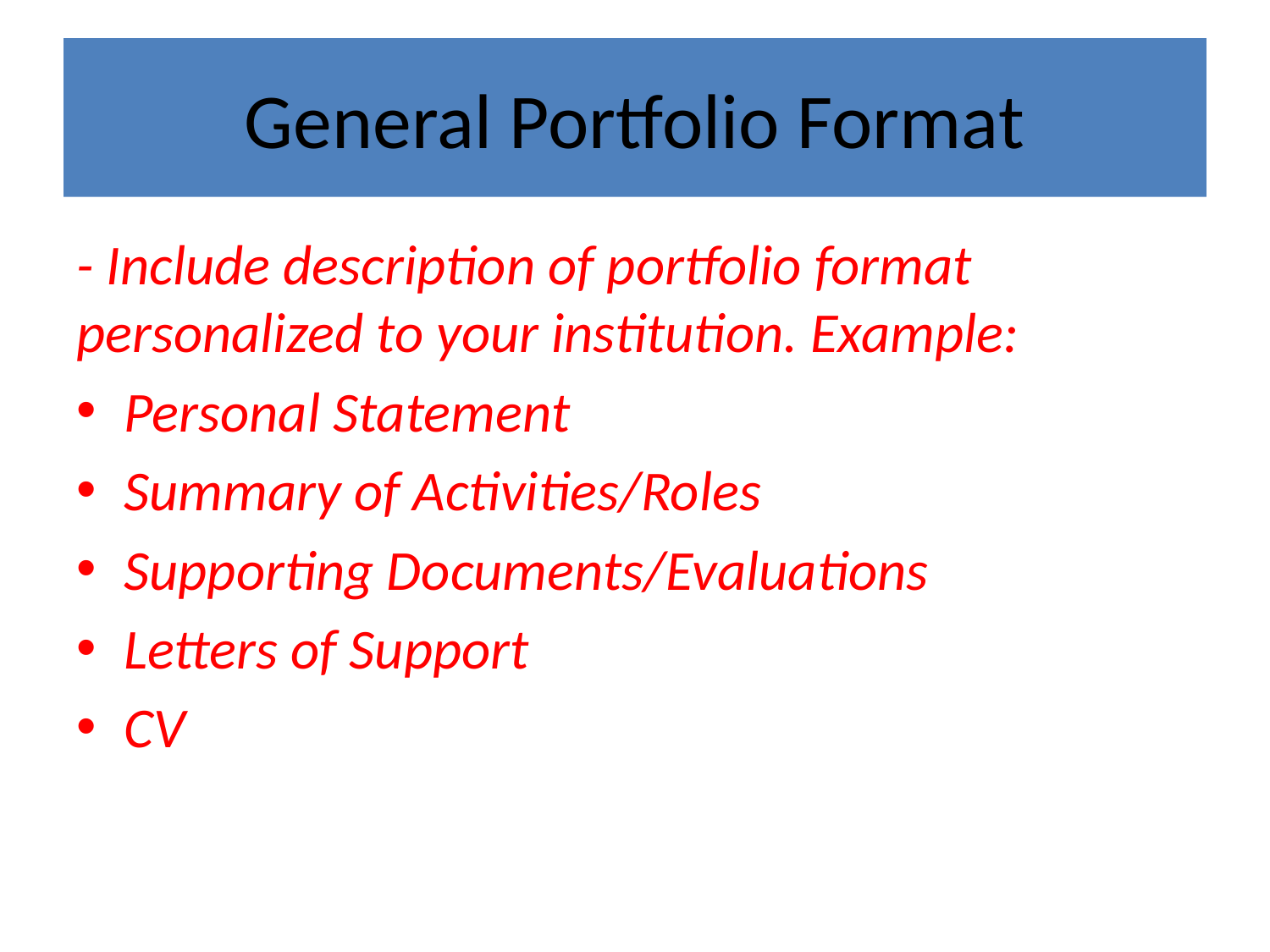

# General Portfolio Format
- Include description of portfolio format personalized to your institution. Example:
Personal Statement
Summary of Activities/Roles
Supporting Documents/Evaluations
Letters of Support
CV

## Slide 23
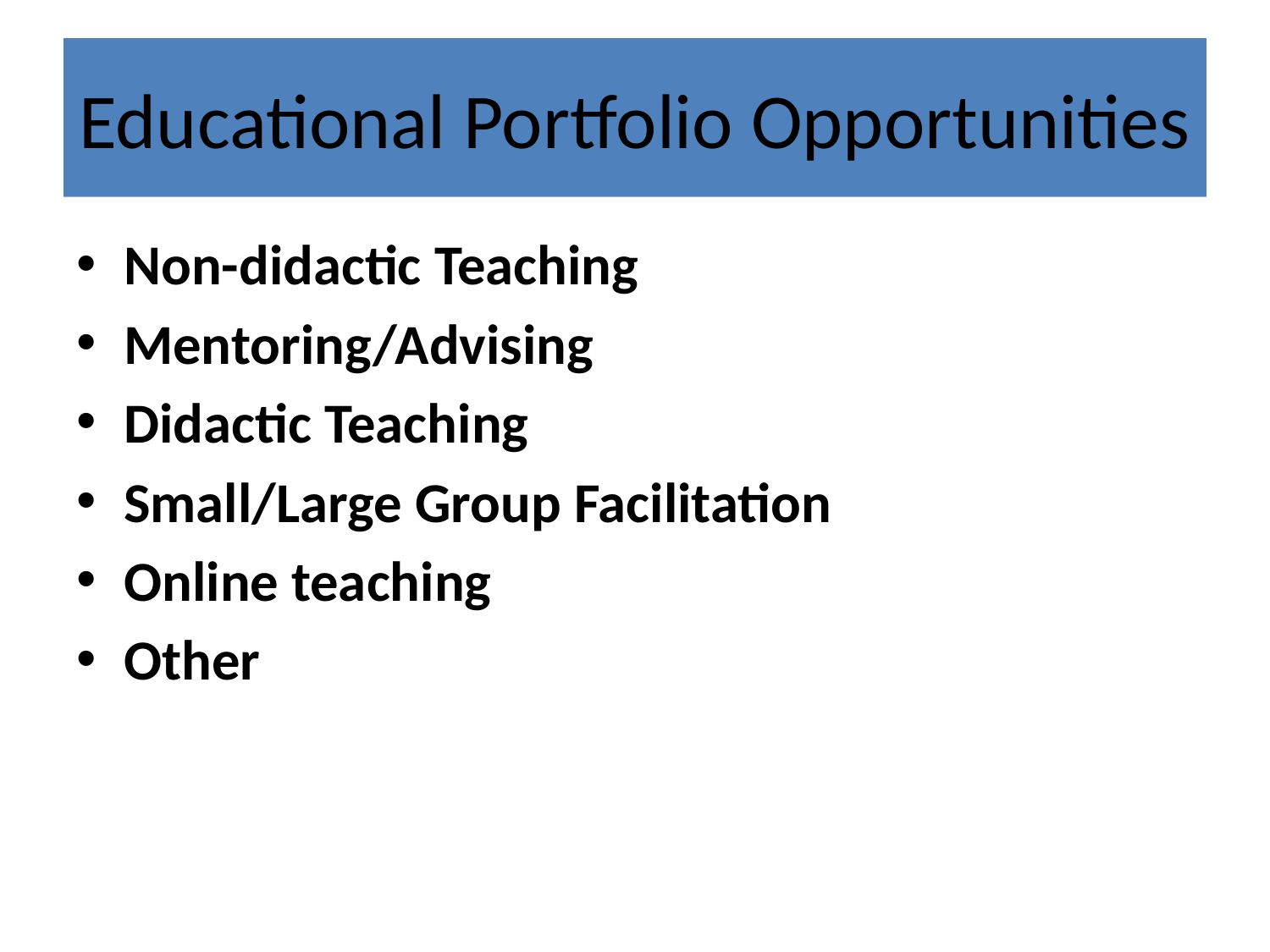

# Educational Portfolio Opportunities
Non-didactic Teaching
Mentoring/Advising
Didactic Teaching
Small/Large Group Facilitation
Online teaching
Other

## Slide 24
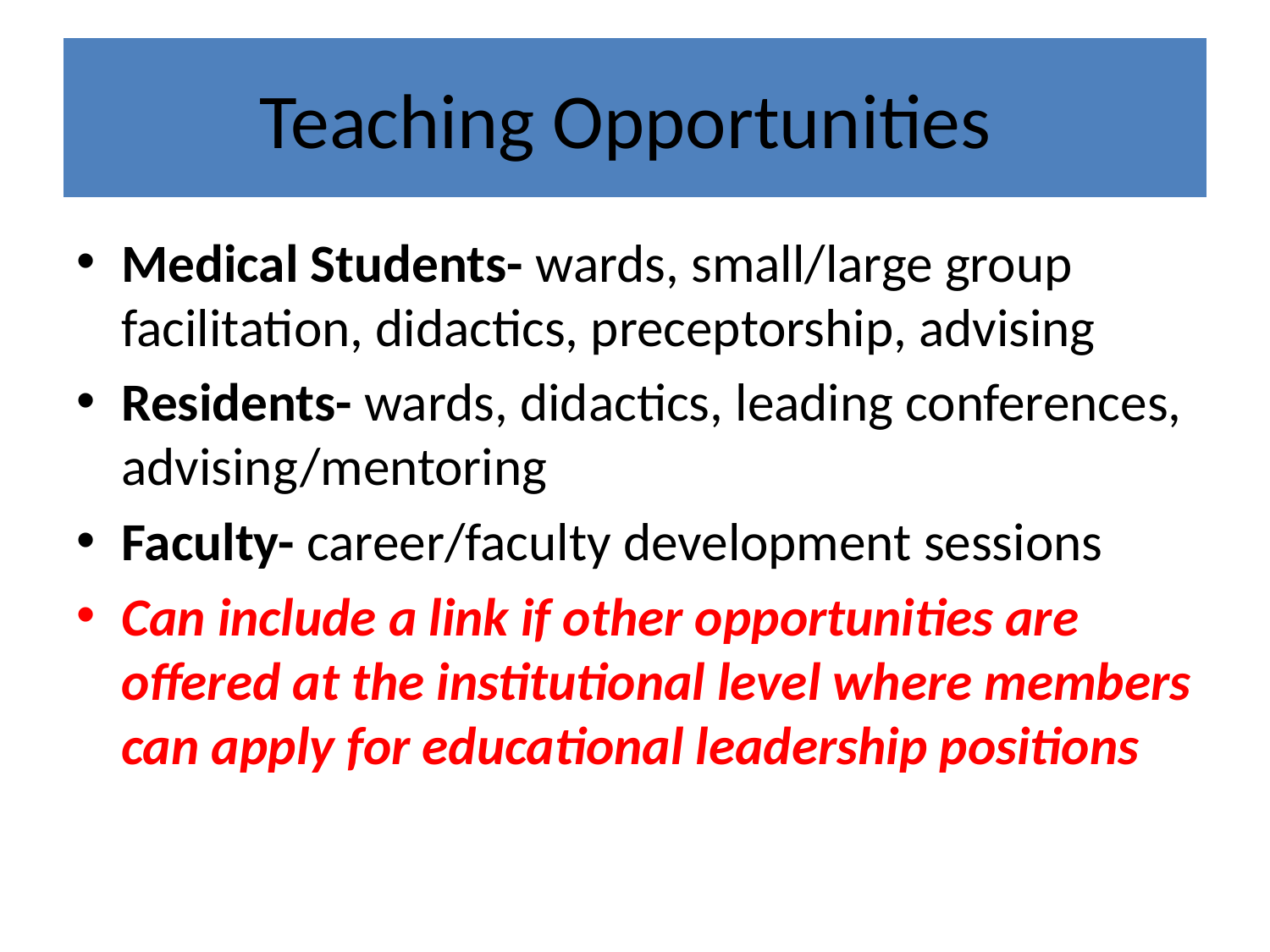

# Teaching Opportunities
Medical Students- wards, small/large group facilitation, didactics, preceptorship, advising
Residents- wards, didactics, leading conferences, advising/mentoring
Faculty- career/faculty development sessions
Can include a link if other opportunities are offered at the institutional level where members can apply for educational leadership positions

## Slide 25
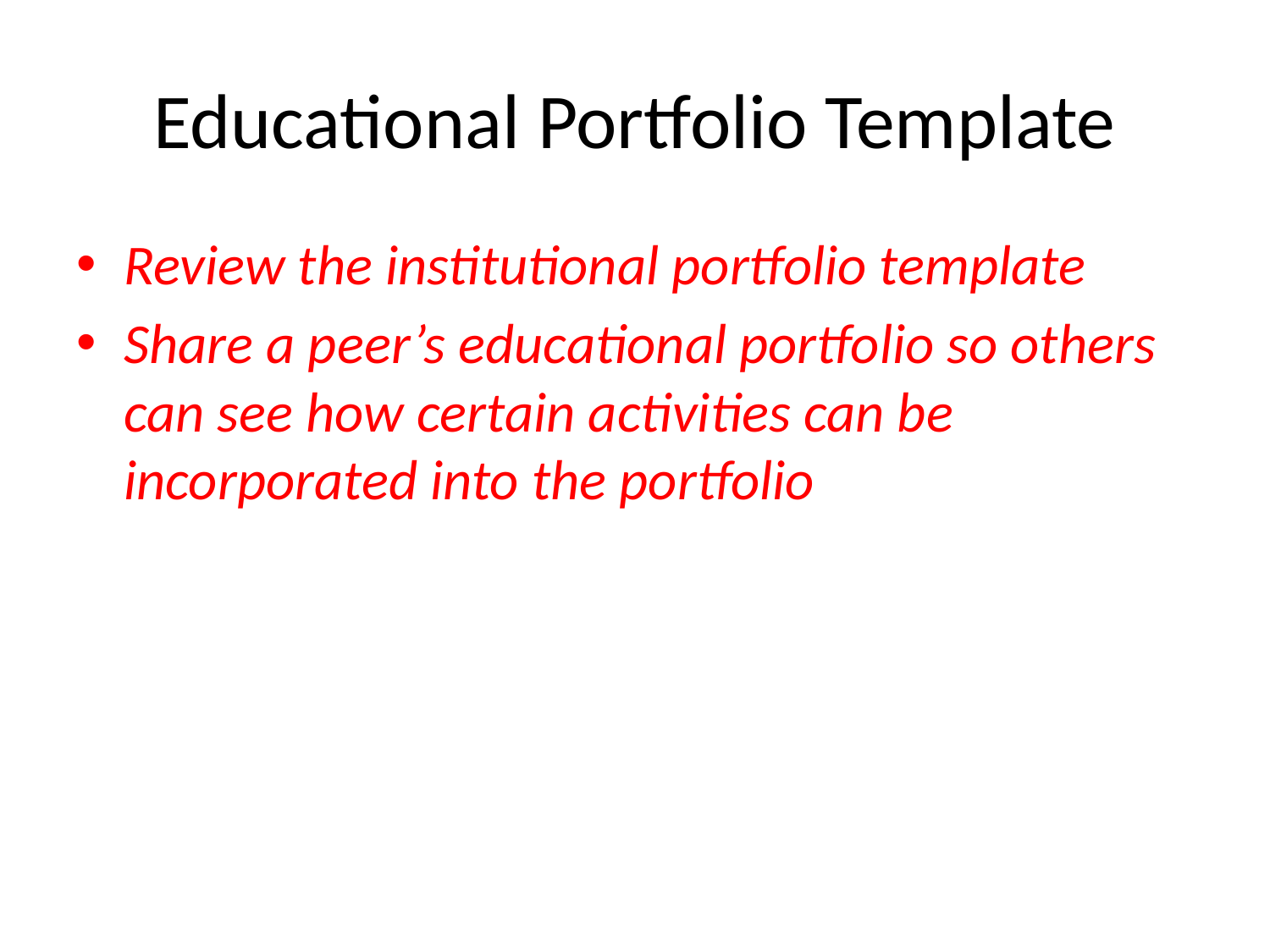

# Educational Portfolio Template
Review the institutional portfolio template
Share a peer’s educational portfolio so others can see how certain activities can be incorporated into the portfolio

## Slide 26
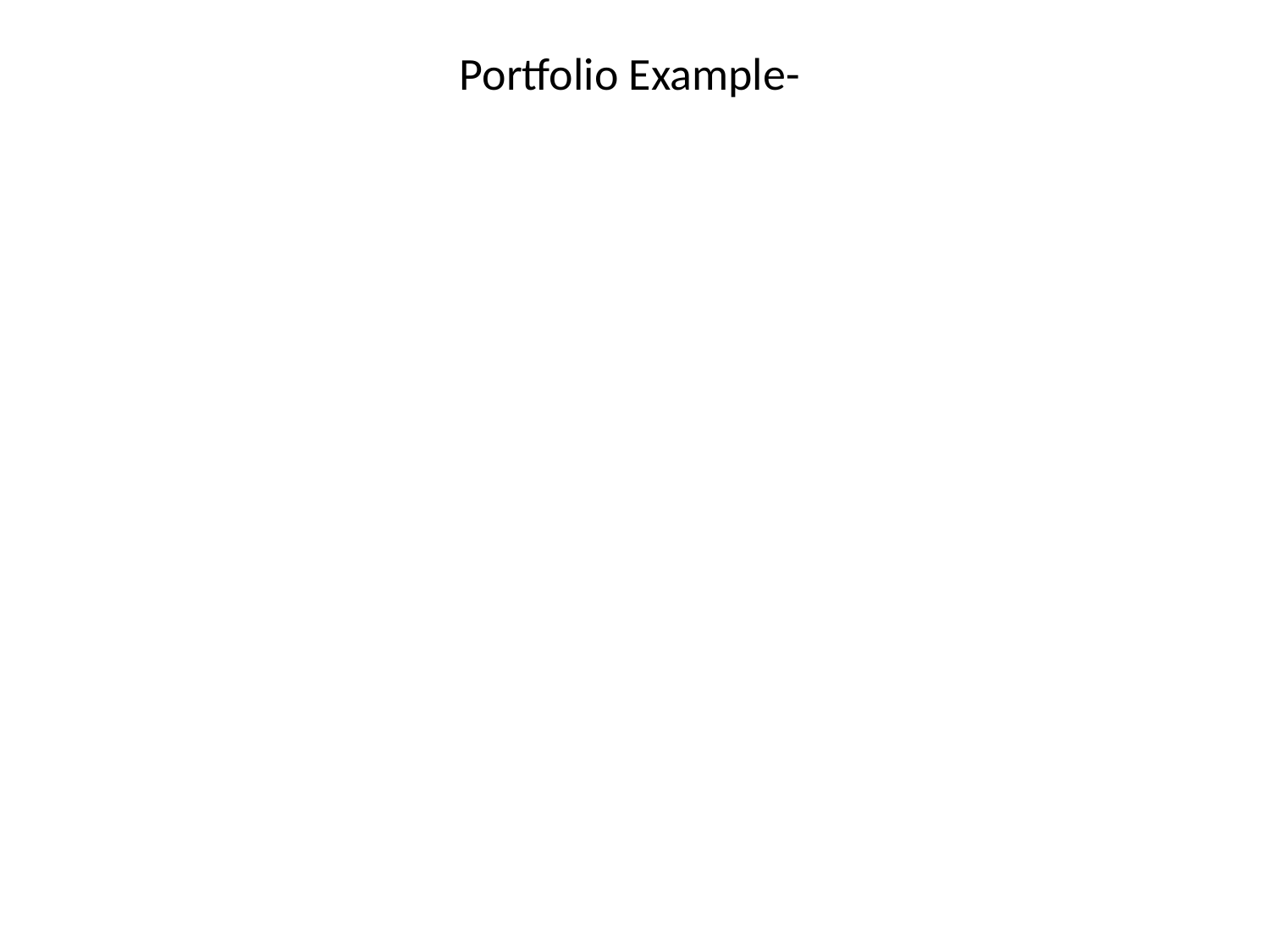

# Portfolio Example-

## Slide 27
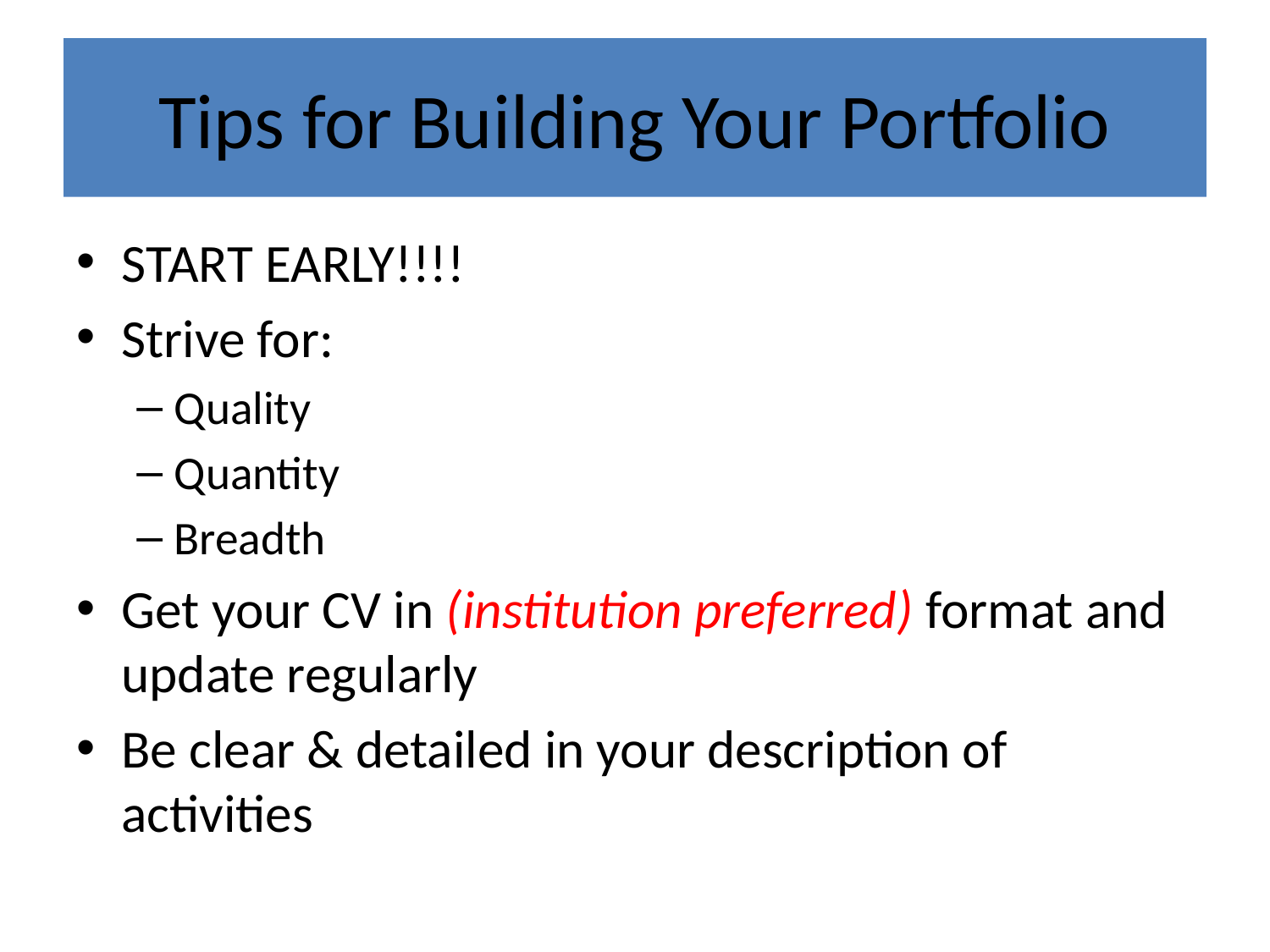

# Tips for Building Your Portfolio
START EARLY!!!!
Strive for:
Quality
Quantity
Breadth
Get your CV in (institution preferred) format and update regularly
Be clear & detailed in your description of activities

## Slide 28
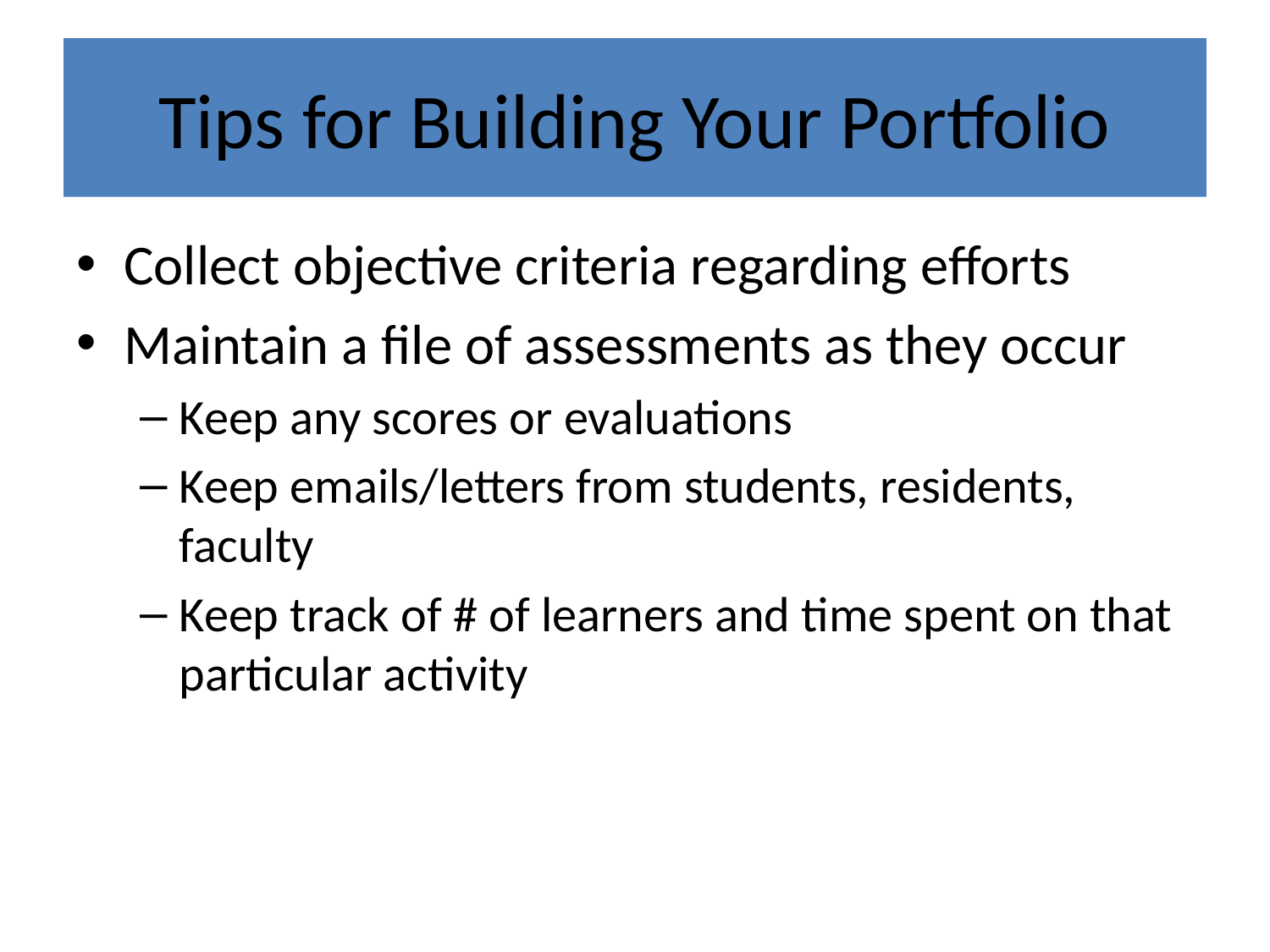

# Tips for Building Your Portfolio
Collect objective criteria regarding efforts
Maintain a file of assessments as they occur
Keep any scores or evaluations
Keep emails/letters from students, residents, faculty
Keep track of # of learners and time spent on that particular activity

## Slide 29
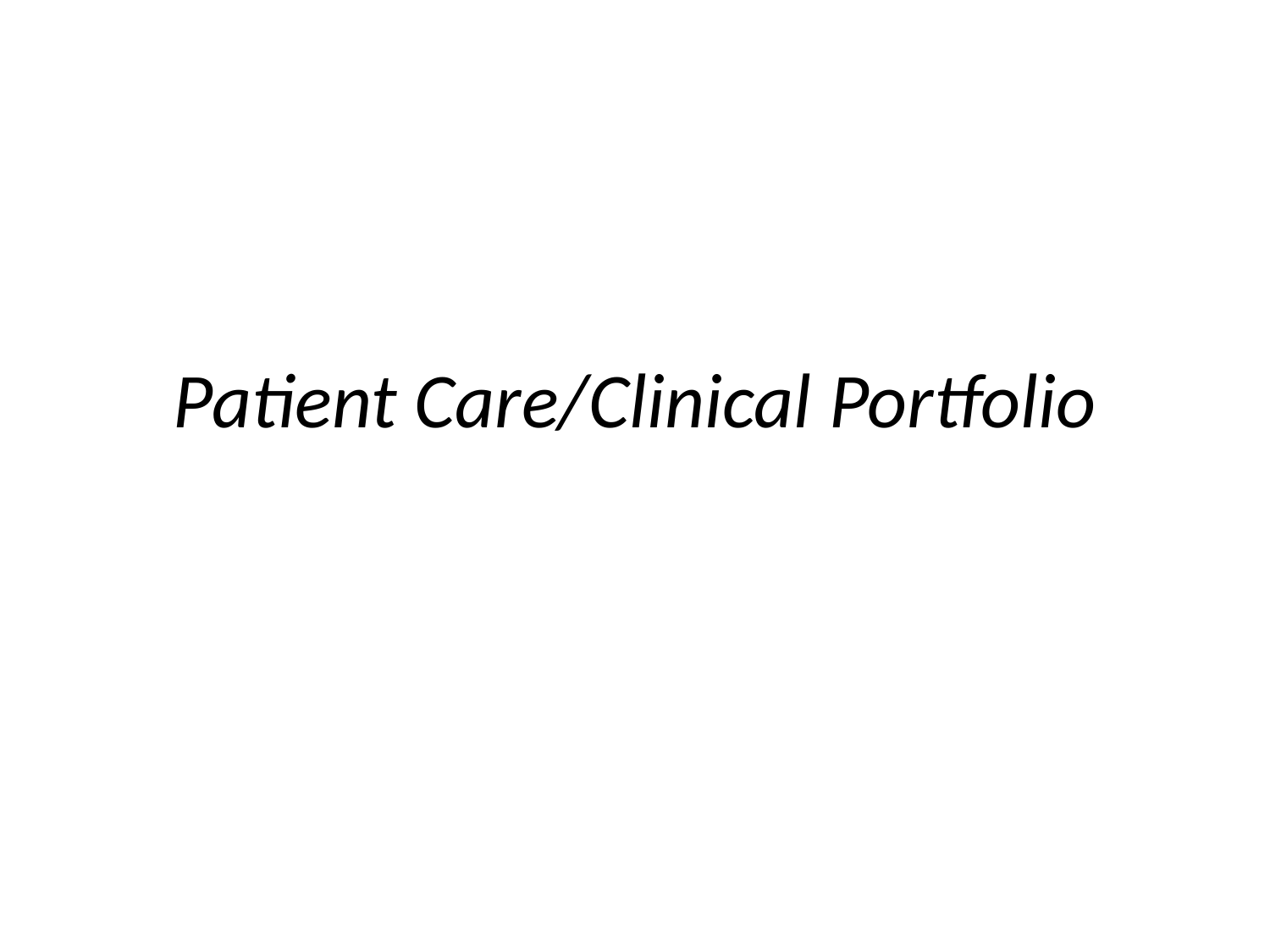

# Patient Care/Clinical Portfolio

## Slide 30
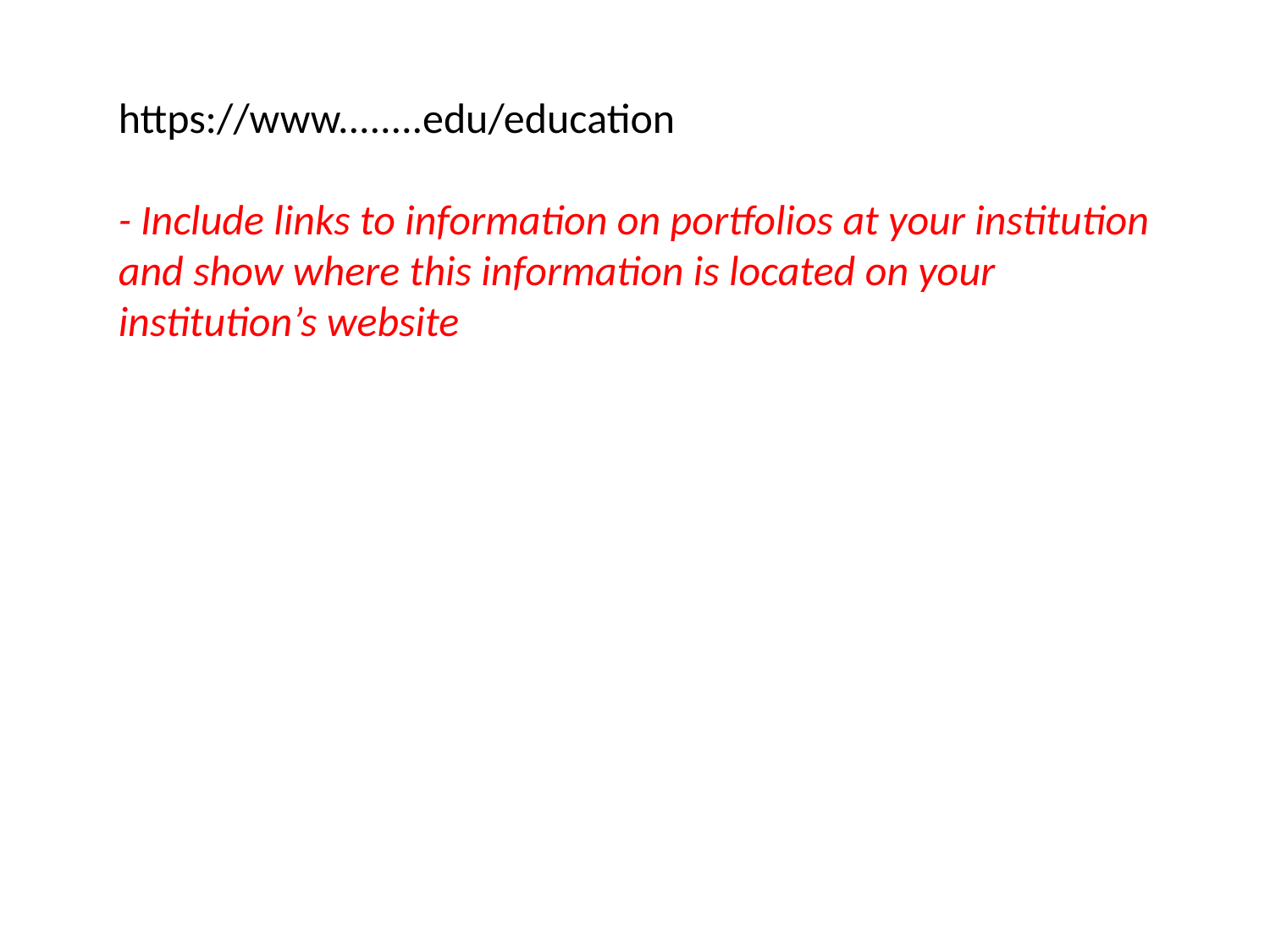

https://www........edu/education
- Include links to information on portfolios at your institution and show where this information is located on your institution’s website

## Slide 31
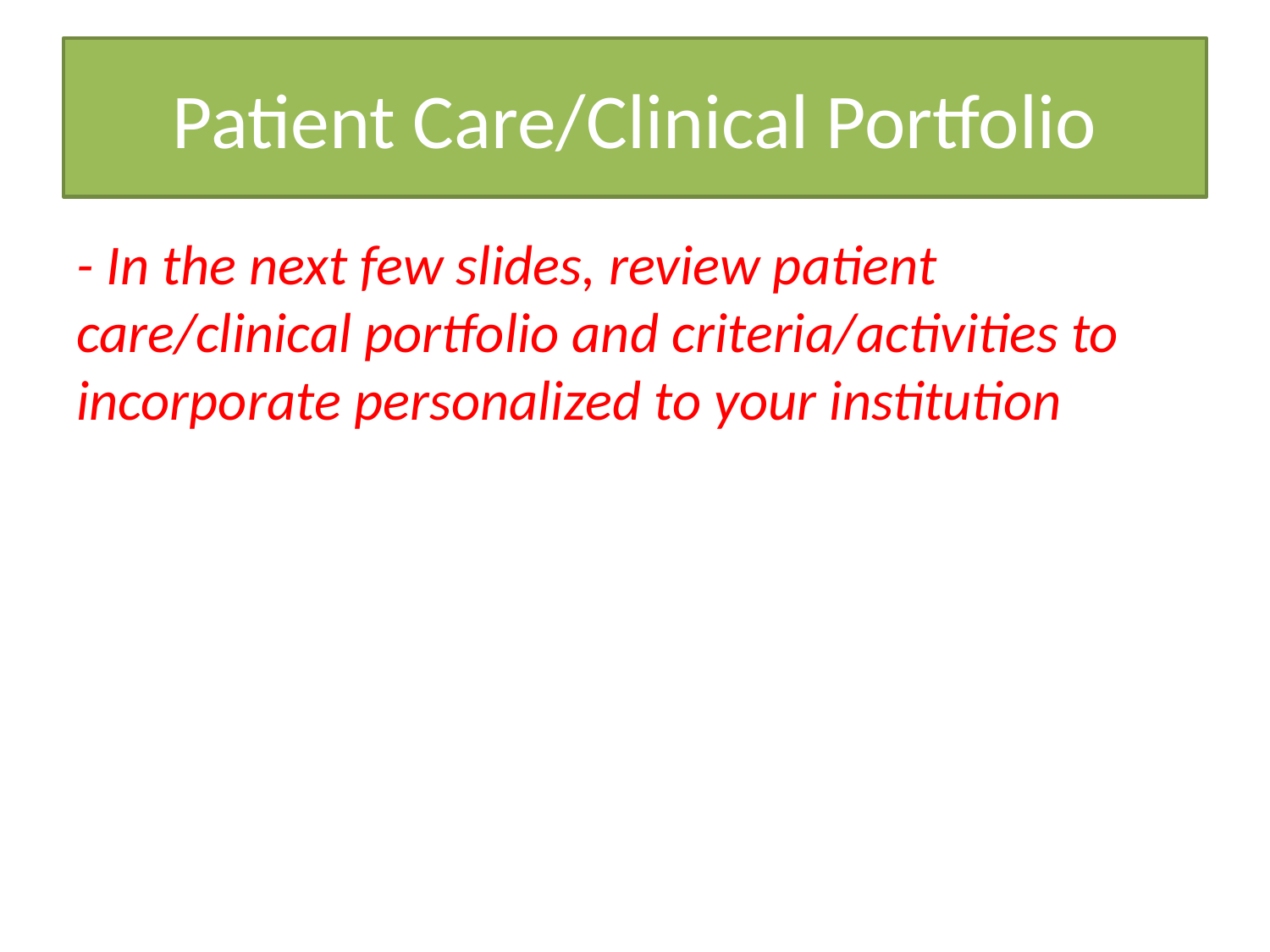

# Patient Care/Clinical Portfolio
- In the next few slides, review patient care/clinical portfolio and criteria/activities to incorporate personalized to your institution

## Slide 32
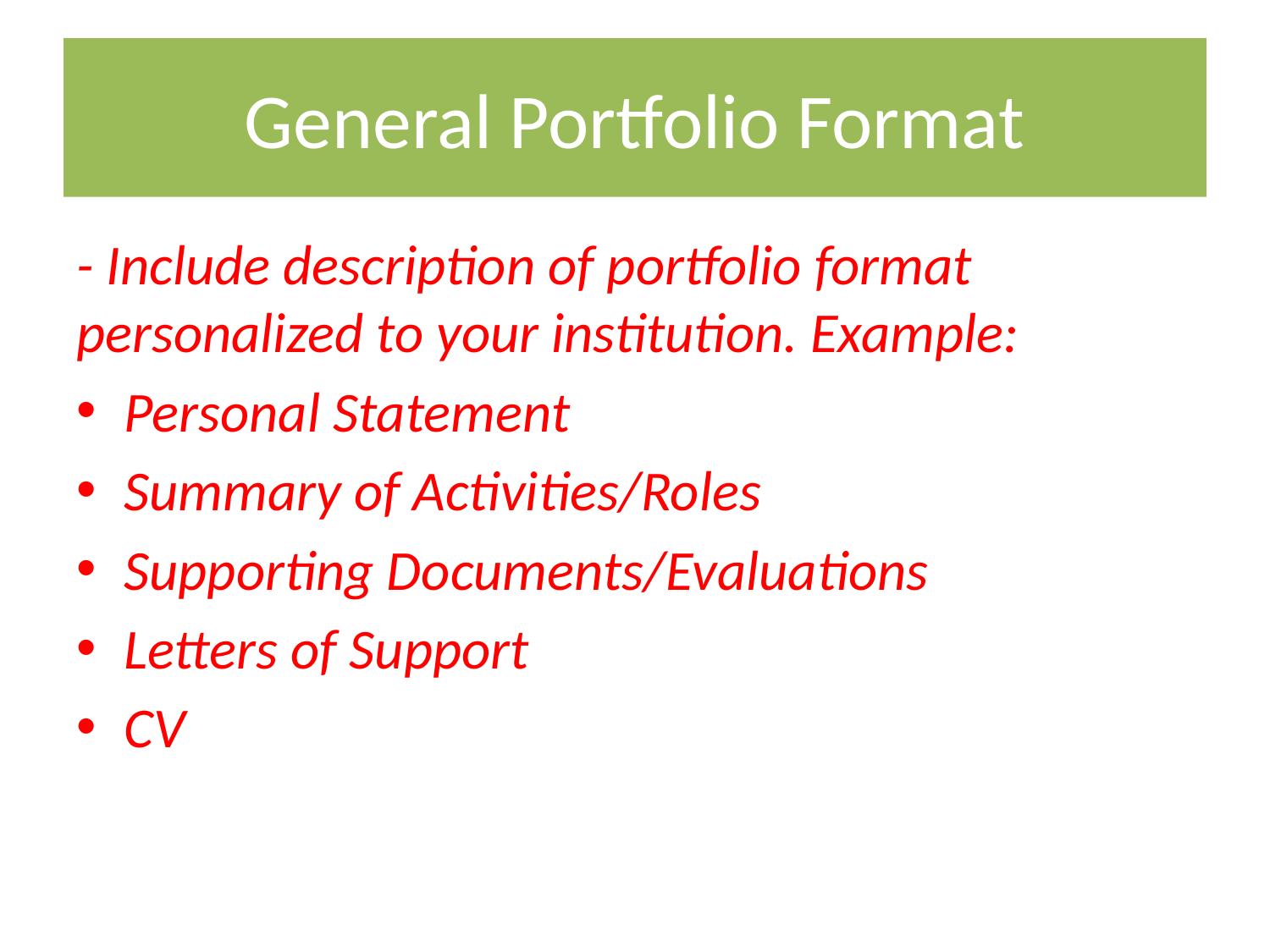

# General Portfolio Format
- Include description of portfolio format personalized to your institution. Example:
Personal Statement
Summary of Activities/Roles
Supporting Documents/Evaluations
Letters of Support
CV

## Slide 33
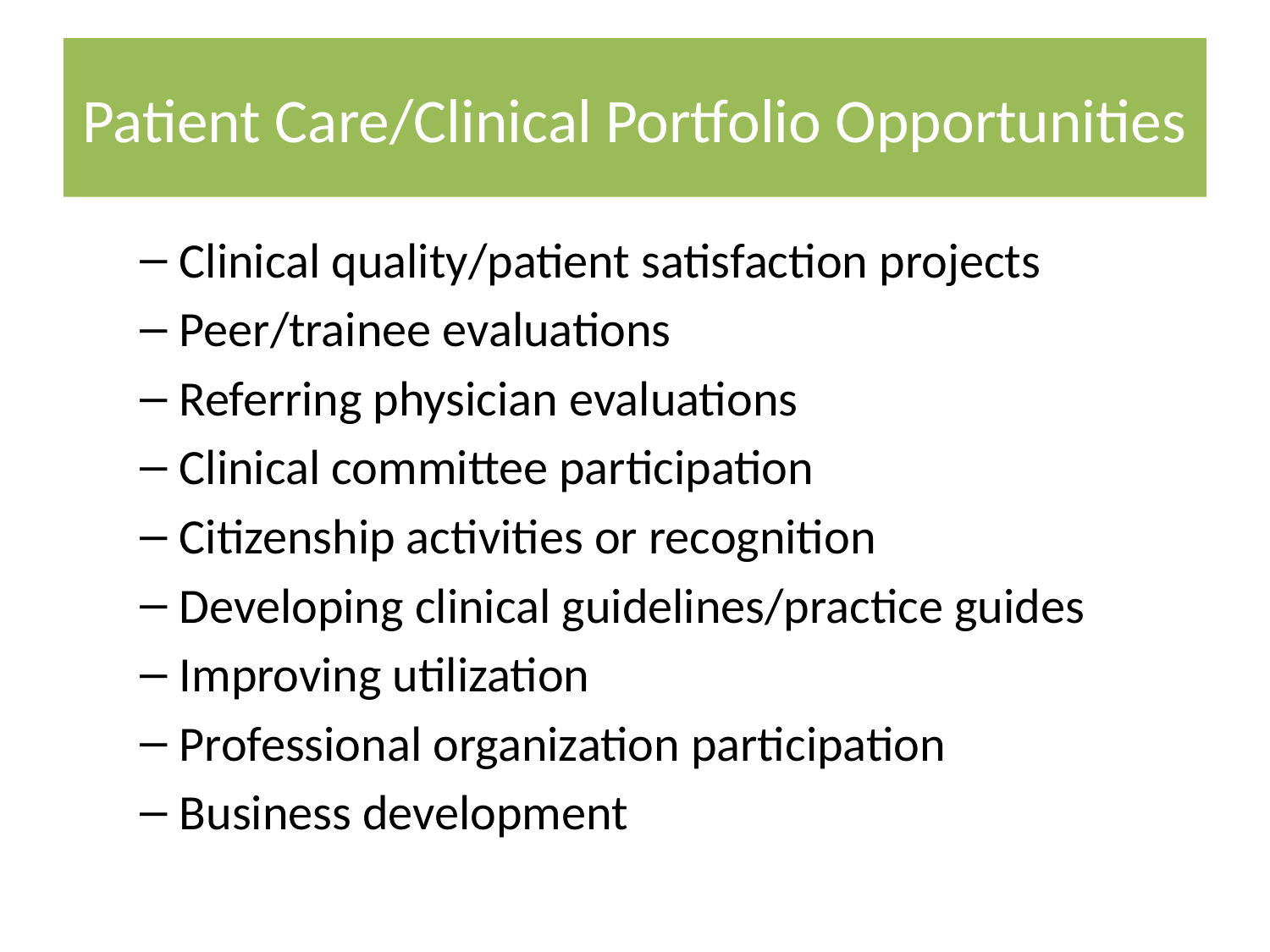

# Patient Care/Clinical Portfolio Opportunities
Clinical quality/patient satisfaction projects
Peer/trainee evaluations
Referring physician evaluations
Clinical committee participation
Citizenship activities or recognition
Developing clinical guidelines/practice guides
Improving utilization
Professional organization participation
Business development

## Slide 34
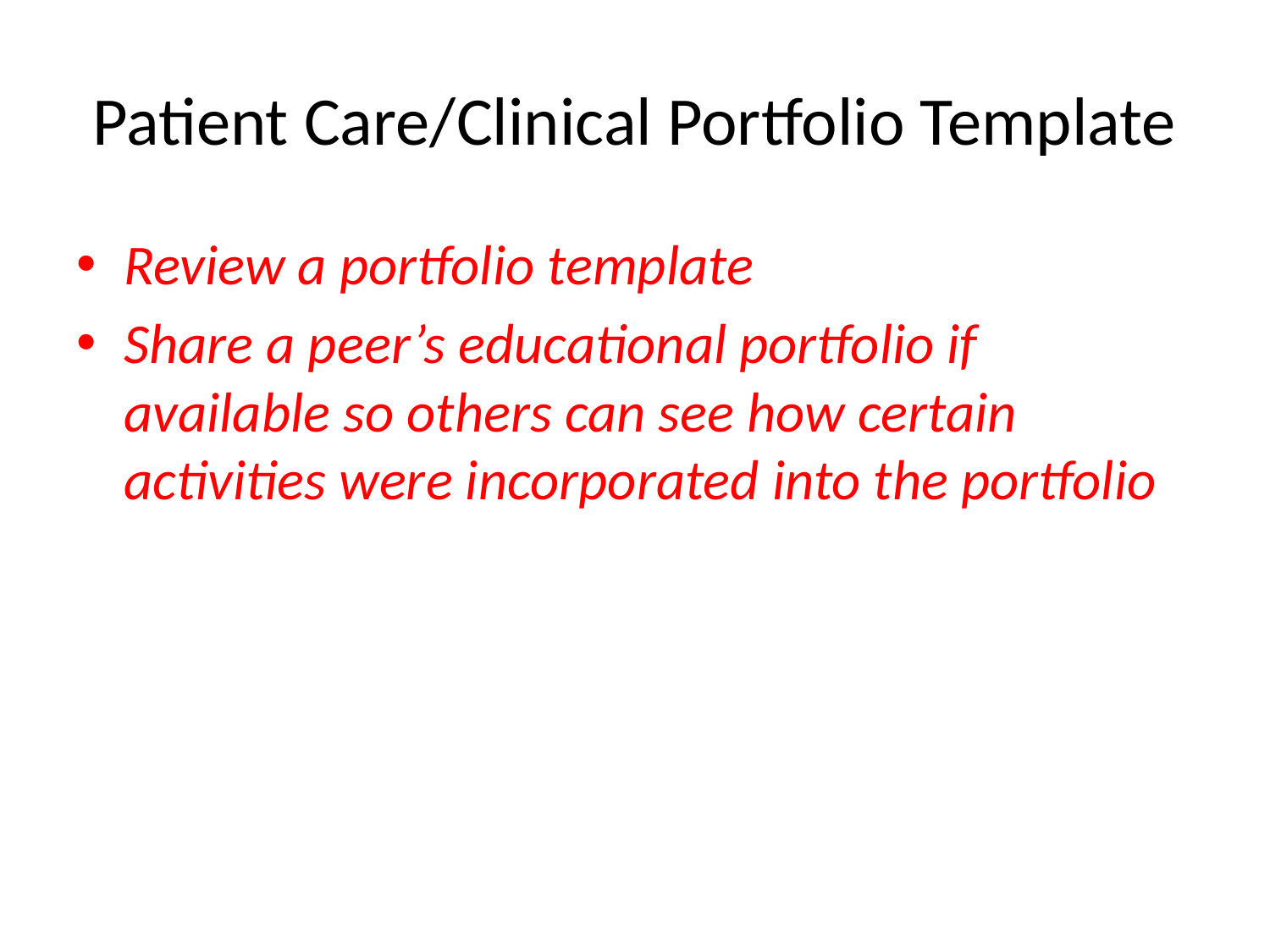

# Patient Care/Clinical Portfolio Template
Review a portfolio template
Share a peer’s educational portfolio if available so others can see how certain activities were incorporated into the portfolio

## Slide 35
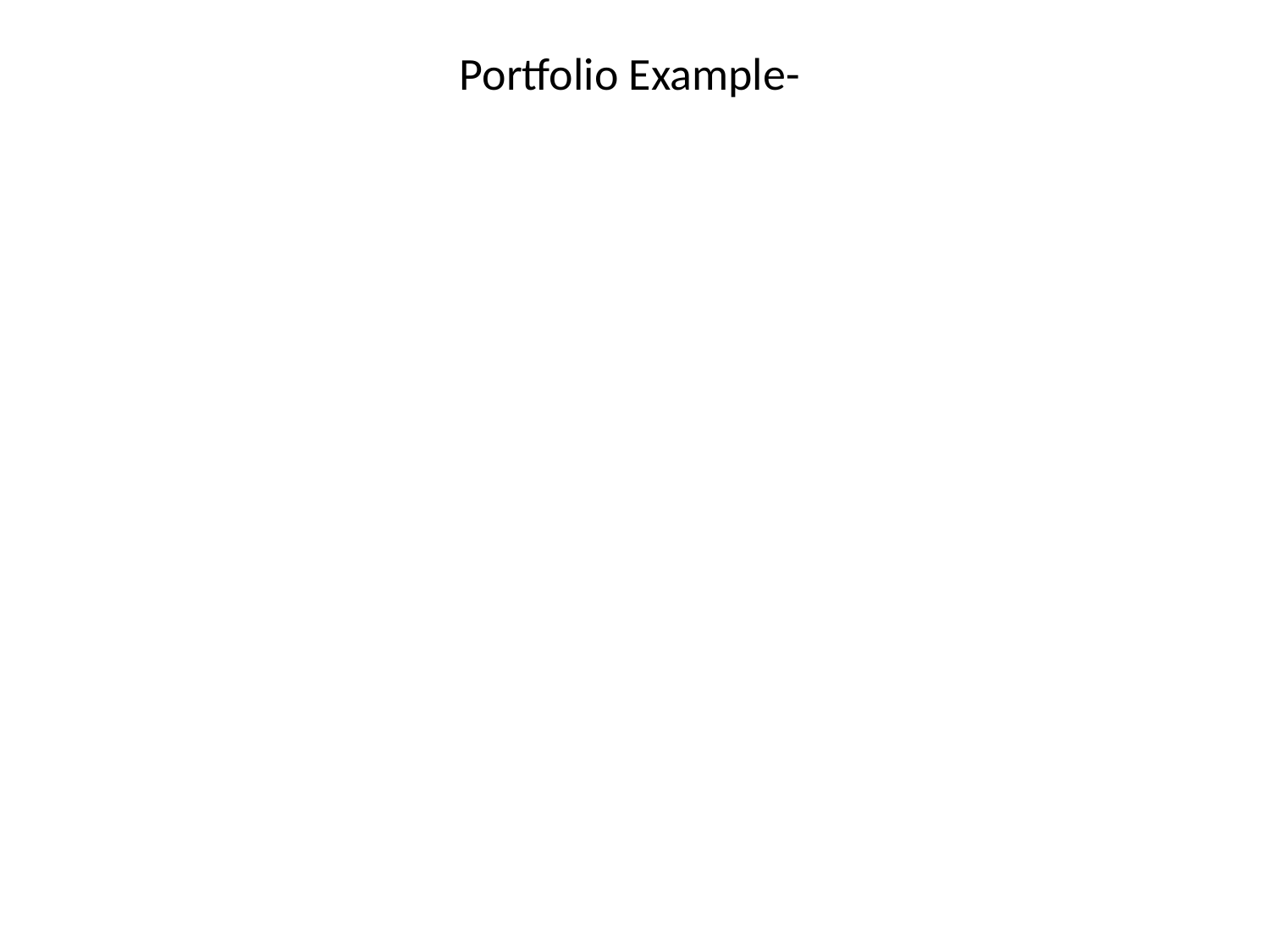

# Portfolio Example-

## Slide 36
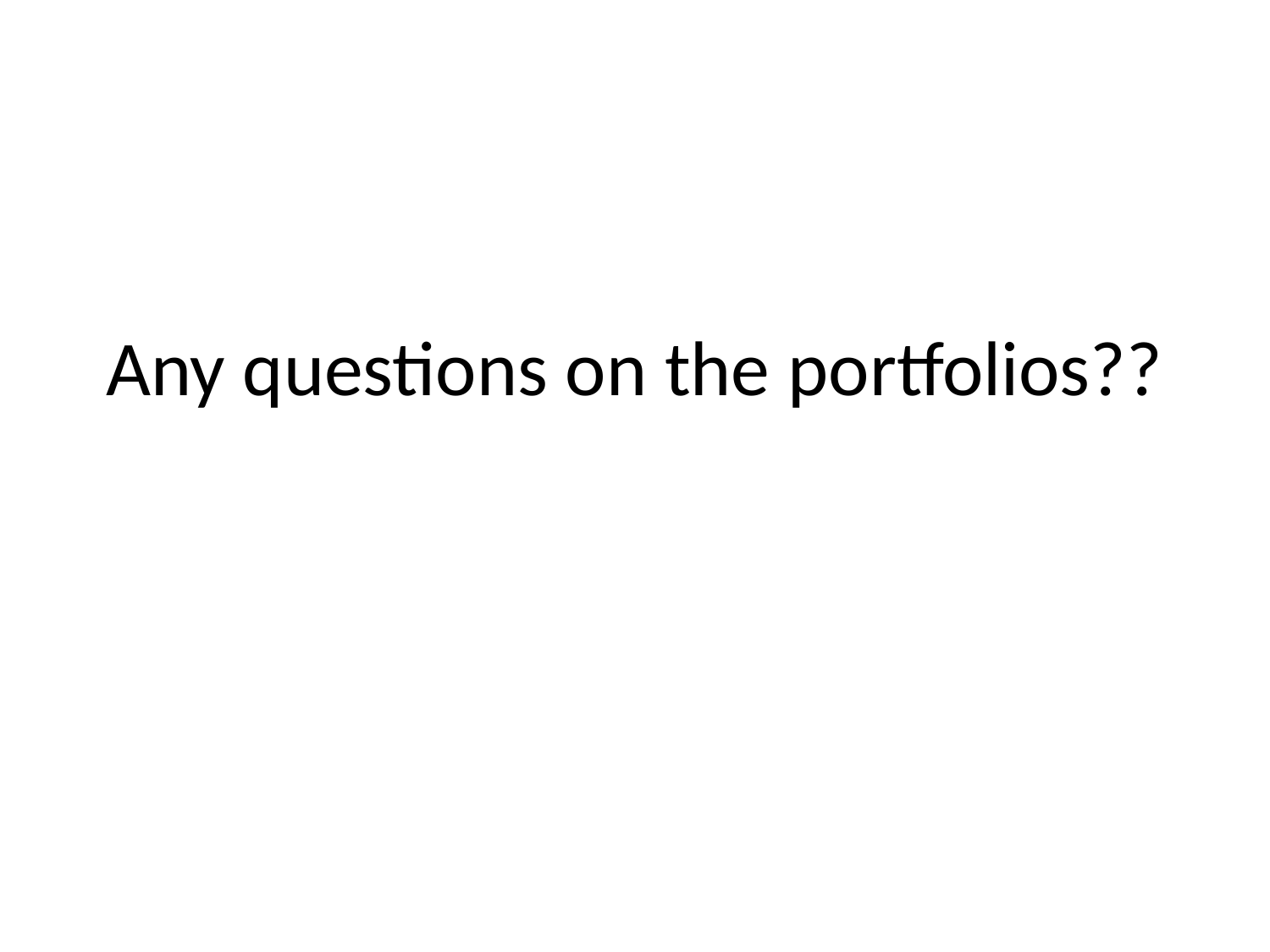

# Any questions on the portfolios??

## Slide 37
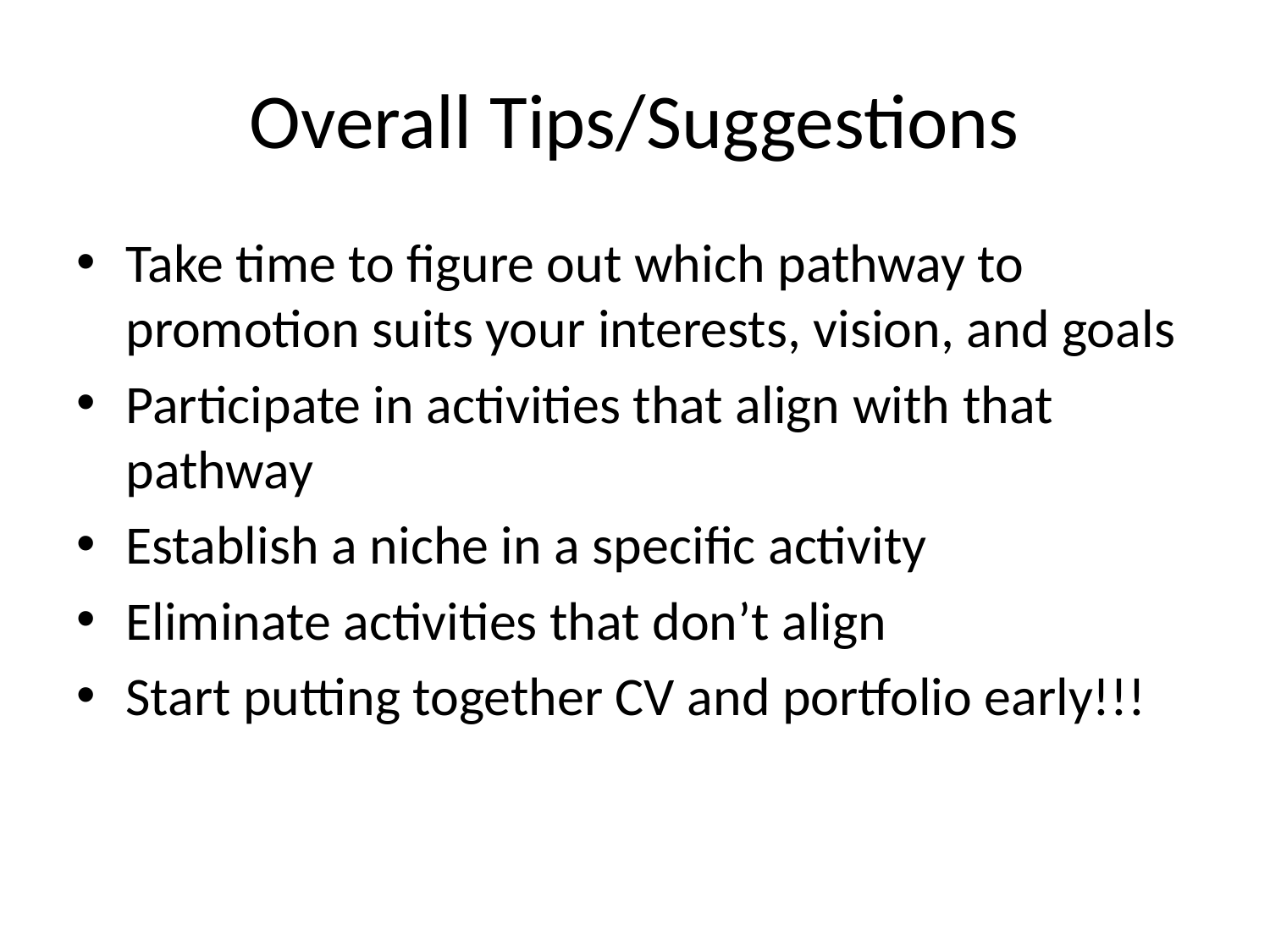

# Overall Tips/Suggestions
Take time to figure out which pathway to promotion suits your interests, vision, and goals
Participate in activities that align with that pathway
Establish a niche in a specific activity
Eliminate activities that don’t align
Start putting together CV and portfolio early!!!

## Slide 38
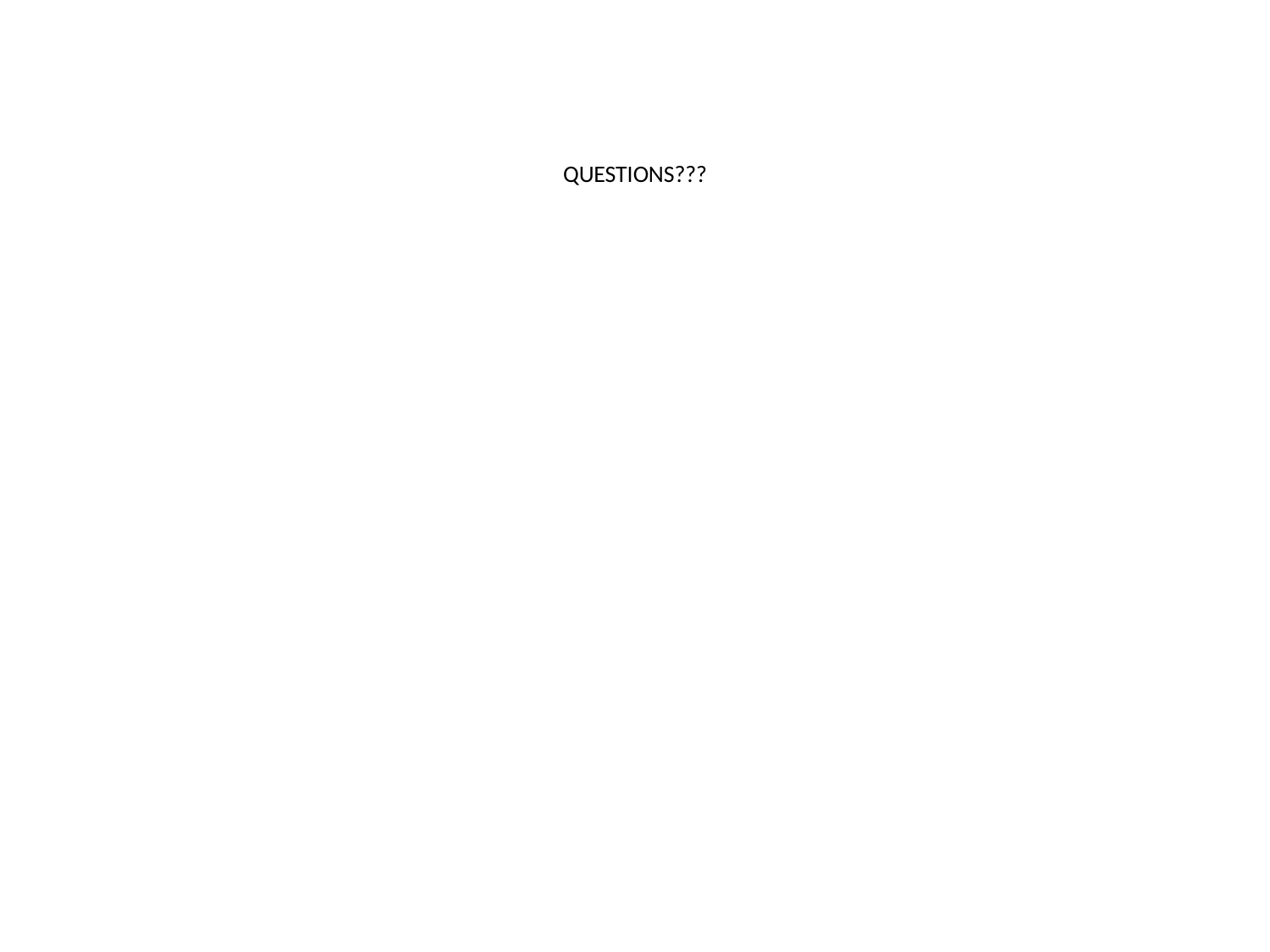

# QUESTIONS???
