# Supplementary material for: A Facilitated Peer Mentoring Program With a Dedicated Curriculum to Foster Career Advancement of Academic Hospitalists
Source: MedEdPORTAL. 2023 Dec 8;19:11366. doi: 10.15766/mep_2374-8265.11366 (PMC10704005; doi:10.15766/mep_2374-8265.11366)
Supplement: Supplementary file 1 — Preprogram Survey.docxPostprogram Survey.docxLarge-Group Session 1.pptxLarge-Group Session 2.pptxLarge-Group Session 3.pptxLarge-Group Session 4.pptxSmall-Group Session 1 Facilitator Guide.docxSmall-Group Session 2 Facilitator Guide.docxSmall-Group Session 3 Facilitator Guide.docx [file mep_2374-8265.11366-s001.zip › G. Small-Group Session 1 Facilitator Guide.docx]

**Appendix G. Facilitator Guide for Small Group Session #1**

**Introduction to Peer Mentorship and Identifying Career Goals**

**Goal/Objective:** Peer members will reflect on their own professional development to create a vision statement and confirm that their activities align with their vision

**Activity:**

Everyone should introduce themselves and discuss their professional interests and projects they may currently be working on.

**Facilitator will:**

1. Pass out the handout on next page to each participant
2. Have the participant create a professional vision statement which can be based on INSPIRE
3. Have the participant list short and long term goals- make sure they are **SMART** goals

**S**- Specific, **M**- Measurable, **A**- Attainable, **R**- Relevant, **T**- Time sensitive

1. Have the participant list activities they currently participate in including committees, projects, learner educational activities, etc.
2. Encourage member(s) to share their vision statement and whether their activities align with their goals.

**Peer members will:**

1. Reflect and create a professional vision statement
2. List short and long term goals
3. List activities that they currently participate in
4. Confirm that these activities align with their goals and ultimate vision for their career path

**Reference:**

Li ST, Frohna JG. Bostwick SB. Using your personal mission statement to INSPIRE and achieve success. *Acad Ped*. 2017;17(2):107-109.

**Vision Statement:**

**List Short Term Goals (1-2 years):**

**List Long Term Goals (5-10 years):**

**What Activities Do You Currently Participate In?**

| **Activities** | **Time Spent on Activity** |
| --- | --- |
|  |  |
|  |  |
|  |  |
|  |  |
|  |  |
|  |  |
|  |  |

**Ask yourself**-- Do all your activities align with your short and long term goals and your ultimate vision? If not, maybe spend less time on these activities. Maybe you have to say “no” to these opportunities. If activities do align, then these are the activities you need to focus more of your time and energy on.

The point is that many times we spend too much time on activities that either don’t align with our goals and interests, or don’t “count” towards anything meaningful in terms of career advancement or what you would like to achieve from participating in that activity.
