# Supplementary material for: A Facilitated Peer Mentoring Program With a Dedicated Curriculum to Foster Career Advancement of Academic Hospitalists
Source: MedEdPORTAL. 2023 Dec 8;19:11366. doi: 10.15766/mep_2374-8265.11366 (PMC10704005; doi:10.15766/mep_2374-8265.11366)
Supplement: Supplementary file 1 — Preprogram Survey.docxPostprogram Survey.docxLarge-Group Session 1.pptxLarge-Group Session 2.pptxLarge-Group Session 3.pptxLarge-Group Session 4.pptxSmall-Group Session 1 Facilitator Guide.docxSmall-Group Session 2 Facilitator Guide.docxSmall-Group Session 3 Facilitator Guide.docx [file mep_2374-8265.11366-s001.zip › H. Small-Group Session 2 Facilitator Guide.docx]

**Appendix H. Facilitator Guide for Small Group Session #2**

**Building a Curriculum Vitae**

**Goal/Objective:** Peer members will begin work and/or review their own CV and determine if it is up-to-date and in line with the institutional format

**Activity:**

**Facilitator will:**

1. Briefly review the institutional CV template (if available) and have peer members format their CV to match the template (a general outline is provided on the next page that can also be used as an example)
2. Instruct members to create a folder on their desktop/email for their CV and have it easily accessible so they can add activities as they occur
3. Ask participants to review the goals/activities they listed in their Vision Statement handout from Small Group Session #1 to help with CV preparation (and portfolios)
4. Encourage peers to ask questions and share how they may incorporate specific activities into their CVs

**Peer members will:**

1. Begin work and/or review their own CV
2. Create a folder in their email/desktop to store information that should go into a CV
3. Review their current activities (from Session #1) and discuss how these activities can be incorporated into their CV

**At the end, the facilitator should remind peer members to:**

1. Update their CVs monthly or as activities occur
2. Review it closely to avoid grammatical or spelling errors
3. Ensure it is clear and easy to read
4. Avoid jargon
5. Avoid acronyms or abbreviations that are not widely known

**EXAMPLE of an Academic Curriculum Vitae Outline**

**I. General Biographical Information**

**A. Personal**

1. Name

2. Office Address, Phone Number

3. Personal Data such as home address (optional)

**B. Education (include institution/location, degree, and dates of attendance)**

1. Undergraduate Education: city, state, degree, dates

2. Medical Education or Graduate Education: city, state, degree(s), dates

3. Postgraduate Training: residency, fellowship, etc.

**C. Licensure and Certification**

1. State(s) licensed: date, name, license number

2. Specialty Board(s): Board, specialty, date

**D. Academic Appointments (include title and dates of appointment)**

1. Current Faculty Position(s)

2. Previous Faculty Position(s)

**E. Other Advanced Training/Experience**

1. Other Specialized Training/Certifications

**F. Other Information**

1. Honors or Awards

2. Specialty Board Certifications

**II. Research Information**

**A. Research Support**

1. Title of Project, Name of Funding Agency, Dates

**B. Local, Regional, National Scientific and Professional Participation (include dates and titles)**

1. Editorial Contributions

2. Review Panels

3. Professional Societies

4. Invited Lectures, Presentations

**C. Publications**

1. Full Papers in Peer Reviewed Journals

2. Full Papers without Peer Review

3. Abstracts/Posters

4. Books

**III. Teaching Information**

**A. Educational Leadership Roles**

**B. Didactic Coursework (include dates, number of hours and approximate numbers of learners)**

1. Courses Taught

**C. Curriculum Development**

1. Course(s)/Curricula to Which Contributions Have Been Made

2. Role in Course/Curriculum Development

3. Audience(s) for Course(s)/Curricula Developed

**D. Non-didactic Teaching (current and prior)**

1. Resident Training (include estimate of time spent, numbers of learners)

2. Medical Student Mentoring (provide information as above)

3. Other Learners (Nursing students, Physician Assistant students, etc.)

**E. Faculty Development**

**F. Lectures and Presentations**

1. Local, Regional, National, International

**IV. Patient care and Clinical Contributions**

**A. Patient Care Responsibilities**

1. Hospitals or clinics

**B. Clinical Leadership**

**C. Volunteer Work**

**D. Contributions to Health Policy**

**V. SERVICE Contributions**

**A. Administrative Assignments and Committees**

**B. National, Regional or Local Participation in Professional or Voluntary Organizations**

**C. Other Pertinent Information (not included previously)**
