# Supplementary material for: A Facilitated Peer Mentoring Program With a Dedicated Curriculum to Foster Career Advancement of Academic Hospitalists
Source: MedEdPORTAL. 2023 Dec 8;19:11366. doi: 10.15766/mep_2374-8265.11366 (PMC10704005; doi:10.15766/mep_2374-8265.11366)
Supplement: Supplementary file 1 — Preprogram Survey.docxPostprogram Survey.docxLarge-Group Session 1.pptxLarge-Group Session 2.pptxLarge-Group Session 3.pptxLarge-Group Session 4.pptxSmall-Group Session 1 Facilitator Guide.docxSmall-Group Session 2 Facilitator Guide.docxSmall-Group Session 3 Facilitator Guide.docx [file mep_2374-8265.11366-s001.zip › I. Small-Group Session 3 Facilitator Guide.docx]

**Appendix I. Facilitator Guide for Small Group Session #3**

**Pathways to Promotion and Introduction to Educational and Patient Care/Clinical Portfolios**

**Goal/Objective:** Peer members will select the promotion pathway that is more aligned with their goals and begin work on the respective portfolio that corresponds with that pathway

**Activity:**

**Facilitator will:**

1. Ask each member which promotion pathway they are interested in
2. Briefly review the institutional portfolio templates (if available) that align with each pathway and have peer members begin work on their own portfolio
3. Show completed portfolio examples from colleagues (if available) to help peer members identify where their activities may fit into their own portfolios
4. Encourage members to have a draft of their CV and aligned portfolio filled out by the completion of the peer program

**Peer members will:**

1. Select a promotion pathway aligned with their goals/activities that they previously listed on their Vision Statement from Small Group Session #1
2. Begin work on the portfolio (educational vs patient care/clinical) that corresponds with their aligned pathway
3. Create a folder in their email/desktop to store information that should go into their portfolio (i.e., awards, evaluations, letters from learners, results from projects/curriculum, etc.)

**Facilitator should remind peer members to:**

1. Start their portfolios early
2. Establish a niche
3. Consider eliminating activities that don’t align with their pathway
4. Keep track and collect any evaluations/letters/emails that may show the quality of their activities and be used in their portfolio
5. Keep track of time to prepare/present their activities, number of learners, and date of the activity
6. Have a peer or senior faculty review their portfolio prior to submission
